# Supplementary material for: Reporting conflicts of interest in randomised trials of patient blood management interventions in patients requiring major surgery: a systematic review and meta-analysis
Source: BMJ Open. 2022 Aug 17;12(8):e054582. doi: 10.1136/bmjopen-2021-054582 (PMC9389106; doi:10.1136/bmjopen-2021-054582)
Supplement: Supplementary data [file bmjopen-2021-054582supp001.pdf]

BMJ Publishing Group Limited (BMJ) disclaims all liability and responsibility arising from any reliance placed on this supplemental material which has been supplied by the author(s)

Reporting bias in randomised trials of Patient Blood Management interventions in patients requiring major surgery: A Systematic review and Meta-analysis

Marius Roman MD, Oluwatomini Fashina, Sara Tomassini MRes, Riccardo Abbasciano MD, Florence Y Lai MPhil, Prof. Toby Richards MD, Prof. Gavin Murphy MD.

Supplementary Appendix

Contents

1 PRISMA abstract and manuscript checklists. ... 3
2 Search strategy ... 7
2.1 Search Strategy Restrictive vs. Liberal Transfusion ... 7
2.2 Search Strategy Tranexamic Acid ... 7
2.3 Search Strategy Iron Therapy ... 8
2.4 Search Strategy Point of Care testing ... 8
2.5 Search Strategy Cell Salvage ... 8
2.6 Search Strategy for Cost Effectiveness ... 10
3 PRISMA flow diagram (eFigure 1.) ... 12
4 Characteristics of included studies (eTable 1) ... 13
5 Risk of bias report and summary for included studies. (eFigure 2) ... 149
6 Secondary outcomes based on Author and Funding Conflicts of Interest. (eTable 2) ... 165
7 Subgroup analysis based on studies that reported their primary outcome as clinical or transfusion related. (eTable 3) ... 176
8 Subgroup analysis for mortality and risk of red blood cells transfusion based on the country of origin of the corresponding author. (eTable 4.) ... 178
9 Subgroup analysis for mortality and risk of red blood cells transfusion based on the studies following the International Committee of Medical Journal Editors (ICMJE) guidelines of reporting. (eTable 5.) ... 179
10 Subgroup analysis for mortality and risk of red blood cells transfusion based on studies being published prior or after 2010 (Epoch) (eTable 6.) ... 180
11 Hidden Conflict of Interest. (eTable 7.) ... 181
12 Sensitivity analysis for mortality and risk of red blood cells transfusion for studies re-classified based on potential undeclared conflicts of interest. (eTable 8.) ... 182
13 Sensitivity analysis for mortality and risk of red blood cells transfusion excluding all studies considered at high or unclear risk of selection (allocation) bias (eTable 9.) ... 184
14 Funnel plots for Mortality and Rate of red blood cells transfusions (eFigure 3.) ... 186
14.1 Mortality - Author COI ... 186
14.2 Mortality – Type of funding ... 188
14.3 Rate of Red blood cells transfusion - Author COI ... 190
14.4 Rate of Red blood cells transfusion - Type of funding ... 192
15 References ... 194

1 PRISMA abstract and manuscript checklists.

PRISMA checklist of items to include in the abstract and manuscript when reporting a systematic review.

Section and Topic	Item #	Checklist item	Reported (Yes/No)
**TITLE**			
Title	1	Identify the report as a systematic review.	Yes
**BACKGROUND**			
Objectives	2	Provide an explicit statement of the main objective(s) or question(s) the review addresses.	Yes
**METHODS**			
Eligibility criteria	3	Specify the inclusion and exclusion criteria for the review.	Yes
Information sources	4	Specify the information sources (e.g. databases, registers) used to identify studies and the date when each was last searched.	Yes
Risk of bias	5	Specify the methods used to assess risk of bias in the included studies.	Yes
Synthesis of results	6	Specify the methods used to present and synthesise results.	Yes
**RESULTS**			
Included studies	7	Give the total number of included studies and participants and summarise relevant characteristics of studies.	Yes
Synthesis of results	8	Present results for main outcomes, preferably indicating the number of included studies and participants for each. If meta-analysis was done, report the summary estimate and confidence/credible interval. If comparing groups, indicate the direction of the effect (i.e. which group is favoured).	Yes
**DISCUSSION**			
Limitations of evidence	9	Provide a brief summary of the limitations of the evidence included in the review (e.g. study risk of bias, inconsistency and imprecision).	Yes
Interpretation	10	Provide a general interpretation of the results and important implications.	Yes
**OTHER**			
Funding	11	Specify the primary source of funding for the review.	Yes
Registration	12	Provide the register name and registration number.	Yes

Section and Topic	Item #	Checklist item	Location where item is reported
**TITLE**			
Title	1	Identify the report as a systematic review.	**1**
**ABSTRACT**			
Abstract	2	See the PRISMA 2020 for Abstracts checklist.	**2**
**INTRODUCTION**			
Rationale	3	Describe the rationale for the review in the context of existing knowledge.	**5**
Objectives	4	Provide an explicit statement of the objective(s) or question(s) the review addresses.	**5**
**METHODS**			
Eligibility criteria	5	Specify the inclusion and exclusion criteria for the review and how studies were grouped for the syntheses.	**6**
Information sources	6	Specify all databases, registers, websites, organisations, reference lists and other sources searched or consulted to identify studies. Specify the date when each source was last searched or consulted.	**6**
Search strategy	7	Present the full search strategies for all databases, registers and websites, including any filters and limits used.	**Supp 8-12**
Selection process	8	Specify the methods used to decide whether a study met the inclusion criteria of the review, including how many reviewers screened each record and each report retrieved, whether they worked independently, and if applicable, details of automation tools used in the process.	**8**
Data collection process	9	Specify the methods used to collect data from reports, including how many reviewers collected data from each report, whether they worked independently, any processes for obtaining or confirming data from study investigators, and if applicable, details of automation tools used in the process.	**9**
Data items	10a	List and define all outcomes for which data were sought. Specify whether all results that were compatible with each outcome domain in each study were sought (e.g. for all measures, time points, analyses), and if not, the methods used to decide which results to collect.	**8**
	10b	List and define all other variables for which data were sought (e.g. participant and intervention characteristics, funding sources). Describe any assumptions made about any missing or unclear information.	**6, 7, 9**
Study risk of bias assessment	11	Specify the methods used to assess risk of bias in the included studies, including details of the tool(s) used, how many reviewers assessed each study and whether they worked independently, and if applicable, details of automation tools used in the process.	**8, 9**
Effect measures	12	Specify for each outcome the effect measure(s) (e.g. risk ratio, mean difference) used in the synthesis or presentation of results.	**9**
Synthesis methods	13a	Describe the processes used to decide which studies were eligible for each synthesis (e.g. tabulating the study intervention characteristics and comparing against the planned groups for each synthesis (item #5)).	**6**
	13b	Describe any methods required to prepare the data for presentation or synthesis, such as handling of missing summary statistics, or data conversions.	**Previous publication**
	13c	Describe any methods used to tabulate or visually display results of individual studies and syntheses.	**Previous publication**
	13d	Describe any methods used to synthesize results and provide a rationale for the choice(s). If meta-analysis was performed, describe the model(s), method(s) to identify the presence and extent of statistical heterogeneity, and software package(s) used.	**Previous publication**
	13e	Describe any methods used to explore possible causes of heterogeneity among study results (e.g. subgroup analysis, meta-regression).	**9**

Section and Topic	Item #	Checklist item	Location where item is reported
	13f	Describe any sensitivity analyses conducted to assess robustness of the synthesized results.	**9, 10**
Reporting bias assessment	14	Describe any methods used to assess risk of bias due to missing results in a synthesis (arising from reporting biases).	**10**
Certainty assessment	15	Describe any methods used to assess certainty (or confidence) in the body of evidence for an outcome.	**9**
**RESULTS**			
Study selection	16a	Describe the results of the search and selection process, from the number of records identified in the search to the number of studies included in the review, ideally using a flow diagram.	**11**
	16b	Cite studies that might appear to meet the inclusion criteria, but which were excluded, and explain why they were excluded.	**Previous publication**
Study characteristics	17	Cite each included study and present its characteristics.	**Supplement**
Risk of bias in studies	18	Present assessments of risk of bias for each included study.	**Supplement**
Results of individual studies	19	For all outcomes, present, for each study: (a) summary statistics for each group (where appropriate) and (b) an effect estimate and its precision (e.g. confidence/credible interval), ideally using structured tables or plots.	**N/A**
Results of syntheses	20a	For each synthesis, briefly summarise the characteristics and risk of bias among contributing studies.	**Supplement**
	20b	Present results of all statistical syntheses conducted. If meta-analysis was done, present for each the summary estimate and its precision (e.g. confidence/credible interval) and measures of statistical heterogeneity. If comparing groups, describe the direction of the effect.	**11-12**
	20c	Present results of all investigations of possible causes of heterogeneity among study results.	**13, Supplement**
	20d	Present results of all sensitivity analyses conducted to assess the robustness of the synthesized results.	**13, Supplement**
Reporting biases	21	Present assessments of risk of bias due to missing results (arising from reporting biases) for each synthesis assessed.	**Supplement**
Certainty of evidence	22	Present assessments of certainty (or confidence) in the body of evidence for each outcome assessed.	**Previous publication**
**DISCUSSION**			
Discussion	23a	Provide a general interpretation of the results in the context of other evidence.	**14, 15**
	23b	Discuss any limitations of the evidence included in the review.	**16, 17**
	23c	Discuss any limitations of the review processes used.	**16**
	23d	Discuss implications of the results for practice, policy, and future research.	**15, 16**
**OTHER INFORMATION**			
Registration and	24a	Provide registration information for the review, including register name and registration number, or state that the review was not registered.	**6**

Section and Topic	Item #	Checklist item	Location where item is reported
protocol	24b	Indicate where the review protocol can be accessed, or state that a protocol was not prepared.	**6**
	24c	Describe and explain any amendments to information provided at registration or in the protocol.	**PROSPERO record**
Support	25	Describe sources of financial or non-financial support for the review, and the role of the funders or sponsors in the review.	**17**
Competing interests	26	Declare any competing interests of review authors.	**17**
Availability of data, code and other materials	27	Report which of the following are publicly available and where they can be found: template data collection forms; data extracted from included studies; data used for all analyses; analytic code; any other materials used in the review.	**17**

From: Page MJ, McKenzie JE, Bossuyt PM, Boutron I, Hoffmann TC, Mulrow CD, et al. The PRISMA 2020 statement: an updated guideline for reporting systematic reviews. BMJ 2021;372:n71. doi: 10.1136/bmj.n71

2 Search strategy

2.1 Search Strategy Restrictive vs. Liberal Transfusion

MEDLINE (OvidSP)

1. *Blood Transfusion/ad, mt, st, td or *Erythrocyte Transfusion/mt, st, td
2. ((transfus* or red cell* or red blood cell* or RBC* or PRBC*) adj5 (trigger* or thresh?old* or target* or restrict* or liberal* or aggressive* or conservative* or prophylactic* or limit* or protocol* or policy or policies or practic* or indicat* or strateg* or regimen* or criteri* or standard* or management or program*)).tw.
3. ((h?emoglobin or h?ematocrit orHB orHCT) adj5 (polic* or practic* or protocol* or trigger* or threshold* ormaintain* or indicator* or strateg* or criteri* or standard*)).tw.
4. (blood adj3 (management or program*)).mp.
5. ((transfus* or red cell* or red blood cell* or RBC* or PRBC*) and (critical* or intensive* or h?emorrhag* or bleed*)).ti.
6. or/1-5
7. randomized controlled trial.pt.
8. controlled clinical trial.pt.
9. randomi*.tw.
10. placebo.ab.
11. clinical trials as topic.sh.
12. randomly.ab.
13. groups.ab.
14. trial.tw.
15. 7 or 8 or 9 or 10 or 11 or 12 or 13 or 14
16. exp animals/ not humans/
17. 15 not 16
18. 6 and 17

2.2 Search Strategy Tranexamic Acid

1. exp Antifibrinolytic Agents/
2. (anti-fibrinolytic* or antifibrinolytic* or antifibrinolysin* or anti-fibrinolysin* or antiplasmin* or antiplasmin* or ((plasmin or fibrinolysis) adj3 inhibitor*)).ab,ti.
3. exp Aprotinin/
4. (Aprotinin* or kallikrein-trypsin inactivator* or bovine kunitz pancreatic trypsin inhibitor* or bovine pancreatic trypsin inhibitor* or basic pancreatic trypsin inhibitor* or BPTI or contrykal or kontrykal or kontrikal or contrical or dilmintal or iniprol or zymofren or traskolan or antilysin or pulmin or amicar or caprocid or epsamon or epsikapron or antilysin or iniprol or kontrikal or kontrykal or pulmin* or Trasylol or Antilysin Spofa or rp?9921 or antagosan or antilysin or antilysine or apronitin* or apronitrine or bayer a?128 or bovine pancreatic secretory trypsin inhibitor* or contrycal or frey inhibitor* or gordox or kallikrein trypsin inhibitor* or kazal type trypsin inhibitor* or (Kunitz adj3 inhibitor*) or midran or (pancrea* adj2 antitrypsin) or (pancrea* adj2 trypsin inhibitor*) or riker?52g or rp?9921or tracylol or trascolan or trasilol or traskolan or trazylol or zymofren or zymophren).ab,ti.
5. exp Tranexamic Acid/
6. (tranexamic or Cyclohexanecarboxylic Acid* or Methylamine* or amcha or trans-4 aminomethylcyclohexanecarboxylic acid* or t-amcha or amca or kabi 2161 or transamin* or exacyl or amchafibrin or anvitoff or spotof or cyklokapron or ugurol oramino methylcyclohexane carboxylate or aminomethylcyclohexanecarbonic acid or aminomethylcyclohexanecarboxylic acid or AMCHA or amchafibrin or amikapron or aminomethyl cyclohexane carboxylic acid or aminomethyl cyclohexanecarboxylic acid or aminomethylcyclohexane carbonic acid or aminomethylcyclohexane carboxylic acid or aminomethylcyclohexanecarbonic acid or aminomethylcyclohexanecarboxylic acid or aminomethylcyclohexanocarboxylic acid or aminomethylcyclohexanoic acid or amstat or anvitoff or cl?65336 or cl65336 or cyclocapron or cyclokapron or cyklocapron or exacyl or frenolyse or hexacapron or hexakapron or tranex or TXA).ab,ti.

7. exp Aminocaproic Acids/ or exp 6-Aminocaproic Acid/
8. (((aminocaproic or amino?caproic or aminohexanoic or amino?hexanoic or epsilon-aminocaproic or E-aminocaproic) adj2 acid*) or epsikapron or cy-116 or cy116 or epsamon or amicar or caprocid or lederle or Aminocaproic or aminohexanoic or amino caproic or amino n hexanoic or acikaprin or afibrin or capracid or capramol or caprogel or caprolest or caprolisine or caprolysin or capromol or cl 10304 or EACA or eaca roche or ecapron or ekaprol or epsamon or epsicapron or epsilcapramin or epsilon amino caproate or epsilon aminocaproate or epsilonaminocaproic or etha?aminocaproic or ethaaminocaproich or emocaprol or hepin or ipsilon or jd?177or neocaprol or nsc?26154 or tachostyptan).ab,ti.
9. 1 or 2 or 3 or 4 or 5 or 6 or 7 or 8
10. randomi?ed.ab,ti.
11. randomized controlled trial.pt.
12. controlled clinical trial.pt.
13. placebo.ab.
14. clinical trials as topic.sh.
15. randomly.ab.
16. trial.ti.
17. 10 or 11 or 12 or 13 or 14 or 15 or 16
18. (animals not (humans and animals)).sh.
19. 17 not 18
20. 9 and 19

2.3 Search Strategy Iron Therapy

(MedLine search strategy not published) Embase Search Strategy
1 exp iron therapy/
2 (iron or ferrous or ferric).af.
3 1 or 2
4 exp anemia/
5 (anemi* OR anaemi*).af.
6 4 or 5
7 exp crossover-procedure/ or exp double-blind procedure/ or exp randomized controlled trial/ or single-blind procedure/
8 (random* or factorial* or crossover* or placebo*).af.
9 7 or 8
10 3 and 6 and 9

2.4 Search Strategy Point of Care testing

1. exp Thrombelastography/ or Thromb?elastograph*.mp.or (ROTEM or TEG or ROTEG).
mp. or Thromboelastometry.mp.
2. ((randomized controlled trial or controlled clinical trial).pt. or randomized.ab. or placebo.
ab. or drug therapy.fs. or randomly.ab. or trial.ab. or groups.ab.) not (animals not (humans and
animals)).sh. (2177961)
3. 1 and 2

2.5 Search Strategy Cell Salvage

1. cell$ sav$.mp.

Roman M, *et al. BMJ Open* 2022; 12:e054582. doi: 10.1136/bmjopen-2021-054582

2. cell$ salvage.mp.
3. blood transfusion, autologous/
4. autotransfusion$.mp.
5. auto-transfusion$.mp.
6. blood salvage.mp.
7. autovac.mp.
8. solcotrans system.mp.
9. constavac.mp.
10. solcotrans.mp.
11. hemovac.mp.
12. BRAT.mp.
13. fresenius.mp.
14. consta vac.mp.
15. cell saver.mp.
16. dideco.mp.
17. electromedic.mp.
18. electromedics.mp.
19. gish biomedical.mp.
20. haemonetics.mp.
21. orth-evac.mp.
22. pleur-evac.mp.
23. sorenson.mp.
24. reinfusion system.mp.
25. sorin biomedical.mp.
26. or/1-25
27. exp blood transfusion/
28. exp hemorrhage/
29. exp anesthesia/
30. transfusion$.mp.
31. bleed$.mp.
32. blood loss$.mp.
33. hemorrhag$.mp.
34. haemorrhag$.mp.
35. or/27-34
36. 26 and 35
37. randomized controlled trial.pt.
38. controlled clinical trial.pt.
39. randomized controlled trials.sh.

40. random allocation.sh.
41. double blind method.sh.
42. single blind method.sh.
43. or/37-42
44. clinical trial.pt.
45. exp Clinical trials/
46. (clin$ adj25 trial$).ti,ab.
47. ((singl$ or doubl$ or trebl$ or tripl$) adj25 (blind$ or mask$)).ti,ab.
48. placebos.sh.
49. placebo$.ti,ab.
50. random$.ti,ab.
51. research design.sh.
52. or/44-51
53. comparative study.sh.
54. exp Evaluation studies/
55. follow up studies.sh.
56. prospective studies.sh.
57. (control$ or prospectiv$ or volunteer$).ti,ab.
58. or/53-57
59. 43 or 52 or 58
60. 36 and 59
61. animal/ not human/
62. 60 not 61

2.6 Search Strategy for Cost Effectiveness

Medline search terms

1 exp blood transfusion/
2 ((blood or red cell or rbc or platelet* or plasma or ffp or cryoprecipitate or prothrombin) adj3 (transfus* or retransfus* or therap*)).ti,ab.
3 (hemotransfus* or haemotransfus*).ti,ab.
4 ((blood adj2 (management or administ*5 or component*1)) or blood support).ti,ab.
5 or/1-4

Embase search terms

1 exp *blood transfusion/
2 ((blood or red cell or rbc or platelet* or plasma or ffp or cryoprecipitate or prothrombin) adj3 (transfus* or retransfus* or therap*)).ti,ab.
3 (hemotransfus* or haemotransfus*).ti,ab.
4 ((blood adj2 (management or administ*5 or component*1)) or blood support).ti,ab.
5 or/1-4

CRD search terms

#1 mesh descriptor blood transfusion explode all trees in NHSEED,HTA

Roman M, *et al. BMJ Open* 2022; 12:e054582. doi: 10.1136/bmjopen-2021-054582

#2 (((blood or red cell or RBC or platelet* or plasma or ffp or cryoprecipitate or prothrombin) adj3 (transfus* or retransfus* or therap*))) in NHSEED, HTA
#3 ((hemotransfus* or haemotransfus*)) in NHSEED, HTA
#4 (blood adj2 (management or administ* or component*)) OR (blood support) in NHSEED, HTA
#5 #1 or #2 or #3 or #4

Roman M, *et al. BMJ Open* 2022; 12:e054582. doi: 10.1136/bmjopen-2021-054582

3 PRISMA flow diagram (eFigure 1.)

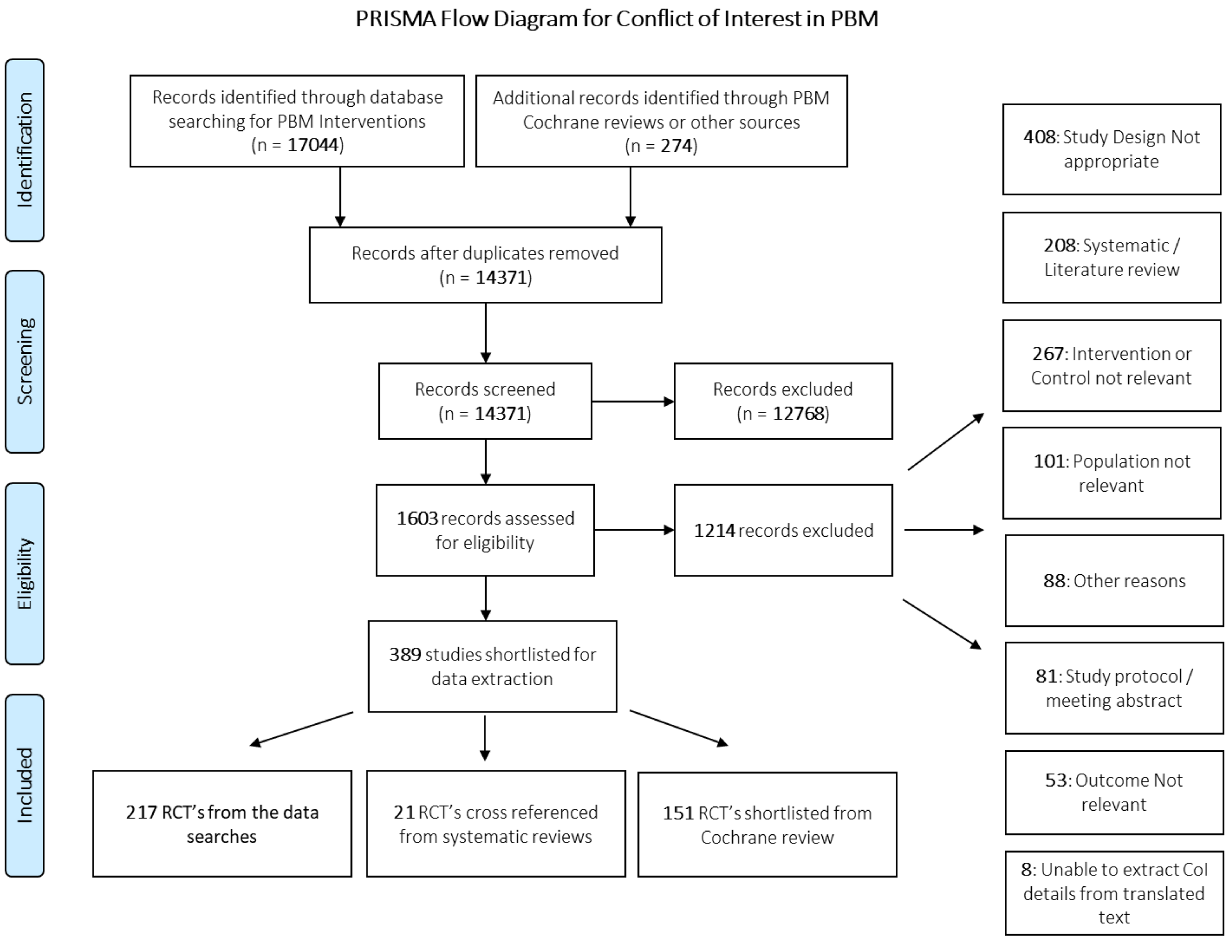

4 Characteristics of included studies (eTable 1)

388 studies were included in this analysis and grouped based on the presence of Author CoI, type of Author CoI, presence of funding disclosure and type of funding.

Thirty one trials (8%) had authors who declared CoI, while 183(47.1%) were unclear about CoI and 174(44.8%) declared none. The number of studies based on the type of author CoI were: Industry - 19(4.8%); Professional Advocacy organisation – 0; Blood Service – 6(1.5%); Non-profit – 10 (2.5%); and Not stated – 352 (90.7%).

Sixty five (16.7%) studies had any funding disclosed, while 193(49.7%) had no clear funding disclosure and 130(33.5%) disclosed no funding. The number of studies based on the type of funding were: Industry – 27(6.9%); Professional Advocacy organisation – 0; Blood Service – 8(2%); Non-profit – 70(18%); and Not stated – 283 (72.9%).

Study *(Author, Year)*	• Country<br>• Language<br>• Year of the trial completion<br>• Single- or Multi-Centre<br>• Study population size (n)<br>• Inclusion criteria (descriptive)	Exclusion criteria (descriptive)	• Type of Intervention (subtype if available)<br>• Type of Control<br>• Concomitant PBMs (list)	Primary Outcomes (list)	Secondary Actual Outcomes (list)	Author Conflict of interest (Any, Unclear, None)	Type:<br>- Industry<br>- Professional Advocacy organisation,<br>- Blood service<br>- Non-Profit<br>- Not stated	Funding Conflict of interest (Any, Unclear, None)	Type:<br>- Industry<br>- Professional Advocacy organisation,<br>- Blood service<br>- Non-Profit<br>- Not stated
Alshryda 2013[1]	• UK<br>• English<br>• 2013<br>• Single-Centre<br>• 157<br>• Patients undergoing unilateral primary total hip replacement	Not stated	• IV TXA<br>• Placebo<br>• -	Blood transfusion rate	Drain blood loss, haemoglobin concentration drop, generic quality of life (EuroQol), Oxford Hip Score, length of stay, a cost analysis, and complications.	Any	Industry	None	Not stated
Clave 2019[2]	• France<br>• English<br>• 2017<br>• Multi-Centre<br>•<br>• 1) Over 18 years of age; 2) awaiting primary elective THA; 3) scheduled for antithrombotic prophylaxis with rivaroxaban; 4) provided informed consent; and 5) registered	1) rapidly destructive osteoarthritis of the hip; 2) previous ipsilateral hip surgery; 3) major contraindications for treatment with TXA, such as epilepsy and renal failure (renal clearance < 30 ml/min); 4) patients already receiving antiplatelet agents (aspirin > 160 mg/j) or anticoagulants; 5) ischaemic arterial disease (myocardial infarction, stroke);	• Long IV TXA<br>• Short IV TXA<br>• Placebo	the difference in perioperative RBL between the baseline level and the level on day 3	The haemostatic effects of TXA on the levels of Hb and Ht and on the need for transfusion. Major bleeding was defined as clinically overt bleeding accompanied by one or more of the following: a decrease in the Hb level of > 2 g/dl over a 24-hour period, transfusion	Any	Industry	Any	Industry

	in the national social security system.	6) previous venous thromboembolism (VTE); 7) contraindication to treatment with rivaroxaban and 8) Child B-stage cirrhosis with coagulopathy.			of two or more units of PRBCs, bleeding at a critical site (intracranial, intra-spinal, intraocular, pericardial, intra-articular, intramuscular with compartment syndrome, or retroperitoneal), or fatal bleeding.				
Cvetanovich 2018[3]	• USA<br>• English<br>• 2016<br>• Single-Centre<br>• 110<br>• Patients undergoing primary anastomotic and reverse TSA	Allergy to TXA, acquired disturbances of colour vision, preoperative use of anticoagulant therapy within 5 days of surgery, history of arterial or venous thromboembolic disease (including deep venous thrombosis, pulmonary embolism, stroke, transient ischemic attack), ongoing pregnancy or breast-feeding, recent myocardial infarction (within 6 months before surgery), cardiac stent placement, renal impairment, haemophilia, refusal of blood products, revision TSA, TSA performed for the indications of acute proximal humeral fracture, or prior open shoulder surgery, including failed open reduction and internal fixation of proximal humeral fractures	• IV TXA<br>• Placebo<br>• -	Calculated postoperative blood loss.	Transfusion rates, weight of haemoglobin loss, hospital length of stay, and thromboembolic events.	Any	Industry	Any	Industry
Georgiadis 2013[4]	• USA<br>• English<br>• 2013<br>• Single-Centre<br>• 101	Religious objection to autologous blood transfusion, preoperative use of anticoagulant medication seven days prior to surgery, history of fibrinolytic disorder or blood dyscrasia,	• IV TXA<br>• Placebo<br>• -	-	-	Any	Industry	Unclear	Not stated

	• Patients who underwent primary total knee arthroplasty	cerebrovascular accident (CVA), myocardial infarction (MI), New York Heart Association Class III or IV heart failure (NYHA III-IV), atrial fibrillation, history of deep vein thrombosis (DVT) or pulmonary embolus (PE), preoperative International Normalized Ratio (INR) N 1.4, activated partial thromboplastin time (aPTT) N 1.4 × normal, platelets b 140,000/mm3, or renal failure defined as creatinine N 1.1 mg/dL or glomerular filtration rate b 60 mL/min/1.73 m2.							
Gillespie 2015[5]	• USA<br>• English<br>• 2014<br>• Single-Centre<br>• 111<br>• Patients who underwent total shoulder arthroplasty	Revision surgery, history of cardiac disease, liver disease, renal disease, preoperative haemoglobin level <11.5 g/dL or haematocrit <35%, severe joint deformity, history of joint infection, history of bleeding or metabolic disorder, history of peripheral vascular disease, history of prior deep venous thrombosis (DVT) or pulmonary embolism (PE), any patient unwilling to accept a blood transfusion, and any patient with a documented allergy to TXA	• IV TXA<br>• Placebo<br>• -	postoperative blood loss	Postoperative haemoglobin level.	Any	Industry	None	Non profit
Goobie 2018[6]	• USA<br>• English<br>• 2018<br>• Single-Centre<br>• 120<br>• Patients with adolescent idiopathic scoliosis who were between the ages of 10 and 18 years were	Haematological, coagulation, hepatic, or renal disorders and the administration of nonsteroidal anti-inflammatory drugs or acetylsalicylic acid within the previous 2 or 14 days, respectively, before surgery.	• IV TXA<br>• Placebo<br>• Cell Salvage	Blood loss	Blood transfusion	Any	Industry	None	Non profit

	included when they were scheduled for elective posterior instrumented spinal fusion at BCH.								
Johansson 2015[7]	• Denmark<br>• English<br>• 2013<br>• 60<br>• Non-anaemic patients undergoing cardiac surgery	Iron overload or disturbances in utilization of iron (e.g. haemochromatosis and haemosiderosis), s-ferritin >800 ng/ml, known hypersensitivity to any excipients in the investigational drug products, history of multiple allergies, decompensated liver cirrhosis and hepatitis, alanine aminotransferase >3 times normal upper value, acute infections, rheumatoid arthritis with symptoms or signs of active joint inflammation, pregnant or nursing women, participation in any other clinical trial where the trial drug had not passed five half-lives prior to screening, untreated vitamin B12 or folate deficiency, other IV or oral iron treatment within 4 weeks prior to screening visit, erythropoietin treatment within 4 weeks prior to screening visit, and impaired renal function defined by creatinine >150 mol/L. Patients who received blood transfusion <30 days before screening and/or during the elective or subacute CABG, valve replacement or a combination	• IV Fe<br>• Placebo	Change in Hb concentrations from baseline to 4 weeks postoperatively	- Proportion of patients who were anaemic (women Hb <12 g/dl and men Hb <13 g/dl) at day 5 and week 4,<br>- Proportion of patients who were able to maintain a Hb between 9·5 and 12·5 g/dl (both values included) at day 5 and week 4<br>- Number of patients in each treatment group who needed blood transfusion and number of transfusions administered<br>- Change from baseline in concentrations of s-ferritin, s-iron, transferrin saturation (TSAT) and reticulocytes at day 5 and week 4<br>- Safety (adverse events, vital signs, electrocardiogram (ECG), s-phosphate, and haematology and biochemistry parameters).	Any	Industry	Any	Industry
Laine 2017[8]	• Finland<br>• English<br>• 2017<br>• Single-Centre	Any hereditary or acquired haemostatic disorders, any malignancies, and severe chronic kidney disease	• Restrictive 80g/L<br>• Liberal<br>• Tranexamic acid<br>• POC testing	-	Amount of bleeding during the surgery and postoperatively from the chest tubes, RBC	Any	Industry	None	Non profit

	• 80<br>• Patients scheduled for elective open-heart surgery<br>• Restrictive threshold 8g/dl	(glomerular filtration rate o30 mL/min).			and blood product transfusions, diuresis, and cumulative fluid balance. Patient data during the surgery and intensive care were collected				
Langille 2013[9]	• Canada<br>• English<br>• 2011<br>• Single-Centre<br>• 28<br>• Patients undergoing functional endoscopic sinus surgery	Patients that had a history of hypertension, renal failure, or vascular disease, or if they were American Society of Anaesthesiologists (ASA) class III or greater	• IV TXA<br>• Placebo<br>• -	The Wormald grading scale.	The Peri-Operative Sinus Endoscopy (POSE) score, Lund-Kennedy endoscopic score, and total estimated blood loss.	Any	Industry	Unclear	Not stated
Mazer 2017[10]	• Canada<br>• English<br>• 2017<br>• Multi-Centre<br>• 4860<br>• Adults undergoing cardiac surgery who had EUROSCORE I of 6 or more<br>• Restrictive threshold 7.5g/dl	Patients unable to receive blood products, declined blood products, were involved in a preoperative autologous donation program, were undergoing heart transplantation, were having surgery solely for the insertion of a ventricular assist device, or were pregnant or lactating.	• Restrictive 75g/L<br>• Liberal<br>• Tranexamic acid	composite outcome of death from any cause, myocardial infarction, stroke, or new-onset renal failure with dialysis by hospital discharge or by day 28, whichever came first	Red-cell transfusion and other clinical outcomes.	Any	Industry	Any	Blood service
Murphy 2004[11]	• UK<br>• English<br>• 2004<br>• Single-Centre<br>• 196<br>• Patients aged 18 or over who were undergoing nonemergency first time coronary artery bypass grafting	Patients who are prevented from utilizing blood and blood products according to a system of beliefs (e.g., Jehovah's Witnesses), patients o warfarin, heparin, or other systemic anticoagulant drugs preoperatively, patients with congenital or acquired platelet, red cell, or clotting disorders, patients with ongoing or recurrent systemic sepsis and patients who were unable to give full informed consent for the study	• Cell salvage<br>• Control Group<br>• POC testing	-	intraoperative homologous blood transfusion, Hb concentration and haematocrit measurements, platelet count, prothrombin time, activated partial thromboplastin time, fibrinogen concentration, D-dimer concentration, and thromboelastography	Any	Industry	Any	Industry

Onodera 2012[12]	• Japan<br>• English<br>• 2012<br>• Single-Centre<br>• 100<br>• Patients scheduled to undergo TKA	Patients showing DVT preoperatively were excluded, as were those with known coagulation disorders, abnormal coagulation test values, or receiving anti-coagulation medication.	• IV TXA<br>• Placebo<br>• -	-	blood loss and the risk of asymptomatic DVT development	Any	Industry	None	Not stated
Palmieri 2017[13]	• USA<br>• English<br>• 2017<br>• Multi-Centre<br>• 345<br>• Admitted to a participating burn centre within 96 hours of injury with a burn injury ≥ 20% TBSA<br>• Restrictive threshold 7-8g/dl	<18 years of age; pregnant; unable or unwilling to receive blood products; chronically anaemic (haemoglobin <9.0 g/dl one month prior to enrolment); on renal dialysis prior to injury; brain dead, imminent brain death, or a non-survivable burn; experiencing angina or acute myocardial infarction on admission; pre-existing hematologic disease; or closed head injury with Glasgow coma scale <9.	• Restrictive 70-80g/L<br>• Liberal<br>• -	Number of BSIs as defined by the Burn Consensus Conference.	mortality, number of infectious episodes (urinary tract infections, pneumonia, wound infection), burn ICU LOS, hospital LOS, duration of mechanical ventilation, organ dysfunction (MODS), and time to 90% burn wound healing (defined as 7 days after the last excision and grafting procedure).	Any	Industry	None	Non profit
Perez-Jimeno 2018[14]	• Spain<br>• English<br>• 2018<br>• Single-Centre<br>• 293<br>• Only cemented or non-cemented primary elective THA were included.	Patients were excluded if presenting with hyper- or hypo-coagulability disorders, known allergy to TXA, intravenous iron, folic acid or recombinant human erythropoietin, epilepsy or hip fracture.	• IV TXA<br>• No TXA<br>• Iron therapy<br>• Restrictive threshold	RBCT rate (percentage of transfused patients) and index (RBCT units per patient)	pre-RBCT haemoglobin, post-operative thromboembolic complications	Any	Industry	None	Not stated
Spahn 2019[15]	• Switzerland<br>• English<br>• 2019<br>• Single-Centre<br>• 484<br>• Adult patients with anaemia scheduled for elective isolated coronary artery bypass grafting (CABG), valve surgery, and	- Patients in need of urgent surgery the day of hospital admission<br>- Participation in another clinical trial during the last 4 weeks prior to patient screening<br>- Impairments, diseases or language problems which do not allow the patient to fully	• IV Fe<br>• Placebo<br>• Restrictive threshold	number of RBC transfusions administered during the first 7 days (starting with the day of operation), until death or hospital discharge, whichever came first	**7 day (short)**: acute kidney injury (increase of creatinine >50% vs preoperative value), infections requiring antibiotic treatment and perioperative course of Hb, reticulocyte count, reticulocyte Hb content,	Any	Industry	Any	Industry

	combined CABG and valve procedures were eligible	understand the consequences of study participation<br>- Age < 18 years<br>- Pregnant and/or breastfeeding women<br>- Jehovah's Witnesses<br>- Patients suffering from endocarditis<br>- Known allergy against iron-carboxymaltose or mannitol<br>- Need for intraoperative extra-corporeal membrane oxygenation<br>- Untractable surgical bleeding with massive transfusion (≥ 10 red blood cell (RBC) transfusions per 24h			platelet and leucocyte counts, international normalised ratio, high-sensitivity troponin, creatinine, C-reactive protein, calculated RBC loss (preoperative RBC mass minus RBC mass at postoperative day 5 plus transfused RBC mass10) as well as tolerance of study drugs and placebo administration.<br>**90 days secondary** outcomes: percentage of patients without any RBC transfusion, number of allogeneic blood products (RBC, plasma, platelets) administered, length of stay in intensive care and in hospital, duration of mechanical ventilation, major adverse cardiac and cerebrovascular events, new onset of atrial fibrillation, thrombotic and thromboembolic complications, mortality, product acquisition costs, and the occurrence of serious adverse events				
Springer 2016[16]	• USA<br>• English<br>• 2016<br>• Single-Centre<br>• 186	1. Patients with a preoperative Hgb b 10 mg/dL 2. Patients who are unwilling to consent to blood transfusions 3. Patients with a history of bleeding	• IV TXA<br>• Reinfusion drains<br>• No TXA<br>• Iron therapy	Allogeneic blood transfusion, measured as a dichotomous variable; the	-	Any	Industry	Any	Non profit

	• 1. Patients presenting for primary unilateral hip or knee arthroplasty 2. N18 y of age 3. Preoperative haemoglobin on day of surgery ≥ 10 mg/dL	disorder 4. Patients on anticoagulation therapy preoperatively (ASA 325 mg, Plavix or Coumadin) 5. Patients with a history of thromboembolic events (DVT, PE, CVA MI) 6.Patients with platelet counts b 100,000 7. Patients with kidney disease (serum Cr N 1.2) 8. Patients with end-stage renal disease or on haemodialysis 9. Patients with renal transplant 10. Patients presenting for bilateral total hip or knee arthroplasty 11. Patients presenting for conversion or revision total hip or knee procedures 12. Patients donating pre-autologous blood 13. Patients with primary hematologic disease or malignancy 14. Patients with allergy to TA 15. Patients with hepatic disease 16. Patients not discontinuing steroids use before surgery 17. Patients with religious beliefs/practices prohibiting blood transfusions 18. Patients with cognitive impairment 19. Patients who are terminally ill.		change in haemoglobin level (delta haemoglobin); autologous blood reinfusion; and hospital costs.					
Vara 2017[17]	• USA<br>• English<br>• 2017<br>• Single-Centre<br>• 102<br>• Patients undergoing primary reverse total shoulder arthroplasty	Minors, acute proximal humeral fracture, concomitant procedures (e.g., latissimus dorsi tendon transfer), known allergy to TXA, preoperative anaemia (Hb <11 g/dL in women, Hb <12 g/dL in men), refusal of blood products, coagulopathy (thrombophilia, platelet count <150,000 mm3, international normalized ratio	• IV TXA<br>• Placebo<br>• -	-	Calculated total blood loss, drain output, and haemoglobin (Hb) drop were measured. Postoperative transfusions were recorded. Complications were assessed out to 6 weeks postoperatively.	Any	Industry	Unclear	Not stated

		>1.4, partial thromboplastin time >1.4 times normal), history of thromboembolic event, major comorbidities (severe pulmonary disease, coronary artery disease, previous myocardial infarction, renal failure), or refusal to give written consent.							
Verma 2014[18]	• USA<br>• English<br>• 2014<br>• Single-Centre<br>• 125<br>• Patients with adolescent idiopathic scoliosis	-	• IV TXA<br>• EACA<br>• Placebo<br>• Cell salvage	Intraoperative blood loss and postoperative drainage.	Transfusion requirements and haematocrit changes both intraoperatively and postoperatively.	Any	Industry	None	Not stated
Watts 2017[19]	• USA<br>• English<br>• 2017<br>• Single-Centre<br>• 138<br>• Patients who presented with a low-energy, isolated, FNF (AO 31B) treated with either hemi- or total hip arthroplasty within 72 hours of injury	Blood transfusion before surgery; creatinine clearance (CrCl) <30 mL/min; previous unprovoked and/or recurrent deep venous thrombosis (DVT) or pulmonary embolism (PE); recent myocardial infarction (MI), cerebrovascular event, or provoked DVT or PE within 30 days; coronary stent placement within 6 months; history of heritable hypercoagulable condition; disseminated intravascular coagulation; subarachnoid haemorrhage; pregnancy; and active breastfeeding.	• IV TXA<br>• Placebo<br>• Restrictive threshold	Proportion of patients who underwent blood transfusion during hospitalization.	Calculated blood loss, number of units transfused during hospitalization, and incidence of adverse events at 30 and 90 days including thromboembolic event, wound complications, reoperation, hospital readmission, and all-cause mortality.	Any	Industry	Any	Industry
Aguilera 2013[20]	• Spain<br>• English<br>• 2013<br>• Single-Centre<br>• 83<br>• Adult patients undergoing elective primary total knee	Patients with an allergy to tranexamic acid or to Aprotinin, a history of coagulopathy or a thromboembolic event, previous vascular or cardiac bypass surgery, treatment with an anticoagulant or	• IV TXA<br>• No TXA<br>• -	total blood loss collected in drains after surgery	Calculated hidden blood loss, transfusion rate, preoperative and postoperative haemoglobin, number of blood units transfused, adverse events, and mortality.	Any	Blood service	Any	Blood service

	arthroplasty from June 2010 to October 2011	contraceptives, presence of a cardiovascular prosthesis, and patients who declined to participate.							
Blauhut 1994[21]	• Switzerland<br>• English<br>• 1994<br>• Single-Centre<br>• 30<br>• Patients undergoing cardiopulmonary bypass for coronary disease	Intake of aspirin, other nonsteroidal anti-rheumatics, or beta-lactam antibiotics; treatment with heparin, fibrinolytic agents, or oral anticoagulants; a condition requiring emergency surgery or reoperation; and liver or kidney disease.	• IV TXA<br>• No TXA<br>• -	-	-	Any	Blood service	Unclear	Not stated
Grover 2006[22]	• UK<br>• English<br>• 2006<br>• Multi-Centre<br>• 260<br>• Patients undergoing elective hip and knee replacement surgery<br>• Restrictive threshold 8g/dl	Exclusion criteria were age < 55 years, digoxin therapy, ECG evidence of conduction defects, ST segment depression, left ventricular hypertrophy or left bundle branch block. Any patient with anaemia was also excluded.	• Restrictive 80g/L<br>• Liberal<br>• -	-	Ischaemic load, blood load, Hb concentration, number of units transfused, length of hospital stay, adverse events, new infections requiring antibiotic therapy	Any	Blood service	Any	Blood service
Kuitunen 2005[23]	• Finland<br>• English<br>• 2005<br>• Single-Centre<br>• 40<br>• Patients who underwent cardiac surgery	Patients with pre-operative coagulation disorders; those taking medication with anticoagulants, acetosalicylic acid, platelet inhibitors or non-steroid anti-inflammatory drugs within the previous 5 days; those with renal insufficiency.	• IV TXA<br>• Placebo<br>• -	-	Perioperative blood loss	Any	Blood service	Unclear	Not stated
So-Osman 2013[24]	• Netherlands<br>• UK<br>• 2013<br>• 603<br>• -<br>• Restrictive threshold: most restrictive transfusion policy	-	• Restrictive (trigger age dependent)<br>• Liberal<br>• -	RBC use	Postoperative complications and quality of life	Any	Blood service	None	Non profit

Carson 2011[25]	• USA<br>• English<br>• 2011<br>• Multi-Centre<br>• 2016<br>• Patients 50 years of age or older who were undergoing primary surgical repair of a hip fracture and who had clinical evidence of or risk factors for cardiovascular disease were eligible if they had a haemoglobin level of less than 10 g per decilitre within 3 days after surgery. According to the original protocol, only patients with cardiovascular disease (a history of ischemic heart disease, electrocardiographic evidence of previous myocardial infarction, a history or presence of congestive heart failure or peripheral vascular disease, or a history of stroke or transient ischemic attack) were eligible.<br>• Restrictive threshold 8g/dl	Patients were excluded if they were unable to walk without human assistance before hip fracture, declined blood transfusions, had multiple trauma (defined as having had or planning to undergo surgery for non–hip-related traumatic injury), had a pathologic hip fracture associated with cancer, had a history of clinically recognized acute myocardial infarction within 30 days before randomization, had previously participated in the trial with a contralateral hip fracture, had symptoms associated with anaemia (e.g., ischemic chest pain), or were actively bleeding at the time of potential randomization.	• Restrictive 80g/L<br>• Liberal<br>• -	inability to walk 10 feet (or across a room) without human assistance or death prior to closure of the window for 60-day mortality	Hb concentration, acute coronary syndrome (ACS), in-hospital myocardial infarction, unstable angina or death, disposition on discharge, survival, functional measures, fatigue/energy, readmission to hospital, pneumonia, wound infection, thromboembolism, stroke or transient ischaemic attack, cognition (Gruber-Baldini), mortality at 30 days, and long-term mortality	Any	Non-profit	Unclear	Not stated
Huang 2017[26]	• China<br>• English<br>• 2017<br>• Single-Centre<br>• 150<br>• Patients who underwent primary total knee arthroplasty	Patients scheduled for revision procedures, bilateral procedures, previous knee surgery, flexion deformity of >30 deg, varus-valgus deformity of >30 deg anaemia (haemoglobin [Hb] level of <12 g/dL for women and <13 g/dL for men), contraindications for the use of TXA (any history of blood clot events within 6	• IV TXA + Tourniquet<br>• IV TXA<br>• No TXA<br>• -	-	total blood loss, hidden blood loss, maximum decline in Hb, transfusion rate, and CRP and IL-6 concentrations. The groups were also compared for swelling ratio, length of hospital stay, patient satisfaction, perioperative visual	Any	Non-profit	Any	Non profit

		months), ASA grade IV, and coagulation disorders			analog scale (VAS) pain score, cases of wound secretion, DVT and PE events, and other complications.				
Lin 2011[27]	• Taiwan<br>• English<br>• 2009<br>• Single-Centre<br>• 100<br>• Patients who underwent minimally invasive total knee arthroplasty	Patients with thrombocytopenia or haemophilia, prior surgery of the affected knee, haemoglobin (Hb) less than 10 g/dL on the day of admission, a history of thromboembolic disease or lifelong warfarin therapy for thromboembolism prophylaxis, declined to participate in the study, who did not withhold use of aspirin for 1 week before admission.	• IV TXA<br>• Placebo<br>• -		Data were collected on demographics, pre-operative investigations, blood loss, and blood products transfused during surgery.	Any	Non-profit	None	Non profit
Myles 2017[28]	• Australia<br>• English<br>• 2017<br>• Multi-Centre<br>• 4631<br>• Patients undergoing CABG surgery	1. Poor (English) language comprehension<br>2. Clinician preference for antifibrinolytic therapy<br>3. Urgent surgery for unstable coronary syndromes where for clinical reasons antiplatelet medication cannot be discontinued<br>4. Active peptic ulceration<br>5. Allergy or contraindication to aspirin or tranexamic acid<br>6. Aspirin therapy within 4 days of surgery<br>7. Warfarin or Clopidogrel therapy within 7 days of surgery, or GIIb/IIIa antagonists within 24 h of surgery<br>8. Thrombocytopenia or any other known history of bleeding disorder<br>9. Severe renal impairment (serum creatinine >250 µmol/l,	• IV TXA<br>• No TXA<br>• -	composite of death and thrombotic complications (nonfatal myocardial infarction, stroke, pulmonary embolism, renal failure, or bowel infarction) within 30 days after surgery.	Death, nonfatal myocardial infarction, stroke, pulmonary embolism, renal failure, bowel infarction, reoperation due to major haemorrhage or cardiac tamponade, and a requirement for transfusion.	Any	Non-profit	None	Non profit

		or estimated creatinine clearance <25 ml/min)<br>10. Recent haematuria<br>11. Thromboembolic disease relating to: history of postoperative or spontaneous pulmonary embolism, spontaneous arterial thrombosis or familial hypercoagulability (e.g. lupus anticoagulant, protein C deficiency)<br>12. Pregnancy							
Yi 2016[29]	• China<br>• English<br>• 2014<br>• Single-Centre<br>• 150<br>• Patients undergoing total hip arthroplasty	Patients with an allergy to TXA; had been treated with warfarin, heparin, or oestrogen before surgery; had a history of hyper-coagulation, haemophilia, deep vein thrombosis, or pulmonary embolism; were morbidly obese; or had hepatic or renal dysfunction.	• IV TXA+Top TXA<br>• IV TXA + Placebo<br>• Placebo<br>• -	Blood-loss variables (total, intraoperative, and drainage blood loss; changes in haemoglobin, haematocrit, and platelet concentration; and amount of IV transfusion fluid) and transfusion values (frequency of transfusion and number of transfused blood units).	The length of the hospital stay, range of hip motion, Harris hip score, and prevalence of deep vein thrombosis and pulmonary embolism.	Any	Non-profit	Any	Non profit
Zonis 1996[30]	• Canada<br>• English<br>• 1996<br>• Single-Centre<br>• 82<br>• Children undergoing cardiac operations in which cardiopulmonary bypass	Patients with a history of haematuria, renal failure, previous thrombotic episodes, or past bleeding complications.	• IV TXA<br>• No TXA<br>• -	-	Post-operative blood loss and fluid replacement were recorded for the next 24 hours. In addition, haemoglobin, platelet counts, and coagulation measures were recorded every 6 hours.	Any	Non-profit	Any	Non profit

Laoruengthana 2019b[31]	• Thailand/USA<br>• English<br>• 2019<br>• Single-Centre<br>• 226<br>• patients diagnosed with primary osteoarthritis of the knee and scheduled for primary unilateral TKA	Patients with previous history of thromboembolic event, cardiovascular disease or cerebrovascular accident were excluded. Patients with preoperative haemoglobin of less than 10 g/dl, bleeding disorder, and patients requiring anticoagulant therapy were also excluded.	• No TXA<br>• IA TXA<br>• IV TXA<br>• -	blood loss reduction	Effect on postoperative 56 pain, morphine consumption and knee flexion after TKA when using the TXA.	Any	Not stated	Any	Industry
Aghdaii 2012[32]	• Iran<br>• English<br>• 2012<br>• Single-Centre<br>• 50<br>• The inclusion criteria were as follows: primary, elective, on -pump CABG surgery; age between 30 and 70 years; left ventricular ejection fraction ≥45%, pump time	The exclusion criteria were: patients with known coagulation disorders; redo or emergency surgery; patients on Warfarin, heparin, or other systemic anticoagulant drugs and antiplatelet drugs such as Aspirin (the patients either did not take Aspirin or took a maximum dose of 80 mg/day) preoperatively; and co -existing diseases (renal and hepatic disease diabetes mellitus, hypertension, and endocrine and haematology disorders) .B	• Cell Salvage<br>• Non Cell Salvage Transfusion<br>• -	-	Volumes of the intraoperative autologous and homologous transfusion, activated clotting time (ACT) of the transfused bloods, and ACT and amount of blood loss in the patients were measured intra and postoperatively.	Unclear	Not stated	None	Not stated
Ahn 2012[33]	• Korea<br>• English<br>• 2012<br>• Single-Centre<br>• 76<br>• Anaemic patients who continued dual antiplatelet therapy until within 5 days of off-pump	Patients with impaired renal function (serum creatinine [sCr] >20 mg/L), hepatic dysfunction, neurologic dysfunction or hematologic disorders	• IV TXA<br>• Placebo<br>• Cell Salvage	perioperative (combined period of intraoperative and postoperative 24h) transfusion requirement between the groups	Amount of perioperative blood loss between the groups.	Unclear	Not stated	None	Not stated
Albirmawy 2013[34]	• Egypt<br>• English<br>• 2013<br>• Single-Centre<br>• 400	Children who had revision adenoidectomy, combined procedure (adenotonsillectomy), haemoglobin level <9.0 g/dL,	• Top TXA<br>• Placebo<br>• -	frequency of post-operative bleeding that occurred during the initial admission or	Perioperative blood loss	Unclear	Not stated	Unclear	Not stated

	• Children underwent primary isolated adenoidectomy	bleeding diathesis (e.g. haemophilia or thrombocytopenia), renal or hepatic impairment, known allergy to TA, recent (<7 days before surgery) intake of antiplatelets (e.g. Aspirin, non-steroidal anti-inflammatory drugs) or Heparin administration within 48 h of operation.		during the follow-up period					
Ali Shah 2015[35]	• Pakistan<br>• English<br>• 2015<br>• Single Centre<br>• 100<br>• Adult patients undergoing elective on pump cardiac surgeries	Patients for surgeries for congenital heart diseases and thoracic aorta redo or emergency procedures, patients who were on anti-platelet drugs (Aspirin/ Clopidogrel) within 7 days of surgery, patients with impaired renal functions (creatinine clearance of < 30 ml/minutes), chronic liver disease and bleeding diathesis.	• Top TXA<br>• Placebo<br>• -	-	Perioperative blood loss	Unclear	Not stated	Unclear	Not stated
Alipour 2013[36]	• Iran<br>• English<br>• 2013<br>• Single-Centre<br>• 53<br>• Patients undergoing knee arthroplasty	Patients with any history of severe ischaemic heart diseases, renal failure, cirrhosis, history of bleeding disorders or thromboembolic events	• PO TXA<br>• No TXA<br>• -	The bleeding rate in surgery drains at 12 and 24 h after surgery.	Risk & number of RBC transfusion<br>Perioperative blood loss	Unclear	Not stated	Unclear	Not stated
Altun 2017[37]	• Turkey<br>• English<br>• 2017<br>• Single-Centre<br>• 28<br>• Emergency coronary bypass surgery patients under the influence of dual antiplatelet therapy	Patients with chronic renal insufficiency, hepatic dysfunction, haematological disorders, drug addiction that might affect the haematological system, requirements for non-coronary cardiac surgery, or use of intra-aortic balloon pumps	• IV TXA<br>• No TXA<br>• -	-	Hb values<br>Total drains drainage<br>Thrombotic complications<br>Length of ICU and Hospital stay	Unclear	Not stated	Unclear	Not stated

Alvarez 2008[38]	• Spain<br>• English<br>• 2008<br>• Single-Centre<br>• 95<br>• All patients ASA-I to -III patients diagnosed with osteoarthrosis and undergoing unilateral bicondylar cemental total knee arthroplasty.	Patients with known allergy to tranexamic acid, ASA-IV physical status or higher, severe ischemia and/or heart valve disease, history of thromboembolic episodes, known coagulopathy, and renal dysfunction (serum creatinine concentration, >1.5 mg/dL).	• IV TXA<br>• Placebo<br>• Iron therapy	Transfusion rate	Postoperative blood loss	Unclear	Not stated	Unclear	Not stated
Andreasen JJ 2004[39]	• Denmark<br>• English<br>• 2004<br>• Single-Centre<br>• 44<br>• Primary, elective, on-pump coronary artery bypass grafting (CABG) patients with low baseline risk of postoperative bleeding	Treatment with acetylsalicylic acid, non-steroidal anti-inflammatory drugs or other platelet inhibitors within 7 days before surgery	• IV TXA<br>• Placebo<br>• Cell salvage	Postoperative blood loss and the proportion of patients requiring allogeneic transfusion	Development of perioperative myocardial infarction (peak CK-MB . 50 U/l and/or development of new Q waves), acute renal insufficiency (creatinine value twice the baseline or need for dialysis), transient ischemic attacks or stroke, early mortality (<30 days+ hospital mortality) and mediastinal infection within 30 days.	Unclear	Not stated	Unclear	Not stated
Antinolfi 2014[40]	• Italy<br>• English<br>• 2014<br>• Single-Centre<br>• 40<br>• Patients receiving primary unilateral total knee arthroplasty due to primary knee osteoarthritis	Tranexamic acid allergy, the use of pharmacological anticoagulant therapy, previous knee surgery and renal failure	• IA TXA<br>• No TXA<br>• -	-	-	Unclear	Not stated	Unclear	Not stated
Armellin 2001[41]	• Italy<br>• English<br>• 2001<br>• Single-Centre<br>• 300	Patients with a known coagulopathy, thrombocytopenia (platelet count, 100,000/mm3),	• IV TXA<br>• Placebo<br>• -	-	-	Unclear	Not stated	Unclear	Not stated

Roman M, *et al. BMJ Open* 2022; 12:e054582. doi: 10.1136/bmjopen-2021-054582

	• Adult cardiac surgery patients	anaemia (haemoglobin level, <10 g/dL), hepatic or renal dysfunction<br>(Creatinine level, >1.5 mg/dL), or endocarditis, autologous blood donors, patients undergoing redo procedures, and patients who refuse blood transfusion for religious reasons.							
Auvinen 1987[42]	• Finland<br>• English<br>• 1987<br>• Single-Centre<br>• 76<br>• Patients who came for scheduled thyroid surgery	Not stated	• IV TXA<br>• Placebo<br>• -	-	-	Unclear	Not stated	Unclear	Not stated
Avidan 2004[43]	• United Kingdom<br>• English<br>• 2004<br>• Single-Centre<br>• 102<br>• Routine elective first-time CABG surgery with cardiopulmonary bypass, managed according to standard clinical practice at local institution treated by the same surgical, intensivist and anaesthetic team	Patients with preoperative abnormal clotting tests, including INR> 1.5, aPTT ratio > 1.5, platelet count < 150 X 109 litre−1, any medication affecting<br>coagulation within 72 hours of surgery, including warfarin, heparin, low molecular weight heparin, aspirin and Clopidogrel	• TEG+Hepcon+PFA<br>• Standard of care<br>• Tranexamic acid<br>• Restrictive Threshold	Blood loss and transfusion, postoperative 24-hour blood loss-	INR, aPTT, TEG variables, haemoglobin and platelet values, coagulation<br>values	Unclear	Not stated	Any	Blood service
Basavaraj 2017[44]	• India<br>• English<br>• 2017<br>• Single-Centre<br>• 60<br>• Patients undergoing thoracic spine fixation	Patients with pre-existing renal or hepatic disorder, bleeding diathesis, history of malignancy or coronary artery disease, thromboembolic event 1 year prior to surgery, haemoglobin< 8gm/dL, and history of uncontrolled hypertension	• IV TXA<br>• Placebo<br>• -	-	Perioperative blood loss, amount of blood transfusion, postoperative haemoglobin and haematocrit levels.	Unclear	Not stated	Unclear	Not stated

Beikaei 2015[45]	• Iran<br>• English<br>• 2015<br>• Single-Centre<br>• 100<br>• Normotensive patients scheduled for elective open rhinoplasty aged 16-42 years with ASA class of either I or II without a history bleeding diathesis	Presence of a history of allergy or hypersensitivity to Tranexamic acid, brain vascular diseases, coronary artery diseases, cardiac dysrhythmia, liver/kidney or metabolic disorders, ASA class of either III or IV.	• IV TXA<br>• Placebo<br>• -	estimated volume of intraoperative bleed	No secondary outcome measures were defined.	Unclear	Not stated	Unclear	Not stated
Benoni G 2001[46]	• Sweden<br>• English<br>• 2001<br>• Single-Centre<br>• 39<br>• Patients with primary total hip arthroplasties	Patients who were to undergo bone grafting or had bleeding disorders or signs of renal insufficiency	• IV TXA<br>• Placebo<br>• -	-	-	Unclear	Not stated	Any	Industry
Blatsoukas 2010[47]	• Greece<br>• English<br>• 2010<br>• Single-Centre<br>• 248<br>• Patients undergoing unilateral TKR for knee osteoarthritis	Exclusion criteria were patients on anticoagulation therapy, with rheumatoid or seronegative arthritis, blood dyscrasia, malignancy or immunocompromised disease	• Intra+Post Cell Salvage<br>• Non Cell Salvage Transfusion<br>• Post-operative Auto-transfusion<br>• -	-	Patients demographic and clinical data including age, gender, body mass index (BMI), preoperative Hb value, operation time, side of operation, the need of ABT, reinfusion blood volume (IAT and PAT), blood loss, side effects, complications, and postoperative Hb levels on post-operative days 1, 2, 3, and 7 were documented.	Unclear	Not stated	Unclear	Not stated
Boylan JF 1996[48]	• Canada<br>• English<br>• 1996<br>• Single-Centre<br>• 45<br>• Patients undergoing primary isolated orthotopic liver transplantation	Patients with primary biliary cirrhosis, Primary sclerosing cholangitis, predisposition to a thrombotic tendency, fulminant hepatic failure.	• IV TXA<br>• Placebo<br>• -	-	-	Unclear	Not stated	Unclear	Not stated

Bracey 1999[49]	• USA<br>• English<br>• 1999<br>• Single-Centre<br>• 428<br>• Patients who underwent first time, elective CABG surgery<br>• Restrictive threshold 8g/dl	Patient exclusion criteria included a preoperative Hb level 2500 mL within 24 hours of operation, and the patient's refusal of blood transfusion for religious reasons.	• Restrictive 80g/L<br>• Liberal<br>• -	-	Mortality, length of hospital stay, blood usage (units), blood loss, complications, infection<br>rates, cardiac events	Unclear	Not stated	Unclear	Not stated
Bradshaw 2012[50]	• Australia<br>• English<br>• 2012<br>• Single-Centre<br>• 46<br>• Orthopaedic Patients for primary total knee replacement as a treatment for osteoarthritis	Patients with a history of thromboembolic events, anticoagulation that<br>could not be ceased within the recommended timeframe before surgery, peripheral vascular disease, oral contraception, pregnancy, current bleeding at any site, immunocompromise<br>from a known medical condition or medical therapy, known hypersensitivity to the study medication, creatinine clearance of less than 30 mLs/min, or significant hepatic disease	• PO TXA<br>• Placebo<br>• Restrictive threshold	-	Haemoglobin and haematocrit taken 24 hours postoperatively and total blood loss in wound drains at 24 hours.	Unclear	Not stated	Any	Industry
Brown RS 1997a[51]	• USA<br>• English<br>• 1997<br>• Single-Centre<br>• 60<br>• Adult patients undergoing primary coronary artery bypass grafting surgery	Patients with a platelet count less than 100,000/mm^3 or a coagulopathy, or those receiving thrombolytic therapy or warfarin	• IV TXA<br>• Placebo<br>• Restrictive threshold<br>• Cell salvage	-	Mediastinal chest tube blood loss measured hourly for the first 24 h in the ICU.<br>New stroke or deaths for any reason within 30 days<br>Mediastinal or systemic infections within 30 days	Unclear	Not stated	Unclear	Not stated
Brown RS 1997b[51]	• USA<br>• English<br>• 1997<br>• Single-Centre	Patients with a platelet count less than 100,000/mm^3 or a coagulopathy, or those	• IV TXA<br>• Placebo<br>• Restrictive threshold	-	Mediastinal chest tube blood loss measured hourly for the first 24 h in the ICU.	Unclear	Not stated	Unclear	Not stated

	• 60<br>• Adult patients undergoing primary coronary artery bypass grafting surgery	receiving thrombolytic therapy or warfarin	• Cell salvage		New stroke or deaths for any reason within 30 days<br>Mediastinal or systemic infections within 30 days				
Bulutcu 2005[52]	• Turkey<br>• English<br>• 2005<br>• Single-Centre<br>• 50<br>• Children undergoing cardiac surgery	Patients undergoing reoperations with sternotomy within 6 months after using Aprotinin or tranexamic acid, patients that required emergency operations, patients taking aspirin, dipyridamole or other anticoagulants, and known coagulation disorders, known metabolic disorders, renal or hepatic insufficiency, or previous exposure to Aprotinin or tranexamic acid	• IV TXA<br>• No TXA<br>• Cell salvage	-	-	Unclear	Not stated	Unclear	Not stated
Bush 1997[53]	• USA<br>• English<br>• 1997<br>• Single-Centre<br>• 99<br>• Patients undergoing elective aortic or infra inguinal arterial reconstructions<br>• Restrictive threshold 9g/dl	Patients were excluded from participation if they refused blood transfusions for religious or other reasons, did not speak English, or had had a myocardial infarction within 3 months preceding the scheduled operation.	• Restrictive 90g/L<br>• Liberal<br>• -	myocardial ischaemia, myocardial infarction, and death	Length of intensive care unit stay, hospital stay, and graft patency	Unclear	Not stated	Unclear	Not stated
Cao 2015[54]	• China<br>• Chinese<br>• 2015<br>• Single-Centre<br>• 100<br>• Patients who underwent total knee arthroplasty	-	• IV TXA<br>• No TXA<br>• Restrictive threshold	-	-	Unclear	Not stated	Unclear	Not stated
Carabini 2017[55]	• USA<br>• English<br>• 2017<br>• Single-Centre	Patients with a history of severe coronary artery disease defined as more than 50% occlusive disease or a history of	• IV TXA<br>• Placebo<br>• Cell salvage	the total volume of red blood cells	estimated blood loss, platelet and cryoprecipitate transfusion, and 24-	Unclear	Not stated	None	Non profit

	• 61<br>• Patients undergoing multi-level complex spinal fusion with and without osteotomies (more than 18 years old, had no reported history of arterial or venous thromboembolic disease, and had a more than 80% chance of requiring major transfusion)	revascularization, cerebral vascular disease with previous cardiovascular accident or transient ischemic attack, venous thromboembolism, or renal insufficiency with a glomerular filtration rate of less than 40 mL/min/m^2. Patients were also excluded if they were unable or unwilling to provide informed consent or were undergoing surgery for tumour, trauma, or infection.		transfused intraoperatively.	hour postoperative allogenic PRBC transfusion.				
Carson 1998[56]	• USA<br>• English<br>• 1998<br>• Single-Centre<br>• 84<br>• Patients were eligible for the trial if their Hb levels were less than 10 g per dL in the immediate postoperative period, defined as the time from the end of anaesthesia in the operating room to 11:59 PM 3 days after surgery (counted from 12:OO midnight on the first day after surgery)<br>• Restrictive threshold 8g/dl	Patients who refused transfusion because of religious beliefs, suffered multiple trauma (defined as any in- jury that required surgical repair in addition to the hip fracture), or had symptoms of anaemia were excluded from the trial.	• Restrictive 80g/L<br>• Liberal<br>• -	-	Mortality, length of hospital stay, blood usage (units), complications, pneumonia, stroke, thromboembolism	Unclear	Not stated	Unclear	Not stated
Casati 2001[57]	• Itay<br>• English<br>• 2001<br>• Single-Centre<br>• 510<br>• Patients undergoing elective cardiac surgery with use of cardiopulmonary bypass	Patients with chronic renal insufficiency (plasmatic creatinine concentration more than 2 mg/kg), history of hematologic disorders, hepatic dysfunction (active hepatitis, cirrhosis), history of pulmonary embolism, deep venous thrombosis, and cerebrovascular injury.	• IV TXA (2mg/kg/h)<br>• IV TXA (1mg/kg/h)<br>• Placebo<br>• -	Bleeding	Hematologic data, allogeneic transfusions, thrombotic complications, intubation time, and intensive care unit and hospital stay duration also were evaluated.	Unclear	Not stated	Unclear	Not stated

Casati 2002[58]	• Italy<br>• English<br>• 2002<br>• Single-Centre<br>• 60<br>• Patients undergoing elective surgery involving thoracic aorta	Patients with advanced chronic renal insufficiency (creatinine >2 mg/dL), active chronic hepatitis or cirrhosis, and history of hematologic disorders.	• IV TXA<br>• Placebo<br>• Restrictive threshold	Perioperative bleeding	Perioperative allogeneic transfusions, major thrombotic complications (myocardial infarction, pulmonary embolism, renal insufficiency), and surgical outcomes	Unclear	Not stated	Unclear	Not stated
Casati 2004a [59]	• Italy<br>• English<br>• 2004<br>• Single-Centre<br>• 51<br>• Patients scheduled for on-pump coronary artery bypass grafting	Patients with a history of hematologic disease, chronic renal insufficiency (creatinine level >2 mg/dL), and liver disease (active chronic hepatitis or cirrhosis).	• IV TXA<br>• Placebo<br>• Restrictive threshold	Bleeding in the first 24 postoperative hours	Requirement for allogeneic transfusions, thrombotic complications, outcomes, and monitoring of coagulation, fibrinolysis, and inflammation	Unclear	Not stated	None	Non profit
Casati 2004b[59]	• Italy<br>• English<br>• 2004<br>• Single-Centre<br>• 51<br>• Patients scheduled for off-pump coronary artery bypass grafting	Patients with a history of hematologic disease, chronic renal insufficiency (creatinine level >2 mg/dL), and liver disease (active chronic hepatitis or cirrhosis).	• IV TXA<br>• Placebo<br>• Restrictive threshold	Bleeding in the first 24 postoperative hours	Requirement for allogeneic transfusions, thrombotic complications, outcomes, and monitoring of coagulation, fibrinolysis, and inflammation	Unclear	Not stated	None	Non profit
Chakravarthy 2012a[60]	• India<br>• English<br>• 2012<br>• Single Centre<br>• 50<br>• Patients underwent off pump coronary artery bypass surgery	Emergency OPCAB surgery. Pre-existing coagulation disorders, Recent thrombolysis (in less than 2 days), and patients on antiplatelet medications. Hemodynamic instability - heart rate >130, MAP<50, CVP>15, PAWP>23. Patient likely to need cardiopulmonary bypass (such as patients with narrow coronary arteries likely to require endarterectomy, combined valve and coronary surgery) low ejection fraction, recent MI, requirement of intra-aortic balloon pump and	• IV TXA+HES<br>• Placebo<br>• POC testing<br>• Cell salvage	-	Intraoperative blood loss by gravimetric method and postoperative blood loss was measured by calculating blood volume lost in the drains until the time of their removal. Duration on ventilator, length of stay (LOS) intensive care unit (ICU) stay were also assessed. Any adverse events such as seizures was noted.	Unclear	Not stated	Unclear	Not stated

		or mechanical ventilation in the preoperative period. Preoperative anaemia Hb less than 9g/dL. Dysfunctions of major organ such as renal and or hepatic failure. Patients with history of convulsion / or receiving anticonvulsant medications							
Chakravarthy 2012b[60]	• India<br>• English<br>• 2012<br>• Single-Centre<br>• 50<br>• Patients underwent off pump coronary artery bypass surgery	Emergency OPCAB surgery. Pre-existing coagulation disorders, Recent thrombolysis (in less than 2 days), and patients on antiplatelet medications. Hemodynamic instability - heart rate >130, MAP<50, CVP>15, PAWP>23. Patient likely to need cardiopulmonary bypass (such as patients with narrow coronary arteries likely to require endarterectomy, combined valve and coronary surgery) low ejection fraction, recent MI, requirement of intra-aortic balloon pump and or mechanical ventilation in the preoperative period. Preoperative anaemia Hb less than 9g/dL. Dysfunctions of major organ such as renal and or hepatic failure. Patients with history of convulsion / or receiving anticonvulsant medications	• IV TXA+RL<br>• Placebo<br>• POC testing<br>• Cell salvage	-	Intraoperative blood loss by gravimetric method and postoperative blood loss was measured by calculating blood volume lost in the drains until the time of their removal. Duration on ventilator, length of stay (LOS) intensive care unit (ICU) stay were also assessed. Any adverse events such as seizures was noted.	Unclear	Not stated	Unclear	Not stated
Chauhan 2003[61]	• India<br>• English<br>• 2003<br>• Single-Centre<br>• 120	Patients with renal impairment, previous neurological events or congenital bleeding disorders	• IV TXA<br>• No TXA<br>• -	-	Postoperatively, total mediastinal chest tube drainage and blood and blood pr oduct usage at 24 h were recorded. Tests of coagulation including	Unclear	Not stated	Unclear	Not stated

	• Children with cyanotic heart disease				activated clotting time, fibrinogen, fibrin degradation products and platelet count were performed at 6 h postoperatively.				
Chauhan 2004[62]	• India<br>• English<br>• 2004<br>• Single-Centre<br>• 150<br>• Children with congenital cyanotic heart disease	Patients with renal dysfunction, a previous neurological event, or a congenital bleeding disorder	• IV TXA (Induction)<br>• IV TXA (Induction+Infusion)<br>• IV TXA (Induction+bypass+end)<br>• IV TXA (Induction+end)<br>• Placebo<br>• -	-	Postoperative cumulative blood loss was recorded at 24 hours. Use of blood and blood products was noted at 24 hours. Blood samples were collected at 6 hours for tests of coagulation including activated clotting time, fibrinogen, fibrin degradation products, and platelet count.	Unclear	Not stated	Unclear	Not stated
Chen 2013[63]	• China<br>• English<br>• 2013<br>• Single-Centre<br>• 120<br>• Patients undergoing heart valve replacement surgery during cardiopulmonary bypass	Patients with 1) Age greater than 80 years; 2) re-operation; 3) use of hormone and antibiotics 1 week prior to the surgery; 4) preoperative examinations that revealed severe coagulation abnormalities such as significant prolongation of prothrombin time and significant reduction in thrombocytes; 5) severe liver and renal failure; 6) detection of pericardial adhesions during surgery; 7) receipt of treatment with recombinant human coagulation factor VII during and after surgery.	• IV TXA<br>• Ulinastatin<br>• TXA+Ulinastatin<br>• No TXA<br>• -	-	Hospital LOS<br>Perioperative blood loss	Unclear	Not stated	Unclear	Not stated
Choudhuri 2015[64]	• India<br>• English<br>• 2015	Patients undergoing redo-cardiac surgery, with renal insufficiency (serum creatinine higher than 2 mg/dl),	• EACA<br>• IV TXA<br>• No TXA	-	Patients were monitored for twenty-four hours postoperatively to	Unclear	Not stated	Unclear	Not stated

	• Single-Centre<br>• 52<br>• Patients scheduled for open heart surgeries under cardiopulmonary bypass	undergoing ant platelet therapy, having haematological disorders or hepatic dysfunctions	• POC testing		assess reopening rate for the management of excessive bleeding.				
Christabel 2014[65]	• India<br>• English<br>• 2014<br>• Single-Centre<br>• 49<br>• Patients undergoing LeFort 1 osteotomy for correction of dentofacial deformity	Patients with cleft lip, palate, or other facial clefts, systemic disease,<br>bleeding disorders, pregnant or breast feeding mothers, those with known allergy to the test drug or who were under the influence of anticoagulants	• IV TXA<br>• Placebo<br>• -	change in Hb% and PCV at 24 hours	total blood loss by estimation of the total suctioned volume and the amount of soaked gauze minus the volume of saline used.	Unclear	Not stated	None	Not stated
Claeys 2007[66]	• Belgium<br>• English<br>• 2007<br>• Single-Centre<br>• 40<br>• Patients scheduled for primary unilateral total hip replacement surgery for degenerative osteoarthrosis	Patients with an allergy to tranexamic acid preoperative renal or hepatic dysfunction, known bleeding disorders or preoperative coagulation anomalies, anticoagulant or aspirin-like medication and long acting NSAID medication.	• IV TXA<br>• Placebo<br>• -	-	Peroperative blood loss was measured by carefully weighting the swabs and measuring the volumes in the suction bottles during surgery. The number of units of packed cells and the time of transfusion was recorded. All patients were examined daily for clinical signs of DVT.	Unclear	Not stated	Unclear	Not stated
Clagett 1999[67]	• USA<br>• English<br>• 1999<br>• Single-Centre<br>• 100<br>• Patients undergoing elective AAA repair or AFB for occlusive disease	Patients undergoing Thoraco-abdominal or suprarenal aneurysm repair, concomitant renal or visceral artery reconstruction, and reoperative aortic operations; those with congenital or acquired bleeding disorders, creatinine levels higher than 3 mg/dL, significant pre-existing anaemia (haemoglobin level [Hgb] less than 10 g/dL), cirrhosis, and liver failure; those undergoing an	• Intra Cell Salvage<br>• Normal Drainage<br>• -	Total amount of allogeneic blood transfusion per patient during the period of hospitalization and the proportion of patients in whom allogeneic blood was not transfused.	Hematologic parameters, fluid and colloid requirements, morbidity, and mortality.	Unclear	Not stated	Unclear	Not stated

		emergency operation; and those who refused to join the study.							
Coffey 1995[68]	• USA<br>• English<br>• 1995<br>• Single-Centre<br>• 30<br>• Patients who were about to undergo cardiac surgery	Patients undergoing cardiac transplantation or patients with a scram creatinine greater than 3.0 mg/dL	• IV TXA<br>• Placebo<br>• -	-	Shed mediastinal blood and transfused homologous blood were made at 6, 12, and 24 hours postoperatively	Unclear	Not stated	Unclear	Not stated
Corbeau 1995[69]	• France<br>• French<br>• 1995<br>• Single-Centre<br>• 61<br>• Adults undergoing either coronary artery bypass grafting (CABG) or aortic valve replacement	Patients who were: minors, cardiac surgery re-operations, antiplatelet therapy within 10 days before the operation, hereditary or acquired coagulopathy,	• IV TXA<br>• Placebo<br>• -	-	Transfusion requirements within 48 hours	Unclear	Not stated	Unclear	Not stated
Cui 2010[70]	• China<br>• English<br>• 2009<br>• Single-Centre<br>• 31<br>• Cyanotic paediatric patients diagnosed with transposition of the great arteries or double-outlet right ventricle; the operation that the patients underwent was arterial switch operation or double roots transplantation. Haematocrit higher than 54% before operation	History of blood disease; anticoagulation treatment before surgery; medication that affects haemostasis (such as prostaglandin E1); difficult sternal closure caused by anatomical or surgical reasons	• TEG + fibrinogen<br>• Standard of care<br>• Cell Salvage	-	chest closure time (c-T); FFP volume used at closure time (c-FFP); PLT units used at closure time (c-PLT); FFP volume used in the first 24 h in ICU (ICU-FFP); PLTs used in ICU (ICU-PLT); red blood cells (RBCs) used in ICU during the first 24 h (ICU-RBC); total FFP (FFP volume used in operation and in ICU during the first 24 h); total RBC (RBC units used in operation and ICU during the first 24 h);total PLT (PLT units used in closure time and ICU during the first 24 h); chest drainage at 1,	Unclear	Not stated	None	Not stated

					6, and 24 h; mechanical ventilator time; ICU stay; and hospitalization time				
Dadure 2011[71]	• USA<br>• English<br>• 2011<br>• Single-Centre<br>• 39<br>• Children, ASA status 1 or 2, scheduled to undergo surgical correction of craniosynostosis	Children with bleeding diathesis and abnormal prothrombin time, partial thromboplastin time, or platelets counts; a history of convulsive seizures; or allergy to TXA	• IV TXA<br>• Placebo<br>• Iron therapy	-	Perioperative blood loss, number and volume of transfusions, percentage of children who underwent transfusion, and side effects were noted after surgery and at the end of the study.	Unclear	Not stated	Unclear	Not stated
Dalmau 2000[72]	• SPAIN<br>• English<br>• 2000<br>• Single-Centre<br>• 82<br>• Patients underwent orthotopic liver transplantation	Patients with 1) Budd-Chiari syndrome, 2) acute liver failure, 3) early re-transplantation, 4) simultaneous kidney and liver transplantation or renal insufficiency with dialysis, and 5) primary familial amyloid neuropathy.	• IV TXA<br>• Placebo<br>• -	-	The number of units of RBCs, FFP, platelets, and cryoprecipitate transfused were recorded throughout the procedure and during the first 24 h in the intensive care unit.	Unclear	Not stated	Unclear	Not stated
Dalrymple-Hay 1999[73]	• UK<br>• English<br>• 1999<br>• Single-Centre<br>• 112<br>• patients undergoing either coronary artery<br>• bypass grafting, valve replacement/repair operations or a combination of the two	Patients with previous cardiac surgery, emergency operations, patients anticoagulated with warfarin and Jehovah Witness patients.	• Post Cell Salvage<br>• Normal Drainage<br>• -	-	Amount of allogeneic blood transfused.Number of patients transfused allogeneic blood.Mortality.Reoperation for bleeding.Blood loss.Coagulopathy.	Unclear	Not stated	Unclear	Not stated
Damgaard 2010[74]	• Denmark<br>• English<br>• 2010<br>• Single-Centre<br>• 29<br>• Patient undergoing CABG	Off-pump, redo or valve operations, current infection or antibiotic treatment, s-creatinine concentration exceeding 200 mol/L, liver disease, immune disease, and anti-inflammatory or immune-modulating treatment, except	• Intra+Post Cell Salvage<br>• Normal Drainage<br>• Tranexamic acid	patient plasma concentrations of IL-6 at 6, 24, and 72 hours after end of CPB.	plasma concentrations of IL-1b, IL-8, IL-10, IL-12, TNF-, sTNF-RI, sTNF-RII, and procalcitonin at the same intervals; bleeding, allogenic transfusions, cell saver effectiveness regarding	Unclear	Not stated	Unclear	Not stated

		for nonsteroidal anti-inflammatory drugs and aspirin			inflammatory marker reduction, and complications.				
Dell'Amore 2012[75]	• Italy<br>• English<br>• 2012<br>• Single-Centre<br>• 89<br>• Patients, scheduled for pulmonary resection	Re-do surgery anti-platelets or chronic anticoagulant therapy, liver cirrhosis, renal failure (creatinine >2 mg/dl), primary bleeding diathesis (haemophilia, etc.), known allergy to TA, preoperative documented ischaemic heart disease, presence of coronary or other arterial stents, redo surgery, pleuro/pneumonectomy or pleurectomy/decortication for mesothelioma, pleurectomy/decortication for empyema, thoracoscopic surgery, pneumonectomy, neoadjuvant chemotherapy	• IV TXA<br>• Placebo<br>• -	-	Postoperative blood loss from the chest tube was recorded at 12 and 24 h from chest closure.	Unclear	Not stated	Unclear	Not stated
Dietrich 1989[76]	• Germany<br>• English<br>• 1989<br>• Single-Centre<br>• 100<br>• Patients undergoing aorto-coronary bypass	Not-stated	• Cell Salvage<br>• Retransfusion of oxygenator blood<br>• Predonation<br>• Pre-donation +Cell separator<br>• -	-	Amount of blood re-transfused from the cell saver. Amount of allogeneic blood transfused. Number of patients transfused allogeneic blood. Complications. Mortality. ICU length of stay. Blood loss. Re-exploration for bleeding. Operation time. Haematological variables. Hct levels.	Unclear	Not stated	Unclear	Not stated
Diprose 2005[77]	• UK<br>• English<br>• 2005<br>• Single-Centre<br>• 123	Patients with emergency surgery, combined or re-do surgery, the use of two or more antiplatelet therapies within 72 h of surgery, carotid stenosis of >50%, any chronic	• IV TXA<br>• Aprotinin<br>• Placebo<br>• Cell salvage	Number of patients in each group exposed to allogeneic red cell transfusion, allogeneic coagulation	Mediastinal drain losses and markers of myocardial injury.	Unclear	Not stated	any	Blood service

	• Patients undergoing first-time cardiac surgery	inflammatory process, steroid therapy, liver disease, or any patient not prepared to receive an allogeneic transfusion		product transfusion or any allogeneic transfusion (allogeneic red cell and/or allogeneic coagulation product) during their hospital stay.					
Eftekharian 2014[78]	• Iran<br>• English<br>• 2014<br>• Single-Centre<br>• 56<br>• Patients who underwent orthognathic surgery	Patients with coagulopathy, those who used anticoagulants, and those requiring additional procedures	• IV TXA<br>• No TXA<br>• -	Blood loss	Age, gender, surgical time, the amount of irrigation solution used, baseline hemoglobin and hematocrit, and weight	Unclear	Not stated	Unclear	Not stated
Ekback 2000[79]	• Sweden<br>• English<br>• 2000<br>• Single-Centre<br>• 40<br>• Patients undergoing total hip replacement	Not stated	• IV TXA<br>• Placebo<br>• Restrictive threshold<br>• Cell salvage	-	-	Unclear	Not stated	Any	Industry
El Shal 2015[80]	• Egypt<br>• English<br>• 2015<br>• Single-Centre<br>• 90<br>• Patients ASA I-II aged from 18 to 50 years and undergoing functional endoscopic sinus surgery	Patients with uncontrolled hypertension, renal or hepatic dysfunction, coronary or cerebral artery disease, autonomic disturbance, deep vein thrombosis or peripheral vascular disease, bleeding diathesis and patients receiving anticoagulants were excluded from the study	• IV TXA<br>• EACA<br>• No TXA<br>• -	-	The duration of surgery, volume of blood loss, pre and postoperative haemoglobin, MAP and HR, surgical field quality surgeon satisfaction and side effects	Unclear	Not stated	Unclear	Not stated
Elawad 1991[81]	• Sweden<br>• English<br>• 1991<br>• Single-Centre<br>• 40<br>• Patients undergoing primary hip arthroplasty	Not stated	• Post Cell Salvage<br>• Control Group<br>• -	-	Amount of allogeneic units transfused. Number of patients receiving allogeneic blood. Complications. Blood loss. Haematological variables.	Unclear	Not stated	None	Not stated

Engel 2001[82]	• Germany<br>• English<br>• 2001<br>• Single-Centre<br>• 36<br>• Patients underwent total knee arthroplasty	Not stated	• IV TXA<br>• Aprotinin<br>• Placebo<br>• -	-	-	Unclear	Not stated	Unclear	Not stated
Felli 2019[83]	• Italy<br>• English<br>• 2016<br>• Single-Centre<br>• 80<br>• All patients at our study location who received a diagnosis of ACL rupture	Patients younger than 18 years or older than 45 years, coagulative disorders, renal impairment, treatment with drugs interfering with coagulation or TXA clearance, and thrombophilia. Also excluded were patients with a history of thrombotic disease, seizures, or ACL revision surgery; patients with a history of knee surgery on the affected knee; patients with multiligament injuries; and patients who received concomitant extra-articular anterolateral procedures.	• IV TXA<br>• Placebo<br>• -	The drained blood volume on PD 1	Clinical data including the patellar circumference, ROM, quadriceps strength (QS), pain assessed with a visual analog scale (VAS), clinical grade of hemarthrosis, International Knee Documentation Committee (IKDC) score, and Lysholm score.	Unclear	Not stated	Unclear	Not stated
Garneti 2004[84]	• UK<br>• English<br>• 2004<br>• Single-Centre<br>• 50<br>• Patients who underwent total hip arthroplasty	Not stated	• IV TXA<br>• No TXA<br>• -	-	-	Unclear	Not stated	Unclear	Not stated
Ghaffari 2012[85]	• Iran<br>• English<br>• 2012<br>• Single-Centre<br>• 100<br>• Patients undergoing on-pump coronary artery bypass graft surgery (CABG)	History of haemorrhagic tendency and blood dyscrasia, history of Plavix use, known hepatic, renal, and metabolic diseases, use of other anti-coagulation drugs like Coumadin for valvular disease and arrhythmias and streptokinase, emergency surgery, rheumatic heart	• IV TXA<br>• Placebo<br>• -	-	The amounts of mediastinal and plural blood shed were measured after six, twelve, and twenty-four hours. Postoperative complications like postoperative myocardial	Unclear	Not stated	Unclear	Not stated

		disease, known allergy to Aprotinin or Transamine and prohibition for their use on the grounds of acquired visual defects and retinal disease, subarachnoid haemorrhage, disseminated intravascular coagulation, gall bladder disease, leukaemia, embolization, and vein thrombosis			infarction (based on rise in cardiac enzyme, change in ECG, and change in the ejection fraction estimated by echocardiography), neurological complications (estimated by clinical examination and CT-scanning), redo-operations for surgical bleeding and pericardial effusion, kidney complications (rise in serum creatinine and low urinary output < 0.5 cc per minute), and other complications were studied.				
Gill 2009[86]	• USA<br>• English<br>• 2007<br>• Single-Centre<br>• 10<br>• Patients who underwent total hip arthroplasty	Patients in need of primary total hip arthroplasty or those with a known prosthetic infection, a bleeding or coagulation disorder, renal insufficiency (serum creatinine>two standard deviations for age), or history of deep venous thrombosis or pulmonary embolism.	• IV TXA<br>• Placebo<br>• Cell salvage	All blood transfusions given	Chest drain output at 48 hours.	Unclear	Not stated	None	Non profit
Good 2003[87]	• Sweden<br>• English<br>• 2003<br>• Single Centre<br>• 51<br>• Patients with osteoarthritis and who had unilateral cemented total knee arthroplasty using spinal anaesthesia	Patients with a history of coagulopathy, an abnormally great prothrombin or activated partial thrombin time, previous history of a thromboembolic event, treatment with aspirin or non-steroidal anti-inflammatory agents (NSAID) in the previous week, plasma creatinine greater than 115 mmol/litre in men and 100	• IV TXA<br>• Placebo<br>• -	-	-	Unclear	Not stated	None	Non profit

		mmol/litre in women, acute infection (e.g. with leucocytosis or fever), and malignant disease, patients with myocardial infarction in the preceding 12 months, those with unstable angina or coronary disease, patients given plasma or other treatment affecting coagulation during the perioperative period.							
Gregersen 2015[88]	• Denmark<br>• English<br>• 2015<br>• Single-Centre<br>• 284<br>• Patients (aged ≥ 65 years) admitted from nursing homes or sheltered housing facilities for unilateral hip fracture surgery and with postoperative Hb levels between 9.7 g/dL (6 mmol/L) and 11.3 g/dL (7 mmol/L) during the first 6 postoperative days.<br>• Restrictive threshold 9.7g/dl	Exclusion criteria were: active cancer, pathological fractures, and inability to understand or speak Danish without an interpreter, refusal of RBC transfusion (e.g. Jehovah's Witness), fluid overload, irregular erythrocyte antibodies, or previous participation in the trial.	• Restrictive 97g/L<br>• Liberal<br>• -	recovery from physical disabilities	total number of infections (pneumonia, urinary tract infection, other), cognition, depression, quality of life, modified Barthels index, and comprehensive frailty index	Unclear	Not stated	None	Non profit
Greiff 2012[89]	• Norway<br>• English<br>• 2008<br>• Single-Centre<br>• 63<br>• Patients, 70 years or older, undergoing combined aortic valve replacement and CABG surgery	Patients receiving treatment with heparin or low–molecular-weight heparin, oral anticoagulants, nonsteroidal anti-inflammatory drugs, platelet inhibitors other than aspirin, or systemic glucocorticoids. Patients with abnormal kidney function (serum creatinine >140 µmol/L) or liver dysfunction with	• IV TXA<br>• Placebo<br>• Cell salvage	-	-	Unclear	Not stated	Unclear	Not stated

		international normalized ratio (INR) >1.5							
Hajjar 2010[90]	• Belgium<br>• English<br>• 2010<br>• Single-Centre<br>• 502<br>• Patients who were undergoing CABG surgery or cardiac valve replacement or repair, alone or in combination.<br>• Restrictive threshold Haematocrit>24%	Patients were excluded for any of the following reasons: younger than 18 years; surgery without cardiopulmonary bypass; emergency procedure; ascending and descending thoracic aortic procedures; left ventricular aneurysm resection; inability to receive blood products; enrolment in another study; chronic anaemia (preoperative haemoglobin concentration less than 10 g/dL); low platelet count (preoperative platelet count less than 150 ×103/μL); coagulopathy (previous history or prothrombin time longer than 14.8 seconds); pregnancy; neoplasm; endocarditis; congenital heart defect; hepatic dysfunction (total bilirubin value higher than 1.5 mg/dL [to convert to μmol/L, multiply by 17.104]); end-stage renal disease (receiving chronic dialysis therapy); and refusal to consent.	• Restrictive 80g/L<br>• Liberal<br>• -	30-day all-cause mortality and severe morbidity (cardiogenic shock; ARDS or acute renal injury requiring dialysis or haemofiltration; respiratory, cardiac, neurologic, and infectious complications; inflammatory complications; bleeding; ICU and hospital lengths of stay, RBC transfusions)	-	Unclear	Not stated	None	Not stated
Hardy 1998[91]	• Canada<br>• English<br>• 1994<br>• Single-Centre<br>• 88<br>• patients older than 18 years scheduled to undergo<br>• elective CABG	Patients allergic to one of the study medications, patients seen with microscopic or macroscopic haematuria, or patients with an un-correctable defect of haemostasis preoperatively	• IV TXA<br>• Placebo<br>• Restrictive threshold	-	The total volume of mediastinal blood shed after the operation and collected until removal of drains (over 12 to 18 hours) was measured hourly by the ICU nurses. Transfusions of packed red blood cells (PRBCs) and haemostatic blood	Unclear	Not stated	Any	Industry

					products (platelets, FFP, or cryoprecipitates) during and after the operation were recorded.				
Hiippala 1995[92]	• Finland<br>• English<br>• 1994<br>• Single-Centre<br>• 28<br>• Patients underwent total knee arthroplasty	Not stated	• IV TXA<br>• Placebo<br>• -	-	Blood loss during surgery, in the recovery room and on the surgical ward was recorded, together with the number of units of blood transfused in hospital	Unclear	Not stated	Unclear	Not stated
Hiippala 1997[93]	• Finland<br>• English<br>• 1996<br>• Single-Centre<br>• 77<br>• Patients scheduled for total knee arthroplasty	Not stated	• IV TXA<br>• Placebo<br>• -	-	Perioperative blood loss gathered in surgical gauzes, suction reservoirs, and postoperative drainage system was measured. The number of transfusions given during hospitalization was registered.	Unclear	Not stated	Unclear	Not stated
Horrow 1990[94]	• USA<br>• English<br>• 1990<br>• Single-Centre<br>• 38<br>• Patients undergoing cardiac operation	Patients with a history of bleeding disorder, those who received aspirin, warfarin, heparin, dipyridamole, streptokinase, NSAID within 7 days of surgery.	• IV TXA<br>• Placebo<br>• Restrictive threshold<br>• Cell salvage	-	-	Unclear	Not stated	Unclear	Not stated
Horrow 1991[95]	• USA<br>• English<br>• 1991<br>• Single-Centre<br>• 81<br>• Patients undergoing cardiac surgery	Patients who took warfarin or oestrogens within 7 days of surgery; had active haematuria, a serum creatinine concentration of 2 mg-/dl or more, or a personal or family history of abnormal bleeding; or underwent intra-aortic balloon counter-pulsation.	• IV TXA<br>• Placebo<br>• -	-	Blood loss consisted of mediastinal tube drainage over 12 hours. Follow-up visits sought evidence of myocardial infarction and stroke.	Unclear	Not stated	None	Non profit

Horrow 1995[96]	• USA<br>• English<br>• 1995<br>• Single-Centre<br>• 148<br>• Patients undergoing cardiac operation with extracorporeal circulation	Patients who took warfarin or oestrogens within 7 days of surgery; had active haematuria, a serum creatinine concentration of 2 mg-/dl or more, or a personal or family history of abnormal bleeding; or underwent intra-aortic balloon counter-pulsation before surgery	• IV TXA<br>• Placebo<br>• Restrictive threshold	-	The blood loss via mediastinal and pleural drains, transfusion of packed erythrocytes.	Unclear	Not stated	None	Non profit
Horstmann 2014[97]	• Netherlands<br>• English<br>• 2014<br>• Single-Centre<br>• 118<br>• Patients undergoing primary total hip arthroplasty	coagulation disorders, including deep venous thrombosis and pulmonary embolism; malignancy; ongoing infections; untreated hypertension; unstable angina pectoris; myocardial infarction within the past 12months; coronary bypass surgery within the past 12 months; renal dysfunction; anticoagulant intake or participation in other clinical trials dealing with any drugs that affect blood loss.	• Post Cell Salvage<br>• Normal Drainage<br>• -	Hb level on the first postoperative day	Hb levels on the second and third postoperative days, the lowest postoperative Hb level, blood loss during surgery, volume of intraoperatively suctioned and re-transfused blood, volume of re-transfused drained wound blood, allogeneic blood transfusions, postoperative pain, hospital stay, adverse events and total blood loss.	Unclear	Not stated	Unclear	Not stated
Hou 2015[98]	• China<br>• Chinese<br>• 2014<br>• Single-Centre<br>• 40<br>• Patients who were candidates for unilateral cemented total knee replacement	-	• IA TXA<br>• IV TXA<br>• Placebo<br>• -	-	Blood loss, hidden blood loss, blood transfusion ratio and per capita of each group were compared. Clinical symptoms of pulmonary embolism and lower limb deep vein thrombosis were observed	Unclear	Not stated	Unclear	Not stated
Hu 2018[99]	• China<br>• Chinese<br>• 2018<br>• Single-Centre	-	• IV TXA (high dose)<br>• IV TXA (low dose)	-	The intraoperative blood loss, haemoglobin level at postoperative 24 and 48 hours, postoperative drainage	Unclear	Not stated	None	Non profit

	• 105<br>• Patients with unilateral knee osteoarthritis undergoing total knee arthroplasty		• No TXA<br>• -		volume and incidence of deep venous thrombosis were recorded.				
Huang 2015[100]	• China<br>• Chinese<br>• 2013<br>• Single-Centre<br>• 60<br>• Patients who underwent total knee arthroplasty	-	• IV TXA<br>• No TXA<br>• -	-	The amount of drainage, the total blood loss, the hidden blood loss, the postoperative Hgb, the amount of blood transfusion, the ratio of blood transfusion, and the incidence of vein thrombosis embolism (VTE) were compared between 2 groups.	Unclear	Not stated	Unclear	Not stated
Imai 2012[101]	• Japan<br>• English<br>• 2011<br>• Single-Centre<br>• 117<br>• Patients with osteoarthritis of hip, undergoing total hip arthroplasty	Patients with a history of ischemic heart disease, severe chronic heart failure, hepatic dysfunction, chronic renal failure on haemodialysis, cerebral infarction, or bleeding disorder as well as those who were currently receiving anticoagulant therapy	• No TXA<br>• IV TXA (1 Post-op dose)<br>• IV TXA (2 Post-op doses)<br>• IV TXA (Pre-op)<br>• IV TXA (Pre-+Post-op)<br>• No TXA<br>• -	-	Intra- and Postoperative blood loss;<br>Complications.	Unclear	Not stated	Unclear	Not stated
Ishida 2011[102]	• Japan<br>• English<br>• 2011<br>• Single-Centre<br>• 100<br>• Osteoarthritis patients with total knee arthroplasty	Those with rheumatoid arthritis, revision TKA and simultaneous bilateral TKA	• IV TXA<br>• Placebo<br>• -	-	-	Unclear	Not stated	Unclear	Not stated
Jansen 1999[103]	• Belgium<br>• English<br>• 1999<br>• Single-Centre<br>• 42	Rheumatoid arthritis, malignancy, previous thrombo-embolic episodes, ischemic heart disease, previous subarachnoid bleeding, haematuria and body weight > 100 kg.	• IV TXA<br>• No TXA<br>• -	-	Blood Loss<br>Use of tranexamic acid for an effective blood conservation strategy after total knee arthroplasty	Unclear	Not stated	Any	Industry

	• Patients after total knee arthroplasty								
Jares 2003[104]	• Czech Republic<br>• English<br>• 2003<br>• Single-Centre<br>• 47<br>• Patients undergoing coronary artery bypass grafting on the beating heart	Impaired renal function (Cr> 150mmol/l), haematological disease, Pre-op anaemia (Hb <11g/dl, Htc<32) and conversion to CPB	• IV TXA<br>• Placebo<br>• Restrictive threshold	-	Preoperative haematological variables, postoperative blood loss at 4 and 24 hours, transfusion requirements of packed red blood cells, and postoperative thrombotic events such as a myocardial infarction, stroke and pulmonary embolism were recorded.	Unclear	Not stated	Unclear	Not stated
Jaszczyk 2015[105]	• Poland<br>• English<br>• 2015<br>• Single-Centre<br>• 124<br>• Patients undergoing total cementless hip arthroplasty	Patients with contraindications to intravenous TXA administration, i.e. allergy to TXA, deep vein thrombosis, a history of pulmonary embolism, arterial thrombosis, angina, a history of myocardial infarction or stroke, fibrinolysis secondary to consumption coagulopathy, severe kidney and liver failure, and a history of seizures.	• IV TXA<br>• No TXA<br>• -	-	Intraoperative blood loss (volume of blood in the aspirator), postoperative blood loss (volume of blood drained), total perioperative blood loss, and the number of patients requiring transfusion as well as the number of thromboembolic complications in both groups.	Unclear	Not stated	Unclear	Not stated
Kakar 2009[106]	• India<br>• English<br>• 2009<br>• Single-Centre<br>• 25<br>• Total knee replacement patients	Patients were excluded if they had one of the following criteria: known or suspected allergy to medications used (TAX, local anaesthetics, midazolam, pethidine, Propofol), inherited or acquired haemostatic diseases, abnormal coagulation screening tests (platelet count, prothrombin time, activated partial thromboplastin time),	• IV TXA<br>• Placebo<br>• -	-	The postoperative blood loss, transfusion requirement, cost effectiveness and complications were noted.	Unclear	Not stated	Unclear	Not stated

		ingestion of aspirin or other nonsteroidal anti-inflammatory drugs within seven days of surgery, renal or hepatic insufficiency, pregnancy, history of deep venous thrombosis (DVT) or pulmonary embolism or history of ocular pathology or ophthalmological procedure other than corrective lenses.							
Karimi 2012[107]	• USA<br>• English<br>• 2012<br>• Single-Centre<br>• 32<br>• Patients scheduled for elective bi-maxillary osteotomy	Not stated	• IV TXA<br>• Placebo<br>• -	-	Intraoperative blood loss, pre and post-operative haemoglobin (Hb) and haematocrit (Hct) concentration, duration of surgery, hospital stay time, and rate of blood transfusion were recorded	Unclear	Not stated	Unclear	Not stated
Karski 2005[108]	• Canada<br>• English<br>• 2005<br>• Single-Centre<br>• 312<br>• Patients undergoing cardiac surgery	Patients with a history of claustrophobia; known contraindications to magnetic resonance imaging (MRI); bleeding disorders; preoperative haemoglobin less than 135 g/L; symptomatic peripheral vascular disease; connective tissue disease; age older than 80 years; impaired renal function (creatinine 2.0 mg/dL); active liver disease; known allergies to TA, aspirin, or contrast dye (Omnipaque; Sterling Winthrop, Inc, Collegeville, Pa); or left ventricular function ejection fraction less than 20%	• IV TXA<br>• Placebo<br>• -	Graft patency	-	Unclear	Not stated	Any	Industry
Karski1995[109]	• Canada<br>• English	Not stated	• IV TXA<br>• Placebo	-	-	Unclear	Not stated	Any	Industry

	• 1995<br>• Single-Centre<br>• 98<br>• Patients undergoing cardiopulmonary bypass		• -						
Kaspar 1997[110]	• USA<br>• English<br>• 1997<br>• Single-Centre<br>• 27<br>• Patients underwent orthotopic liver transplantation	Not stated	• IV TXA<br>• Placebo<br>• Cell salvage	-	Intraoperative transfusion requirements were recorded during the procedure and for the first 24 h postoperatively. A record was kept of any intraoperative epsilon-aminocaproic acid administered for uncontrolled fibrinolysis.	Unclear	Not stated	Unclear	Not stated
Katoh 1997[111]	• Japan<br>• English<br>• 1997<br>• Single-Centre<br>• 62<br>• Patients undergoing either coronary artery bypass grafting or heart valve operation	Not stated	• IV TXA<br>• Placebo<br>• -	-	Mediastinal blood loss during the operation, but after discontinuation of CPB and drainage from mediastinal tubes for the first 24 hours after operation were measured.	Unclear	Not stated	Unclear	Not stated
Katsaros 1996[112]	• USA<br>• English<br>• 1993<br>• Single-Centre<br>• 210<br>• Patients who had first time CABG, valve replacement and reoperation with cardiopulmonary bypass	Previous pulmonary embolism, Takayasu's arteritis, and known allergy to TXA	• IV TXA<br>• No TXA<br>• Restrictive threshold	-	Shed mediastinal blood was measured for the first 24 hours postoperatively.	Unclear	Not stated	None	Non profit
Keyhani 2016[113]	• Iran<br>• English<br>• 2014<br>• Single-Centre	Patients with coagulation disorders, history of cardiovascular diseases, history of cerebrovascular disorders, history of thromboembolic	• IV TXA<br>• No TXA<br>• -	Volume of bleeding based on the amount of drainage, the level of Hb at 24	All complications	Unclear	Not stated	Unclear	Not stated

	• 80<br>• Patients who underwent primary total knee arthroplasty	problems, renal and hepatic diseases, pregnant women, anaemia, abnormal thrombin and prothrombin time, and abnormal platelet counts		postoperative hours, the frequency of transfusion, and the number of packed red blood cells transfused.					
Kim 2014[114]	• Korea<br>• English<br>• 2014<br>• Single-Centre<br>• 146<br>• Patients who underwent total knee arthroplasty	Patients with a diagnosis other than primary OA, those with an acquired or congenital coagulopathy, those on current anticoagulation therapy, those with preoperative hepatic or renal dysfunction or severe ischaemic heart disease, and those with a history of thromboembolic disease	• IV TXA<br>• No TXA<br>• Iron therapy<br>• Restrictive threshold	total blood loss and the allogenic transfusion rate.	rate of autologous transfusion with preoperative autologous blood donation, blood loss via the drain, postoperative Hb drop, proportions of patients with the Hb level below the three cut-off values, namely 7.0, 8.0, and 9.0 g/dL, the incidences of symptomatic DVT and PE, and functional outcomes.	Unclear	Not stated	Unclear	Not stated
Klein 2008[115]	• UK<br>• English<br>• 2008<br>• Single-Centre<br>• 213<br>• Nonemergency first time CABG, valve surgery or combined CABG, and valve procedures requiring cardiopulmonary bypass (CPB)	Patient refusal to receive blood or blood products; previous cardiac or thoracic surgery; known coagulation disorders; contraindication to antifibrinolytic; participation in another trial of an investigational drug or device; or specific request for cell salvage by the operating surgeon. Operations associated with a high risk of transfusion, such as transplantation and operations on the thoracic aorta were excluded	• Cell Salvage<br>• Control Group<br>• Tranexamic acid	any allogeneic blood transfusion.	the number of units of RBCs, FFP, or platelets transfused. Serious adverse events, hematology, and biochemistry variables (sampled preoperatively and at 1 h, 24 h, and 5 days after operation) were recorded to monitor safety.	Unclear	Not stated	Any	Industry
Koch 2017[116]	• USA<br>• English<br>• 2017<br>• Multi-Centre	Not Stated	• Restrictive 80g/L<br>• Liberal<br>• -	composite of postoperative morbidities and mortality.	lengths of ICU and postoperative hospital stays, number of RBC units transfused, and	Unclear	Not stated	None	Non profit

	• 717<br>• Patients aged 18 years and older scheduled for elective isolated heart valve procedures, coronary artery bypass graft surgery (CABG) with or without valve procedures, and ascending aorta replacement performed on CPB at two centres: Cleveland Clinic (USA) and SAL Hospital (India).<br>• Restrictive threshold Haematocrit <24%				individual components of the composite.				
Kojima 2001[117]	• Japan<br>• English<br>• 2001<br>• Single-Centre<br>• 22<br>• Patients undergoing cardiopulmonary bypass surgery	Patients on medication likely to influence coagulation and fibrinolysis, as well as those with renal or hepatic dysfunction.	• IV TXA<br>• Placebo<br>• -	-	Intraoperative blood loss was assessed by estimated blood volume on drapes, weighing surgical gauzes, and measuring suction bottle returns. Postoperative blood loss during 24 h after surgery was measured from mediastinal and chest tube drainage following surgery. Blood products were transfused according to a standard protocol.	Unclear	Not stated	Unclear	Not stated
Kuitunen 2006[118]	• Finland<br>• English<br>• 2006<br>• Single-Centre<br>• 30<br>• Patients who underwent cardiac surgery	Patients with preoperative coagulation disorders, renal or hepatic failure or medication with Coumarin anticoagulants, Heparin or Acetosalicylic acid within the previous 5 days.	• IV TXA<br>• Placebo<br>• POC testing	-	Perioperative blood loss	Unclear	Not stated	None	Non profit
Kumar 2013[119]	• India<br>• English<br>• 2012	Patients with a serum creatinine greater than 1.5 mg/dl and specific	• IV TXA<br>• No TXA	perioperative total blood loss	Complications associated with PCNL, and to study the factors	Unclear	Not stated	Unclear	Not stated

	• Single-Centre<br>• 200<br>• Patients undergoing percutaneous nephrolithotomy	contraindications to tranexamic acid, namely hypersensitivity to the drug, active intravascular clotting, acquired defective colour vision and subarachnoid haemorrhage.	• Restrictive threshold		influencing blood loss and the safety of tranexamic acid in PCNL				
Later 2009[120]	• Netherlands<br>• English<br>• 2006<br>• Single-Centre<br>• 202<br>• Patients scheduled for low or intermediate risk first time heart surgery with use of cardiopulmonary bypass	Patients with previous sternotomy, known bleeding disorders, an abnormal preoperative coagulation profile for reasons other than anticoagulant therapy, or treatment with antiplatelet agents within 5 days before surgery.	• IV TXA<br>• Placebo<br>• Aprotinin<br>• Restrictive threshold; Cell salvage	postoperative blood loss and transfusion requirements	In-hospital mortality, morbidity, and length of intensive care and hospital stay.	Unclear	Not stated	None	Non profit
Laub 1993[121]	• USA<br>• English<br>• 1993<br>• Single-Centre<br>• 38<br>• Patients undergoing primary coronary revascularization between July and December 1989	Not stated	• Cell Salvage<br>• Control Group<br>• -	-	Amount of blood re-transfused from the cell saver. Number of patients transfused allogeneic blood. Amount of allogeneic blood transfused. Amount of any blood product transfused.	Unclear	Not stated	Unclear	Not stated
Lee 2013a[122]	• Korea<br>• English<br>• 2011<br>• Single-Centre<br>• 72<br>• Osteoarthritis patients undergoing unilateral total knee arthroplasty	Patients who had (1) planned bilateral knee or multiple joint replacements, (2) evidence of chronic or acute preoperative DVT on colour Doppler ultrasonography, (3) rheumatoid arthritis, haemophilia or post-traumatic osteoarthritis, (4) history of thromboembolic disease, (5) renal insufficiency (serum creatinine [1.5 mg/dL), (6) severe cardiovascular or respiratory disease, (7) severe ischaemic or heart disease, (8) acquired disturbances of colour	• IV TXA<br>• Placebo<br>• Restrictive threshold<br>• Cell salvage	-	Post-operative retransfusion volume, allogenic transfusion volume and drain amount were recorded for each patient. Ecchymosis around the operative leg was assessed. The level of haemoglobin, prothrombin time, activated partial thromboplastin time and D-dimer was recorded before and on the first, second and	Unclear	Not stated	None	Not stated

		vision, (9) preoperative anaemia (a haemoglobin value \11 g/dL in females and \12 g/dL in males), (10) congenital or acquired coagulopathy, or (11) preoperative use of anticoagulant therapy within 5 days before surgery			fifth days after operation. The incidence of total venous thromboembolism (DVT total, proximal and distal and symptomatic pulmonary embolism) and mortality was evaluated from all causes up to day 7.				
Lee 2013b[123]	• Korea<br>• English<br>• 2013<br>• Single-Centre<br>• 68<br>• Adults, ASA status 1 and 2, undergoing primary unilateral cementless total hip replacement	Patients older than 70 years, those with previous hip surgery, drug sensitivity, anaemia (haemoglobin [Hb] b 12 g/ dL for men and b 11 g/dL for women), coagulopathy, thrombocytopenia, hepatic or renal failure, history of deep vein thrombosis (DVT) or embolism, severe aortic or mitral valve stenosis, or neurological or cerebrovascular disease	• IV TXA<br>• Placebo<br>• -	-	Intraoperative blood loss was measured using the difference between the weights of used gauze and the original unused gauze, in addition to the blood volume accumulated in suction bottles. Postoperative blood loss was considered to be the amount of blood accumulated in drainage bags.	Unclear	Not stated	Unclear	Not stated
Lemay 2004[124]	• Canada<br>• English<br>• 2004<br>• Single-Centre<br>• 39<br>• Patients undergoing primary unilateral total hip replacement	History of previous ipsilateral hip surgery, known or suspected allergy to medications used (TA, local anaesthetics, Midazolam, Fentanyl, Propofol, or Dalteparin), anaemia [haemoglobin (Hb) < 115 g/L for women, Hb < 130 g/L for men], inherited or acquired haemostatic diseases, abnormal coagulation screening tests (platelet count, prothrombin time, activated partial thromboplastin time), ingestion of aspirin or other nonsteroidal anti-inflammatory	• IV TXA<br>• Placebo<br>• -	intraoperative and total blood losses	-	Unclear	Not stated	Unclear	Not stated

		drugs within seven days of surgery, renal (serum creatinine > two standard deviation for age) or hepatic insufficiency, pregnancy, history of deep venous thrombosis (DVT) or pulmonary embolism as well as a history of ocular pathology or ophthalmological procedure other than corrective lenses							
Li 2015[125]	• China<br>• Chinese<br>• 2014<br>• Single-Centre<br>• 224<br>• Patients who underwent unilateral primary total hip arthroplasty	-	• IV TXA<br>• Placebo<br>• -	-	Total blood loss, total volume of drainage and transfusion were recorded. Postoperative deep vein thrombosis and other complications was also measured.	Unclear	Not stated	Unclear	Not stated
Liang 2016[126]	• China<br>• English<br>• 2015<br>• Single-Centre<br>• 60<br>• Patients undergoing surgery for multilevel posterior lumbar degenerative procedures	Allergy to TXA, anaemia (male haemoglobin <13 g/dl, female haemoglobin <12 g/dl), coagulopathy, treatment with anticoagulants or antiplatelet agents, history of thromboembolic events (deep vein thrombosis, ischemic heart disease, pulmonary embolism, transient ischemic attack, strokes, subarachnoid haemorrhage), renal impairment (creatinine >2.0 mg/dl), chronic liver disease, and pregnancy. We also excluded patients more than 65 years of age because elderly patients usually limited their activities and are more prone to have deep vein thrombosis.	• Top TXA<br>• Placebo<br>• Restrictive threshold	-	Data were collected on demographics, pre-operative investigations, blood loss, and blood products transfusedduring surgery.	Unclear	Not stated	Unclear	Not stated
Lin 2015[127]	• Taiwan<br>• English	(1) allergy to TXA; (2) a known history of thromboembolic	• Top TXA<br>• IV TXA	-	Postoperative Hb levels, Hb drop, total drain	Unclear	Not stated	Unclear	Not stated

	• 2013<br>• Single-Centre<br>• 120<br>• Patients who underwent total knee arthroplasty	disease; (3) preoperative renal or hepatic dysfunction; (4) cardiovascular disease (a history of myocardial infarction or angina); (5) cerebral vascular disease (a history of stroke); (6) preoperative anaemia (a haemoglobin (Hb) value less than 11 g/dL in female and less than 12 g/dL in male); and (7) preoperative coagulopathy (a platelet count less than 150,000/mm3 or an international normalized ratio greater than 1.4)	• Placebo<br>• -		amount, total blood loss, and transfusion rate.				
Lotke 1999[128]	• USA<br>• English<br>• 1999<br>• Single-Centre<br>• 127<br>• Patients undergoing primary TKA who were able to donate 2 units of blood pre-operatively<br>• Restrictive threshold 9g/dl	-	• Restrictive 90g/L<br>• Liberal<br>• -	-	Complications, cardiac events,Hb levels, blood usage (units),mental confusion, lethargy, orthostatic hypotension, number of participants transfused	Unclear	Not stated	Unclear	Not stated
Macgillivray 2011[129]	• UAE<br>• English<br>• 2011<br>• Single-Centre<br>• 60<br>• Patients presenting for concurrent total knee arthroplasty	Patients with known allergy to TXA, a history of hepatic or renal dysfunction, severe cardiac or respiratory disease (myocardial infarction within 6 months, unstable angina, aortic or mitral valvular stenosis), previous stroke, congenital or acquired coagulopathy, or history of thromboembolic disease.	• IV TXA (low dose)<br>• IV TXA (high dose)<br>• Placebo<br>• Cell salvage	-	Risk of RBC transfusion Perioperative blood loss	Unclear	Not stated	None	Not stated
Maddali 2007[130]	• Oman<br>• English<br>• 2005<br>• Single-Centre<br>• 222	Patients requiring concomitant non-coronary procedures and those with a history of bleeding diathesis or known coagulation factor deficiency	• IV TXA<br>• Placebo<br>• POC testing	-	Postoperative drainage and transfusion requirements were measured in all patients.	Unclear	Not stated	Unclear	Not stated

	• Patients undergoing on-pump primary coronary bypass surgery								
Malhotra 2011[131]	• India<br>• English<br>• 2011<br>• Single-Centre<br>• 50<br>• Patients undergoing total hip arthroplasty	Patients with a history of severe ischemic heart disease, chronic renal failure, cirrhosis of the liver, and bleeding disorders, as well as those who were currently receiving anticoagulant therapy	• IV TXA<br>• Placebo<br>• -	-	The intraoperative and postoperative blood loss and the number of blood transfusions required were recorded.	Unclear	Not stated	None	Not stated
Marberg 2010[132]	• Sweden<br>• English<br>• 2010<br>• Single-Centre<br>• 77<br>• Elective CABG patients	Known liver, kidney or bleeding disorder, perioperative use of Aprotinin or Clopidogrel treatment within 5 days before surgery.	• Post Cell Salvage<br>• Normal Drainage<br>• Tranexamic acid	bleeding during the first 12 postoperative hours.	postoperative transfusion requirements, haemoglobin levels, thrombo-elastometric variables and plasma concentrations of interleukin-6, thrombin—anti-thrombin complex and D-dimer. R	Unclear	Not stated	None	Not stated
Markatou 2012[133]	• Greece<br>• English<br>• 2012<br>• Single-Centre<br>• 58<br>• Patients scheduled for major abdominal surgery<br>• Restrictive threshold 7.7g/dl	history of bleeding diathesis associated with thrombocytopenia, hereditary haemostatic defects such as haemophilia or chronic anticoagulant administration, refusal of transfusions for religious reasons, ischemic heart disease (unstable angina or myocardial infarction within the last six months), and pre-existing infectious or autoimmune diseases as well use of corticosteroids or immunosuppressive drugs within the last six months	• Restrictive 77g/L<br>• Liberal<br>• -	Units of red blood cells (RBC) per patient and the incidence of transfused patients in each group	Clinical outcome measures, as expressed by time to patient mobilization, time of first liquid and solid food intake and duration of hospital stay.	Unclear	Not stated	Unclear	Not stated
McGill 2002[134]	• USA<br>• English<br>• 2002<br>• Single-Centre	Emergency operation<br>Redo procedures and multiple procedures<br>Known carotid stenosis > 50%	• Cell salvage<br>• Cell salvage+normov	-	Number of patients transfused allogeneic blood. Number of patients receiving any	Unclear	Not stated	Any	Blood service

	• 168<br>• Age 18-80 years Ejection fraction > 30%, Serum creatinine concentration < 150 umol/l, International normalised ratio and activated partial, thromboplastin time < 1.5, Platelet count > 150 × 10^9/l, Haemoglobin concentration > 120 g/l, Haematocrit > 0.36, Weight > 60 kg	Myocardial infarction in past three weeks<br>Heparin or warfarin taken in previous five days<br>Antiplatelet treatment other than aspirin<br>Cerebrovascular disease<br>History of liver disease<br>Jehovah's Witnesses	olaemic haemodilution<br>• Control Group<br>• Tranexamic acid		blood product. Amount of allogeneic blood transfused. Blood loss. Re-operation for bleeding. Hospital length of stay. Infection. Stroke. Renal failure. Myocardial infarction.				
Mehr-Aein 2007[135]	• Iran<br>• English<br>• 2007<br>• Single-Centre<br>• 200<br>• Patients undergoing coronary artery bypass	Patients undergoing redo operation, emergency CABG, off-pump CABG, haemoglobin < 10 g/dL, platelet count < 100 K·µ/L, a known coagulopathy disorder, and renal insufficiency.	• IV TXA<br>• No TXA<br>• Cell salvage	-	Blood loss, whole blood transfusions.	Unclear	Not stated	Unclear	Not stated
Menges 1992[136]	• German<br>• German<br>• 1992<br>• Single-Centre<br>• 26<br>• Requires Translation	Requires Translation	• Cell salvage<br>• Control Group<br>• Tranexamic acid	-	Amount of blood re-transfused from the cell saver. Number of patients transfused allogeneic blood.Blood loss. Hb & Hct levels. Clotting status (PT/TT/PTT/ATIII). Immunological methods.	Unclear	Not stated	Unclear	Not stated
Menichetti 1996[137]	• Italy<br>• English<br>• 1996<br>• Single-Centre<br>• 96<br>• Patients who underwent coronary artery bypass surgery	1) emergency operation 2) EF<4% 3) Pre-op Hct <38% 4) Allergy to anti-fibrinolytics 5) thromboembolic disease treated with anticoagulant therapy 6) patients with peripheral vascular disease 7) renal insufficiency (Cr >1.5 mg/dl 8) LFT derangement 9) coagulopathy 10) re-do procedures. 11) Use of acetyl-	• IV TXA<br>• Aprotinin<br>• Epsilon aminocaproic acid<br>• No TXA<br>• Restrictive threshold	-	Postoperative bleeding and need for transfusion showed that the aprotinin group had significantly lower mediastinal bleeding.	Unclear	Not stated	Unclear	Not stated

		salicylic acid or dipyridamole within two week of operation date.							
Mercer 2004[138]	• UK<br>• English<br>• 2004<br>• Single-Centre<br>• 81<br>• Patients undergoing elective repair of infrarenal AAA	Not stated	• Intra Cell Salvage<br>• Control Group<br>• -	incidence of systemic inflammatory response syndrome (SIRS)	requirement for homologous blood transfusion and postoperative infection	Unclear	Not stated	None	Not stated
Miller 1980[139]	• UK<br>• English<br>• 1980<br>• Single-Centre<br>• 100<br>• Patients undergoing<br>• transurethral prostatectomy (92) or endoscopic<br>• bladder tumour resection	Not stated	• PO TXA<br>• No TXA<br>• -	-	Four weeks after operation all patients were reviewed and the severity of haemorrhage and its timing were recorded on standard pro formas. Details of duration of haemorrhage and the association of clots were also noted.	Unclear	Not stated	Unclear	Not stated
Mohib 2015[140]	• Pakistan<br>• English<br>• 2014<br>• Single-Centre<br>• 100<br>• Patient who underwent for intertrochanteric fracture	-	• IV TXA<br>• Placebo<br>• Restrictive threshold	-	Numbers of blood transfusions required postoperatively were noted based on the postoperative haemoglobin readings.	Unclear	Not stated	Unclear	Not stated
Mu 2019[141]	• China<br>• English<br>• 2017<br>• Single-Centre<br>• 150<br>• Patients diagnosed with lumbar degenerative disease and who had no history of posterior lumbar decompression or interbody fusion with pedicle screw fixation	1) history of thromboembolism or evidence of existing thrombus on preoperative vascular B-mode ultrasound; 2) use of antiplatelet aggregation drugs within 6 months or symptom of coagulation dysfunction before surgery; 3) internal diseases such as cardiovascular disease, hepatorenal insufficiency, and hematologic system disease; 4)	• IV TXA<br>• Top TXA<br>• Placebo<br>• -	-	blood biochemical indices, blood loss, and the number of blood transfusions	Unclear	Not stated	Any	Non profit

		confirmed allergy history or high risk of allergy to TXA; 5) history of smoking (more than 10 cigarettes per day for more than 6 months) or drinking (at least 50 g of liquor with an alcohol volume ratio over 40% per day for more than 3 months) with unsuccessful cessation within 6 months before surgery; 6) a body mass index less than 18.5 or over 30.0; and 7) an inability to understand the study protocol after explanation or an unwillingness to participate.							
Murphy 2005[142]	• UK<br>• English<br>• 2005<br>• Single-Centre<br>• 61<br>• Patients aged 18 years or more and who were undergoing nonemergency first-time CABG	Patients who are prevented from receiving blood and blood products according to a system of beliefs (eg, Jehovah Witnesses); patients receiving preoperative warfarin, heparin, or other systemic anticoagulant drugs; patients with congenital or acquired platelet, red blood cell, or clotting disorders; patients with ongoing or recurrent systemic sepsis; and patients who were unable to give full informed consent for the study	• Cell salvage<br>• Control Group<br>• POC testing	-	24-hour postoperative haemoglobin concentration, frequency of homologous blood product use, platelet count, prothrombin time, activated partial thromboplastin time, fibrinogen concentration, D-dimer concentration, and thromboelastography	Unclear	Not stated	Unclear	Not stated
Murphy 2006[143]	• UK<br>• English<br>• 2006<br>• Single-Centre<br>• 100<br>• Patients who underwent off-pump CABG surgery	Advanced chronic renal insufficiency (creatinine >2 mg/dL), active chronic hepatitis or cirrhosis, neurologic dysfunction, hematologic disorders and the use of Clopidogrel pre-operatively.	• IV TXA<br>• No TXA<br>• Cell salvage	-	Homologous packed red cells as blood replacement therapy	Unclear	Not stated	Unclear	Not stated

Nagabhushan 2017[144]	• India<br>• English<br>• 2017<br>• Single-Centre<br>• 50<br>• The patients with American society of Anaesthesiologists (ASA) physical status I and II, aged 18-65 yr, scheduled for elective lumbar spine single level fusion surgery expected to last less than 3 hours, under general anaesthesia were included in the study.	Patients known to have any coagulation disorder, altered liver and renal parameters, and on anticoagulants, antiplatelet medications were excluded from the study.	• IV TXA<br>• Batroxobin<br>• IV TXA + Batroxobin<br>• Placebo<br>• -	-	Intraoperative and postoperative blood loss, haematocrit, allogenic blood transfusion, and deep vein thrombosis (DVT), postoperatively.	Unclear	Not stated	Any	Non profit
Neilipovitz 2001[145]	• Canada<br>• English<br>• 2001<br>• Single-Centre<br>• 40<br>• Patients with scoliosis undergoing posterior spinal fusion surgery	Patients with a history of a bleeding disorder, a low platelet count (,150), abnormal partial thromboplastin time or international ratio test, body mass index .30 kg/m2, previous thromboembolic event, or a family history of thromboembolism	• IV TXA<br>• Placebo<br>• Cell salvage	-	Total amount of blood transfused in the perioperative period, thrombotic complications.	Unclear	Not stated	Any	Industry
Niskanen 2005[146]	• Finland<br>• English<br>• 2003<br>• Single-Centre<br>• 39<br>• Patients with primary cemented hip arthroplasty for osteoarthritis	Patients with rheumatoid arthritis and osteonecrosis, Patients with known coagulation disturbances including thromboembolic events, Patients using warfarin related preparations, or with allergy to tranexamic acid, or with signs of renal insufficiency	• IV TXA<br>• Placebo<br>• -	Blood loss during the operation and the amount of drainage after the operation.	The amount of transfused units of red cells, wound leakage postoperatively, swelling and ecchymoses of the thigh, haematocrit, and possible complications.	Unclear	Not stated	Unclear	Not stated
Nouraei 2013[147]	• Iran<br>• English<br>• 2013<br>• Single-Centre<br>• 80<br>• Patients who underwent CABG surgery	Age of more than 75 years; advanced liver, kidney, lung, or severe peripheral vascular disease; internal carotid artery narrowing of >50%; recent myocardial infarction, New York Heart Association class 3	• Top TXA<br>• Placebo<br>• -	Volume of mediastinal bleeding	Units of transfused packed red cells, FFP, and platelet concentrate	Unclear	Not stated	Any	Non profit

		and 4; CABG with valve operation; insulin-dependent diabetes mellitus; re-exploration; history of seizure disorder; haemoglobin (Hb) levels of <10 g/dL or haematocrit (Hct) levels of <30%; and anticoagulation usage 5 days before surgery.							
Nuttall 2000[148]	• USA<br>• English<br>• 2000<br>• Single-Centre<br>• 160<br>• Cardiac surgery patients at high risk for bleeding	Patients with histories of bleeding or a platelet disorder, prothrombin time (PT). 15.0 s, blood urea nitrogen level greater than 100 mg/dl, or a recent history of thrombolytic, warfarin, or heparin therapy. Patients were excluded if they were taking >325 mg of aspirin a day, had a bleeding time. 8.0 min, or had congenital heart disease; patients with weight less than 45 kg, or if they had a preoperative haemoglobin level <12.5 g/dl.	• IV TXA<br>• Combined<br>• Aprotinin<br>• Placebo<br>• POC tesing	Number of allogeneic blood transfusions in the OR and in the first 24 h in the ICU.	Volume of intraoperative and ICU blood loss over the first 24 h, and duration of time between the end of CPB and OR discharge.	Unclear	Not stated	Unclear	Not stated
Nuttal 2001[149]	• USA<br>• English<br>• 2001<br>• Single-Centre<br>• 92<br>• Adult men and not pregnant adult women with abnormal microvascular bleeding after CPB, all types of elective open cardiac surgery requiring CPB	Patients were not excluded if they received preoperative aspirin or antiplatelet therapy	• TEG+SLT<br>• Control<br>• Tranexamic acid	need for allogenic blood products during the entire stay in hospital	platelet count, TEG variables, PT, aPTT, mediastinal drainage in the ICU, risk of reoperation due to bleeding	Unclear	Not stated	Any	Industry
Oertli 1994[150]	• Switzerland<br>• English<br>• 1994<br>• Single-Centre<br>• 160	Patients with a history of thromboembolic events, severe varicose veins. Coagulation disorders or were receiving anticoagulant drugs.	• PO TXA<br>• Placebo<br>• -	-	-	Unclear	Not stated	Unclear	Not stated

	• Women with breast cancer undergoing lumpectomy								
Orpen 2006[151]	• UK<br>• English<br>• 2006<br>• Single-Centre<br>• 29<br>• Patients due to undergo primary unilateral total knee arthroplasty	Patients with a history of thromboembolic disease, cerebrovascular disease, recent myocardial infarction or unstable angina, a coagulation defect, those with an allergy to TA and those who, not fit to undergo surgery under general anaesthetic.	• IV TXA<br>• Placebo<br>• -	-	On table blood losses, haemoglobin levels.	Unclear	Not stated	Unclear	Not stated
Painter 2018[152]	• Australia<br>• English<br>• 2016<br>• Multi-Centre<br>• 140<br>• Patients undergoing lower limb arthroplasty	Contraindications to the administration of TA including active thromboembolic disease or a history of venous (spontaneous or provoked) or arterial thromboembolic disease	• IV TXA<br>• Placebo<br>• Restrictive threshold	proportion of patients receiving allogenic blood transfusion and the feasibility of extending our trial methodology	change in Hb concentration and PCV, the incidence of adverse clinical events, incidence of surgical complications, length of hospital stay, and the change in a range of quality of life (EQ-5D), quality of recovery (QoR-15), osteoarthritis severity and joint specific questionnaires (Oxford Hip or Knee score).	Unclear	Not stated	None	Not stated
Parrot 1991[153]	• France<br>• English<br>• 1991<br>• Single-Centre<br>• 44<br>• Patients undergoing aortocoronary bypass surgery	Emergency patients, patients with an intra-aortic balloon pump or preoperative haematocrit less than 3S%, and re-operative patients were not included in this study.	• Intra Cell Salvage<br>• Control<br>• -	-	Amount of blood re-transfused from the cell saver. Amount of allogeneic blood transfused. Number of patients transfused allogeneic blood. Complications. Mortality. Blood loss. Hct levels.	Unclear	Not stated	Unclear	Not stated
Pauzenberger 2017[154]	• Austria<br>• English<br>• 2015<br>• Single-Centre<br>• 54	Patient refusal to participate in the study, revision surgery, indication for hemiarthroplasty, known allergy to TXA, anticoagulative	• IV TXA<br>• Placebo<br>• -	Post-operative drain blood loss	Need for post-operative transfusions, and early clinical outcome.	Unclear	Not stated	Unclear	Not stated

	• Patients undergoing unilateral primary stemless anatomical or stemmed reverse total shoulder arthroplasty	medication, severe comorbidities, history of arterial or venous thromboembolic events, coagulopathy, haematological disorders, retinopathy, refusal to receive blood transfusion, pregnancy, or breastfeeding.							
Penta de Peppo 1995[155]	• Italy<br>• English<br>• 1995<br>• Single-Centre<br>• 30<br>• Patients undergoing elective open-heart surgery	Patients with a history of gastrointestinal bleeding	• IV TXA<br>• E-aminocaproic acid<br>• Aprotinin<br>• No Treatment<br>• Cell salvage	-	The amount of blood drained intraoperatively by the Cell Saver system and postoperatively through the chest drains was recorded before reinfusion to the patient, as was the total blood loss both 1 hour and 24 hours after surgery.	Unclear	Not stated	Unclear	Not stated
Pertlicek 2015[156]	• Czech Republic<br>• Czech<br>• 2015<br>• Single-Centre<br>• 119<br>• Patients having primary unilateral total knee arthroplasty	-	• IV TXA<br>• No Treatment<br>• -	-	The intra-operative blood loss, post-operative blood loss based on drainage, pre- and post-operative levels of haemoglobin and haematocrit, and the number of administered blood transfusions	Unclear	Not stated	Unclear	Not stated
Pinosky 1997[157]	• USA<br>• English<br>• 1997<br>• Single-Centre<br>• 39<br>• first-time CABG patients	patient age > 85 years, pregnancy, history of bleeding diathesis, gastrointestinal or upper urinary tract bleeding, or history of allergies to any previous antifibrinolytic therapy.	• IV TXA<br>• EACA<br>• No TXA<br>• Cell salvage	-	The absolute amount of blood loss	Unclear	Not stated	Unclear	Not stated
Pleym 2003	• Norway<br>• English<br>• 2003<br>• Single-Centre<br>• 79	Patients receiving treatment with heparin or low-molecular-weight heparin, oral anticoagulants, nonsteroidal	• IV TXA<br>• Placebo<br>• Cell salvage	-	Transfusions. Preoperative haemoglobin and plasma creatinine levels. Haematocrit,	Unclear	Not stated	Unclear	Not stated

	• Patient undergoing CABG	anti-inflammatory drugs, or other platelet inhibitors.			platelet count, international normalized ratio, activated partial thromboplastin time, fibrinogen, and D-dimer values recorded before surgery and in the morning on the first postoperative day.				
Pourfakhr 2016[158]	• Iran<br>• English<br>• 2016<br>• Single-Centre<br>• 186<br>• Patients who underwent prostatectomy surgery	Patients using anticoagulant drugs such as aspirin and dipyridamole, with high PT (prothrombin time) and PTT (partial thromboplastin time) for any reason, with any history of thrombotic events, with a history of bleeding disorders, with chronic kidney disease (serum creatinine > 180 umol/L), with cardiovascular disease treated with drug eluting stent, with atrial fibrillation, with congenital or acquired thrombophilia, with known or suspected allergy to TRA, and undergoing general or epidural anaesthesia with the acknowledgment of the supervising physician.	• IV TXA<br>• Placebo<br>• -	-	The amount of bleeding and the rate of blood transfusion, the amount of blood inside the blood bags.	Unclear	Not stated	Unclear	Not stated
Prabhu 2015[159]	• India<br>• English<br>• 2015<br>• Single-Centre<br>• 36<br>• Patients underwent total knee arthroplasty	1. Patients aged less than 60 years<br>2. History of haemoglobinopathies /haemophilia/sickle cell disease or with minor or major coagulopathies were all excluded.<br>3. Those on medications on thyroid were excluded.	• PO TXA<br>• Placebo<br>• -	-	The total amount of blood loss	Unclear	Not stated	Unclear	Not stated

		4. Those on immunomodulators and long term steroid intake.							
Pugh 1995[160]	• London<br>• English<br>• 1995<br>• Single-Centre<br>• 45<br>• Patients, age 18 years or over, who were scheduled for routine primary cardiac surgery.	Not stated	• IV TXA<br>• Placebo<br>• Cell salvage	-	The volume of blood loss and blood replacement were measured in the operative and postoperative periods. Haemoglobin concentration, platelet count, and white cell counts were determined preoperatively and at 24 hours postoperatively.	Unclear	Not stated	Unclear	Not stated
Raksakietisak 2015[161]	• Thailand<br>• English<br>• 2013<br>• Single-Centre<br>• 78<br>• Low-risk adult patients undergoing complex laminectomy	Patients with history of thromboembolic diseases	• IV TXA<br>• Placebo<br>• -	Perioperative blood loss occurring intraoperatively and 24 hours postoperatively.	Incidence of blood transfusions.	Unclear	Not stated	Any	Non profit
Rannikko 2004[162]	• Finland<br>• English<br>• 2002<br>• Single-Centre<br>• 136<br>• Men requiring TURP for obstructive urinary symptoms	Patients taking finasteride or with a history of prostate cancer	• PO TXA<br>• Placebo<br>• -	-	-	Unclear	Not stated	Unclear	Not stated
Reid 1997[163]	• USA<br>• English<br>• 1997<br>• Single-Centre<br>• 41<br>• Paediatric patients undergoing repeat cardiac surgery	Children with pre-existing coagulopathy or preoperative anticoagulation	• IV TXA<br>• No TXA<br>• -	-	Total blood loss and transfusion requirements	Unclear	Not stated	Unclear	Not stated

Reyes 2010[164]	• Spain<br>• English<br>• 2010<br>• Single-Centre<br>• 63<br>• Patients undergoing coronary or valve procedure	Combined procedure, aorta procedure, redo surgery, emergency procedures, creatinine levels of 2mg/ml, anaemic patients and patients with body surface area (BSA) 1.6m2	• Cell Salvage<br>• Normal Drainage<br>• Tranexamic acid<br>• Restrictive Threshold	-	Need of blood products and clinical outcomes	Unclear	Not stated	Unclear	Not stated
Rollo 1995[165]	• US<br>• English<br>• 1995<br>• Single-Centre Quasi-randomised by age<br>• 73<br>• Patients undergoing primary uncemented THAs	Patients were excluded from the study if they had a history of a bleeding disorder, infection, carcinoma, or previous surgery involving the operative hip.	• Cell Salvage<br>• Re-infusion<br>• Auto-transfusion<br>• Normal Drainage<br>• -	-	Amount of allogeneic and/or autologous blood transfused. Number of patients transfused allogeneic blood. Complications. Hb & Hct levels. Thigh circumference measures. Wound drainage.	Unclear	Not stated	Unclear	Not stated
Royston 2001[166]	• United Kingdom<br>• English<br>• 2010<br>• Single-Centre<br>• 60<br>• Adult patients (> 21 years), high risk of requiring haemostatic products, cardiac surgery (heart transplantation, revascularization, bypass, Ross procedure, multiple valve or valve and revascularization surgery)	If reoperation due to bleeding was performed or early death of the patient, the data were excluded and replaced by measurements from an additional patient allocated to the same group	• TEG<br>• Control<br>• -	reduced total exposure to haemostatic component therapies	mortality, TEG variables, PT, aPTT, platelet count, fibrinogen concentration, mediastinal tube drainage at 6 and 12 hours	Unclear	Not stated	Unclear	Not stated
Sa-Ngasoongsong 2011[167]	• Thailand<br>• English<br>• 2009<br>• Single-Centre<br>• 48<br>• Patients with primary knee osteoarthritis i) no previous knee surgery; ii) no risk of abnormal bleeding	Patients with incomplete data collection, for example, malfunctioned drain or accidental drain removal.	• IV TXA<br>• Placebo<br>• -	-	Basic postoperative data, such as drain volume, haematocrit (Hct), haemoglobin (Hb), amount of blood transfusion, and WOMAC score, were collected by well-trained research	Unclear	Not stated	Unclear	Not stated

	tendency or bleeding disorder (normal coagulogram, serum creatinine <2.0 mg/dL, stop nonsteroidal anti-inflammatory drugs and antiplatelet drugs more than 7 days; and iii) no contra-indication for TXA use (no active intravascular clotting process, no acquired defective colour vision, no subarachnoid haemorrhage, no hypersensitivity to TXA, and no any of history of serious adverse effects, thrombotic disorder and haematuria)				assistant. Complicated postoperative data requiring clinical examination or physician diagnosis, such as range of motion, and diagnosis of complication, were collected by one of the authors				
Santos 2006[168]	• Brazil<br>• English<br>• 2006<br>• Single-Centre<br>• 60<br>• Patients undergoing CABG	Patients undergoing cardiac surgery reoperation, renal insufficiency (plasma creatinine concentration higher than 2 mg/kg), and a history of haematological disorders, hepatic dysfunction or antiplatelet therapy within seven days of surgery.	• IV TXA<br>• Placebo<br>• -	-	The mass of blood collected via mediastinal and pleural drains for a period beginning with chest closure and lasting 24 h represented blood loss. Other clinical outcomes were also analysed, such as reopening rates, myocardial infarction (new persistent Q-wave and creatine kinase myocardial-band levels more than 30 U/mL), acute renal insufficiency (plasma creatinine concentration higher than 2 mg/ kg), number of RBC transfusions, allergic reactions, convulsive seizures, mortality, and stroke	Unclear	Not stated	Any	Non profit

					(stroke as neurologic complication was defined by hemiparesis, hemiplegia, aphasia, or confusion and disorientation).				
Sarkanovic 2013[169]	• Serbia<br>• English<br>• 2013<br>• Single-Centre<br>• 112<br>• Patients undergoing TKR surgery in a 3-months period during 2010.	patients with<br>septic complications, multiple fractures, malignancy, ASA physical status classification IV or more, hemiarthroplasty and all patients<br>with incomplete data	• Cell Salvage<br>• Normal Drainage<br>• -	-	transfusion of allogeneic blood, length of hospital stay	Unclear	Not stated	Unclear	Not stated
Savvidou 2009[170]	• Greece<br>• English<br>• 2009<br>• Single-Centre<br>• 50<br>• Patients for posterolateral fusion with internal fixation	Not stated	• Post Cell Salvage<br>• Non Cell Salvage Transfusion<br>• Restrictive Threshold	-	surgical time, intraoperative blood loss, haemoglobin and haematocrit levels preoperatively and at discharge were recorded. Intraoperative blood loss was measured by the drain output of the surgical field.	Unclear	Not stated	Unclear	Not stated
Seddighi 2017[171]	• Iran<br>• English<br>• 2011<br>• Single-Centre<br>• 40<br>• Patients aged 20–70 years who were a candidate for major spinal surgeries, good medical condition, and accepted informed consent to attend the study.	Patients aged < 20 and more than 70-year-old who had ischemic heart disease, diabetes, hepatic failure, traumatic vertebral fractures, severe renal failure, active intravascular clotting process, recent thromboembolic events, pregnancy, blurred color vision, coagulopathy, alcoholism and consumption of fluoxetine, contraceptives, insulin, and carbamazepine.	• IV TXA<br>• Placebo<br>• -	-	The patient's characteristics, type and duration of surgery, and the intra and postoperative blood loss were recorded	Unclear	Not stated	Unclear	Not stated
Seo 2013[172]	• Korea<br>• English<br>• 2011	Patients with any cardiovascular problems (such as myocardial infarction	• IV TXA<br>• Placebo<br>• -		The amount of drainage was recorded in order to estimate the blood	Unclear	Not stated	Unclear	Not stated

	• Single-Centre<br>• 150<br>• Patients aged between 55 and 80 years who planned to undergo TKA due to degenerative arthritis on a knee joint.	history, atrial fibrillation, angina), patients with cerebrovascular conditions (such as previous stroke or vascular surgery history), patients with thromboembolic disorders, or those exhibiting a deteriorating general condition.			loss during TKA, and the difference in haemoglobin levels between the preoperative and the postoperative lowest one was also calculated. The frequency of transfusion, the number of blood units transfused, any perioperative complications or events such as infection, deep vein thrombosis (DVT), and pulmonary embolism were also recorded accordingly.				
Sethna 2005[173]	• USA<br>• English<br>• 2005<br>• Single-Centre<br>• 44<br>• Patients scheduled to undergo elective spinal fusion	Patients with (1) pre-existing renal and hepatic disorders; (2) bleeding diathesis and abnormal prothrombin time, partial thromboplastin time (PTT), or platelet counts; and (3) intake of acetylsalicylate within 2 weeks or nonsteroidal anti-inflammatory drugs within 7 days before surgery.	• IV TXA<br>• Placebo<br>• Cell salvage	-	Blood loss, transfusion requirements, coagulation parameters, and complications were assessed	Unclear	Not stated	Unclear	Not stated
Shehata 2012[174]	• Canada<br>• English<br>• 2012<br>• Single-Centre<br>• 50<br>• Eligible participants were adults patients undergoing cardiac surgery with a CARE score (a score for cardiac surgery patients used to predict morbidity and mortality) of 3 or 4 or patients of advanced age	Patients were excluded if they refused participation, were unable to receive or refused blood products, or were involved in the autologous pre-donation program.	• Restrictive 70g/L<br>• Liberal<br>• Tranexamic acid<br>• Cell Salvage	Enrolment rate and overall adherence to the transfusion strategies.	RBC transfusions, clinical outcomes, and physiologic indicators of hypoxemia (mixed venous oxygen saturation). Clinical outcomes were defined as 1) in-hospital all-cause mortality; SHEHATA ET AL. 92 TRANSFUSION Volume 52, January 2012 2) a composite score of morbidity consisting of	Unclear	Not stated	Any	Blood service

	defined as greater than or equal to 80 years on the day of screening were included.<br>• Restrictive threshold 7g/dl				a) neurologic events defined as a new focal neurologic deficit lasting more than 24 hours or irreversible encephalopathy, b) dialysis-dependent renal failure or greater than 50% increase in creatinine, c) prolonged low cardiac output state (i.e., need for two or more inotropes for 24 hours or more, intraaortic balloon pump or ventricular assist device for greater than 48 h), and/or myocardial infarction, defined as troponin I level greater than 2.5 mg/L and new Q waves on electrocardiogram or a clinical diagnosis; and 3) hospital lengths of stay				
Shenolikar 1997[175]	• UK<br>• English<br>• 1997<br>• Single-Centre<br>• 100<br>• patients with a preoperative haemoglobin>11 g /dL, scheduled for knee replacement surgery	Not stated	• Post Cell Salvage<br>• Control<br>• -	-	Amount of blood collected by the cell saver. Amount of allogeneic blood transfused. Number of patients transfused allogeneic blood. Complications. Hospital length of stay.	Unclear	Not stated	Unclear	Not stated
Shimizu 2011[176]	• Japan<br>• English<br>• 2007<br>• Single-Centre<br>• 160	Neonates of less than 1 month of age, children on mechanical ventilation preoperatively, and children on inotropic support before surgery were excluded	• IV TXA<br>• Placebo<br>• -	24-h blood loss.	re-exploration of the chest for bleeding, transfusions of blood products requirement, Mechanical ventilation	Unclear	Not stated	Unclear	Not stated

	• Children younger than 18 years of age who were scheduled to undergo elective cardiac surgery with CPB	from the study. Other exclusion criteria included a pre-existing coagulation disorder, re-operation within 48 h, obvious kidney or liver disease, and known allergy to TXA			in the ICU, length of stay, and complications.				
Shore-Lesserson 1996[177]	• USA<br>• English<br>• 1996<br>• Single-Centre<br>• 30<br>• Adult patients undergoing repeat open heart surgery	Patients were excluded if they had preoperative coagulopathy that included thrombocytopenia (Platelet count <100,000/mm^3), uremic thrombocytopathy (patients receiving preoperative dialysis), and inherited or acquired coagulopathy (von Willebrand disease, haemophilia A, residual Warfarin effect, etc.). Also excluded were patients receiving inotropic therapy or intra-aortic balloon counter-pulsation, and patients who refused blood transfusion for religious reasons.	• IV TXA<br>• Placebo<br>• POC testing<br>• Cell salvage	-	Routine coagulation tests, D-dimer levels, mediastinal tube drainage, and transfusion requirements were compared	Unclear	Not stated	Unclear	Not stated
Shore-Lesserson 1999[178]	• USA<br>• English<br>• 1999<br>• Single-Centre<br>• 105<br>• Adult cardiac surgical patients at moderate to high risk of microvascular bleeding and thus had a moderate to high risk for requiring a transfusion. Included patients underwent single valve replacement, multiple valve replacement, combined coronary artery bypass plus valvular	Significant pre-existing hepatic disease (transaminase levels > 2 times control) or renal disease requiring dialysis, or if they required preoperative inotropic support	• TEG<br>• Control<br>• -	reduction in transfusion requirements	Coagulation tests, TEG variables, postoperative blood loss into mediastinal drainage at 6-hour intervals for 2 days postoperatively, platelet count, PT, aPTT, fibrinogen level, TEG variables	Unclear	Not stated	Unclear	Not stated

	procedure, cardiac reoperation, or thoracic aortic replacement. Patients receiving preoperative heparin infusion and those who had taken aspirin within the past 7 days were included								
Spark 1997[179]	• UK<br>• English<br>• 1997<br>• Single-Centre<br>• 50<br>• Patients undergoing elective infrarenal abdominal aortic aneurysm repair.	-	• Intra Cell Salvage<br>• Control<br>• -	-	Amount of allogeneic blood transfused. Number of patients transfused allogeneic blood. Complications. Hospital length of stay. Blood loss. Mortality.	Unclear	Not stated	None	Not stated
Speekenbrink 1995[180]	• Netherlands<br>• English<br>• 1995<br>• Single-Centre<br>• 60<br>• Patients undergoing CABG (with a preoperative platelet count of less than 246 x 10(9)/L)	Patients with a body weight of more than 100 kg. Patients with already impaired renal function (creatinine level more than 200 µmol/L) were not included. Also patients with intravenous heparin treatment or a history of coagulopathy were excluded.	• IV TXA<br>• Dipyridamole<br>• Aprotinin<br>• Placebo<br>• -	-	Intraoperative haemoglobin loss. The volume of mediastinally shed blood was measured 6 and 24 hours after the operation. Intraoperative and postoperative transfusions of homologous blood products were recorded.	Unclear	Not stated	Unclear	Not stated
Stowers 2017[181]	• New Zealand<br>• English<br>• 2017<br>• Multi-Centre<br>• 134<br>• Patients older than 18 years undergoing primary unilateral TKA	History or risk of thrombosis, active thromboembolic disease, refused blood products, known hypersensitivity to TXA or any of its ingredients, complex hematologic disorders requiring manipulation, pregnant and lactating women, taking anticoagulant therapy within 5 days of surgery	• IV TXA<br>• IA TXA<br>• Placebo<br>• -	estimated blood loss (EBL) as calculated from the difference from preoperative haemoglobin (Hb) and final Hb before discharge or day 3 at the latest.	Functional measurements using patient self-reported questionnaires (Short-Form 12 survey and Oxford knee scores) were performed preoperatively and at 6 weeks after surgery. Transfusion rates, median length of stay,	Unclear	Not stated	None	Not stated

		(warfarin, dabigatran, heparin, rivaroxaban), or had severe renal failure (estimated glomerular filtration rate <29)			and 30-day readmissions and complications were also measured. Important complications captured included symptomatic deep vein thrombosis (DVT), pulmonary embolism (PE), and infection. ROM, both passive and active, was measured as a surrogate for postoperative swelling.				
Taghaddomi 2009b[182]	• Iran<br>• English<br>• 2009<br>• Single-Centre<br>• 100<br>• Patients undergoing off-pump coronary artery bypass surgery	Patients with a history of bleeding disorders, active chronic hepatitis or cirrhosis, chronic renal insufficiency (serum creatinine >2 mg/dL), preoperative anaemia (Hb < 11 g/dL), previous cardiac surgery, and myocardial infarction >7 days before surgery. Also, patients receiving potent antiplatelet agents like adenosine diphosphate inhibitors (Ticlopidine and Clopidogrel) but not aspirin were excluded	• IV TXA<br>• No TXA<br>• -	-	Hematologic parameters, volume of blood loss, blood transfusion, and other clinical data were recorded throughout the perioperative period.	Unclear	Not stated	Unclear	Not stated
Tanaka 2001[183]	• Japan<br>• English<br>• 2001<br>• Single-Centre<br>• 99<br>• Patients who were undergoing total knee arthroplasty	Known allergy to TNA, preoperative hepatic or renal dysfunction, serious cardiac or respiratory disease, congenital or acquired coagulopathy, and a history of thromboembolic disease.	• IV TXA<br>• Pre-op TXA<br>• Post-op TXA<br>• No TXA<br>• -	-	The need for blood transfusion and apparent blood loss. Thromboembolic and other complications were noted during the hospital stay.	Unclear	Not stated	None	Not stated
Tempe 1996[184]	• India<br>• English<br>• 1996<br>• Single-Centre	Patients having a re-operation or preoperative coagulation abnormalities were excluded	• Intra+Post Cell Salvage<br>• Control<br>• Iron therapy	-	Amount of allogeneic blood transfused. Number of patients transfused allogeneic	Unclear	Not stated	Unclear	Not stated

	• 100<br>• Patients undergoing elective valve surgery, using cardiopulmonary bypass (CPB)				blood. Complications. Re-exploration for bleeding. Chest drainage. Hct levels.				
Tempe 2001[185]	• India<br>• English<br>• 2001<br>• Single-Centre<br>• 40<br>• Patients scheduled for elective primary valve surgery	-	• Cell Salvage<br>• Control<br>• Iron therapy	-	Amount of allogeneic blood transfused. Re-exploration for bleeding.	Unclear	Not stated	Unclear	Not stated
Tengberg 2016[186]	• Denmark<br>• English<br>• 2016<br>• Single-Centre<br>• 72<br>• Patients undergoing surgery for extra-capsular hip fractures	Allergy to tranexamic acid, ongoing thromboembolic event (deep venous thrombosis (DVT), pulmonary embolism (PE), arterial thrombosis or cerebral thrombosis), reduced kidney function (defined as a serum creatinine > 120 umol/L), anticoagulation therapy including vitamin K-antagonists, direct thrombin inhibitors, direct factor X-a inhibitors and platelet aggregation inhibitors (not including acetylsalicylic acid), disseminated intravascular coagulation (DIC), bleeding in the upper urinary tract (risk of obstruction), patients with a history of cramps, subarachnoid bleeding, malignancy, pathological fracture, previous operation on the affected hip, more than one current fracture, or bodyweight in excess of 100 kg.	• IV TXA<br>• IV TXA<br>• Placebo<br>• -	Total blood loss (TBL)	number of transfusions, risk reduction for receiving at least one transfusion and surgical blood loss during the operative procedure.	Unclear	Not stated	None	Not stated
Thomas 2001[187]	• UK<br>• English	Not stated	• Post Cell Salvage<br>• Control	-	Number of patients transfused allogeneic	Unclear	Not stated	None	Not stated

	• 2001<br>• Single-Centre<br>• 231<br>• Patients undergoing TKR		• -		blood. Amount of allogeneic blood transfused. Complications.				
Thomassen 2012[188]	• Netherlands<br>• English<br>• 2012<br>• Multi-Centre<br>• 216<br>• Patients receiving primary or revision total hip arthroplasty with ASA I, II, or II	-Exclusion due to ethical concern included previous randomization in this study, involvement in the planning and/or conduct of this study, and participation in an interfering study.<br>– Exclusion due to safety concerns included current symptoms of haemophilia and contraindications for autologous blood use, i.e. hyperkalaemia, current systemic infection or local infection in the operation field or impaired renal function, known malignancy in the last five years and expected use of cytotoxic drugs.<br>– Exclusion due to expected impact on outcome included untreated anaemia (haemoglobin (Hb) level <11 g/dL), revision total hip arthroplasties with expected serious bone grafting, and use of other alternatives for blood conservation such as recombinant erythropoietin, fibrin sealant, Aprotinin and other autologous blood transfusion.	• Post Cell Salvage<br>• Control<br>• Tranexamic acid	allogeneic blood transfusion frequency	blood loss, postoperative haemoglobin/haematocrit, safety and quality of life Perioperative blood loss	Unclear	Not stated	Any	Industry
Tsutsumimoto 2011[189]	• Japan<br>• English<br>• 2011<br>• Single-Centre<br>• 40	Patients with chronic renal failure, cirrhosis of the liver, serious cardiac disease, allergy to TXA, a history of thromboembolic disease, bleeding disorders, hyper-	• IV TXA<br>• Placebo<br>• -	-	Intra- and postoperative blood loss	Unclear	Not stated	None	Not stated

	• Patients undergoing total hip and knee arthroplasty.	coagulation status, disseminated intravascular coagulation, and those who were receiving antiplatelet and/or anticoagulant drugs.							
Ugurlu 2017[190]	• Turkey<br>• English<br>• 2015<br>• Single-Centre<br>• 123<br>• Patients undergoing primary unilateral total knee arthroplasty	Flexion deformity of > 30 degrees, varus/valgus > 30 degrees, preoperative use of anticoagulants (acetylsalicylic acid, enoxaparin, warfarin, or any other oral or IV agent), abnormalities in coagulation screening tests, history of DVT or pulmonary embolism, transient ischemic attack, stroke, renal (serum creatinine > 2 standard deviation [SD] for age) or hepatic insufficiency, and pregnancy	• IV TXA<br>• Top TXA<br>• No TXA<br>• Restrictive threshold	-	The haemoglobin values were recorded preoperatively and postoperatively on the same day and on day 1 and day 2. Removal of the drain postoperatively and length of hospital stay, as well as any complications such as pulmonary embolism or deep venous thrombosis, were also noted.	Unclear	Not stated	Unclear	Not stated
Uozaki 2001[191]	• Japan<br>• English<br>• 2001<br>• Single-Centre<br>• 14<br>• Patients undergoing elective cardiopulmonary bypass for coronary artery bypass surgery.	Not stated	• IV TXA<br>• Placebo<br>• -	-	Intraoperative and postoperative blood loss	Unclear	Not stated	Unclear	Not stated
Vanek 2005[192]	• Czech Republic<br>• English<br>• 2004<br>• Single-Centre<br>• 91<br>• Patients undergoing OPCAB	Not stated	• IV TXA<br>• Aprotinin<br>• Placebo<br>• -	30-day mortality	ICU LOS<br>Hospital LOS<br>Risk of RBC transfusion<br>Perioperative blood loss<br>Reoperation for bleeding	Unclear	Not stated	Any	Non profit
Veien 2002[193]	• Denmark<br>• English<br>• 2002<br>• Single-Centre<br>• 30	Patients with age less than 18 years, recent myocardial infarction (<6months), unstable angina, severe aortic or mitral valve stenosis, previous stroke,	• IV TXA<br>• Placebo<br>• Cell salvage	-	Blood loss	Unclear	Not stated	Unclear	Not stated

	• Patients scheduled for TKR in spinal anaesthesia with the use of a tourniquet,	unmedicated hypertension, history of thromboembolic episodes, bleeding disorders or warfarin medication.							
Vermeijden 2015[194]	• Netherlands<br>• English<br>• 2015<br>• Multi-Centre<br>• 366<br>• Patients undergoing elective coronary, valve, or combined surgical procedures	Patients scheduled for off-pump surgery and patients with known coagulation disorders except after the use of aspirin, Clopidogrel, or low molecular-weight heparin	• Cell Salvage<br>• Normal Drainage<br>• Tranexamic acid<br>• Restrictive threshold	the number of allogeneic blood products transfused in each group during hospital admission.	percentage of patients who received any allogeneic blood products, number of re-explorations, myocardial infarction, stroke, postoperative ventilation time, length of stay in the intensive care unit and in the hospital, and 1-year mortality.	Unclear	Not stated	None	Not stated
Virani 2016[195]	• India<br>• English<br>• 2015<br>• Single-Centre<br>• 137<br>• Patients above 65 years of age, underwent peritrochanteric fracture surgery	Patients with low preoperative platelet counts, bleeding disorders and coagulopathies, patients with severe hepato-renal dysfunction and cardiopulmonary disease, and those on aspirin or NSAIDS in the week preceding surgery	• IV TXA<br>• No TXA<br>• -	-	The postoperative drain output was recorded, as well as the haemoglobin level and the patients needing blood transfusion.	Unclear	Not stated	Unclear	Not stated
Wang 2010[196]	• Taiwan<br>• English<br>• 2010<br>• Single-Centre<br>• 28<br>• Adult patients undergoing orthotopic liver transplantation	None stated	• TEG<br>• Control<br>• Restrictive threshold	-	3 years mortality, transfusion requirements, total amount of IV fluids (fluid total, hydroxyethyl starch, albumin), blood loss, urine output	Unclear	Not stated	Any	Non profit
Weber 2012[197]	• Germany<br>• English<br>• 2010<br>• Single-Centre<br>• 100<br>• Patients were suitable for this trial after two inclusion steps Step 1: Patients (>=	Pregnancy	• ROTEM + PLT MAPPING<br>• Control<br>• Tranexamic acid<br>• Restrictive Threshold<br>• Cell Salvage	the number of transfused units of packed erythrocytes during the period between inclusion into the study and 24	•The number of transfused units of FFP, platelet concentrates and any other administered haemostatic therapy during the period between inclusion into	Unclear	Not stated	Unclear	Not stated

	18 years) scheduled for elective, complex cardiothoracic surgery (combined CABG and valve surgery, double or triple valve procedures, aortic surgery or redo surgery) with CPB were re-operatively screened for eligibility, and written consent was obtained Step 2: Patients were enrolled in the study after heparin reversal following CPB if at least one of the two inclusion criteria were fulfilled: (1) diffuse bleeding from capillary beds at wound surfaces requiring haemostatic therapy as assessed by the anaesthesiologist and surgeon by inspecting the operative field and/or (2) intraoperative or postoperative (during the first 24 postoperative hours) blood loss exceeding 250 mL/hour or 50 mL/10 min			hours after ICU admission	the study and 24 hours after ICU admission<br>• Volume of intraoperatively and up to 24 hours postoperatively re-transfused salvaged washed erythrocytes<br>• Postoperative chest tube blood loss 6, 12, and 24 hours after ICU admission<br>• Lowest haemoglobin concentration between inclusion into the study and 24 hours after ICU admission<br>• Number of re-thoracotomies during the first 24 postoperative hours<br>• PaO2/FiO2 indices at 2, 4, 12, and 24 hours after ICU admission<br>• Postoperative time of mechanical ventilation<br>• Length of ICU stay and hospital stay<br>• Incidence of acute renal failure, sepsis, thromboembolism, and allergic complications<br>• Mortality during a 6-month follow-up<br>• Costs of haemostatic therapy as prescribed by local pharmacy and blood bank				
Wei 2006[198]	• China<br>• English<br>• 2006<br>• Single-Centre	Patients with valve diseases, myocardial infarction less than four weeks before surgery, left ventricular ejection fraction	• IV TXA<br>• Placebo<br>• -	-	Hematochemical parameters including platelet adhesion rate, Ddimer and	Unclear	Not stated	Any	Non profit

	• 76<br>• Patients undergoing elective OPCAB	lower than 40%, neurologic or pulmonary disorders, renal and liver failure were not eligible.			fibrinopeptide-A (FPA) were analysis. Volume of blood loss, blood transfusion and other clinical data were recorded throughout the perioperative period.				
Westbrook 2009[199]	• Australia<br>• English<br>• 2009<br>• Single-Centre<br>• 69<br>• All patients presenting for cardiac surgery with the exception of lung transplantation	None stated	• TEG + PLT MAPPING<br>• Control<br>• Tranexamic acid	-	Blood loss, intubation time (hours), minimum Hb (g/L), ICU stay, hospital stay (days)	Unclear	Not stated	Any	Industry
Wong 2008[200]	• Canada<br>• English<br>• 2008<br>• Single-Centre<br>• 147<br>• Patients having spinal fusion surgery	Patients with a history of allergy to TXA, acquired disturbances of colour vision, spine tumour, intra-dural pathology, ankylosing spondylitis, preoperative anaemia, i.e., haemoglobin <11 g/dL in females; haemoglobin <12 g/dL in males, refusal of blood products i.e., Jehovah's witnesses, coagulopathy, preoperative anticoagulant therapy, fibrinolytic disorders requiring intraoperative antifibrinolytic treatment, preoperative platelet count <150,000/mm3, International Normalized Ratio (INR) >1.4, prolonged partial thromboplastin time (PTT) (>1.4 x normal), a history of thromboembolic disease, pregnancy, significant co-	• IV TXA<br>• Placebo<br>• Cell salvage	The total perioperative estimated and calculated blood loss intraoperatively and 24 h postoperatively.	Incidence of allogeneic blood exposure, and duration of hospital stay.	Unclear	Not stated	Unclear	Not stated

		morbidities i.e., severe ischemic heart disease New York Heart Association Class III–IV, previous myocardial infarct (MI), severe pulmonary disease, i.e., forced expiratory volume in 1 min <50% normal, chronic renal failure, hepatic failure. If intraoperative surgical complications such as uncontrollable surgical bleeding from broken vertebral laminae, or dural tears, etc. occurred, the patients were excluded from the study.							
Wu 2006[201]	• Taiwan<br>• English<br>• 2004<br>• Single-Centre<br>• 214<br>• Patients undergoing liver resections for various liver tumours	Patients who underwent emergency surgery for a ruptured liver tumour or patients whose liver tumours were resected under cardiopulmonary bypass	• IV TXA<br>• Placebo<br>• Restrictive threshold	-	The patients' background, blood transfusion rates, and early postoperative results in the 2 groups were compared.	Unclear	Not stated	Any	Non profit
Xu 2012[202]	• China<br>• English<br>• 2012<br>• Single-Centre<br>• 80<br>• Patients undergoing scheduled idiopathic scoliosis surgery	Pre-existing cardiac, pulmonary, renal and hepatic disorders; intake of NSAIDs within 7 days before surgery; history of coagulation disorders, Deep vein thrombosis (DVT) or pulmonary embolisms; lower preoperative Hb (\100 g/l); abnormal clotting tests, such as prothrombin time (PT) and platelet counts.	• Placebo<br>• Batroxobin<br>• IV TXA<br>• IV TXA+Batroxibin<br>• Placebo<br>• -	-	The amounts of blood loss, transfusion requirements, frozen fresh plasma (FFP) and overall drainage were assessed. The hemoglobin concentration (Hb), hematocrit and platelet counts were recorded preoperative y, postoperatively and on the first operative day. The coagulation parameters were measured meanwhile.	Unclear	Not stated	Unclear	Not stated

					Deep vein thrombosis (DVT) was diagnosed by ultrasound.				
Xu 2015[203]	• China<br>• English<br>• 2014<br>• Single-Centre<br>• 224<br>• Patients were adults who received primary unilateral THA regardless of the type or size of prosthesis implanted; the intervention was topical (intra-articular) administration of TXA; the full text of each article was available; (iv) outcome measures included total blood loss, transfusion rate, and incidence of thromboembolic complications	Patients who had allergy to tranexamic acid; thrombotic disorder; patients who were on anticoagulant treatment.	• Top TXA<br>• Placebo<br>• Restrictive threshold	The rate of deep vein thrombosis (DVT) and pulmonary embolism (PE), transfusion rate, difference between the preoperative haemoglobin and the lowest postoperative haemoglobin during the hospital stay.	Total volume of drainage, intraoperative blood loss, total blood loss and other perioperative complications.	Unclear	Not stated	Unclear	Not stated
Xu 2019[204]	• China<br>• English<br>• 2018<br>• Single-Centre<br>• 150<br>• patients aged 20 to 70 years and elective cardiac valvular surgery under extracorporeal circulation, without preoperative anaemia and blood transfusion.	(1) history of iron allergy;<br>(2) determined iron overload or hereditary iron utilization disorder;<br>(3) severe hepatic insufficiency (alanine aminotransferase >3 times normal upper value).	• IV Fe<br>• Placebo<br>• Restrictive threshold	changes in Hb concentration on POD 7 and POD 14 between the 2 groups	changes in HCT, RBC count, serum ferritin and transferrin saturation, the length of ventilation,<br>ICU stay and postoperative hospital stay, and occurrence of adverse events during admission between the 2 groups	Unclear	Not stated	None	Not stated
Yassen 1993[205]	• UK<br>• English<br>• 1993<br>• Single-Centre<br>• 20	No stated	• IV TXA<br>• No TXA<br>• Cell salvage	-	Transfusion and blood loss	Unclear	Not stated	Unclear	Not stated

	• Patients undergoing orthoptic liver transplantation								
Zabeeda 2002[206]	• Israel<br>• English<br>• 2002<br>• Single-Centre<br>• 50<br>• Patients scheduled for elective or urgent CABG.	Patients with an ejection fraction less than 40%, impaired kidney function (creatinine > 2 mg/dL), a history of abnormal bleeding, or an abnormal coagulation profile. Patients receiving bilateral mammary artery grafts were excluded from the study.	• IV TXA<br>• Placebo<br>• Restrictive threshold	-	Blood loss, transfusion, reoperation, fibrinogen level, fibrinogen split products, platelet size, and platelet function.	Unclear	Not stated	Unclear	Not stated
Zhao 2017[207]	• China<br>• English<br>• 2017<br>• Single-Centre<br>• 120<br>• Patients undergoing off-pump coronary artery bypass operations.	-	• Cell Salvage<br>• Non Cell Salvage Transfusion<br>• -	-	all adverse reactions, such as haemoglobin urine, allergic reactions, and coagulation abnormalities, autologous blood transfusion volume and allogeneic blood transfusion volume were also recorded. One day after the operation, routine blood tests and biochemistry were performed; ICU retention time and complications were recorded.	Unclear	Not stated	Unclear	Not stated
Zhao 2018[208]	• China<br>• English<br>• 2017<br>• Single-Centre<br>• 120<br>• Patients undergoing primary THA	Patients with a body weight index (BMI) > 30 kg/m2; Crowe type 3 or 4 dysplasia; previous hardware; prior hip surgery; and an inability to tolerate general anaesthesia. Patients meeting the above inclusions are being operated via the direct anterior approach for	• IV TXA<br>• PO TXA<br>• Placebo<br>• -	Haemoglobin drop, haematocrit levels, total blood loss, intra-operative blood loss, need for transfusion, and volume transfused.	Thromboembolic events, wound complications, the length of post-operative hospital stay, and 30-day readmission.	Unclear	Not stated	None	Not stated

		THA. In addition, patients were excluded if they had bilateral arthroplasty, allergy to TXA, or history of renal failure, kidney transplant, a recent arterial thromboembolic event such as myocardial infarction or stroke, hyper-coagulation, haemophilia, deep vein thrombosis, or pulmonary embolism. Patients were also excluded if they declined to participate or to receive blood products.							
Zohar 2004[209]	• Israel<br>• English<br>• 2004<br>• Single-Centre<br>• 40<br>• Patients undergoing elective total knee replacement	Patients with a history of severe ischemic heart disease (New York Heart Association Class III and IV), chronic renal failure, cirrhosis, bleeding disorders, or current anticoagulant therapy	• IV TXA<br>• Placebo<br>• -	-	-	Unclear	Not stated	Unclear	Not stated
Zufferey 2010[210]	• France<br>• English<br>• 2010<br>• Single-Centre<br>• 110<br>• Patients requiring surgery for an isolated hip fracture of less than 48 h	Pregnancy or breast-feeding, contraindication for tranexamic acid (previous arterial or venous thrombosis, creatinine clearance < 30 ml/min, previous seizure or Oestroprogestative therapy), multiple fractures, contraindication for prophylaxis with Fondaparinux (Arixtra, GlaxoSmithKline, Brentford, UK), and requirement for anticoagulant therapy that could not be stopped.	• IV TXA<br>• Placebo<br>• -	Incidence of patients requiring the transfusion of at least 1 U of allogeneic RBC from surgery up to day 8.	postoperative bacterial infection, which was defined as the composite of pneumonia, other lower respiratory tract infection, blood stream infection, urinary tract infection, superficial wound infection, deep wound infection, and osteomyelitis or septic arthritis up to 6 weeks.	Unclear	Not stated	Any	Non profit
Slagis 1991[211]	• USA<br>• English<br>• 1991<br>• Single-Centre	Patients who needed transfusion pre-operatively and those who had refused to participate.	• Intra+Post Cell Salvage<br>• Normal Drainage	-	Amount of blood collected by the cell saver. Amount of blood re-transfused from the	None	Blood service	None	Not stated

	• 102<br>• Patients undergoing hip or knee arthroplasty at the University of Arizona Medical Centre between August 1, 1988 and June 1, 1989.		• -		cell saver. Amount of allogeneic blood transfused. Number of patients transfused allogeneic blood. Complications. Coagulopathy. Blood loss. Transfusion reactions.				
Aguilera 2015[212]	• Spain<br>• English<br>• 2015<br>• Multi-Centre<br>• 100<br>• Adult patients undergoing primary total knee arthroplasty	known allergy to TXA, a history of coagulopathy or a thromboembolic event, previous bypass surgery, use of anticoagulant or contraceptive treatment, cardiovascular prosthesis, and refusal to participate	• IV TXA<br>• No TXA<br>• -	total blood loss	Hidden blood loss, blood collected in drains, transfusion rate, number of blood units transfused, adverse events, and mortality.	None	Not stated	Any	Industry
Ak 2009[213]	• Turkey<br>• English<br>• 2009<br>• Single-Centre<br>• 224<br>• Adult patients undergoing elective first time CABG with cardiopulmonary bypass	Preoperative haemodynamic instability, malignancies, history of bleeding diathesis, use of low molecular weight heparin until the day of operation, recent treatment (<5days) with a glycoprotein IIb/IIIa antagonist or Clopidogrel, impaired renal function (creatinine>2mg/dL) and liver disease resulting in elevated liver function tests	• TEG<br>• Standard of care<br>• Tranexamic Acid	incidence of blood transfusion, blood loss	amount of blood and blood products consumed perioperatively, blood loss<br>mediastinal chest tube drainage, need for additional protamine, need of tranexamic acid infusion, mortality, risk of surgical cause of reoperation for bleeding and clinical complications<br>outcome after CABG (superficial soft tissue infection, major respiratory complications, postoperative renal dysfunction) and haematological variables (haematocrit and platelets)	None	Not stated	None	Not stated

Alizadeh 2014[214]	• Iran<br>• English<br>• 2014<br>• Single-Centre<br>• 200<br>• Patients undergoing elective coronary artery revascularisation	Patients with a serum creatinine level of >2 mg/dl, previous history of bleeding or coagulation disorders, taking oral anticoagulation medications within 72 hours of the surgery and allergy to the study medications	• IV TXA<br>• Placebo<br>• -	The total volume of mediastinal bleeding during the first 24 hours after surgery	MI<br>Adverse Reaction<br>AKI<br>Acute brain injury<br>Sepsis<br>Risk & number of RBC transfusion<br>Perioperative blood loss<br>Risk of receiving non red cell component	None	Not stated	Unclear	Not stated
Apipan 2017[215]	• Thailand<br>• English<br>• 2017<br>• Single-Centre<br>• 40<br>• Patients scheduled for elective bi-maxillary osteotomy	Patients with a known allergy to the study drug, a history or a risk of thromboembolism (including taking oral contraceptive pills), or a body mass index (BMI) more than 30 kg/m2	• IV TXA (20mg/kg)<br>• IV TXA (15mg/kg)<br>• IV TXA (10mg/kg)<br>• Placebo<br>• -	Intraoperative blood loss and the number of patients receiving a transfusion of allogeneic blood products.	Difference between preoperative and 24-h postoperative haematocrit, the volume of 24-h postoperative vacuum drainage, and the length of hospital stay.	None	Not stated	None	Not stated
Arantes 2016[216]	• Brazil<br>• English<br>• 2016<br>• Single-Centre<br>• 70<br>• Patients who underwent primary palatoplasty with no known or suspected coagulation disorders	Patients with a platelet count lower than 100,000/mm3, with known or suspected coagulation disorders, family history of coagulopathy, or indication of secondary palatoplasty for the correction of oronasal fistula	• IV TXA<br>• Placebo<br>• -	-	The occurrence of significant haemorrhagic events, defined as the need to use blood products, the need to redo surgery, or the need to use antifibrinolytic drugs during the postoperative period to control excessive bleeding,	None	Not stated	None	Non profit
Ausen 2015[217]	• Norway<br>• English<br>• 2015<br>• Single-Centre<br>• 30<br>• Consecutive women undergoing bilateral reduction mammoplasty	A history of any thromboembolic disease, pregnancy or severe co-morbidity (American Society of Anaesthesiologists (ASA) fitness grade III or IV)	• IV TXA<br>• Placebo<br>• -	Drain fluid production in the first 24 h after surgery.	Postoperative pain, which was registered for each breast both 3 and 24 h after surgery, using a visual analogue scale from 0 (no pain) to 10 (unbearable).	None	Not stated	Unclear	Not stated

Bansal 2017[218]	• India<br>• English<br>• 2017<br>• Single-Centre<br>• 400<br>• Patients who were planned for percutaneous nephrolithotomy	Patients having hypersensitivity to tranexamic acid, defective colour vision, anticoagulant usage, subarachnoid haemorrhage, abnormal liver function test, unstable cardiovascular disease, acute or chronic renal failure or any haematological disease	• IV TXA<br>• Placebo<br>• -	fall in hemoglobin/hematocrit level and total blood loss.	Overall complications rate of PCNL	None	Not stated	None	Not stated
Baradaranfar 2017	• Iran<br>• English<br>• 2017<br>• Single-Centre<br>• 60<br>• Patients with chronic rhinosinusitis with polyposis	Patients with previous sinus or nasal surgery, underlying disease with increased risk of thromboses (hypercoagulable states) such as Factor V Leiden, antiphospholipid syndrome, heparin-induced thrombocytopenia, cancer, pregnancy, high blood pressure (systolic >140 mmHg and/or diastolic >90 mmHg), contraindications for the use of tranexamic acid (active clot inside arteries), and patient unwillingness or participation in other similar clinical trials.	• Top TXA<br>• Placebo<br>• -	-	-	None	Not stated	Unclear	Not stated
Barrachina 2016[220]	• Spain<br>• English<br>• 2016<br>• Multi-Centre<br>• 78<br>• ASA physical status I to III patients undergoing unilateral total hip replacement surgery	pregnancy or breastfeeding, severe vascular ischemia, history of venous thrombosis, pulmonary embolism or diseases causing embolism, known coagulopathies, long-term treatment with acetylsalicylic acid or nonsteroidal anti-inflammatory drugs not discontinued before surgery, a haemoglobin (Hb) concentration <10 mg/dL, moderate renal impairment, liver cirrhosis, or any	• IV TXA<br>• Placebo<br>• Cell salvage	total blood loss up to day 2 after surgery	Blood loss up to 1 and 6 hours after the start of surgery.	None	Not stated	None	Not stated

		contraindications to prophylaxis with enoxaparin.							
Baruah 2016[221]	• India<br>• English<br>• 2016<br>• Single-Centre<br>• 60<br>• Patients who underwent open reduction and internal fixation with a dynamic hip screw plate for stable trochanteric fracture	Patients who had (1) a fracture unsuitable for dynamic hip screw plate fixation, (2) an allergy to TXA, (3) preoperative renal impairment (serum creatinine >2 mg% or creatinine clearance <30 ml/min), (4) preoperative hepatic impairment (international normalised ratio [INR] for prothrombin time >1.5 or liver enzymes elevated by >3 times the normal range, (5) known bleeding disorder or preoperative coagulation anomaly determined by prolonged bleeding time and clotting time, an INR >1.5, or a prolonged partial thromboplastin time, (6) a history of any thrombo-embolic events (such ascerebrovascular accident, acute coronary syndrome/ myocardial infarction, pulmonary embolism, deep vein thrombosis, or arterial thrombosis), (7) anticoagulants or aspirin-like drugs, oestroprogestive drugs, or long-acting non-steroidal anti-inflammatory drugs, or (8) were pregnant or breastfeeding.	• IV TXA<br>• Placebo<br>• -	-	-	None	Not stated	Unclear	Not stated
Benoni 1996[222]	• Sweden<br>• English<br>• 1996<br>• Single-Centre<br>• 86	-	• IV TXA<br>• Placebo<br>• -	-	-	None	Not stated	none	Non profit

	• Patients with knee arthroplasty								
Benoni G 2000[223]	• Sweden<br>• English<br>• 2000<br>• Single-Centre<br>• 40<br>• Primary total hip replacement operations	Not stated	• IV TXA<br>• Placebo<br>• -	-	-	None	Not stated	any	Industry
Bernabeu Wittel 2016[224]	• Spain<br>• English<br>• 2016<br>• Multi-Centre<br>• 303<br>• Patients >65years admitted with hip fracture and Hb level 90-120 g/L	Marrow diseases that could interfere in the erythropoietic process, blood coagulation diseases or current treatment with anticoagulants, documented allergy or intolerance and/or contraindication to EPO use and/or IV iron, rheumatoid arthritis and/or another demonstrated origin of inflammatory anaemia and/or uncontrolled arterial hypertension, current or previous treatment with EPO or IV iron for at least 3 months, and chronic renal failure receiving haemodialysis or peritoneal dialysis.	• S/C EPO + IV Fe<br>• IV Fe<br>• Placebo	Percentage of patients receiving RBC transfusion	- Survival<br>- Number of RBC transfused/patient<br>- Haemoglobinemia - Health-related quality of life	None	Not stated	Any	Industry
Bidolegui 2014[225]	• Argentina<br>• English<br>• 2014<br>• Single-Centre<br>• 50<br>• Osteoarthritis patient undergoing primary unilateral total knee arthroplasty	Patients who had allergy to tranexamic acid, a prior history of thromboembolic disease, congenital or acquired coagulopathy, renal or liver dysfunction, myocardial infarction within the last 6 months or retinopathy.	• IV TXA<br>• Placebo<br>• -	transfusion rate	Drain output, haemoglobin/haematoc rit levels.	None	Not stated	None	Not stated
Campbell 2012[226]	• UK<br>• English<br>• 2012	Patients older than 70 years of age, those with a known clotting deficiency, those taking	• Intra+Post Cell Salvage<br>• Control	thrombelastometr ic parameters, platelet count	INTEM (ellagic acid activated intrinsic pathway) clotting time,	None	Not stated	None	Not stated

	• Single-Centre<br>• 20<br>• Patients undergoing CABG	warfarin or antiplatelet drugs within 5 days of surgery, or those who had a pre-operative platelet count	• -	after surgery and the amount of blood present in chest drains in the first 4 hours.	clot formation time and maximum clot firmness and FIBTEM (tissue factor-triggered extrinsic pathway with platelet inhibitor) maximum clot firmness were measured by Rotem® (Pentapharm, Munich, Germany) thrombelastometry				
Carvalho 2015[227]	• Brazil<br>• English<br>• 2015<br>• Single-Centre<br>• 125<br>• Patients undergoing total knee arthroplasty	Allergy to TXA or povidone-iodine solution, preoperative anaemia, refusal of blood products, preoperative use of anticoagulants (acetylsalicylic acid, enoxaparin, or any other, oral or intravenous, agent), fibrinolytic disorders, coagulopathy, arterial or venous thromboembolic disease and pregnancy	• Top TXA<br>• Top TXA<br>• Placebo<br>• -	-	Haematimetrics indices (haemoglobin, haematocrit, prothrombin time, activated partial thromboplastin time and international normalised ratio), drain volume (mL), allogenic blood transfusion, thromboembolic events, total calculated blood loss and acute postoperative infec-tion.	None	Not stated	Unclear	Not stated
Castro-Menendez 2016[228]	• Spain<br>• English<br>• 2016<br>• Single-Centre<br>• 240<br>• Patients underwent total hip and knee arthroplasty	Patients with (1) inflammatory or autoimmune disease; (2) blood coagulation disorders; (3) a history of thromboembolic dis-ease; (4) severe anaemia (preoperative Hb <7 mg/dl); (5)peripheral neuropathy; (6) malign tumour; (7) contraindication or intolerance of the administration of low molecular weight heparin or TXA; (8) a history of epilepsy or severe kidney failure, defined as an estimated glomerular filtration rate of <30 mg	• IV TXA (2g)<br>• IV TXA (1g+1g)<br>• No TXA<br>• Restrictive threshold	-	Postoperative blood loss, transfusion rate, and thromboembolic complications	None	Not stated	None	Not stated

		albumin per g of creatinine in urine (9),patients with an ASA score of 4 or 5							
Chareancholvanich 2012a[229]	• Thailand<br>• English<br>• 2012<br>• Single-Centre<br>• 120<br>• Patients who diagnosed primary osteoarthritis and scheduled to undergo primary total knee arthroplasty	Patients who had secondary osteoarthritis (such as rheumatoid arthritis, post-traumatic arthritis, gouty arthritis, post septic arthritis), high risk medical co-morbidity, history of thromboembolic disease, bleeding disorder, known allergy to tranexamic acid, and receiving the anti-coagulant drugs	• IV TXA (post-op)<br>• Placebo<br>• -	-	The amount of drained blood was recorded at 48 hrs. At 48 hours after the operation, the Hb levels of all patients were recorded. Clinical thromboembolic events and wound complications were also examined.	None	Not stated	Unclear	Not stated
Chareancholvanich 2012b[229]	• Thailand<br>• English<br>• 2012<br>• Single-Centre<br>• 120<br>• Patients who diagnosed primary osteoarthritis and scheduled to undergo primary total knee arthroplasty	Patients who had secondary osteoarthritis (such as rheumatoid arthritis, post-traumatic arthritis, gouty arthritis, post septic arthritis), high risk medical co-morbidity, history of thromboembolic disease, bleeding disorder, known allergy to tranexamic acid, and receiving the anti-coagulant drugs	• IV TXA (pre-op)<br>• Placebo<br>• -	-	The amount of drained blood was recorded at 48 hrs. At 48 hours after the operation, the Hb levels of all patients were recorded. Clinical thromboembolic events and wound complications were also examined.	None	Not stated	Unclear	Not stated
Charoencholvanich 2011[230]	• Thailand<br>• English<br>• 2011<br>• Single-Centre<br>• 100<br>• Patients with primary osteoarthritis undergoing unilateral cemented total knee arthroplasty	Patients with secondary osteoarthritis (e.g., rheumatoid arthritis, posttraumatic arthritis, gouty arthritis, post septic arthritis), and patients with a high-risk medical comorbidity, simultaneous bilateral TKAs, history of thromboembolic disease, bleeding disorder, known allergy to tranexamic acid, and receiving anticoagulant drug treatment	• IV TXA<br>• Placebo<br>• -	-	Differences in the mean age, preoperative haemoglobin, volume of drained blood, decrease in haemoglobin 12 hours postoperatively, and the mean number of transfused units	None	Not stated	Unclear	Not stated
Chaudhary 2018[231]	• Pakistan<br>• English<br>• 2018	Patients with abnormal coagulation profile.	• Top TXA<br>• Placebo<br>• -	-	48 hours of blood loss, number of pints transfused,	None	Not stated	Unclear	Not stated

	• Single-Centre<br>• 100<br>• Patients scheduled for primary isolated elective or urgent open heart surgery				perioperative complications, re-exploration for excessive bleeding.				
Chen 2008[232]	• Taiwan<br>• English<br>• 2008<br>• Single-Centre<br>• 60<br>• Patients who underwent head and neck operations	Patients with an allergy to TXA, a history of hematologic disorders, advanced chronic renal insufficiency (creatinine >2mg/dL), undergoing anticoagulation therapy, previous radiation to the head and neck region, or who were reluctant to enrol in this protocol	• IV TXA<br>• No TXA<br>• -	-	Basic data, laboratory study, and operation types, which included gender, age, prothrombin time (PT), activated partial thromboplastin time (aPTT), plasma fibrinogen, D-dimers, and perioperative blood loss, were obtained and recorded.	None	Not stated	None	Non profit
Chen 2016b[233]	• China<br>• English<br>• 2015<br>• Single-Centre<br>• 120<br>• Patients undergoing simultaneous bilateral total knee arthroplasty	Age less than 18, age greater than 80, bleeding or clotting disorders, preoperative anticoagulation therapy, renal disorders or insufficiency, cardiovascular problems, cerebrovascular conditions, thromboembolic disorders, preoperative anaemia, and allergy to TXA	• IV TXA<br>• Placebo<br>• -	total blood loss.	Blood transfusion rate, transfusion units, intraoperative blood loss, drainage volumes, hidden blood loss, maximum decline of haemoglobin, and postoperative suprapatellar girth increment.	None	Not stated	None	Not stated
Cholette 2013[234]	• USA<br>• English<br>• 2013<br>• Single-Centre<br>• 106<br>• Children ≤ 20 kg presenting to the University of Rochester Medical Centre (URMC) for cardiac surgical repair/palliation with CPB	Weight > 21 kg, if their parent/guardian did not speak English, or if consent could not be obtained.	• Cell Salvage<br>• Control<br>• Restrictive threshold	-	Number of RBC and component blood product transfusions, donor exposures, and volume of crystalloid/colloid administered were recorded. Length of mechanical ventilation, vasoactive agents, PCICU and hospital length of stay was followed. Infections (based on clinical and	None	Not stated	Any	Industry

					culture data), bleeding complications and thrombosis (based on clinical and radiographic data) were recorded. Mediastinal tube drainage, Hb, platelet and coagulant protein levels were also followed.				
Cip 2013[235]	• Austria<br>• English<br>• 2013<br>• Single-Centre<br>• 140<br>• Patients treated with primary elective TKA for osteoarthritis from December 2007 to January 2009	Patients not willing to take part in the study or receiving revision arthroplasty	• Cell Salvage<br>• Control<br>• -	-	demographic data, medical history (coronary artery disease, use of anticoagulants, and American Society of Anesthesiologists [ASA] classification [13]), preoperative and postoperative hemoglobin levels, duration of surgery, need for ABT, amount of retransfused WSB, and early complications (including allergic reactions, wound infections, minor and major bleeding, deep venous thrombosis, nerve injuries, pulmonary embolism) at the preoperative examination and during the hospital stay.	None	Not stated	None	Not stated
Colomina 2017[236]	• Spain<br>• English<br>• 2017<br>• Multi-Centre<br>• 95	History of allergy or hypersensitivity to TXA, current treatment with drugs that interfere with coagulation (oral anticoagulant or antiplatelet agents), a clinical history of frequent	• IV TXA<br>• Placebo<br>• Iron therapy<br>• Cell salvage	total number of transfusion units required during the intraoperative and postoperative period up to	Intraoperative blood loss and total blood loss.	None	Not stated	None	Non profit

	• Patients undergoing posterior instrumented spine surgery	bleeding, baseline plasma creatinine>1.5mg dL1, platelet count<150 109 Litre1, prothrombin time (PT)<60% and activated partial thromboplastin time (APTT)>38s, history of any thromboembolic episode before surgery, or a family history of thromboembolism.		postoperative day seven.					
Crescenti 2011[237]	• Italy<br>• English<br>• 2011<br>• Single-Centre<br>• 200<br>• patients older than 18 years and undergoing radical retro-pubic prostatectomy	Patients with atrial fibrillation, coronary artery disease treated with drug eluting stent, severe chronic renal failure, congenital or acquired thrombophilia, and known or suspected allergy to tranexamic acid.	• IV TXA<br>• Placebo<br>• -	number of patients receiving blood tra nsfusions perioperatively	Intraoperative blood los s	None	Not stated	None	Not stated
Das 2015[238]	• India<br>• English<br>• 2015<br>• Single-Centre<br>• 80<br>• Patients, ASA II-III scheduled for unilateral head and neck cancer surgeries	Patients refusal, patients having previous HNC surgery, anaemia (haemoglobin [Hb] <10 mg/dl for women and Hb <12 mg/dl for men), abnormal coagulation profile, aspirin intake within 7 days, hepatorenal insufficiency, cardiopulmonary abnormality, pregnancy, and history of embolic manifestations like deep venous thrombosis, transient ischemic attack, and stroke	• IV TXA<br>• Placebo<br>• -	-	-	None	Not stated	None	Not stated
De Almeida 2015[239]	• Brazil<br>• English<br>• 2015<br>• Single-Centre<br>• 198<br>• All adult patients who had a major surgical procedure for abdominal cancer and	Patients with the following characteristics: age less than 18 yr, haematological malignancy, a Karnofsky score less than 50, pre-existing anaemia (defined as a preoperative haemoglobin concentration <9 g/dl), pre-existing thrombocytopenia	• Restrictive 70g/L<br>• Liberal<br>• -	composite of all-cause mortality or severe clinical complications within 30 days.	major cardiovascular complications, septic shock, acute kidney injury requiring renal replacement therapy, ARDS, and reoperation	None	Not stated	Unclear	Not stated

	required postoperative care in the ICU because of physiological instability and had an expected ICU stay of more than 24 h were included.<br>• Restrictive threshold 7g/dl	(defined as a platelet count <50,000/mm3), pre-existing coagulopathy (defined as a prothrombin time >14.8 s) or anticoagulation therapy, active or uncontrolled bleeding, expected death within 24 h of ICU admission, end-stage renal failure requiring renal replacement therapy, pregnancy, a do-not-resuscitate order, inability to receive transfusion of blood components, or refusal to participate in the study.							
De Napoli 2016[240]	• Argentina<br>• Spanish<br>• 2016<br>• Single-Centre<br>• 62<br>• Patients going under primary hip and knee arthroplasty	-	• IV TXA<br>• Placebo<br>• Restrictive threshold	-	Preoperative and postoperative haematocrit and haemoglobin, days of stay in hospital and number of red cell unit transfusion. We looked for complications and adverse effects.	None	Not stated	None	Not stated
Dell'Atti 2016[241]	• Italy<br>• English<br>• 2016<br>• Single-Centre<br>• 359<br>• Patients taking chronic low dose aspirin, underwent trans-rectal prostate biopsy	Patients with a history of biopsy, surgical treatment of prostatic disease, neoadjuvant therapy or incomplete clinical data	• Oral TXA<br>• No TXA<br>• -	-	Complications, their frequency, severity of bleeding	None	Not stated	none	Not stated
Digas 2015[242]	• Greece<br>• English<br>• 2013<br>• Single-Centre<br>• 90<br>• Patients who underwent unilateral total knee arthroplasty	Patients with secondary and patients with history of thromboembolic disease, bleeding disorder, a history of hepatic or renal dysfunction and severe cardiac respiratory disease.	• IV TXA<br>• IA TXA<br>• Placebo<br>• -	-	Thromboembolic complications, such as clinical deep vein thrombosis and pulmonary emboli, and other complications (e.g., wound complications) were	None	Not stated	Unclear	Not stated

					noted during the hospital stay				
Drakos 2016[243]	• Greece<br>• English<br>• 2014<br>• Single-Centre<br>• 200<br>• Patients over 65years with intertrochanteric fracture treated by intramedullary nail	Polytrauma patients, patients with pathologic fractures or known history of malignancy, delayed surgery beyond 48 hours, known allergy to tranexamic acid, history of venous or arterial thromboembolic disease, hepatic failure, severe renal insufficiency, hematologic disorder, Coumadin anticoagulant medication, and coagulopathy (INR >1.4).	• Top TXA<br>• No TXA<br>• -	-	Complications at the surgical site (hematoma formation, infection and wound dehiscence), deep vein thrombosis, pulmonary embolism, myocardial infarction and cerebral stroke	None	Not stated	Unclear	Not stated
Drosos 2016[244]	• Greece<br>• English<br>• 2016<br>• Single-Centre<br>• 90<br>• Patients who underwent total knee replacement using enhanced recovery after surgery regime	Patients with a history of thromboembolic episode, hepatic/cardiorespiratory/renal insufficiency, and congenital or acquired coagulopathy	• IV TXA<br>• Top TXA<br>• No TXA<br>• -	Calculated blood loss and the need for allogeneic blood transfusion.	complications such as symptomatic deep vein thrombosis (DVT), pulmonary embolism, or any other thromboembolic event, superficial and deep infections and any deterioration of hepatic or renal function during the first 30 post-operative days.	None	Not stated	Unclear	Not stated
Edwards 2009[245]	• UK<br>• English<br>• 2009<br>• Single-Centre<br>• 60<br>• All patients scheduled to undergo bowel resection for suspected colorectal cancer at the centre during the study period.	Patients were excluded if age <18 years, those receiving oral iron/blood transfusion supplementation within 6 weeks of being approached, if the date of their scheduled surgery fell within 15 days of the date of recruitment	• IV Fe<br>• Placebo	Median number of units transfused at peri-operative period.	Transfusion rate<br>- Changes in serum iron markers over the same time period<br>- Length of hospital stay<br>- Adverse perioperative events.	None	Not stated	Any	Industry
Eldaba 2013[246]	• Egypt<br>• English<br>• 2013	Parent refusal, systemic diseases affecting the nose, medical treatment	• IV TXA<br>• No TXA<br>• -	-	Blood loss, time of operation, Side-effects of TA such as nausea, vomiting, pruritus,	None	Not stated	Unclear	Not stated

	• Single-Centre<br>• 100<br>• Children recruited to undergo functional endoscopic sinus surgery	affecting the study or any congenital anomalies, patients with pre-existing renal and hepatic disorders, bleeding diathesis, abnormal prothrombin time, partial thromboplastin time (PTT) or platelet counts, usage of non-steroidal anti-inflammatory drugs within 7 days of surgery			hematoma or haemorrhage, thrombotic complications, local infection, fever or convulsive seizure were reported.				
Elshamaa 2015[247]	• Egypt<br>• English<br>• 2015<br>• Single-Centre<br>• 50<br>• Patients undergoing spine surgery	Patients outside the age range, history of thrombo-embolic event e.g. pulmonary embolism, deep venous thrombosis, traumatic spine injury, morbid obesity (weight > 125 kg), known congenital bleeding disorder, known allergy to the used drugs and known pregnant or lactating patients. Inclusion criteria were the ability to consent, and absence of renal and hepatic diseases.	• IV TXA<br>• No TXA<br>• -	total volume of blood loss in the perioperative period.	Perioperative transfusion requirement, and the number of patients who needed transfusion, as well as time of operation.	None	Not stated	Unclear	Not stated
Elwatidy 2008[248]	• Saudi Arabia<br>• English<br>• 2008<br>• Single-Centre<br>• 64<br>• Patients underwent spinal surgery with expected significant blood loss	Microdiscectomy, and patients on anticoagulation therapy or with coagulopathy, have previous thrombo-embolic events, renal impairment, hepatic disease, as well as patients known to have contraindications to anti-fibrinolytic treatment	• IV TXA<br>• Placebo<br>• -	-	Preoperative, intraoperative, and postoperative haemoglobin (HB) and haematocrit (HCT) values were documented, as well as the amount of blood and blood products transfused during and after surgery.	None	Not stated	None	Non profit
Emara 2014[249]	• Egypt<br>• English<br>• 2014<br>• Single-Centre<br>• 40	Allergy to TXA; acquired disturbances of colour vision; pre-operative anaemia (haemoglobin <11 gm% in females and haemoglobin <12 gm% in males); pre-operative use of anticoagulant therapy,	• IV TXA<br>• Top TXA<br>• Placebo<br>• POC testing	Blood loss	Thromboembolic complications (DVT, PE and cerebrovascular stroke	None	Not stated	None	Not stated

	• Patients who underwent pelvic hemiarthroplasty	heparin within 5 days of surgery, fibrinolytic disorders requiring intraoperative anti-fibrinolytic treatment; coagulopathy i.e., pre-operative platelets count <150,000 mm, international normalized ratio (INR) >1.4 and prolonged prothrombin time (PT) >1.4 s; previous history of thromboembolic disease; significant co-morbidities; severe ischemic heart disease, New York Heart Association Class III and IV; previous myocardial infarction; severe pulmonary disease; plasma creatinine greater than 115 mmol/L in males and more than 100 μmol/L in females; hepatic failure; occurrence of intraoperative surgical/medical/anaesthetic complications; patients who need massive blood transfusion; postoperative bleeding of surgical causes.							
Esfandiari 2013[250]	• Iran<br>• English<br>• 2013<br>• Single-Centre<br>• 150<br>• Patients who were candidates for coronary artery bypass	Patients who had emergency surgery, rheumatic fever, bleeding diathesis (haemophilia or platelet count <100x10^9/L), renal failure (creatinine>160mg/dl), known allergy or contraindication to TA (acquired visual defect, subarachnoid haemorrhage, gall bladder disease, emboli, venous thrombosis), recent (<7 days before surgery) intake of Plavix or heparin, or streptokinase administration within 48 h of operation	• IV TXA<br>• Placebo<br>• -	-	Mortality, MI, Reoperation, Acute tubular necrosis, Cerebrovascular accident	None	Not stated	None	Not stated

Fan 2014[251]	• China<br>• English<br>• 2014<br>• Single-Centre<br>• 186<br>• Consecutively admitted patients, with the age of more than 65 years, undergoing elective unilateral total hip replacement from October, 2011 to May 2013 were enrolled in the present study.<br>• Restrictive threshold 8g/dl	The exclusion criteria were as follows: ASA physical status ≧ IV; preoperative delirium; unwilling to comply with the procedures; inability to understand the language (Mandarin Chinese); hearing loss, or a failure in spinal anaesthesia.	• Restrictive 80g/L<br>• Liberal<br>• -	-	Delirium, cerebrovascular accident, cardiac failure, myocardial infarction, pulmonary embolism, pneumonia, superficial wound infection, urinary tract infection, acute renal failure	None	Not stated	None	Non profit
Faraoni 2014[252]	• USA<br>• English<br>• 2014<br>• Single-Centre<br>• 33<br>• Cardiac surgery patients requiring cardiopulmonary bypass	Cmergency procedures, previous sternotomy, endocarditis, complex surgeries of the aortic arch, preoperative severe chronic kidney injury (creatinine level >180mmol l1), preoperative haemoglobin level less than 10 g dl1 , preoperative coagulopathy, history of stroke or thrombo-embolic disease, allergy or contraindication to tranexamic acid.	• IV TXA (High)<br>• IV TXA (Low)<br>• Placebo<br>• POC testing	Fibrinolysis was evaluated by thromboelastogra phy	Blood loss, transfusion requirement and side effects.	None	Not stated	None	Non profit
Farrokhi 2011[253]	• Iran<br>• English<br>• 2009<br>• Single-Centre<br>• 92<br>• Patients undergoing spinal fixation surgery, aged 40 to 80 years, with physical status I and II	Platelet count <150,000mm^3, heart disease, severe allergy to TXA, body mass index >30 kg/m2, and history of bleeding disorders.	• IV TXA<br>• Placebo<br>• -	-	Administered liquids (crystalloids, colloids), blood transfusions, and urine output were measured at the end of recovery. Patients were assessed daily for any thromboembolic complications.	None	Not stated	Any	Industry
Fernandez-Cortinas 2017[254]	• Spain<br>• English<br>• 2017<br>• Single-Centre	Patients allergic to TXA, those with liver failure, haematological diseases, retinopathy, cerebrovascular	• IV TXA<br>• Placebo<br>• -	-	-	None	Not stated	Unclear	Not stated

	• 134<br>• Patients who have undergone total hip arthroplasty operation	disease, severe ischaemic cardiopathy, severe kidney failure, severe lung failure, INR > 1.4, coagulopathies, and a background of arterial or venous thromboembolic disease.							
Foss 2009[255]	• Denmark<br>• English<br>• 2009<br>• Single-Centre<br>• 120<br>• Inclusion criteria were primary hip fracture occurring in the community in patients older than 65 years of age with an independent pre-fracture walking function, community dwelling, and intact cognitive status.<br>• Threshold 8g/dl	Patients with multiple fractures, pre-fracture terminal condition, alcoholism, chronic transfusion needs, acute cardiac or other acute severe medical conditions, or contraindication to epidural analgesia were excluded.	• Restrictive 80g/L<br>• Liberal<br>• -	-	Ambulatory capacity, mortality, length of stay, cardiac complications, infectious complications	None	Not stated	None	Non profit
Fraval 2016[256]	• Australia<br>• English<br>• 2015<br>• Single-Centre<br>• 101<br>• Patients who underwent total hip arthroplasty	Patients with contraindications to the use of TXA such as known drug reaction to TXA, active intravascular clotting (deep vein thrombosis [DVT], pulmonary embolism [PE], or cerebral thrombosis), predisposition to thrombosis (previously documented DVT or PE), or a subarachnoid haemorrhage. Patients with rheumatoid arthritis	• IV TXA<br>• Placebo<br>• -	thigh swelling	Visual analogue pain score, timed up and go test, a 10 meter walk test, and length of stay. Blood loss and the incidence of blood transfusions were also recorded.	None	Not stated	None	Not stated
Fraval 2018[257]	• Australia<br>• English<br>• 2016<br>• Single-Centre<br>• 105<br>• Patients undergoing elective total hip	Patients with contraindications to the use of tranexamic acid such as known drug reaction to TXA, active intravascular clotting (DVT, pulmonary embolism [PE] or cerebral thrombosis), predisposition to	• IV TXA<br>• Placebo<br>• -	thigh swelling	Blood loss and the incidence of blood transfusions was also recorded. Secondary outcome measures including postoperative functional scores and	None	Not stated	None	Not stated

	arthroplasty for the treatment of osteoarthritis over the age of 40 years.	thrombosis (previously documented DVT or PE) or a subarachnoid haemorrhage. Patients with rheumatoid arthritis were also excluded.			mobility, pain scores and length of stay.				
Froessler 2016[258]	• Australia<br>• English<br>• 2014<br>• 72<br>• Patients undergoing abdominal surgery with iron deficiency anaemia between August 2011 and November 2014. (>18 yrs with IDA, ferritin <300 mcg/L, transferrin saturation <25%, Hb <12.0 g/dL for women, Hb <13.0 g/dL for men	Not stated	• IV Fe<br>• Standard Care	Incidence of Autologus Blood Transfusion	- Hemoglobin (Hb) on admission<br>- Hb difference from randomization to admission<br>- ICU admission<br>- Perioperative morbidity (defined as new onset infection, respiratory failure, renal impairment, deep venous thrombosis)<br>- Discharge Hb<br>- Length of stay<br>- Hb at follow-up<br>- Hb difference from discharge to follow-up<br>- Iron status<br>- 30-day mortality<br>- Quality of life (QoL)	None	Not stated	None	Not stated
Garrido-Martin 2012[259]	• Spain<br>• English<br>• 2012<br>• Single-Centre<br>• 210<br>• Patients older than 18 years of age, elective cardiac surgery under extracorporeal circulation, without previous anaemia, susceptible to treatment, without preoperative blood transfusion, able to complete all study visits per protocol and providing written informed consent	Elective cardiac surgery patients without extracorporeal circulation, treatment with fibrinolytic therapy 48 h before CPB surgery, history of impaired renal function (creatinine clearance <50 ml/min), previous surgery for active endocarditis, redo-surgery patients, pregnant or lactating, signs of active gastrointestinal bleeding, vitamin B12 deficit, ferropenic anaemia, clinical history of asthma or allergy, active infection, included in another clinical study, hepatic	• IV Fe<br>• Oral Fe<br>• Placebo	Number of patients transfused at end of follow up	- Protocol outcomes not reported by the study Quality of life at end of follow-up<br>- Length of hospital stay at end of follow-up<br>- Mortality (all causes) at 30 days<br>- Mortality (transfusion related) at 30 days<br>- Infections (includes pneumonia, surgical site infection, UTI and septicaemia/bacteraemi a) at within 30 days of surgery	None	Not stated	None	Not stated

		disease, history of allergy to iron, unlikely to adhere to protocol follow-up, unable to comply with the study protocol.			- Bleeding at end of follow-up<br>- Serious adverse events (as described in studies) at end of follow-up<br>- Mortality (all causes) at 1 year<br>- Thrombosis at end of follow-up<br>- Number of units transfused at end of follow-up				
Gatling 2018[260]	• USA<br>• English<br>• 2018<br>• Single-Centre<br>• 82<br>• Patients scheduled for primary cardiac surgery with anticipated CPB.	Patients were excluded if they weighed < 30 kg, had pre-existing coagulopathy (INR > 1.5, platelets < 100 ×109/L), had renal failure (defined as BUN / Cr ≥ 20: 1), had severe liver disease (AST&ALT > 3x normal), or were undergoing cardiac surgery known to be associated with greater risk for bleeding and transfusion such as complex aortic surgery, or combination valve replacement with coronary artery bypass graft surgery.	• IV TXA<br>• EACA<br>• Restrictive threshold	difference in transfusion amounts	the amount of transfusion during the operative procedure, calculated Red blood cell (RBC) volume change, postoperative creatinine, time to extubation, chest tube output and length of ICU stay.	None	Not stated	None	Not stated
Gautam 2013[261]	• India<br>• English<br>• 2013<br>• Single-Centre<br>• 27<br>• Patients who underwent total knee arthroplasty	Patients who were allergic to tranexamic acid or having inherited or acquired hypercoagulable state, abnormal coagulation profile (BT, CT, platelet count, prothrombin time, aPTT), patients who had taken aspirin or other NSAIDS 3 days prior to surgery, patients with renal insufficiency or history of deep vein thrombosis or pulmonary embolism and people who were at risk of these	• IV TXA<br>• No TXA<br>• -	-	Blood loss, general condition and vitals were assessed.	None	Not stated	Unclear	Not stated

Geng 2017[262]	• China<br>• English<br>• 2017<br>• Single-Centre<br>• 100<br>• Patients who underwent spinal tuberculosis surgery	1. People suffering from the second surgery of spine tuberculosis; 2. Tranexamic acid allergy; 3. People who previously used warfarin and other anticoagulant drugs; 4. People with severe renal insufficiency, renal pelvis or ureteral solid lesions, diabetes and other diseases that may affect coagulation function; 5. People who had previous history of deep vein thrombosis.	• IV TXA<br>• No TXA<br>• -	-	Blood loss during operation, the postoperative drainage volume within 48 hours after operation, the postoperative haemoglobin (HB) and haematocrit (HCT).	None	Not stated	Unclear	Not stated
Girdauskas 2010[263]	• Germany<br>• English<br>• 2010<br>• Single-Centre<br>• 56<br>• adult patients (> 18 years) undergoing high risk aortic surgery including urgent and emergency surgery (25 with acute type A dissection) with hypothermic circulatory arrest	Pregnant, known (inherited) coagulation disorders (haemophilia A or B, activated protein C resistance, etc), inability to give informed consent	• ROTEM<br>• Control<br>• Tranexamic acid<br>• Restrictive Threshold<br>• Cell Salvage	cumulative transfusion of allogeneic blood units (PRBCs, FFP, and platelets)	use of prothrombin complex concentrate, fibrinogen concentrate, and recombinant factor VIIa (NovoSeven), blood losses in the first 12 and 24 postoperative hours, risk of surgical re-exploration for bleeding, time to extubation, neurologic and renal complications, length of stay in ICU	None	Not stated	None	Not stated
Guerreiro 2017[264]	• Brazil<br>• English<br>• 2015<br>• Single-Centre<br>• 43<br>• Patients who underwent total knee arthroplasty	patients with major deformities that would lead to bone cuts or release of a more extensive area of soft tissue; presence of inflammatory diseases; patients who had undergone previous surgeries of the same knee; use of anticoagulation medication up to seven days before surgery; and patients with history of atrial fibrillation, deep vein thrombosis or prior pulmonary embolism.	• IV TXA<br>• Placebo<br>• -	-	1. Haemoglobin (Hb) levels preoperatively and 24 and 48 hours after surgery.<br>2. Reports of clinical flexion gain examination using a goniometer for evaluations 24 hours, 48 hours, 7 days, 21 days and 2 months after surgery.	None	Not stated	None	Not stated

					3. Pain evaluation using a visual analogue scale (VAS)<br>4. Evaluations of knee function, preoperatively and 2 months after surgery, using the"WOMAC" instrument, were translated and validated for the Portuguese language				
Gupta 2012[265]	• India<br>• English<br>• 2011<br>• Single-Centre<br>• 60<br>• Adult consented female patients, ASA class I and II, scheduled for elective radical surgery	Patients with an allergy to medication (tranexamic acid), anaemia, preoperative hepatic or renal dysfunction, serious cardiac or respiratory disease, congenital or acquired coagulopathy or a history of deep vein thrombosis/thromboembolic disease	• IV TXA<br>• Placebo<br>• -	-	Blood Loss<br>All patients' preoperative and 12th hour postoperative blood samples were analysed for haemoglobin, haematocrit, platelet count, prothrombin time (PT), activated partial thromboplastin time (aPTT), serum creatinine, fibrinogen, D-dimer and symptoms of pulmonary embolism such as dyspnea, haemoptysis, pleuritic chest pain, apprehension, tachypnea, tachycardia, rales etc. Doppler ultrasound of lower limbs was done daily in all patients for signs of deep vein thrombosis (DVT).	None	Not stated	None	Not stated
Guzel 2016[266]	• Turkey<br>• English<br>• 2014<br>• Single-Centre	Patients with a history of venous thromboembolism, preoperative use of	• IV TXA<br>• No TXA<br>• Cell salvage	-	-	None	Not stated	Unclear	Not stated

	• 100<br>• Patients who underwent primary unilateral total knee arthroplasty	anticoagulants (acetylsalicylic acid, enoxaparin, or any other oral or intravenous agent), obvious anaemia or coagulopathy before surgery							
Haghighi 2017[267]	• Iran<br>• English<br>• 2017<br>• Single-Centre<br>• 38<br>• Patient who were undergoing surgery for femoral shaft fractures in trauma setting	Coronary artery disease, history of arterial fibrillation, thrombophilia, chronic renal failure, haemoglobin<10 g/dl, thromboembolic episodes (DVT or pulmonary embolus), taking anticoagulant medication or oral contraceptive pills (OCP) and allergy to TA, presence of subarachnoid haemorrhage (SAH), pregnancy and breast feeding	• IV TXA<br>• Placebo<br>• -	-	The total amount of blood transfusion during operation and four hours after the surgery was measured	None	Not stated	None	Non profit
Hashemi 2011[268]	• Iran<br>• English<br>• 2009<br>• Single-Centre<br>• 100<br>• Patients undergoing on-pump coronary artery bypass grafting surgery (CABG)	Patients with a history of haemorrhagic tendency and blood dyscrasia, history of Plavix usage, known hepatic, renal and metabolic diseases, use of other anti-coagulation drugs like Comadin for valvular disease and arrhythmias and streptokinase, emergency surgery, rheumatic heart disease, known allergy to Aprotinin or Transamine and prohibition for their use such as acquired visual defects and retinal disease, subarachnoid haemorrhage, disseminated intravascular coagulation, gall bladder disease, leukaemia, embolization, and vein thrombosis.	• IV TXA<br>• Placebo<br>• -	-	Post-operative complications like post-operative MI (based on cardiac enzyme rising, ECG changing and EF changing estimated by echocardiography), Neurological complications (estimated by clinical examination and CT-Scanning), redo operation for surgical bleeding and pericardial effusion, kidney complication(rising of serum creatinine and low urinary out put under 0.5 cc per minute) and other complications were studied.	None	Not stated	Unclear	Not stated

Hogan 2015[269]	• United Kingdom<br>• English<br>• 2015<br>• Single-Centre<br>• 53<br>• Patient undergoing elective or urgent CABG or valve surgery or both utilizing CPB	Emergency surgery, a contra-indication to either heparin, protamine or tranexamic acid, or inability to understand the study protocol.	• Post Cell Salvage<br>• Non Cell Salvage Transfusion<br>• Tranexamic acid	haemoglobin concentration after autotransfusion	red cell or blood product transfusions, total fluid administration or blood loss in the first 12 h, and ICU length of stay.	None	Not stated	Any	Industry
Hooda 2017[270]	• India<br>• English<br>• 2017<br>• Single-Centre<br>• 60<br>• Adults undergoing elective craniotomy for meningioma excision	Patients who refused to participate in the study or were allergic to tranexamic acid, had a history suggestive of bleeding diathesis, thromboembolic episode prior to surgery or family history of thromboembolism, patients on medication that could interfere with coagulation, epilepsy, plasma creatinine values more than 1.5 mg/dl and pregnant or lactating mothers	• IV TXA<br>• Placebo<br>• Cell salvage	intra-operative blood loss and transfusion requirements	The effect of tranexamic acid on the quality of surgical haemostasis, perioperative complications, length of hospital stay and neurological outcome were also evaluated.	None	Not stated	Unclear	Not stated
Horstmann 2013[271]	• Netherlands<br>• English<br>• 2013<br>• Single-Centre<br>• 204<br>• Total hip arthroplasty patients	Coagulation disorders including deep venous thrombosis and pulmonary embolism, malignancy, ongoing infections, untreated hypertension, unstable angina pectoris, myocardial infarction within the past 12 months, coronary bypass operation within the past 12 months, intake of anticoagulants or participation in other clinical trials dealing with any drugs that affect blood loss.	• Intra+Post Cell Salvage<br>• Control<br>• -	Hb level on the first postoperative day	Hb levels on the day of surgery, the second and third days, the lowest post-operative level, any HBT requirement, adverse events, and total blood loss.	None	Not stated	Any	Not stated
Hosseini 2014[272]	• Iran<br>• English<br>• 2011<br>• Single-Centre<br>• 71	Patients with clotting disorders, kidney failure (Cr> 1.7), allergy to tranexamic acid, consumption of antiplatelet drugs, prescription of heparin	• IV TXA<br>• Placebo<br>• -	-	Patients were examined to find any deep veins thrombosis (DVT), renal failure and cerebrovascular	None	Not stated	None	Not stated

	• Patients who underwent off pump CABG	48 h prior to surgery and patients with ejection fraction (EF) <40.			accident (CVA). The amount of blood products including packed red blood cells (RBCs), FFP and platelets were recorded for each group.				
Hsu 2015[273]	• Taiwan<br>• English<br>• 2015<br>• Single-Centre<br>• 60<br>• Patients underwent unilateral minimally invasive uncemented total hip arthroplasty	Patients with a pre-operative level of haemoglobin was < 10 g/dl, or there was a history of ischaemic heart disease, myocardial infarction, cerebrovascular disease, thromboembolic disease or ipsilateral infection of the hip.	• IV TXA<br>• Placebo<br>• -	-	Blood loss	None	Not stated	Unclear	Not stated
Huang 2016[274]	• China<br>• English<br>• 2014<br>• Single-Centre<br>• 108<br>• Patients who underwent total knee arthroplasty	Patients presenting with any blood disease, or diabetes, or any coagulation disorders or any history of thromboembolism.	• IV TXA<br>• Placebo<br>• -	-	The volumes of blood loss, drainage and transfusion in each group were recorded to calculate the measured/hidden red blood loss (RBL). Haematocrit (Hct) was recorded preoperatively and 72 h postoperatively.	None	Not stated	None	Non profit
Husted 2003[275]	• Denmark<br>• English<br>• 2003<br>• Single-Centre<br>• 40<br>• Patients scheduled for primary total hip arthroplasty	Patients with rheumatoid arthritis, malignancy, previous thrombo-embolic episodes, ischemic heart disease, previous subarachnoid bleeding, haematuria and body weight > 100 kg.	• IV TXA<br>• Placebo<br>• -	-	Perioperative blood loss and number of transfusions	None	Not stated	Unclear	Not stated
Jendoubi 2017a[276]	• Tunisia<br>• French<br>• 2017<br>• Single-Centre<br>• 60	Patients with ASA III or IV, with a known or suspected allergy to tranexamic acid (ATX) or to the excipient, presenting a medical contraindication to the use of ATX: history of	• IV TXA<br>• Placebo<br>• -	-	Blood loss was evaluated in terms of reduction in the serum haemoglobin level	None	Not stated	Unclear	Not stated

	• Patients, ASA status I or II, undergoing endoscopic transurethral resections (TURP)	convulsion, severe renal insufficiency (creatinine clearance <30 mL / min), coagulopathy, history of venous thromboembolism (deep vein thrombosis, pulmonary embolism) and / or arterial (angina, myocardial infarction, stroke, Acute leg ischemia), atrial fibrillation or acquired or congenital thrombophilia were not included in the study.							
Jendoubi 2017b[276]	• Tunisia<br>• French<br>• 2017<br>• Single-Centre<br>• 71<br>• Patients, ASA status I or II, undergoing endoscopic transurethral resections (TURBT)	Patients with ASA III or IV, with a known or suspected allergy to tranexamic acid (ATX) or to the excipient, presenting a medical contraindication to the use of ATX: history of convulsion, severe renal insufficiency (creatinine clearance <30 mL / min), coagulopathy, history of venous thromboembolism (deep vein thrombosis, pulmonary embolism) and / or arterial (angina, myocardial infarction, stroke, Acute leg ischemia), atrial fibrillation or acquired or congenital thrombophilia were not included in the study	• IV TXA<br>• Placebo<br>• -	-	Blood loss was evaluated in terms of reduction in the serum haemoglobin level	None	Not stated	Unclear	Not stated
Jimenez 2007[277]	• Spain<br>• English<br>• 2007<br>• Single-Centre<br>• 160<br>• Elective cardiopulmonary bypass patients	No informed consent, age < 18 years, emergencies, off-pump cardiac surgery, chronic coagulopathy (prothrombin time [PT] <50% or international normalized ratio (INR) >2 and platelets <50,000/ mm3 or aggregation dysfunction), renal	• IV TXA<br>• No TXA<br>• -	-	Core body temperature, laboratory data (haematology, inflammation, coagulation, and fibrinolysis), and hemodynamic parameters were	None	Not stated	None	Non profit

		failure (creatinine >2 mg/dL), gross haematuria, TA hypersensibility, chronic hepatopathy (Child-B or higher), immunosuppression, endocarditis and post-operative sepsis within 24h			recorded before intervention (baseline), on ICU admission after surgery (0 h), and at 4 h and 24 h post-CPB, once hemodynamic stability was confirmed. We also recorded blood loss (chest-tube drainage and hemoderivatives) at the above time points and on chest tubes removal.				
Johansson 2005[278]	• Sweden<br>• English<br>• 2005<br>• Single-Centre<br>• 100<br>• Patients receiving total hip arthroplasty	History or laboratory signs of bleeding disorders, malignancy and rheumatic joint disease, consumption of aspirin or NSAIDs within a week before surgery, history of coagulopathy or thrombo-embolic events and plasma creatinine levels above 115 µmol/L in men and 100 µmol/L in women.	• IV TXA<br>• Placebo<br>• -	-	Total blood loss was calculated from the haemoglobin (Hb) balance. Volume and Hb concentration of the drainage was measured 24 h after the operation. Intraoperative blood loss was estimated volumetrically and visually.	None	Not stated	None	Non profit
Karaaslan 2015a[279]	• Turkey<br>• English<br>• 2015<br>• Single-Centre<br>• 81<br>• Patients who underwent arthroscopic anterior cruciate ligament reconstruction	Bleeding or clotting disorders, preoperative anticoagulation therapy, abnormal coagulation profile, renal disorders or insufficiency, sickle cell disease, and allergy to local anaesthetics/TXA.	• IV TXA<br>• Placebo<br>• -	-	The amount of drained blood. Thromboembolic and other complications were noted during the hospital stay	None	Not stated	Unclear	Not stated
Karaaslan 2015b[280]	• Turkey<br>• English<br>• 2015<br>• Single-Centre<br>• 105	Bleeding or clotting disorder, preoperative anticoagulation therapy, abnormal coagulation profile, renal disorder or insufficiency, sickle cell disease, allergy to local anaesthetics/ TXA, significant preoperative	• IV TXA<br>• Placebo<br>• -	grade of hemarthrosis, according to the classification of Coupens and Yates, and pain was measured by	VAS for pain score, hemarthrosis grade, range of motion (ROM), as well as the presence of any complications were documented. Patient satisfaction and	None	Not stated	Unclear	Not stated

	• Patients who underwent simultaneous bilateral total knee arthroplasty	pain (VAS score .5), large preoperative swelling (grade 3 or 4 effusion), or a revision case.		a visual analog scale (VAS)	knee function were recorded.				
Kazemi 2010[281]	• Iran<br>• English<br>• 2010<br>• Single-Centre<br>• 64<br>• Patients who underwent total hip arthroplasty	Patients with previous hip surgery, drug sensitivity, anaemia (haemoglobin <11.5 for females and <12.5 for males), congenital or acquired haemostatic disease, disturbed coagulation and platelet count, hepatic or renal failure, pregnancy, history of DVT (deep vein thrombosis) or embolism and atherosclerotic vascular disease	• IV TXA<br>• Placebo<br>• -	-	6- and 24-hour postoperative haemoglobin levels, intraoperative and postoperative bleeding, and allogenic blood transfusion	None	Not stated	Unclear	Not stated
Kim 2016[282]	• Korea<br>• English<br>• 2015<br>• Single-Centre<br>• 48<br>• Patients who underwent posterior lumbar interbody fusion	Patients with previous spinal surgery, previous or current bleeding or coagulation issues, established renal or hepatic diseases, or contraindication to antifibrinolytic agents	• IV TXA<br>• Placebo<br>• -	amount of intraoperative and postoperative blood loss.	-	None	Not stated	None	Not stated
Kim 2018[283]	• Korea<br>• English<br>• 2018<br>• Single-Centre<br>• 48<br>• Patients who underwent unilateral or bilateral total knee arthroplasty	Exclusion criteria were as follows: platelet count (PLT), < 50 × 10^3/μL; prothrombin time (PT) or activated partial thromboplastin time (aPTT) > 1.5 times the reference value; history of convulsive seizure, epilepsy, or brain surgery; treatment with a non-steroidal anti-inflammatory agent within the previous 2 days; treatment with aspirin within 14 days prior to surgery; and known allergy to TXA.	• IV TXA<br>• Placebo<br>• POC testing	blood loss during surgery		None	Not stated	None	Non profit
Kimenai 2016[284]	• Netherlands<br>• English<br>• 2016	Emergency cardiac interventions, minimally invasive surgery (port access	• IV TXA<br>• Placebo<br>• POC testing	12-h postoperative blood loss	Number of transfusion-free patients, the amount of blood	None	Not stated	None	Not stated

	• Single-Centre<br>• 500<br>• Adults aged 18 or older, scheduled for elective cardiac surgery on cardiopulmonary bypass	surgery, thoracoscopic surgery or mini-sternotomy), off-pump procedures and patients with an increased or decreased bleeding tendency (Factor V Leiden thrombophilia, protein C deficiency, protein S deficiency, anti-thrombin deficiency and prothrombin mutation).			component transfusions given, the variables of routine coagulation tests, morbidity and in-hospital mortality.				
Kulkarni 2016[285]	• India<br>• English<br>• 2016<br>• Single-Centre<br>• 219<br>• Patients undergoing major head and neck cancer surgeries	Patients with coagulopathy (partial prothrombin time >50 s, or international normalised ratio >1.5, platelets <50 × 10^9/L), or those who had recent history of (<5 days) acetylsalicylic acid ingestion, patients on anticoagulant therapy (heparin received within 4 h or warfarin received 3 days pre-operatively) or those with peripheral vascular disease, pre-existing renal dysfunction (serum creatinine >1.2 mg/dL), liver dysfunction or known allergy to TA were excluded.	• IV TXA<br>• Placebo<br>• POC testing<br>• Restrictive threshold	reduction in blood loss	the number of patients needing transfusion.	None	Not stated	None	Non profit
Kultufan Turan 2006[286]	• Turkey<br>• Turkish<br>• 2010<br>• Single-Centre<br>• 40<br>• Cardiac surgery either CABG or valve surgery	None stated	• TEG<br>• Control<br>• -	incidence of blood transfusion (whole blood, RBCs, FFP, and platelets)	-	None	Not stated	None	Not stated
Kundu 2015[287]	• India<br>• English<br>• 2014<br>• Single-Centre<br>• 60	Patients with history of previous ipsilateral knee surgery, suspected allergy to medication (TA, local anaesthetics, low-molecular weight heparin), anaemia (haemoglobin [Hb] <10 mg/dl	• IV TXA<br>• Placebo<br>• Restrictive threshold	-	Number of transfusion given to the patients.	None	Not stated	None	Not stated

	• Patients undergoing unilateral total knee replacement	for women and Hb <12 mg/dl for men), abnormalities in coagulation screening tests, aspirin intake within 7 days of surgery, renal (serum creatinine >2 standard deviation [SD] for age) or hepatic insufficiency, pregnancy and history of deep vein thrombosis (DVT) or pulmonary embolism, transient ischemic attack and stroke were excluded.							
Lack 2017[288]	• USA<br>• English<br>• 2017<br>• Single-Centre<br>• 88<br>• Patients undergoing unilateral total knee replacement	History of VTE or a baseline hypercoagulable state (ie, factor V Leiden and antiphospholipid antibody).	• IV TXA<br>• Placebo<br>• Cell salvage	allogeneic blood transfusion	estimate blood loss (EBL) and venous thromboembolism (VTE).	None	Not stated	None	Non profit
Lacko 2017[289]	• Slovakia<br>• English<br>• 2017<br>• Single-Centre<br>• 60<br>• Patients with knee osteoarthritis undergoing unilateral cemented total knee replacement	Patients with known TA allergy, history of thromboembolism, cerebrovascular accidents, severe liver and kidney disease or blood clotting disorders.	• IV TXA<br>• No TXA<br>• Restrictive threshold	-	perioperative blood loss and blood loss to drainage for 24 hours postoperatively, time of operation and the occurrence of postoperative complications in the period of three months.	None	Not stated	None	Not stated
Laoruengthana 2019a[290]	• Thailand/USA<br>• English<br>• 2016<br>• Single-Centre<br>• 228<br>• All patients with the diagnosis of primary osteoarthritis of the knee scheduled for primary unilateral TKA	Patients with preoperative haemoglobin of less than 10 g/dL, previous history of a thromboembolic event, renal insufficiency, cardiovascular disease or cerebrovascular accident were excluded. Patients with a bleeding disorder and patients requiring anticoagulant therapy were also excluded.	• No TXA<br>• IA TXA<br>• IV TXA<br>• -	-	Blood loss (CBL), drain volume (DV) and an average number of units of blood transfused (ANUBT).	None	Not stated	Unclear	Not stated

Lee 2017[291]	• Hong Kong<br>• English<br>• 2015<br>• Single-Centre<br>• 189<br>• Patients with primary total knee replacement	Patients with bilateral arthroplasty, thromboembolic diseases, history of clotting disorder or drug history of antiplatelet, anticoagulant, or deep vein thrombosis (DVT) prophylaxis in the perioperative period, complicated primary total hip arthroplasties with osteotomy, pre-existing implant removal or bone grafting, renal disease, and history of allergy to TXA.	• PO TXA<br>• No TXA<br>• Restrictive threshold	Hb drop	Intraoperative blood loss, drain output, total blood loss (TBL), hidden blood loss, transfusion requirement, thromboembolic complications, cerebrovascular or cardiovascular complications and 30-day mortality.	None	Not stated	None	Not stated
Lei 2017[292]	• China<br>• English<br>• 2017<br>• Single-Centre<br>• 77<br>• Patients undergoing hip surgery for intertrochanteric fracture	Revisions, bilateral procedures, flexion deformity ≥30°, varus/valgus deformity ≥ 30°, patients with anaemia (<120 g/L for female, <130 g/L for male), pre-operative hepatic or renal dysfunction, serious cardiac or cerebrovascular problems, previous history of deep venous thrombosis or pulmonary embolism, congenital or acquired clotting disorders, contraindications for the use of TXA.	• IV TXA<br>• Placebo<br>• -	-	Haemoglobin and haematocrit levels 1 day before surgery and on postoperative Day 1 and 3; duration of surgery; and visible blood loss collected with a sterile plastic foil, a funnel, and gauzes were measured. Complications associated with surgery—including hematoma, infection, deep vein thrombosis (examined by ultrasonography on day 3 post-operation), pulmonary embolism, myocardial infarction, ischemic cerebral infarction, respiratory infection, and renal failure—were also recorded.	None	Not stated	None	Non profit
Liang 2014[293]	• China<br>• English<br>• 2014<br>• Single-Centre	Scoliosis patients who underwent osteotomy, growing rod extending or revision surgery, with a history of a bleeding disorder, a low	• Intra Cell Salvage<br>• Normal Drainage<br>• Iron Therapy	-	perioperative haemoglobin levels, surgical time, levels fused, perioperative estimated blood loss,	None	Not stated	None	Not stated

	• 110<br>• scoliosis patients undergoing posterior instrumented spinal fusion between January 2012 and June 2013 at a single hospital	platelet count (<150,000), abnormal partial thromboplastin time or international ratio test, previous thromboembolic event, or a family history of thromboembolism	• Restrictive Threshold		perioperative transfusions and incidence of transfusion-related complications.				
Lidder 2007[294]	• UK<br>• English<br>• 2007<br>• Single-Centre<br>• 49<br>• Patients diagnosed with colorectal cancer who are fit for surgery	Not stated	• Oral Fe<br>• Standard Care<br>• -	-	Functional Recovery<br>Hospital LOS<br>Risk & number of RBC transfusion<br>Perioperative blood loss	None	Not stated	Unclear	Not stated
Lin 2012[295]	• Taiwan<br>• English<br>• 2010<br>• Single-Centre<br>• 151<br>• Patients undergoing unilateral minimally invasive TKR	Patients with a history of previous surgery on the same knee, thromboembolic disease, myocardial infarction, cerebrovascular disease or a pre-operative haemoglobin < 10 g/dl were excluded from the trial.	• IV TXA (2 dose)<br>• IV TXA (1 dose)<br>• Placebo<br>• Restrictive threshold	-	The volume of blood drained was recorded every two hours during the first eight post-operative hours, and then every eight hours until the drains were removed on the second post-operative day. The haemoglobin and haematocrit were checked on the first, second, and fourth days after operation.	None	Not stated	None	Non profit
Liu 2017[296]	• China<br>• English<br>• 2015<br>• Single-Centre<br>• 224<br>• Patients undergoing total knee arthroplasty<br>• 1) Participants: patients undergoing primary THA. 2) Intervention: combined topical with intravenous TXA. 3) Comparison: IV TXA	Articles that without the outcome measures of interest. 2) Quasi-RCT or non-RCT. 3) Retrospective studies, letters, comments, editorials and practice guidelines.	• IV TXA (low dose)<br>• IV TXA (high dose)<br>• Placebo<br>• POC testing	-	The intraoperative blood loss, postoperative drainage volume, occult blood loss, blood transfusion rate, and blood transfusion volume in each group were recorded	None	Not stated	None	Non profit

	alone. 4) Outcomes: the primary outcomes included total blood loss, hidden blood loss, transfusion rate, and postoperative complications (including DVT/pulmonary embolism (PE)). Secondary outcomes included haemoglobin drop and length of hospital stay. 5) Study: only RCTs were included.								
Lopez-Hualda 2018	• Spain<br>• English<br>• 2018<br>• Single-Centre<br>• 90<br>• Patients scheduled for unilateral total knee arthroplasty	The exclusion criteria were having had previous coagulopathies and receiving chronic anticoagulant treatment.	• IV TXA<br>• Top TXA<br>• No TXA<br>• Restrictive threshold	-	Blood loss and drain outputs	None	Not stated	Unclear	Not stated
Lundin 2013[297]	• Sweden<br>• English<br>• 2012<br>• Single-Centre<br>• 100<br>• Women undergoing radical debulking ovarian cancer surgery	Patients with an allergy to tranexamic acid; treatment with anticoagulants within the past month; a history or present laboratory signs of bleeding disorders, coagulopathy or thromboembolic events; a history of myocardial infarction within the last year; present unstable angina or severe coronary disease; reduced renal function with plasma creatinine levels above 250 µmol/L, and severe psychiatric or mental disorder	• IV TXA<br>• Placebo<br>• -	Blood loss and red blood cell transfusions.		None	Not stated	None	Non profit
Luo 2019[298]	• China<br>• English<br>• 2017<br>• Single-Centre<br>• 90	(1) preoperative examination revealed DVT; (2) they had any contraindication for anticoagulation therapy; (3) they had a pathological	• IV TXA<br>• Placebo<br>• -	perioperative blood loss	Postoperative transfusion rate, postoperative haemoglobin level, and length of the hospital	None	Not stated	None	Not stated

	• (1) had intertrochanteric fracture (extracapsular fractures of AO/OTA types 31-A1 to 31-A3) treated with PFNA, (2) closed fracture with low-energy damage, and (3) age ≥60 years.	fracture; (4) they had one of the following diseases in the preceding year: myocardial infarction, cerebral infarction, coronary syndrome, DVT, or pulmonary embolism; (5) the duration from injury to operation was >3 weeks; (6) they had allergy to TXA; (7) patients who had adverse drug reactions when using TXA and stopped the medication; (8) they had multiple fractures, with the other fracture also needing surgical treatment; (9) preoperative hemoglobin (Hb) was <8 g/dL; (10) closed reduction failed, and therefore open reduction was performed; and (11) there was any change in the fixation method or if, intraoperatively, the decision was made to perform arthroplasty.			stay. The safety outcomes were the incidence of thrombotic events and the mortality rate within 6 weeks after surgery.				
Maniar 2012[299]	• India<br>• English<br>• 2011<br>• Single-Centre<br>• 200<br>• Patients undergoing knee arthroplasty	Known allergy to tranexamic acid; preoperative hepatic or renal dysfunction; serious cardiac or respiratory disease; congenital or acquired coagulopathy; and a history of thromboembolic disease.	• IV TXA (intra-op)<br>• IV TXA (pre-op + intra-op)<br>• IV TXA (intra-op+post-op)<br>• IV TXA (all 3 doses)<br>• IV TXA (local application)<br>• No TXA<br>• -	-	Drain loss and total blood loss. We recorded blood transfusions for quantity and determined the haemoglobin concentration of each transfused unit.	None	Not stated	Unclear	Not stated
Mansouri 2012[300]	• Iran<br>• English<br>• 2012<br>• Single-Centre<br>• 90	(i) Pump time >120 min; and (ii) bleeding with a surgical source (identified at postoperative reoperation).	• IV TXA<br>• Aprotinin<br>• Placebo<br>• Cell salvage	-	The major parameters that we evaluated in this study were as follows: chest-tube drainage, the type and number of units of	None	Not stated	Unclear	Not stated

	• Patients underwent valvular heart surgery (i) age >18 years; (ii) not pregnant; (iii) elective operation; (iv) absence of known or suspected allergy to Aprotinin or tranexamic acid; (v) absence of previous sternotomy, pre-existing renal dysfunction (serum creatinine >1.36 mg/dl), preoperative coagulation defects [prothrombin time (PT) >18 s or activated partial prothrombin time (aPTT) >50 s or platelet count <100 × 109/l], recent (<5 days) ingestion of acetylsalicylic acid, thrombolytic therapy (streptokinase, Urokinase or tissue plasminogen activator <1 day preoperatively), anticoagulant therapy (heparin <4 h preoperatively or warfarin <3 days preoperatively), autologous pre-donation of blood, history of thrombotic events such as deep vein thrombosis, disseminated intravascular coagulation and cerebral thromboembolic accident in the previous 6 months, or unstable angina				blood and blood products transfused, coagulation tests and haemoglobin/haematoc rit and platelet count preoperatively, 6 and 24 h after ICU admission, neurological deficits (drowsiness, agitation, focal neurological deficit, convulsion and coma), renal failure and plasma FDP concentration at the end of surgery. In addition, we assessed demographic items, the number of exchanged heart valves, the length of stay in the ICU bedridden and the hospital mortality.				
Martin 2014[301]	• USA<br>• English<br>• 2012<br>• Single-Centre	Revisions, bilateral joint arthroplasty procedures, known hypersensitivity to TXA or its ingredients, active	• IV TXA<br>• Placebo<br>• Restrictive threshold	the maximum decline in postoperative	the number of patients who received packed red blood cell transfusions, the	None	Not stated	Any	Non profit

	• 100<br>• Patients who underwent total hip and total knee arthroplasty	intravascular clotting disorders, and acute subarachnoid haemorrhage. Patients with a history of DVT or PE		haemoglobin (g/dL)	average length of hospital stay, number of postoperative wound infections, number of patients diagnosed with deep vein thrombosis (DVT) or pulmonary embolism (PE) within 30 days of surgery.				
McConnell 2011[302]	• UK<br>• English<br>• 2008<br>• Single-Centre<br>• 44<br>• Patients who had cemented total hip arthroplasty	If there were contraindications to giving the medications in the study: known allergy to the medications used, including allergy to aspirin; previous reaction to blood products; ethical/religious objection to receiving blood products; or previous thromboembolism	• IV TXA<br>• Placebo<br>• Cell salvage	-	total blood volume	None	Not stated	Unclear	Not stated
Melo 2017[303]	• Brazil<br>• English<br>• 2017<br>• Single-Centre<br>• 42<br>• Patients who underwent primary total hip arthroplasty	Patients younger than 18 years Chronic kidney disease (creatinine clearance less than 60 mL/min m²)<br>Bleeding disorders or thrombophilia;<br>Trauma; Low platelet count (preoperative platelet count less than 150 000) Chronic anaemia (preoperative haemoglobin less than 10 g/dL)<br>Refusal to consent	• IV TXA (low dose<br>• IV TXA (high dose)<br>• No TXA<br>• -	-	The mean blood loss	None	Not stated	Unclear	Not stated
Meng 2019[304]	• China<br>• English<br>• 2013<br>• Single-Centre<br>• 60<br>• patients diagnosed with BPH and undergoing TURP	Preoperative heart and cerebrovascular diseases, renal insufficiency, kidney stones, high risk or a history of thrombosis, long-term anticoagulant therapy, preoperative long-term bed confinement, prostate cancer diagnosis, blood coagulation dysfunction. Patients were also excluded if they had taken 5-a	• IV TXA<br>• Placebo	-	Intraoperative and postoperative bladder irrigation volumes and blood loss volumes	None	Not stated	Unclear	Not stated

		reductase inhibitors, aspirin or warfarin prior to surgery.							
Min 2015[305]	• China<br>• Chinese<br>• 2015<br>• Single-Centre<br>• 64<br>• Patients with primary osteoarthritis undergoing a unilateral total knee arthroplasty	-	• IV TXA<br>• Placebo<br>• -	-	Intraoperative blood loss, postoperative blood loss, postoperative haemoglobin levels, amount of blood transfusion, and number of patients requiring blood transfusion were compared. Fibrinogen, prothrombin time and other coagulation indicators were also examined before operation and 3 hours after operation.	None	Not stated	Unclear	Not stated
Mirmohammadsadeghi 2018[306]	• Iran<br>• English<br>• 2018<br>• Single-Centre<br>• 125<br>• Inclusion criteria were patients undergoing CABG surgery alone, interrupting aspirin 3 days and Plavix at least 5 days before surgery, lack of consuming any other anticoagulant drugs such as heparin or warfarin, lack of coagulation and bleeding disorders, and lack of liver and kidney disease.	Exclusion criteria were complex surgery, emergency surgery, and anticoagulation therapy before surgery, and having haemoglobin lower than 8 g per decilitre before surgery.	• Top TXA<br>• Placebo<br>• -	-	24 and 48 h chest tube drainage, haemoglobin decrease and packed RBC transfusion	None	Not stated	Any	Non profit
Moller 2019[307]	• Denmark<br>• English<br>• 2019<br>• Single-Centre<br>• 58	Potential patients were excluded if they refused RBC transfusion, had previous serious adverse reaction with blood products, had previously	• Restrictive 80g/L<br>• Liberal<br>• POC	mean postoperative Hb day 0–15	(1) units of RBCs transfused<br>(2) randomization rate<br>(3) proportion of patients with protocol	None	Not stated	Unclear	Not stated

	• Patients older than 40 years of age, who were referred for elective open infra-renal AAA repair or lower limb bypass (infra-inguinal arterial bypass surgery or femuro-femoral crossover surgery)<br>• Restrictive threshold 8g/dl	participated in the TV-trial or if they were unable to understand the benefits and risks of participating.			suspensions (4) adherence to haemoglobin concentrations used for transfusion triggers (5) intraoperative tissue oxygenation as determined by NIRS, and (6) severe adverse events within 30 days of surgery				
Molloy 2007[308]	• UK<br>• English<br>• 2005<br>• Single-Centre<br>• 100<br>• Patients who underwent total knee replacement	previous surgery to the knee, with the exception of meniscectomy, bleeding disorders, platelet or bone-marrow disorders, a level of creatinine > 250 µmol/l since this is a contraindication to the administration of tranexamic acid, or a history of thromboembolism.	• IV TXA<br>• No TXA<br>• -	-	Total blood loss. The number of units of blood transfused during the hospital stay was recorded, along with any complications attributed to the surgery or occurring within 90 days of the operation.	None	Not stated	Unclear	Not stated
Motififard 2015[309]	• Iran<br>• English<br>• 2013<br>• Single-Centre<br>• 90<br>• Patients undergoing total knee arthroplasty	Patients with previous history of cerebrovascular disease, thromboembolism, myocardial infarction, and those who were candidates for bilateral TKA	• IV TXA<br>• Placebo<br>• -	Level of Hb 48 hours after surgery.	Hb levels, 6 and 24 hours after surgery, drain output during the first 48 hours after surgery, and blood product administration after surgery and duration of hospitalization.	None	Not stated	Unclear	Not stated
Na 2016[310]	• Korea<br>• English<br>• 2016<br>• Single-Centre<br>• 55<br>• Patients undergoing total hip replacement arthroplasty	Pre- and intra-operative blood transfusion; venous thrombo-embolism; coagulopathy; preoperative haemoglobin of < 10 g/dl; haematological or renal disease; and antiplatelet or anticoagulant medications, including regular and long-term use of nonsteroidal anti-inflammatory drugs within one month of surgery.	• IV TXA<br>• Placebo<br>• POC testing<br>• Restrictive threshold	Results of the ROTEM analyses.	Patients' characteristics; surgery- and anaesthesia related information; laboratory results (haemoglobin, haematocrit, platelets, PT-INR, aPTT and fibrinogen); input (infused volume of crystalloid and colloid); output (intra- and	None	Not stated	None	Not stated

					postoperative blood loss and urine output); and transfusion of blood components.				
Napoli 2016[311]	• Argentina<br>• Spanish<br>• 2016<br>• Single-Centre<br>• 62<br>• Patients who underwent primary hip and knee arthroplasties	-	• IV TXA<br>• Placebo<br>• Restrictive threshold	-	Preoperative and postoperative haematocrit and haemoglobin, days of stay in hospital and number of red cell unit transfusion, complications and adverse effects.	None	Not stated	Unclear	Not stated
Oremus 2014[312]	• Croatia<br>• English<br>• 2014<br>• Single-Centre<br>• 98<br>• Adult patients undergoing primary THA or TKA	1) known hypersensitivity to TXA, 2) history of coagulation abnormalities and thromboembolic disease or current abnormal coagulation test values, 3) history of stroke or acute coronary syndromes within 3 months before surgery, 4) renal failure (serum creatinine > 250 mmol/L [2.83 mg/dL]) or liver cirrhosis, and 5) chronic (ongoing) anticoagulant therapy	• IV TXA<br>• Placebo<br>• Cell salvage	Proportion of patients receiving postoperatively collected autologous drained blood reinfusion and total volume of blood drained within 24 postoperative hours.	Reinfused autologous blood volume, intraoperative blood loss, total external blood loss, and development of Hb and Hct over time (until fourth postoperative day).	None	Not stated	None	Not stated
Ozta 2015[313]	• Turkey<br>• English<br>• 2013<br>• Single-Centre<br>• 60<br>• Patients with unilateral TKR	Patients with inflammatory arthritis, history of thromboembolism, myocardial infarction and stroke and TXA allergy	• IV TXA<br>• No TXA<br>• -	-	Total blood loss and transfusion rate	None	Not stated	None	Not stated
Parker 2013[314]	• UK<br>• English<br>• 2013<br>• Single-Centre<br>• 200<br>• Patients treated at a single centre with a proximal femoral (hip) fracture were considered for inclusion in	Exclusion criteria were age <60 years, patients unwilling or unable to provide written informed consent, multiple trauma (defined as either more than two other fractures), patients treated conservatively, patients treated with percutaneous screw fixation	• Restrictive 80g/L<br>• Liberal<br>• -		Mobility, mental agility, physical status using the American Society of Anaesthesiologists grade	None	Not stated	None	Not stated

	the study if their haemoglobin measured on the first or second day after surgery was between 8.0 and 9.5 g dl1 and no definite symptoms of anaemia were present.<br>• Restrictive threshold symptoms guided	and those with pathological fractures from tumours.							
Pawar 2016[315]	• India<br>• English<br>• 2016<br>• Single-Centre<br>• 80<br>• All males with moderate and severe bladder outlet obstruction with international prostate symptom score of 13 or more and quality of life score of three or more	Patients having neurogenic bladder, prostate carcinoma, previous prostatic surgery, and bladder stones	• IV TXA<br>• No Treatment<br>• -	-	Adverse Reaction<br>Risk & number of RBC transfusion<br>Haemoglobin (Hb), packed cell volume (PCV), and vitals recorded preoperatively, after 30 min of operation and 24 h of operation.	None	Not stated	None	Not stated
Peters 2015[316]	• USA<br>• English<br>• 2012<br>• Single-Centre<br>• 32<br>• Patients undergoing posterior spinal fusion of at least 5 levels for correction of adult spinal deformity	Patients were excluded if they had renal dysfunction identified by elevated blood urea nitrogen and creatinine (Cr) or blood urea nitrogen to Cr ratio greater than 20:1, had religious and/or other beliefs limiting blood transfusion, were using anticoagulant medications, had medical history leading to an abnormal coagulation profile preoperatively, or had significant medical history preventing the use of TXA or EACA described in the protocol or any history of coronary artery disease with stent placement.	• IV TXA<br>• Placebo<br>• Cell salvage	Intraoperative blood loss and total blood transfusion rate.	Postoperative drain output, total blood loss (estimated blood loss [EBL] + wound drainage), and the change in haematocrit (Hct).	None	Not stated	None	Not stated

Prakash 2017[317]	• India<br>• English<br>• 2015<br>• Single-Centre<br>• 100<br>• Patients undergoing primary total knee arthroplasty	All patients with secondary osteoarthritis (rheumatoid and other inflammatory arthritis, post-traumatic arthritis), known allergies to tranexamic acid, major comorbidities, coagulopathies (International Normalised Ratio [INR] > 1.4), previous history of stroke or severe ischaemic cardiopathy and patients undergoing bilateral total knee arthroplasty.	• IV TXA<br>• No TXA<br>• -	-	Post-operative blood loss, Requirement of blood transfusion, Requirement of blood transfusion	None	Not stated	None	Not stated
Prasad 2018[318]	• India<br>• English<br>• 2018<br>• Single-Centre<br>• 60<br>• American Society of Anaesthesiologist's classification physical status 1 and 2 patients, both males and females, electively posted for open abdominal tumour surgery in the department of surgical oncology were included as study population.	Patients with a history of bleeding diathesis, pulmonary embolism or deep vein thrombosis, those posted for hepatic resection or liver surgery, those posted for laparoscopic tumour removal, and those with a known allergy to tranexamic acid were excluded from the study.	• IV TXA+Placebo<br>• IV TXA + IV TXA<br>• Placebo<br>• -	Intraoperative blood loss	Total volume of intravenous fluids infused and whole blood units or blood products transfused were noted. Total duration of surgery in minutes (from skin incision to skin closure) was noted.	None	Not stated	None	Not stated
Raviraj 2012[319]	• India<br>• English<br>• 2012<br>• Single-Centre<br>• 175<br>• Patients undergoing simultaneous bilateral total knee arthroplasty	Patients with bleeding or clotting disorders, those on preoperative anticoagulation therapy, abnormal coagulation profile, rheumatoid arthritis, renal disorders or insufficiency, sickle cell disease, patients allergic to local anaesthetics/tranexamic acid.	• IV TXA<br>• Placebo<br>• -	-	Haemoglobin levels were measured on postoperative day 1 and day 2, and the difference between the preoperative levels and lowest postoperative level was taken as the drop in haemoglobin level. The number of units of packed red blood cells received in	None	Not stated	None	Not stated

					each group was documented.				
Roy 2012[320]	• India<br>• English<br>• 2012<br>• Single-Centre<br>• 50<br>• Patients undergoing primary unilateral total knee arthroplasty	Patients with known allergy to tranexamic acid, severe anaemia (Hb %< 9 gm/dl), hepatic/cardio-respiratory/renal insufficiency, congenital or acquired coagulopathy and recent history of thromboembolic episode. Patients with severe deformity (> than 20 deg varus and flexion) and restricted range of motion (<90 deg) were also excluded	• IV TXA<br>• Placebo<br>• -	-	Total blood loss and transfusion requirements	None	Not stated	Unclear	Not stated
Sabry 2018[321]	• Egypt<br>• English<br>• 2017<br>• Single-Centre<br>• 70<br>• Patients who underwent decortication surgery for chronic thoracic empyema, encysted effusion, or clotted hemothorax on the elective way.	Patients who required lung resection, reopening due to surgical bleeding, patients requiring anticoagulant postoperatively for fear of deep vein thrombosis, patients with renal failure, patients with liver cirrhosis, primary blood disease such as haemophilia or else, know allergy to tranexamic acid, and pregnant female patients.	• Top TXA<br>• Placebo<br>• -	-	Total drainage and postoperative blood transfusion	None	Not stated	None	Not stated
Sadeghi 2007[322]	• Iran<br>• English<br>• 2005<br>• Single-Centre<br>• 67<br>• Patients with a diagnosis of fracture of the hip<br>• necessitating hip surgery	Patients with un-displaced subcapital fractures treated by pinning that have been shown to be fractures with low level loss of blood. Patients with preoperative haemoglobin less than 10 g/L., platelets count less than 100×10^9/l of blood, a known coagulopathies disorders, renal insufficiency (creatinine > 2 mg/dL), advanced hepatic dysfunction, and history of thromboemboli were also excluded.	• PO TXA<br>• Placebo<br>• -	-	Blood loss during surgery, Transfusions	None	Not stated	Unclear	Not stated

Sa-Ngasoongsong 2013[323]	• Thailand<br>• UK<br>• 2011<br>• Single-Centre<br>• 135<br>• patients undergoing conventional TKR	(1) no risk of abnormal bleeding tendency or bleeding disorder (normal coagulogram, serum creatinine < 2.0 mg/dL, stop nonsteroidal anti-inflammatory drugs and antiplatelet drugs more than 7 days; and (2) no contra-indication for TXA use (no active intravascular clotting process, no acquired defective colour vision, no subarachnoid haemorrhage, no hypersensitivity to TXA, and no any of history of serious adverse effects, thrombotic disorder and haematuria).	• IV TXA (high dose)<br>• IV TXA (low dose)<br>• Placebo<br>• -	-	Blood transfusion requirement was measured by recording the number of patients receiving transfusion and amount of blood transfusion in unit. Functional outcomes, such as KSK and WOMAC score, were evaluated at the clinic at 3-month, 6-month and 1-year period postoperatively. Postoperative complications such as wound hematoma, surgical site infection or systemic infection were evaluated at ward, at clinic as time of follow-up and/or by phone interview periodically.	None	Not stated	Unclear	Not stated
Sarzaeem 2014[324]	• Iran<br>• English<br>• 2012<br>• Single-Centre<br>• 200<br>• Patients with age over 18 years with planned TKA due to degenerative arthritis	Patients with any cardiovascular problems (such as myocardial infarction, atrial fibrillation, angina), cerebrovascular conditions (such as previous stroke or previous vascular surgery) and thromboembolic disorders	• IV TXA<br>• IA TXA<br>• Top TXA<br>• No TXA<br>• -	-	The amount of drainage was recorded in order to estimate the postoperative blood loss. Transfusion data.	None	Not stated	None	Not stated
Schiavone 2018[325]	• Italy<br>• English<br>• 2015<br>• Single-Centre<br>• 90<br>• Patients suffering from pertrochanteric fractures surgically treated with	Polytrauma, patients operated more than 48 hours after the traumatic event; refusal of consent to participate in the study; dementia; patients whose relatives have not given their consent to participate; oral anticoagulant therapy; contraindications to treatment	• Top TXA<br>• Placebo<br>• -	proportion of patients receiving at least 1 U of allogenic RBC transfusion according to transfusion protocol.	-	None	Not stated	None	Not stated

	osteosynthesis with SupernailGT	with tranexamic acid (a history of prior venous or arterial thrombosis, brain stroke, patients with creatinine clearance below 30 ml/min); patients who were administered tranexamic acid during or at the end of surgery; patients who require one or more transfusions before surgery; patients with INR> 1.2; patients with hematological diseases; patients who had the intra-operative complication of the migration of the intra-pelvic wire guide							
Scrascia 2012[326]	• Italy<br>• English<br>• 2012<br>• Single-Centre<br>• 34<br>• Patients undergoing first-time, elective, isolated CABG	Patients aged >80 years old, preoperative haemoglobin (Hb) <12 g/dL, body surface area (BSA) <1.7 m2, redo or emergency surgery, valvular, thoracic aorta or combined procedures, liver insufficiency (Child Pugh B or C class), platelet count below 50,000 or antiplatelet treatment taken within 5 days before surgery, pre-existing haemolytic or haemostatic disorders, anticoagulant treatment, inflammatory disorders or steroids treatment.	• Cell Salvage<br>• Normal Drainage<br>• Tranexamic acid	The influence of CPB circuit residual blood salvage infusion after cell saving treatment on inflammatory, coagulative and fibrinolytic system activation, measuring specific parameters.	The influence of pump blood salvage on postoperative haemoglobin levels and transfusion rate.	None	Not stated	None	Not stated
Seol 2016[327]	• Korea<br>• English<br>• 2016<br>• Single-Centre<br>• 100<br>• TKA patients	Patients with secondary osteoarthritis (e.g., rheumatoid arthritis, posttraumatic osteoarthritis, gouty arthritis), a cardiovascular problem (e.g., myocardial infarction, atrial fibrillation, angina, heart failure), simultaneous bilateral TKA, a history of	• IV TXA<br>• Placebo<br>• -	-	The total volume of drained blood and the decrease in haemoglobin at 6 hours, 24 hours, 48 hours and 5 days postoperatively were recorded. Blood transfusions were	None	Not stated	Unclear	Not stated

		thromboembolic disease, bleeding disorder, known allergy to tranexamic acid, and lifelong warfarin therapy for thromboembolism prophylaxis			recorded as the number of units of packed erythrocytes.				
Serrano-Trenas 2011[328]	• Spain<br>• English<br>• 2008<br>• Single-Centre<br>• 200<br>• Patients aged over 65 undergoing hip fracture surgery at the Orthopaedic and Trauma Surgery Unit of the Hospital Reina Sofia in Córdoba (Spain) between October 2006 and October 2008	Patients with diseases diagnosed before the admission of patient (iron overload disorders, hypersensitivity to oral or parenteral iron preparations, asthma or other severe atopic, active infection or neoplasm), treatment with Clopidogrel or with acetylsalicylic acid at dose rates greater than 150 mg/24 hr, no surgical indication for the current fracture, disorders impaired coagulation (partial thromboplastin time > 2.5%, international normalized ratio > 1.5), liver disorders with elevated transaminases (aspartase aminotransferase [AST] > 70 U/L, alanine aminotransferase [ALT] > 55 U/L), and chronic kidney failure (creatinine > 2 mg/dL) or patients including in dialysis.	• IV Fe<br>• No treatment	30-day mortality	Functional Recovery<br>Sepsis<br>Hospital LOS<br>Risk & number of RBC transfusion<br>Risk of receiving non red cell component	None	Not stated	None	Not stated
Seviciu 2016[329]	• USA<br>• English<br>• 2016<br>• Single-Centre<br>• 121<br>• Patients over 18 years of age undergoing elective total primary knee arthroplasty, under spinal anaesthesia	Patients with adverse reaction to TXA; congenital or acquired coagulation disorder; preoperative platelet count <100,000/mL or international normalized ratio >1.4; history of DVT, PE, or CVA; acquired defective colour vision; renal insufficiency (glomerular filtration rate <20 mL/min); severe liver disease; coronary stents; or pregnant patients	• IV TXA<br>• IV TXA+BSS<br>• BSS only<br>• Placebo<br>• -	The change in Hb at day 3	change in haematocrit and estimated blood loss.	None	Not stated	Unclear	Not stated

Shakeri 2018[330]	• Iran<br>• English<br>• 2018<br>• Single-Centre<br>• 50<br>• Patients who had either lumbar spinal stenosis or lumbar spondylolisthesis and were candidates for 2 or more than 2 levels of laminectomy and posterolateral fusion performed with instruments (pedicle screw and rods).	Patients with a history of treatment with anticoagulant drugs, dipyridamole and oral contraceptives, those with abnormal international normalized ratio, prothrombin time and partial thromboplastin time, patients with cerebrovascular accident, myocardial infarction, coagulopathies, traumatic brain injury, cardiopulmonary resuscitation, renal failure, smoking, opioids, diabetes mellitus, hypertension, coronary artery disease, pregnant and breastfeeding women, and those who received packed cell transfusion during or after operation	• IV TXA<br>• Placebo<br>• -	-	The two groups were compared with respect to age, sex, weight, body mass index (BMI), bleeding in the operation room, total volume of bleeding, bleeding volume in the first 12 hours after surgery, volume of bleeding between 12–24 hours after surgery, packed cells received, and hospitalization time.	None	Not stated	Unclear	Not stated
Shen 2015[331]	• China<br>• English<br>• 2013<br>• Single-Centre<br>• 81<br>• 1) Primary knee osteoarthritis and (2) unilateral TKA.	(1) inflammatory or autoimmune diseases; (2) blood coagulation disorders; (3) history of thromboembolic disease; (4) severe anaemia; (5) peripheral neuropathy; (6) malignant tumour; (7) TXA or low molecular heparin contraindication; (8) pre-operative anticoagulant drug use; and (9) those who did not cooperate in the experiment.	• IV TXA<br>• Placebo<br>• -	-	The following data were obtained: (1) height, and weight, and body mass index; (2) intraoperative blood loss, i.e., the liquid of the drainage bottle minus the intraoperative flushing fluid plus the net increase in gauze; (3) post-operative drainage amount at 12 h and total drainage amount; (4) Hgb, Hct, PLT, D-dimer, total blood loss, and hidden blood loss which was calculated according to Sehat-design mathematical	None	Not stated	Unclear	Not stated

					methods [9], pre-operative and post-operative levels of Hgb, Hct, and PLT at 1, 3, and 5 days, and pre-operative and post-operative 24-h D-dimer values; and (5) DVT.				
Shen 2016[332]	• China<br>• English<br>• 2016<br>• Single-Centre<br>• 103<br>• High bleeding risk undergoing cardiac surgery with CPB	Emergency cardiac surgery with CPB<br>The first time single valve replacement	• Intra+Post Cell Salvage<br>• Normal Drainage<br>• Tranexamic acid<br>• POC testing<br>• Restrictive threshold	the incidence of impairment of blood coagulation during perioperative period (peri-op)	the incidence of adverse events during postoperative period (post-op)	None	Not stated	None	Not stated
Shi 2013a[333]	• China<br>• English<br>• 2013<br>• Multi-Centre<br>• 552<br>• Patients eligible for randomization were 1173 men and women aged 18 to 85 years undergoing primary and isolated on-pump CABG	Previous cardiac surgery, haematocrit level less than 33%, platelet count less than 100 000 x 10^3/uL, allergy to tranexamic acid, and being recruited in other studies.	• IV TXA<br>• Placebo<br>• -	blood loss, major bleeding, and red blood cell (RBC) transfusion volume and exposure.	Major morbidity and mortality. Major morbidity was defined as permanent disability caused by stroke, postoperative myocardial infarction, renal failure, and respiratory failure.	None	Not stated	Any	Non profit
Shi 2013b[334]	• China<br>• English<br>• 2013<br>• Single-Centre<br>• 117<br>• Patients receiving on-pump coronary artery bypass grafting without clopidogrel and aspirin cessation	Previous cardiac surgery, haematocrit less than 33%, platelet count less than 100,000/mL, or allergy to tranexamic acid, and those recruited in other studies.	• IV TXA<br>• Placebo<br>• -	Volume of allogeneic erythrocyte transfused perioperatively.	-	None	Not stated	Any	Non profit
Shi 2017[335]	• China<br>• English<br>• 2016	(1) Allergy to TA. (2) History of bleeding disorders or thromboembolic events. (3) Severe cardiac or respiratory	• IV TXA<br>• Placebo<br>• -	Intraoperative estimated blood loss and total blood loss.	Packed red blood cells received and postoperative	None	Not stated	Any	Non profit

	• Single-Centre<br>• 100<br>• (1) Patients with lumbar spinal stenosis or lumbar spondylolisthesis who were scheduled to undergo posterior lumbar decompression interbody fusion; the conservative therapy had failed. (2) Patients aged 18 to 80 years. (3) Patients who provided written informed consent.	disease and renal or hepatic dysfunction. (4) Platelet count <150,000/mm³. (5) Preoperative Hb <10 g/dL. (6) Uncontrolled hypertension; high blood pressure (BP >160/90 mm Hg). (7) ASA physical status >III. (8) Intake of nonsteroidal anti-inflammatory drugs within 7 days before surgery. (9) Pregnancy.			haemoglobin and haematocrit levels.				
Shinde 2015[336]	• India<br>• English<br>• 2015<br>• Single-Centre<br>• 56<br>• Patients of Indian origin undergoing TKA for primary osteoarthritis of the knee joint	Allergy to TEA, rheumatoid arthritis, revision total knee arthroplasty, coagulopathy (preoperative platelet count ≤150000/mm³, BT, PT, CT abnormality), previous history of thromboembolic disease (cerebrovascular accident, deep vein thrombosis, myocardial infarction), severe ischemic heart disease, NYHA class 3 and 4, serum creatinine >1.5 mg/dL, severe pulmonary disease, e.g. FEV1 ≤50% normal, hepatic failure and preoperative anaemia (Hb <10 g/dL).	• IV TXA<br>• Placebo<br>• -	-	Blood loss, blood transfusion requirements.	None	Not stated	None	Not stated
Song 2017[337]	• Korea<br>• English<br>• 2015<br>• Single-Centre<br>• 200<br>• Patients undergoing primary navigated TKA	patients with secondary osteoarthritis (rheumatoid and other inflammatory arthritis, posttraumatic arthritis), known allergies to TXA, major comorbidities (American Society of Anaesthesiology (ASA) grade 4 and above), coagulopathies (INR >1.4), history of previous deep vein thrombosis (DVT) or patients	• IV TXA<br>• Top TXA<br>• Combined<br>• Placebo<br>• -	-	Evident loss through drain, total loss based on Gross method and haemoglobin balance method, hidden losses, haemoglobin and haematocrit drop, functional scores, and all possible complications related to TXA.	None	Not stated	None	Not stated

		on antithrombotic treatment, previous history of stroke or severe ischemic cardiopathy, and patients undergoing bilateral total knee arthroplasty							
So-Osman 2014[338]	• Germany<br>• English<br>• 2014<br>• Single-Centre<br>• 1759<br>• Adult elective hip-and knee surgery patients	Hb (haemoglobin) less than 13 g/dl, untreated hypertension (diastolic blood pressure >95 mmHg); a serious disorder of the coronary, peripheral, and/or carotid arteries; a recent myocardial infarction or stroke (within 6 months); sickle cell anaemia; a malignancy in the surgical area; a contraindication for anticoagulation prophylaxis; an infected wound bed; a revision of an infected prosthesis, which was being treated with local antibiotics difficulty understanding the Dutch language (unable to give informed consent); or were pregnant or refused homologous blood transfusions.	• Intra+Post Cell Salvage<br>• Normal Drainage<br>• Restrictive threshold	RBC use	Cost effectiveness, in which length of hospital stay was included.	None	Not stated	Any	Blood service
Spitler 2019[339]	• USA<br>• English<br>• 2019<br>• Single-Centre<br>• 93<br>• Patients with fractures of the pelvic ring, acetabulum, and proximal femur.	-	• IV TXA<br>• No TXA<br>• Cell Salvage	Transfusion rates and total blood loss (TBL)		None	Not stated	Any	Non profit
Sudprasert [340]	• Thailand<br>• English	Renal insufficiency History of thromboembolic events (e.g.,	• Top TXA<br>• Placebo	Requirement for PRC transfusion	Total drainage volume, time to drain removal,	None	Not stated	Unclear	Not stated

	• 2016<br>• Single-Centre<br>• 57<br>• Men and women, 18 to 70 years of age with injuries involving the thoracic or lumbar spine (Thoracolumbar Injury Classification and Severity score ≥5) undergoing long-segment instrumented posterior spinal fusion with local autologous bone graft No neurological deficits American Society of Anesthesiologists physical status class I, II, or III	pulmonary embolism, embolic stroke, and deep venous thrombosis) History of significant cardiovascular diseases (e.g., unstable angina, recent myocardial infarction, significant arrhythmia, and uncontrolled hypertension) History of acquired defective colour vision Coagulation disorder Gross haematuria or microhematuria Displaced laminar fracture on computed tomography axial section that might be associated with dural tears Allergy to tranexamic acid Take aspirin or nonsteroidal anti-inflammatory drugs within a week before randomization and during the hospitalization		postoperatively prior to discharge home.	and duration of postoperative hospitalization.				
Sun 2017[341]	• China<br>• English<br>• 2017<br>• Single-Centre<br>• 180<br>• Patients who were scheduled to undergo primary unilateral TKA	Allergy to TA, anaemia, severe cardiopulmonary disease, and refusal of blood products and those complicated with haematological or thromboembolism disease	• IV TXA (High dose)<br>• IV TXA (Medium dose)<br>• IV TXA (Low dose)<br>• No TXA<br>• -	Postoperative blood transfusion	The blood loss including intraoperative blood loss (fluid volume in intraoperative drainage bottle _ rinse solution volume) and postoperative blood loss (the drainage volume for 48 hours postoperatively)	None	Not stated	Unclear	Not stated
Taghaddomi 2009a[342]	• Iran<br>• English<br>• 2009<br>• Single-Centre<br>• 80<br>• Patients undergoing lumbar hernial disc resection	History of bleeding disorder, chronic renal insufficiency (serum creatinine>2 mg/dL), perioperative anaemia (Hb<10 gr/dL), and warfarin medication	• Total intravenous +TXA<br>• Total intravenous - TXA<br>• Inhalation Anaesthetic +TXA<br>• Inhalation Anaesthetic - TXA	-	The patients characteristics and intraoperative variables including the amount of blood loss, duration of the surgery, hemodynamic changes, the time of awareness, duration of recovery period were collected	None	Not stated	Any	Non profit

			• -						
Taksaudom 2017[343]	• Thailand<br>• English<br>• 2015<br>• Single-Centre<br>• 80<br>• Patients who underwent elective on-pump cardiac surgery	Re-sternotomy procedure, emergency or urgent cases, bleeding diathesis (haemophilia or platelet count<10010^9/L, preoperative coagulopathy), renal failure (creatinine level>2.0 mg/dL), history of TA allergy, discontinuation of antiplatelet medication less than 7 days before surgery, heparin infusion within 24 h before surgery, aortic surgery, and complex adult congenital heart disease.	• Top TXA<br>• Placebo<br>• -	24-h blood loss	The volume of blood products transfused, re-exploration rate, length of hospital stay, mortality, morbidity, and TA-related complications.	None	Not stated	None	Not stated
Tang 2018[344]	• China<br>• English<br>• 2015<br>• Single-Centre<br>• 587<br>• Patients were diagnosed with elbow stiffness by Kay classification; patients diagnosed with heterotopic ossification of bone; (3) patients without skin sensibility aging from 45 to 81 years old; (4) patients without surgical contraindication	Patients with muscle atrophy, nerve damage or poor postoperative recovery; patients with severe primary diseases, mental disease, severe skin diseases or other complications affects elbow joint; (3) patients with a joint instability; (4) clinical trial subjects who didn't respond well to treatment or had other reasons	• IV TXA<br>• No TXA<br>• -	-	Postoperative haemorrhage and complications	None	Not stated	Any	Non profit
Tavares Sanchez 2018[345]	• Spain<br>• Spanish<br>• 2015<br>• Single-Centre<br>• 119<br>• Patients undergoing cementless total hip arthroplasty	Patients who were allergic to tranexamic acid (Amchafibrin) or any of its components, who had experienced adverse reactions previously after administration of the drug and when the reason for surgery was an acute fracture (admitted via the emergency	• Top TXA<br>• Placebo<br>• -	-	Bleeding, transfusion requirements and length of stay, and describe the complications	None	Not stated	Unclear	Not stated

		department) were excluded from the study.							
Thipparampall 2017[346]	• India<br>• English<br>• 2017<br>• Single-Centre<br>• 59<br>• Patients undergoing hip surgeries	Patients with a history of severe ischaemic heart disease, pulmonary embolism, deep vein thrombosis (DVT), hepatic or renal failure or allergy to TA were excluded from the study.	• IV TXA (bolus)<br>• IV TXA (bolus+infusion)<br>• Placebo<br>• -	Intraoperative blood loss	Need for transfusions. Hb and haematocrit values were recorded at 6 h after surgery, on the morning of post-operative day 1 and 2. Patients were monitored clinically for evidence of DVT twice daily.	None	Not stated	None	Not stated
Tian 2018[347]	• China<br>• English<br>• 2017<br>• Single-Centre<br>• 100<br>• patients of intertrochanteric fractures, underwent with proximal femoral nail anti-rotation	(1) pathological fracture; (2) allergy to TXA; (3) Serious cardiac or respiratory disease; (4) congenital or acquired coagulopathy; (5) history of thromboembolic disease such as cerebral infarction, pulmonary embolism, myocardial infarction, or deep vein thrombosis; (6) recent thrombophilia; (7) preoperative hepatic or renal dysfunction (male creatinine level >115 mmol/L, female creatinine level >100 mmol/L); and (8) diabetic.	• IV TXA<br>• No TXA<br>• -	-	Volume of intraoperative blood loss and postoperative drainage, and the need for postoperative blood transfusion and transfusion volume for all patients.	None	Not stated	Unclear	Not stated
Triyudanto 2016[348]	• Indonesia<br>• English<br>• 2016<br>• Single-Centre<br>• 22<br>• Patients having TKR	Patients who consumed anticoagulant and anti-thrombocyte aggregation, had preoperative Hb ≤10.5 g/dl for man and woman, had intraoperative blood loss ≥500 cc, with mental illness, had uncontrolled diabetes mellitus (DM), rheumatoid arthritis, malignancy, and immunosuppression, had infected knee, had abnormal prothrombin time (PT) and	• IV TXA<br>• IA TXA<br>• Placebo<br>• -	Postoperative bleeding	Number of RBC transfusion<br>Perioperative blood loss	None	Not stated	Unclear	Not stated

		activated partial thromboplastin test (APTT)							
Tzatzairis 2016[349]	• Greece<br>• English<br>• 2015<br>• Single-Centre<br>• 120<br>• Patients with a diagnosis of primary osteoarthritis undergoing unilateral TKR without tourniquet	Allergy and/or hypersensitivity to TXA; subarachnoid haemorrhage; a known history of thromboembolic disease, cardiovascular disease (a history of myocardial angina or infarction); coronary or vascular stent placed within the past 12 months; preoperative renal or hepatic dysfunction; cerebral vascular disease (a history of stroke); preoperative coagulopathy (a platelet [PLT] count <150,000/mm3 or an international normalized ratio greater than 1.4; retinal vein or artery occlusion	• IV TXA<br>• Top TXA<br>• No TXA<br>• -	calculated blood loss, the transfusion rate, and quantity of allogeneic blood units	Complications such as DVT, pulmonary embolism, superficial and deep infections, and any deterioration of hepatic or renal function.	None	Not stated	None	Not stated
Vijay 2013[350]	• India<br>• English<br>• 2013<br>• Single-Centre<br>• 90<br>• Patients undergoing hip fracture surgery	Patients with chronic disease like Rheumatoid arthritis, ischemic heart disease, malignancy, history of any previous thromboembolic episodes, haemoglobin <8 g/dl were excluded from the study.	• IV TXA<br>• Placebo<br>• Cell salvage	-	Postoperative bleeding (volume of blood in the drain), percentage fall of haemoglobin, transfusions and complications were recorded	None	Not stated	None	Not stated
Volquind 2016[351]	• Brazil<br>• English<br>• 2013<br>• Single-Centre<br>• 62<br>• Patients undergoing primary total knee replacement	Patient's refusal to participate in the study, allergies to drugs used, changes related to coagulation, use of nonsteroidal anti-inflammatory or antiplatelet drugs seven days before surgery, kidney or liver failure, pregnancy, and previous history of deep venous thrombosis or pulmonary embolism	• IV TXA<br>• Placebo<br>• -	-	Haemoglobin, haematocrit, and blood loss were recorded 24 h after surgery. Deep vein thrombosis was investigated during patient's hospitalization and 15 and 30 days after surgery in review visits.	None	Not stated	Unclear	Not stated
Wang 2012[352]	• China<br>• English<br>• 2012	Known allergy to the study drug, history of bleeding	• IV TXA<br>• No TXA<br>• POC testing	-	Postoperative bleeding and transfusion requirements	None	Not stated	Any	Non profit

	• Single-Centre<br>• 231<br>• Patients scheduled for elective OPCAB	disorders, preoperative anaemia (haemoglobin [Hb] <10 g/dL), chronic renal insufficiency (serum creatinine >2 mg/dL), active chronic hepatitis or cirrhosis, previous cardiac surgery, myocardial infarction < 30 days, and withdrawal of clopidogrel or aspirin <5 days before surgery.							
Wang 2013[353]	• China<br>• English<br>• 2013<br>• Single-Centre<br>• 60<br>• Patients with degenerative lumbar instability with stenosis	Patients with chronic renal failure, cirrhosis of the liver, serious cardiac disease, allergy to TXA, thromboembolic disease, bleeding disorders, hyper coagulation status, disseminated intravascular coagulation, and those who were receiving antiplatelet and/or anticoagulant drugs at the time of the study	• IV TXA<br>• Placebo<br>• Restrictive threshold	-	Intraoperative and postoperative blood loss	None	Not stated	Unclear	Not stated
Wang 2015a[354]	• China<br>• English<br>• 2015<br>• Single-Centre<br>• 60<br>• patients treated with unilateral primary cement TKA	Patients with a body mass index (BMI) < 35 kg/m2, rheumatoid arthritis, simultaneous bilateral TKA, allergy to TXA, preoperative anaemia (haemoglobin [Hb] value of <11 g/dL in females and <12 g/dL in males), refusal of allogeneic blood products, or a history of coagulopathy or a thromboembolic event	• Top TXA<br>• Placebo<br>• -	Total blood loss, transfusion rate, and the number of blood units transfused.	Coagulation-fibrinolysis markers, including prothrombin time (PT), activated partial thromboplastin time (APTT), thrombin time (TT), platelet numbers (PLT), fibrinogen (FIB) and D-dimer levels recorded on PODs 1, 3, and 5. The wound healing condition (skin necrosis, hematoma, infection) was monitored the patients discharged.	None	Not stated	Unclear	Not stated
Wang 2015b[355]	• China<br>• English<br>• 2014<br>• Single-Centre	Patients with preoperative anaemia or coagulopathy; patients with infectious active diseases like lower limb infection or systemic infection	• Top TXA<br>• Placebo<br>• -	-	Postoperative haemoglobin, blood coagulation index, total blood loss volume, drainage volume, blood	None	Not stated	Any	Non profit

	• 100<br>• Patients underwent primary unilateral TKA	disease; patients with TXA contraindications; patients with a history of venous thromboembolic disease or thromboembolic disorders; patients with clotting problem like liver tumour or cirrhosis; patients intended to participate in autologous blood transfusion; incompatibility patients.			transfusion rate and lower extremity deep vein thrombosis (DVT) rate				
Wang 2015c[356]	• China<br>• Chinese<br>• 2015<br>• Single-Centre<br>• 69<br>• Patients who received bilateral total knee arthroplasty	-	• IV TXA<br>• Placebo<br>• -	-	Total blood loss, intraoperative blood loss, the hidden blood loss, amount of postoperative drainage, the ratio of blood transfusion, hemoglobin, D-dimer, prothrombin time and activated partial thromboplastin time	None	Not stated	Unclear	Not stated
Wang 2016[357]	• China<br>• English<br>• 2014<br>• Single-Centre<br>• 80<br>• Patients scheduled for THA	History of any of the following: haemophilia, deep vein thrombosis, pulmonary embolism, stents, ischemic heart disease, anticoagulant medication, serious liver or renal dysfunction, or allergy to tranexamic acid.	• IV TXA<br>• Placebo<br>• -	proportions of patients in each group (a) requiring blood transfusion, (b) experiencing deep vein thrombosis (DVT) or (c) experiencing pulmonary embolism (PE).	Total blood loss, drained blood loss, decrease in haemoglobin and haematocrit as well as other complications.	None	Not stated	Any	Non profit
Wang 2017a[358]	• Taiwan<br>• English<br>• 2015<br>• Single-Centre<br>• 198<br>• Primary unilateral minimally invasive TKA	Patients who had a coagulopathy, severe renal impairment (creatinine clearance, <30 mL/min), concomitant use of protease inhibitors of human immunodeficiency virus, or fibrinolytic agents that contraindicated the use of	• IV TXA<br>• Placebo<br>• -	-	Total blood loss was calculated from the maximum haemoglobin drop after surgery plus amount of transfusion. The transfusion rate and wound complications were recorded in all patients.	None	Not stated	Any	Non profit

		rivaroxaban, prior surgery on the affected knee, a history of thromboembolic disease requiring life-long anticoagulant therapy or antiplatelet drugs that could not be stopped before operation, previous allergic history to TXA, or contrast medium for radiographic examination or a preoperative Hb level less than 10 g/dL							
Wang 2017b[359]	• Taiwan<br>• English<br>• 2017<br>• Single-Centre<br>• 150<br>• Patients aged 30 years and older, who were scheduled for a primary unilateral TKA for end-stage osteoarthritis	1. Patients with preoperative Hb <110 g/L. 2. Patients with thromboembolic history or preoperative situation like DVT or PE, or arterial stenosis with or without concomitant coronary artery bypass grafting. 3. Patients with preoperative D-dimer >3 times normal level. 4. Patients with cardiovascular history, such as myocardial infraction, angina, or atrial fibrillation. 5. Patients with cerebrovascular history of previous stroke. 6. Patients with clotting disorders including prolonged prothrombin time or activated partial thromboplastin time, or abnormal international normalized ratio. 7. Patients with allergic history of TXA. 8. Pregnant or lactating women, drug abusers or alcoholics. 9. Patient with severe complications, such as severe liver and kidney diseases, New York Heart Association class III or above, heart failure, or patients with severe infection.	• IV TXA<br>• Placebo<br>• -	-	The amount of total and hidden blood loss (HBL), drainage, transfusion, changes in haemoglobin levels, and complications were recorded.	None	Not stated	Any	Non profit

		10. Patients combined the use of other medicine that may have an impact on the outcome of the study. 11. Patients diagnosed as inflammatory arthritis including rheumatoid arthritis, pigmented villonodular synovitis, and so on.							
Wang 2019[360]	• China<br>• English<br>• 2018<br>• Single-Centre<br>• 300<br>• all patients (age > 18 years) with hip osteoarthritis or osteonecrosis of the femoral head, scheduled for elective, unilateral, primary THA, were consecutively screened	known allergy to TXA; a haemoglobin (Hb) level of < 11 g/dL; a history of arrhythmia, pulmonary embolism (PE), deep venous thrombosis (DVT) or severe ischaemic heart disease; an acquired or congenital coagulopathy; previous vascular or cardiac bypass surgery; a history of high-risk medical co-morbidities (severe renal insufficiency, hepatic failure or severe pulmonary disease); current full dose anticoagulant therapy (warfarin or heparin) within 1 week; refusal of blood products or participation; or participation in another clinical trial during the last year.	• Placebo<br>• PO TXA (3g+3g Placebo)<br>• PO TXA (4g + 2g Placebo)<br>• PO TXA (5g+1g Placebo)<br>• PO TXA (6g)<br>• Restrictive threshold	Total blood loss on POD 3.	Hb drops on POD 1 and 3, total blood loss on POD 1, intra-operative blood loss, allogeneic red cell transfusion rates, the number of blood units transfused, the length of hospital stay, the post-operative changes in joint function (i.e. the range of motion [ROM] and the severity of hip pain at rest and with movement based on visual analogue scale [0, no pain, and 100, worst pain imaginable] on POD 1, 2 and 3) and Harris Hip Score (HHS) at discharge.	None	Not stated	Unclear	Not stated
Wei 2014[361]	• China<br>• English<br>• 2014<br>• Single-Centre<br>• 201<br>• 1. Age 45–80 years 2. Preoperative haemoglobin values N11 g/dl 3. Normal international normalized ratio (INR), prothrombin time (PT), partial	1. Had a documented history of thrombo-embolism 2. Had an allergy to TXA 3. Had a high risk of venous thrombosis for intravenous use of TXA according to the American Academy of Orthopaedic Surgeons Guideline	• IV+Top TXA<br>• Placebo<br>• -	the nadir in-patient Hct, maximum Hct drop from preoperative levels, length of hospital stay, transfusion rates, wound complications and total blood loss (TBL)	-	None	Not stated	Any	Non profit

	thromboplastin time (PTT) values 4. Consented to undergo unilateral cementless THA 5. Had no history of previous hip surgery								
Wiefferink 2007[362]	• Netherlands<br>• English<br>• 2007<br>• Single-Centre<br>• 30<br>• Adult patients, undergoing isolated primary elective myocardial re-vascularization	Not stated	• Post Cell Salvage<br>• Control<br>• -	-	the volume of the chest tube drainage was noted 2 hours after arrival at the ICU, and the transfusion requirements were noted during the entire ICU period.	None	Not stated	Unclear	Not stated
Xie 2015a[363]	• China<br>• English<br>• 2015<br>• Single-Centre<br>• 141<br>• 3 inclusion criteria that should be satisfied at the same time: firstly, patients were scheduled for cardiac surgery with CPB; secondly, surgery was combined aortic valve replacement and mitral valve replacement, or Bentall, or reoperation; thirdly, at least two of the following conditions are satisfied: age >70 years; body surface area (BSA)<1.6 m2; renal dysfunction (creatinine >15mg/L); liver insufficiency (Child -Pugh B or C); coagulation disorders (thromboelastography, TEG, R value before surgery >10 min); haemoglobin(HB)	Not stated	• Intra+Post Cell Salvage<br>• Normal Drainage<br>• Tranexamic acid<br>• POC testing<br>• Restrictive Threshold	-	perioperative allogeneic red blood cell (RBC) transfusion, perioperative impairment of blood coagulative function, postoperative adverse events and costs of transfusion-related.	None	Not stated	None	Not stated

	levels < 130 g L-1 in males or <120 g L-1 in females; Platelets (PLT) count <50 ×10^9 L-1; intake of aspirin 3 days before surgery or Clopidogrel 7 days before surgery								
Xie 2015b[364]	• China<br>• English<br>• 2012<br>• Single-Centre<br>• 90<br>• Age 18 to 65 years, the presence of a unilateral closed calcaneal fracture, type II or type III, according to Sanders classification (14), and the absence of chronic disease (e.g., hypertension, hypercholesterolemia, and diabetes mellitus) or the presence of well controlled chronic illness	Patients with bilateral calcaneal fractures or other injuries, a known coagulopathy disorder, renal insufficiency, hepatic dysfunction, serious cardiac disease, an allergy to TXA, or receiving antiplatelet and/or anticoagulant drugs at the time of the study	• IV TXA<br>• Placebo<br>• Restrictive threshold	blood loss	Wound complications	None	Not stated	None	Not stated
Xu 2017[365]	• China<br>• English<br>• 2016<br>• Single-Centre<br>• 80<br>• Patients with spinal degenerative diseases	(1) patients with comorbid severe medical diseases such as Osteoporosis, anaemia, renal failure, and cardiovascular diseases; (2) patients with abnormal coagulation function; (3) patients who have taken anti-platelet aggregates such as aspirin or anticoagulants in the last month; and (4) patients who had a history of thromboembolisms.	• Top TXA<br>• No TXA<br>• -	-	Intraoperative blood loss, drainage, transfusion requirements	None	Not stated	None	Not stated
Yanartas 2015[366]	• Turkey<br>• English<br>• 2015<br>• Single-Centre	Re-do cardiac surgery, emergent surgery, preoperative coagulation disorder, preoperative use of	• IV TXA (RS)<br>• RS only<br>• IV TXA (HES)<br>• HES only	values of haemoglobin, haematocrit, platelet,	the effect of priming solution on clinical out-comes such as; 1-Aortic cross-clamp time, 2-	None	Not stated	Unclear	Not stated

	• 132<br>• Patients undergoing CABG , 18 to 75 years of age, body mass index between 25 and 31, with normal ejection fraction (≥50%), initial haematocrit value within the boundaries of the normal for adult male and female patients (31 to 40% for women and 34 to 45% for men).	Clopidogrel, Coumarin anticoagulants, heparin, or acetylsalicylic acid within the previous 5 days before operation, preoperative congestive heart failure, ejection fraction <49%, preoperative renal dysfunction (serum creatinine > 1.3 mg/dL), chronic oliguria/anuria requiring dialysis, preoperative hepatic dysfunction (serum aspartate/alanine amino transferase > 40 U/L), preoperative electrolyte imbalance, history of pancreatitis or current Corticosteroid treatment.	• -	prothrombin time, activated prothrombin time, international normalized ratio (INR), blood urea nitrogen (BUN), creatinine, sodium, potas-sium, chloride, lactate, pH, base excess	Cardiopulmonary bypass time, 3-The use of inotropic support, 4-Intra-aortic balloon pump, 5-Prolonged mechanical ventilation, 6-Deve-lopment of pneumonia, 7-Perioperative myo-cardial infarction, 8-Cerebrovascular event (stroke, transient ischemic attack), seizure, 9-Atrial fibrillation and other rythm disturbances, 10-Need for renal replacement therapy (RRT), 11-Reoperation secondary to bleeding, 12-Intensive care unit stay, 13-Hospital stay and, 14-Thirty-day mortality				
Yang 2015[367]	• Greece<br>• English<br>• 2013<br>• Single-Centre<br>• 80<br>• Patients underwent Primary TKA	Patients with haemorrhagic blood diseases; haemoglobin (Hb)<90 g/L; with peripheral nerve vascular disease, cancer, history of thromboembolic disease; affected lower limb with a history of infection; and ASA rating>3.	• IA TXA<br>• Placebo<br>• -	-	Routine blood examination, blood loss and blood transfusion after TKA	None	Not stated	Unclear	Not stated
Yen 2017[368]	• Taiwan<br>• English<br>• 2016<br>• Single-Centre<br>• 98<br>• Patients who underwent primary minimally invasive TKA	Patients with a documented history of thromboembolic disease, cardiovascular disease (myocardial infarction or angina), stroke, coagulopathy, lifelong warfarin treatment for thromboembolic prophylaxis, impaired hepatic or renal function (impaired hepatic function was defined as liver	• IV TXA<br>• Top TXA<br>• Placebo<br>• -	Estimated total blood loss. Haemoglobin (Hb) and haematocrit (Hct) levels were measured on PODs 1, 2, and 4.	The rate of perioperative blood transfusion, the rate of deep-vein thrombosis (DVT), wound complications, visual analogue scale (VAS) on POD 1, the length of hospital stay, and the	None	Not stated	None	Not stated

		enzyme level, AST or ALT, which is more than twice normal range, history of liver cirrhosis, elevated total bilirubin level, or coagulopathy (INR < 1.3); and impaired renal function was defined as GFR<55ml/min/1.73 m^2, which is relative contraindicated for chemical venous thromboembolism and venography), and patients with an allergy history to tranexamic acid or concomitant use of protease inhibitors of human immunodeficiency virus, or fibrinolytic agent that contraindicated the use of rivaroxaban and preoperative anaemia (a haemoglobin level of ≤10 g/dl).			range of motion of the knee.				
Yuan 2017[369]	• China<br>• English<br>• 2017<br>• Single-Centre<br>• 560<br>• Patients who underwent TKA, osteoarthritis or rheumatoid arthritis, primary unilateral TKA, at least a 3-week follow-up, normal clotting mechanism, and effectively controlled medical diseases.	Previous bilateral TKA, revision TKA, severe hepatic and/or renal diseases, coagulopathy, or a bleeding disorder.	• IV TXA<br>• Top TXA<br>• PO TXA<br>• Placebo<br>• -	Postoperative 48-hour Hb loss and drainage volume, number of transfusions, transfusion and TXA costs, and thromboembolic complications.	Postoperative inpatient time and wound healing 3 weeks after TKA.	None	Not stated	Unclear	Not stated
Yue 2014[370]	• China<br>• English<br>• 2013<br>• Single-Centre<br>• 101	Patients who were receiving anticoagulant therapy, patients with a history of haemophilia, deep venous thrombosis, pulmonary embolism or ischemic heart disease and	• Top TXA<br>• Placebo<br>• -	The transfusion rate, the DVT and PE events.	Total blood loss, drain blood loss, haemoglobin and hematocrit drop, postoperative hospitalization days and other complications.	None	Not stated	None	Not stated

	• Patients undergoing primary unilateral total hip arthroplasty for OA or ONFH	patients who were allergic to tranexamic acid							
Zekcer 2017[371]	• Brazil<br>• English<br>• 2014<br>• Single-Centre<br>• 90<br>• Patients with unilateral total knee arthroplasty (TKA) as a result of Ahlbäch grade III, IV and V arthrosis	History or identified risk of deep venous thrombosis or pulmonary embolism or history of coagulation or cardiovascular disorders; vascular diseases	• IV TXA<br>• Top TXA<br>• No TXA<br>• -	volume of blood loss	Need for transfusion (patient received two units of packed red blood cells every time haemoglobin levels were below 8.0 g/dL).	None	Not stated	Unclear	Not stated
Zeng 2017[372]	• China<br>• English<br>• 2014<br>• Single-Centre<br>• 100<br>• All adult patients (aged between 18 and 90 years) undergoing primary unilateral THA	Allergy to TXA, preoperative hepatic or renal dysfunction, preoperative use of anticoagulant medication 7 days prior to surgery, history of fibrinolytic disorder, cerebrovascular accident, myocardial infarction, New York heart association class III or IV heart failure, atrial fibrillation, history of deep vein thrombosis or pulmonary embolus, preoperative international normalized ratio (INR) >1.4, activated partial thromboplastin time (aPTT) >1.4× normal, platelets <140 000/mm3, and failure to give consent.	• IV TXA<br>• Placebo<br>• -	total blood loss (calculated using Gross's equation), haemoglobin, haematocrit and platelet concentration changes on the third postoperative day, the amount of drainage, the amount of intraoperative blood loss, the frequency of transfusion, and the number of blood units transfused.	the length of postoperative stay, range of hip motion (measured by goniometer), Harris hip scores (HHS), and any perioperative complications or events such as infection, DVT or PE.	None	Not stated	Any	Non profit
Zhang 2007[373]	• Chinese<br>• Chinese<br>• 2007<br>• Single-Centre<br>• 102<br>• Patients underwent total knee arthroplasty	-	• IV TXA<br>• Placebo<br>• -	-	The amounts of blood loss and blood transfusion during operation and after operation.	None	Not stated	None	Not stated

Zhang 2015[374]	• China<br>• Chinese<br>• 2015<br>• Single-Centre<br>• 65<br>• Patients undergoing primary total hip arthroplasty	-	• IV TXA<br>• Placebo<br>• -	-	Intraoperative blood loss, postoperative dominant blood loss and hidden blood loss, pain score, blood transfusion rate, deep vein thrombosis and day of hospitalization	None	Not stated	None	Not stated
Zhang 2016[375]	• China<br>• English<br>• 2014<br>• Single-Centre<br>• 50<br>• Patients with osteonecrosis of the femoral head who underwent unilateral THA	Patients with diabetes, bleeding disorders, preoperative anaemia (haemoglobin Hb<120g/l),malignancies, history of venous thrombosis disease, arteriosclerosis, varicose veins and other cardiovascular diseases, allergy to TXA, liver and kidney dysfunction, participation in other clinical trials and intraoperative adverse events which were believed could lead to intraoperative and postoperative bleeding.	• IV TXA<br>• No TXA<br>• Restrictive threshold	-	Adverse events, intraoperative blood loss, postoperative drainage, total loss of red blood cells.	None	Not stated	None	Not stated
Zhou 2018[376]	• China<br>• English<br>• 2018<br>• Single-Centre<br>• 170<br>• All adult patients scheduled to undergo primary unilateral THA in our hospital and consented	e allergy to TXA; coagulopathy (preoperative platelet count < 150,000/ mm3; international normalized ratio (INR) > 1.4; or any indicator of prolonged partial thromboplastin, prothrombin, and thrombin time of >1.4 times the normal.); history of thromboembolic disease, including deep vein thrombosis (DVT), pulmonary embolism (PE), myocardial infarction (MI), and cerebral infarction (CI); taking anticoagulant drugs within a week before surgery; major comorbidities, including	• IV TXA<br>• Top TXA<br>• Placebo<br>• -	total blood loss	Allogeneic blood transfusion requirement, drain blood loss, decreased haemoglobin level.	None	Not stated	None	Not stated

		severe ischemic heart disease (New York Heart Association Class III or IV), renal dysfunction (glomerular filtration rate < 60), or hepatic dysfunction (glutamic–pyruvic transaminase > 80 or glutamic oxaloacetic transaminase > 80); retinopathy; pregnancy; participated in another clinical trial within a year; and those who completely stay in bed for more than 3 weeks.							
Dryden 1997[377]	• Canada<br>• English<br>• 1997<br>• Single-Centre<br>• 41<br>• Patients scheduled for re-do valve replacement	Patients with a history of thrombosis, pre-existing coagulopathy, creatinine > 250 mg/dl, or a known allergy to TA. A history of thrombosis referred to previous deep vein thrombosis, disseminated intravascular coagulation, non-embolic stroke within six months, unstable angina, or bleeding into the renal tract	• IV TXA<br>• Placebo<br>• -	-	Blood loss, and the transfusion of blood products.	None	Non profit	Any	Industry
Johnson 1992[378]	• USA<br>• English<br>• 1992<br>• Single-Centre<br>• 38<br>• Autologous blood donors undergoing elective myocardial revascularization.<br>• Restrictive threshold Haematocrit <25%	-	• Restrictive 80g/L<br>• Liberal<br>• -	-	Cardiac events, complications, postoperative blood loss, blood use (total units), allogeneic blood use (units), autologous blood use (units), all product blood use (units), number of participants receiving transfusions, mean cardiac index, mean systemic resistance, exercise capacity, Hct levels, length of ICU stay, length of hospital stay	None	Non profit	None	Non profit

Murphy 2015[379]	• UK<br>• English<br>• 2015<br>• Multi-Centre<br>• 2003<br>• Patients older than 16 years of age who were undergoing non-emergency cardiac surgery. Patients providing written informed consent. Post-operative haemoglobin level below 9.0g/dL or haematocrit below 27 at any stage during patient's post-operative hospital stay<br>• Restrictive threshold 7.5g/dl	Patients who are prevented from having blood and blood products according to a system of beliefs. Patients with congenital or acquired platelet, red cell or clotting disorders. Patients with ongoing or recurrent sepsis. Patients with critical limb ischemia. Patients undergoing emergency cardiac surgery. Patients already participating in another interventional research study. Patients unable to give full informed consent for the study.	• Restrictive 75g/L<br>• Liberal<br>• Tranexamic acid<br>• Cell salvage	composite of a serious infection (sepsis or wound infection) or an ischaemic event (permanent stroke, myocardial infarction, infarction of the gut, or acute kidney injury)within 3months after randomisation.	units transfused, infection, ischaemic events, acute kidney injury, hospital stay and ICU stay, and cost	None	Non profit	None	Non profit
Nielsen 2014[380]	• Denmark<br>• English<br>• 2014<br>• Single-Centre<br>• 66<br>• Patients were eligible if they were at least 18 years of age and scheduled for elective hip revision surgery.<br>• Restrictive threshold 7.3g/dl	Exclusion criteria were disseminated cancer or cardiac disease with functional impairment (NYHA class II or above).	• Restrictive 73g/L<br>• Liberal<br>• Tranexamic acid	"Time up and go" test (time it takes a patient to stand up, walk three meters, turn around, walk back and sit down again)	pneumonia, wound infection, gastrointestinal complications, dizziness, hypotension, fatigue, deep vein thrombosis, and fall	None	Non profit	Unclear	Not stated
Karkouti 2016[381]	• Canada<br>• English<br>• 2015<br>• Multi-Centre<br>• 7402<br>• patients undergoing cardiac surgery with cardiopulmonary bypass	None stated	• ROTEM + PLT MAPPING<br>• Control<br>• -	red cell transfusion from surgery to postoperative day seven-	Transfusion of other blood products, major bleeding, and major complications.				

5 Risk of bias report and summary for included studies. (eFigure 2)

The overall risk of bias is indicated by [green] for low risk of bias, [yellow] for unclear risk of bias, and [red] for high risk of bias. The results are expressed as percentages, with 388 studies included. For the details of the criteria used for rating, please see: Higgins JPT, et al. 2011. Assessing risk of bias in included studies. Chapter 8. Cochrane Handbook for Systematic Reviews of Interventions Version 5.10: The Cochrane Collaboration.

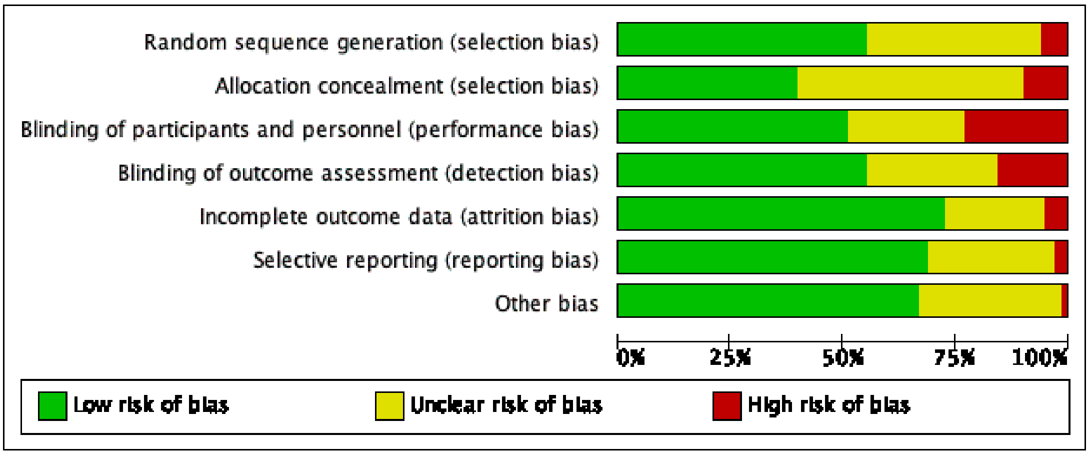

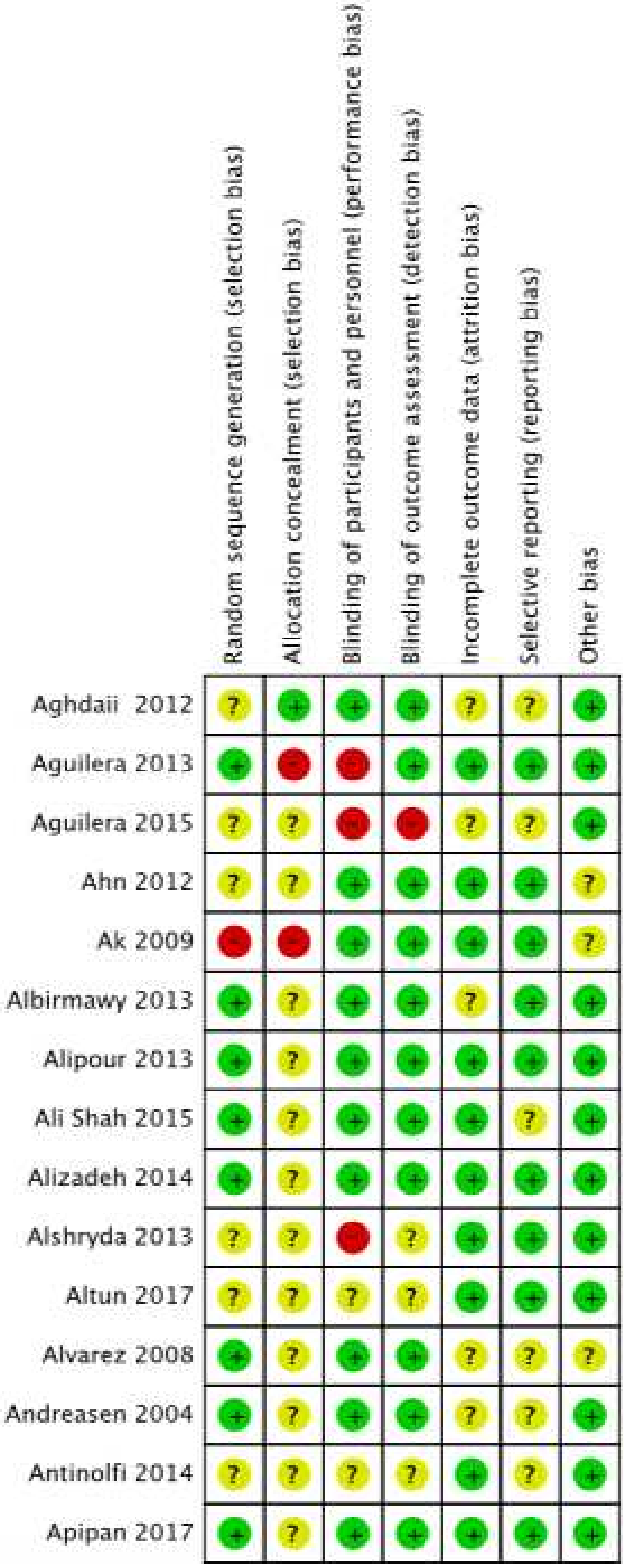
Random sequence generation (selection bias)
Allocation concealment (selection bias)
Blinding of participants and personnel (performance bias)
Blinding of outcome assessment (detection bias)
Incomplete outcome data (attrition bias)
Selective reporting (reporting bias)
Other bias
Aghdaii 2012
Aguilera 2013
Aguilera 2015
Ahn 2012
Ak 2009
Albirmawy 2013
Alipour 2013
Ali Shah 2015
Alizadeh 2014
Alshryda 2013
Altun 2017
Alvarez 2008
Andreasen 2004
Antinolfi 2014
Apipan 2017

Arantes 2016	+	?	+	+	+	+	?
Armellin 2001	?	?	?	+	?	?	?
Ausen 2015	+	+	+	+	+	?	+
Auvinen 1987	?	?	+	+	+	?	+
Avidan 2004	?	+	-	-	+	+	+
Bansal 2017	+	?	+	+	+	+	+
Baradaranfar 2017	+	?	+	+	+	?	+
Barrachina 2016	+	?	+	+	+	+	+
Baruah 2016	?	?	?	-	+	+	+
Basavaraj 2017	?	+	+	+	+	+	+
Beikaei 2015	+	?	+	+	?	?	?
Benoni 1996	?	+	+	+	?	?	?
Benoni 2000	+	?	+	+	?	?	+
Benoni 2001	?	+	+	+	?	?	+
Bernabeu Wittel 2016	+	?	+	+	?	+	+
Bidolegui 2014	?	?	-	-	+	+	+
Blatsoukas 2010	?	?	-	-	+	+	+
Blauhut 1994	?	?	?	?	?	?	?
Boylan 1996	?	+	+	+	+	?	+
Bracey 1999	-	-	?	+	+	+	+
Bradshaw 2012	+	?	?	?	?	+	?
Brown 1997a	?	?	?	?	+	+	?
Brown 1997b	?	?	?	?	+	+	?
Bulutcu 2005	?	?	+	+	+	?	?
Bush 1997	?	-	-	?	+	+	+
Campbell 2012	?	?	+	+	?	+	+
Cao 2015	-	?	-	?	+	+	?
Carabini 2018	+	?	+	+	+	+	?

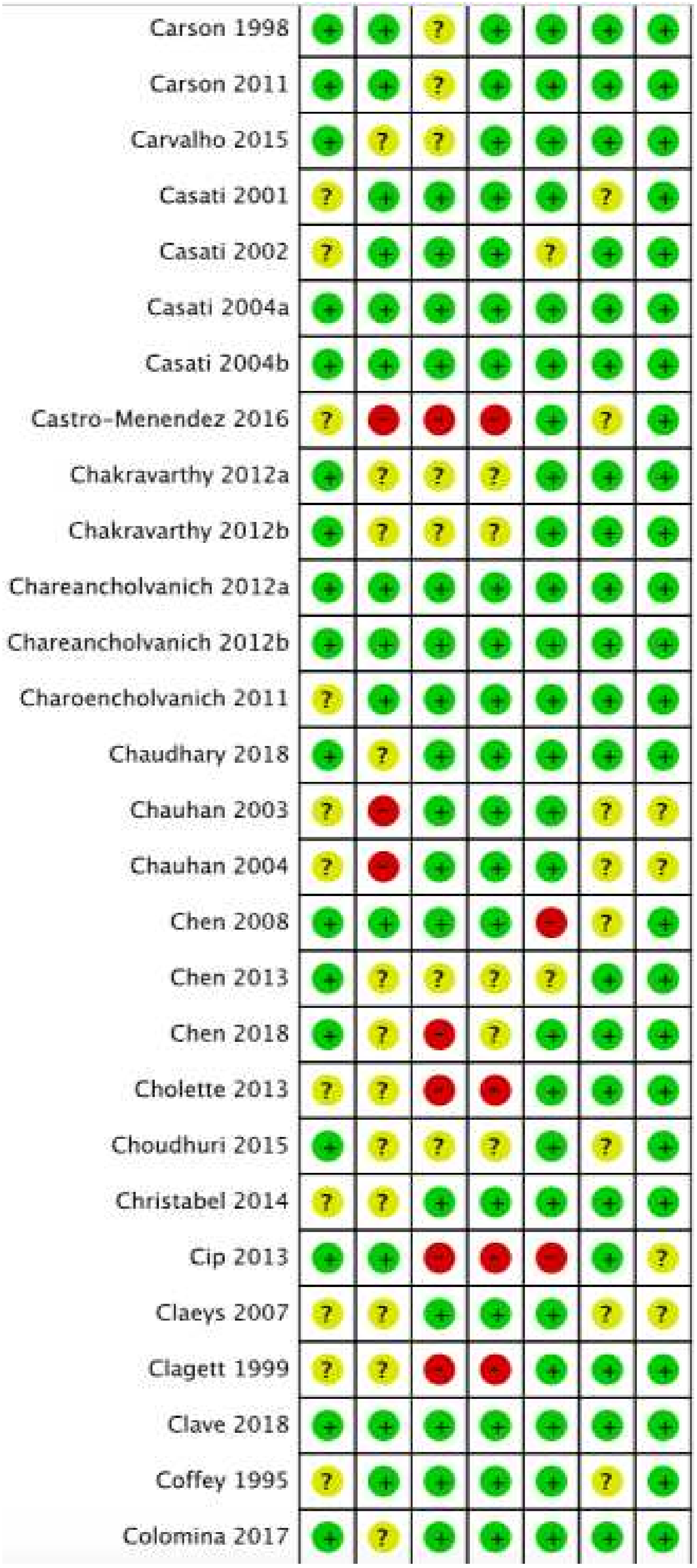
Carson 1998 + + ? + + + +
Carson 2011 + + ? + + + +
Carvalho 2015 + ? ? + + + +
Casati 2001 ? + + + + ? +
Casati 2002 ? + + + ? + +
Casati 2004a + + + + + + +
Casati 2004b + + + + + + +
Castro-Menendez 2016 ? - - - + ? +
Chakravarthy 2012a + ? ? ? + + +
Chakravarthy 2012b + ? ? ? + + +
Chareancholvanich 2012a + + + + + + +
Chareancholvanich 2012b + + + + + + +
Charoencholvanich 2011 ? + + + + + +
Chaudhary 2018 + ? + + + + +
Chauhan 2003 ? - + + + ? ?
Chauhan 2004 ? - + + + ? ?
Chen 2008 + + + + - ? +
Chen 2013 + ? ? ? ? + +
Chen 2018 + ? - ? + + +
Cholette 2013 ? ? - - + + +
Choudhuri 2015 + ? ? ? + ? +
Christabel 2014 ? ? + + + + +
Cip 2013 + + - - - + ?
Claeys 2007 ? ? + + + ? ?
Clagett 1999 ? ? - - + + +
Clave 2018 + + + + + + +
Coffey 1995 ? + + + + ? +
Colomina 2017 + ? + + + + +

Corbeau 1995	?	?	?	?	?	?	?
Crescenti 2011	+	+	+	+	+	+	+
Cui 2010	?	?	−	−	−	?	−
Cvetanovich 2018	+	+	+	+	+	+	+
Dadure 2011	+	+	+	?	+	+	+
Dalmau 2000	?	?	+	+	?	?	?
Dalrymple-Hay 1999	+	?	−	−	?	+	+
Damgard 2010	?	?	−	?	+	+	+
Das 2015	+	?	+	+	+	+	+
de Almeida 2015	+	+	?	+	+	+	+
Dell'Amore 2012	+	?	+	+	+	+	+
Dell'Atti 2016	?	?	?	?	+	?	+
De Napoli 2016	?	+	+	?	−	−	+
Dietrich 1989	?	?	−	?	?	?	?
Digas 2015	?	+	?	+	+	+	+
Diprose 2005	+	+	+	+	?	?	+
Drakos 2016	?	?	+	+	+	+	+
Drosos 2016	?	?	?	?	+	+	+
Dryden 1997	?	?	+	+	+	?	?
Edwards 2009	+	+	−	+	+	+	+
Eftekharian 2014	?	?	+	+	+	+	+
Ekback 2000	?	?	+	+	+	?	?
Elawad 1991	?	?	−	−	+	+	+
Eldaba 2013	+	+	+	+	+	+	+
El Shahl 2015	+	?	+	+	+	+	+
Elshamaa 2015	?	+	+	+	+	+	+
Elwatidy 2008	−	+	+	+	+	?	+
Emara 2014	?	?	+	+	+	+	+

Engel 2001	?	?	?	+	+	?	?
Esfandiari 2013	?	?	+	?	+	+	+
Fan 2014	+	+	?	?	+	+	+
Faraoni 2014	?	?	?	?	?	?	?
Farrokhi 2011	+	+	+	+	+	+	+
Felli 2019	+	+	+	+	+	+	?
Fernandez-Cortinas 2017	-	?	?	?	?	+	?
Foss 2009	+	?	+	+	?	+	+
Fraval 2016	+	+	+	+	?	+	?
Fraval 2018	?	?	+	+	+	+	+
Froessler 2016	+	+	?	?	?	+	?
Garneti 2004	+	?	+	+	+	?	+
Garrido Martin 2012	+	?	+	+	-	+	?
Gatling 2018	+	+	?	?	+	+	?
Gautam 2013	?	?	?	?	?	+	+
Geng 2017	+	?	?	?	+	+	+
Georgiadis 2013	+	+	+	+	+	+	+
Ghaffari 2012	?	?	+	+	?	+	+
Gill 2009	+	?	+	+	+	?	+
Gillespie 2015	?	?	+	+	?	+	+
Girdauskas 2010	+	+	-	-	+	+	?
Goobie 2018	+	?	?	+	+	+	?
Good 2003	+	?	+	+	-	?	?
Gregersen 2015	+	+	?	+	+	+	+
Greiff 2012	?	?	+	+	+	+	+
Grover 2006	+	?	?	+	?	?	+
Guerreiro 2017	?	?	-	-	+	+	+
Gupta 2012	-	?	+	+	?	+	+

Guzel 2016	?	?	?	?	+	+	+
Haghighi 2017	?	?	+	+	+	+	+
Hajjar 2010	+	+	?	+	+	+	+
Hardy 1998	?	+	+	+	?	?	+
Hashemi 2011	?	?	+	+	+	+	+
Hiippala 1995	+	?	?	?	-	+	?
Hiippala 1997	?	?	+	+	?	+	+
Hogan 2015	+	+	-	?	?	+	+
Hooda 2017	+	?	+	+	+	+	+
Horrow 1990	+	+	+	+	?	+	+
Horrow 1991	+	+	+	+	+	?	+
Horrow 1995	+	+	+	+	?	?	+
Horstmann 2013	?	+	+	+	+	+	+
Horstmann 2014	+	+	?	+	+	?	+
Hosseini 2014	+	?	+	?	?	+	+
Hou 2015	+	-	-	-	+	+	?
Hsu 2015	+	+	+	?	?	?	+
Hu 2018	+	?	?	-	+	?	?
Huang 2015	+	-	-	-	?	?	-
Huang 2016	?	?	?	?	+	+	+
Huang 2017	+	+	+	+	+	+	+
Husted 2003	+	+	+	+	+	?	+
Imai 2012	?	?	-	-	+	?	+
Ishida 2011	?	?	+	?	+	+	+
Jansen 1999	+	?	+	+	+	?	+
Jares 2003	?	?	-	-	+	?	?
Jaszczyk 2015	?	+	?	?	+	+	+
Jendoubi 2017a	?	?	+	?	+	?	+

Jendoubi 2017b	?	?	+	?	+	?	+
Jimenez 2007	?	+	+	+	+	?	+
Johansson 2005	+	+	+	+	+	?	+
Johansson P 2015	+	+	+	+	?	+	+
Johnson 1992	-	?	?	?	?	+	+
Jordan 2019	+	+	-	-	+	+	?
Kakar 2009	?	?	+	+	+	+	+
Karaaslan 2015a	+	?	+	+	+	+	+
Karaaslan 2015b	+	?	+	+	+	+	+
Karimi 2012	+	+	+	+	+	+	+
Karkouti 2016	+	-	-	-	+	-	?
Karski 1995	+	+	+	+	+	+	+
Karski 2005	?	?	+	+	+	?	+
Kaspar 1997	?	+	+	+	?	+	+
Katoh 1997	?	?	?	?	+	?	?
Katsaros 1996	?	?	+	+	+	?	+
Kazemi 2010	?	?	+	+	+	?	+
Keyhani 2016	?	-	?	?	+	+	+
Kim 2014	+	?	?	+	+	+	+
Kim 2016	+	+	?	?	?	+	?
Kim 2018	+	+	+	+	?	+	+
Kimenai 2016	+	?	+	+	+	+	+
Klein 2008	+	-	-	-	+	+	+
Koch 2017	?	?	+	+	+	+	+
Kojima 2001	?	?	?	?	+	?	?
Kuitunen 2005	?	+	+	+	+	?	+
Kuitunen 2006	?	?	?	?	?	?	?

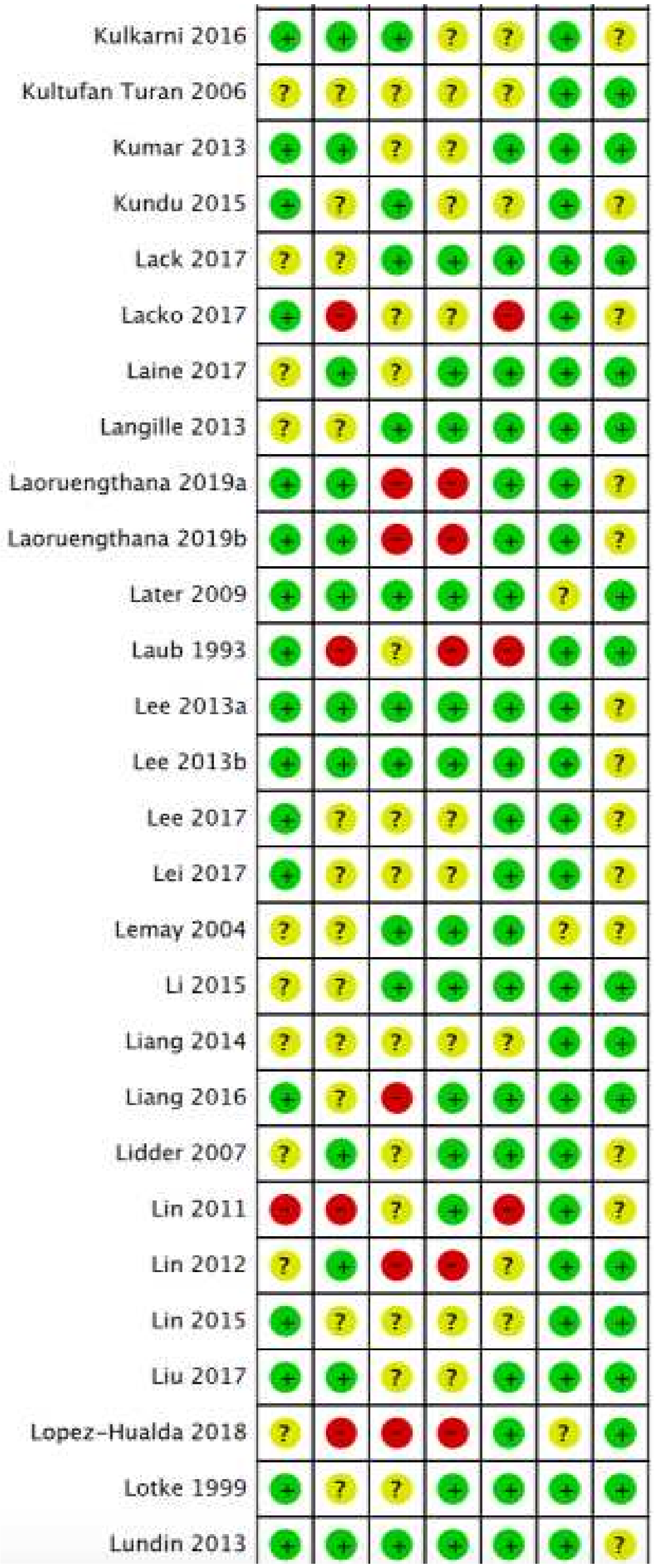
Kulkarni 2016
Kultufan Turan 2006
Kumar 2013
Kundu 2015
Lack 2017
Lacko 2017
Laine 2017
Langille 2013
Laoruengthana 2019a
Laoruengthana 2019b
Later 2009
Laub 1993
Lee 2013a
Lee 2013b
Lee 2017
Lei 2017
Lemay 2004
Li 2015
Liang 2014
Liang 2016
Lidder 2007
Lin 2011
Lin 2012
Lin 2015
Liu 2017
Lopez-Hualda 2018
Lotke 1999
Lundin 2013

Luo 2019	+	−	−	?	?	+	?
MacGillivray 2011	?	?	+	+	+	?	?
Maddali 2007	+	+	+	+	+	?	+
Malhotra 2011	?	?	+	+	+	?	+
Maniar 2012	?	+	?	+	+	+	?
Mansouri 2012	?	?	+	?	+	?	+
Marberg 2010	+	+	−	−	+	+	+
Markatou 2012	?	−	−	?	+	−	−
Martin 2014	+	+	+	+	+	?	?
Mazer 2017	+	+	?	+	+	+	+
McConnell 2011	?	+	?	+	+	+	+
McGill 2002	+	−	−	−	+	+	+
Mehr-Aein 2007	?	?	+	+	+	?	?
Melo 2017	?	−	−	?	+	−	?
Meng 2019	−	−	−	−	+	+	?
Menges 1992	?	?	−	?	+	+	?
Menichetti 1996	?	?	?	?	+	+	+
Mercer 2004	?	?	−	−	+	+	+
Miller 1980	−	?	?	?	?	?	−
Min 2015	+	?	−	−	+	+	?
Mirmohammadsadeghi 2018	−	−	−	?	+	+	?
Mohib 2015	+	+	+	?	+	?	?
Moller 2019	+	+	−	−	+	+	+
Molloy 2007	?	?	+	+	+	?	+
Motififard 2015	+	?	+	+	+	+	+
Mu 2019	−	−	−	−	+	?	?
Murphy 2004	+	+	−	−	+	+	?

Murphy 2005	+	+	−	−	+	+	+
Murphy 2006	?	+	+	+	+	?	+
Murphy 2015	+	+	?	+	+	+	+
Myles 2017	+	+	+	+	+	+	+
Na 2016	+	+	+	?	?	+	?
Nagabhushan 2017	+	+	+	?	+	+	+
Napoli 2016	?	+	+	?	+	+	?
Neilipovitz 2001	+	?	+	+	+	?	+
Nielsen 2014	+	+	?	?	+	+	+
Niskanen 2005	?	?	+	+	?	?	?
Nuttal 2001	+	+	−	−	+	+	?
Nuttall 2000	+	?	+	+	?	?	+
Oertli 1994	?	?	?	?	?	?	?
Onodera 2012	+	?	?	?	?	+	+
Oremus 2014	+	+	+	+	−	−	+
Orpen 2006	?	?	+	+	+	?	+
Oztas 2015	+	+	+	+	+	?	+
Painter 2018	+	+	+	+	+	+	+
Palmieri 2017	+	?	−	?	+	+	?
Parker 2013	?	+	?	?	?	+	+
Parrot 1991	?	?	−	−	+	+	+
Pauzenberger 2017	+	−	−	+	+	+	?
Pawar 2016	?	?	?	?	?	+	+
Penta de Peppo 1995	−	−	−	−	−	−	?
Perez-Jimeno 2018	−	?	−	−	−	+	+
Pertlicek 2015	+	−	−	?	+	+	?
Peters 2015	+	+	+	+	+	+	?
Pinosky 1997	?	?	+	+	+	?	?

Pleym 2003	+	?	+	+	?	?	+
Pourfakhr 2016	?	−	−	−	−	−	−
Prabhu 2015	+	+	+	−	?	+	+
Prakash 2017	+	?	+	+	?	+	+
Prasad 2018	+	+	+	+	+	+	+
Pugh 1995	?	?	−	−	?	?	?
Raksakietisak 2015	+	+	+	+	+	+	+
Rannikko 2004	?	?	?	+	−	?	?
Raviraj 2012	+	+	+	+	+	+	?
Reid 1997	?	?	+	+	−	+	?
Reyes 2010	?	?	−	?	?	?	+
Rollo 1995	?	−	−	−	+	+	+
Roy 2012	−	?	+	−	+	+	+
Royston 2001	?	+	?	?	+	+	?
Sabry 2018	+	+	+	+	+	+	?
Sadeghi 2007	+	+	?	+	+	+	+
Sa-Ngasoongsong 2011	+	+	+	+	+	+	+
Sa-Ngasoongsong 2013	+	+	+	+	+	+	?
Santos 2006	?	?	+	+	+	+	+
Sarkanovic 2013	?	?	−	?	?	?	+
Sarzaeem 2014	−	?	+	?	+	−	?
Savvidou 2009	?	?	−	?	−	−	+
Schiavone 2018	?	?	?	?	+	+	+
Scrascia 2012	+	?	−	−	+	+	+
Seddighi 2017	?	−	+	−	+	+	+
Seo 2013	−	+	−	−	+	+	?
Seol 2016	−	?	+	+	+	+	+

160

Serran-Trenas 2011	+	+	-	-	+	+	?
Sethna 2005	?	?	?	?	?	+	?
Seviciu 2016	+	+	+	+	+	+	?
Shakeri 2018	+	+	-	+	+	+	+
Shehata 2012	+	+	?	?	+	+	+
Shen 2015	+	+	+	+	-	-	+
Shen 2016	+	?	-	?	+	+	+
Shenolikar 1997	+	?	-	-	+	+	+
Shi 2013a	+	+	+	+	+	+	+
Shi 2013b	+	+	+	+	+	+	+
Shi 2017	+	+	+	+	+	+	+
Shimizu 2011	+	?	-	-	+	+	+
Shinde 2015	+	+	+	+	+	+	+
Shore-Lesserson 1996	+	?	+	+	-	?	+
Shore-Lesserson 1999	+	+	+	+	+	+	+
Slagis 1991	?	?	-	-	?	+	+
Song 2017	+	+	+	+	?	+	?
So-Osman 2013	+	+	?	?	+	+	+
So-Osman 2014	+	+	-	+	+	+	+
Spahn 2019	+	+	+	+	+	+	+
Spark 1997	?	-	-	-	+	+	+
Speekenbrink 1995	?	?	?	?	+	?	?
Spitler 2019	+	?	?	?	+	+	?
Springer 2016	+	+	?	?	-	?	?
Stowers 2017	+	+	+	+	+	?	?
Sudprasert 2019	+	?	?	?	+	+	?
Sun 2017	+	+	+	?	+	+	+
Taghaddomi 2009a	+	?	?	?	+	?	?

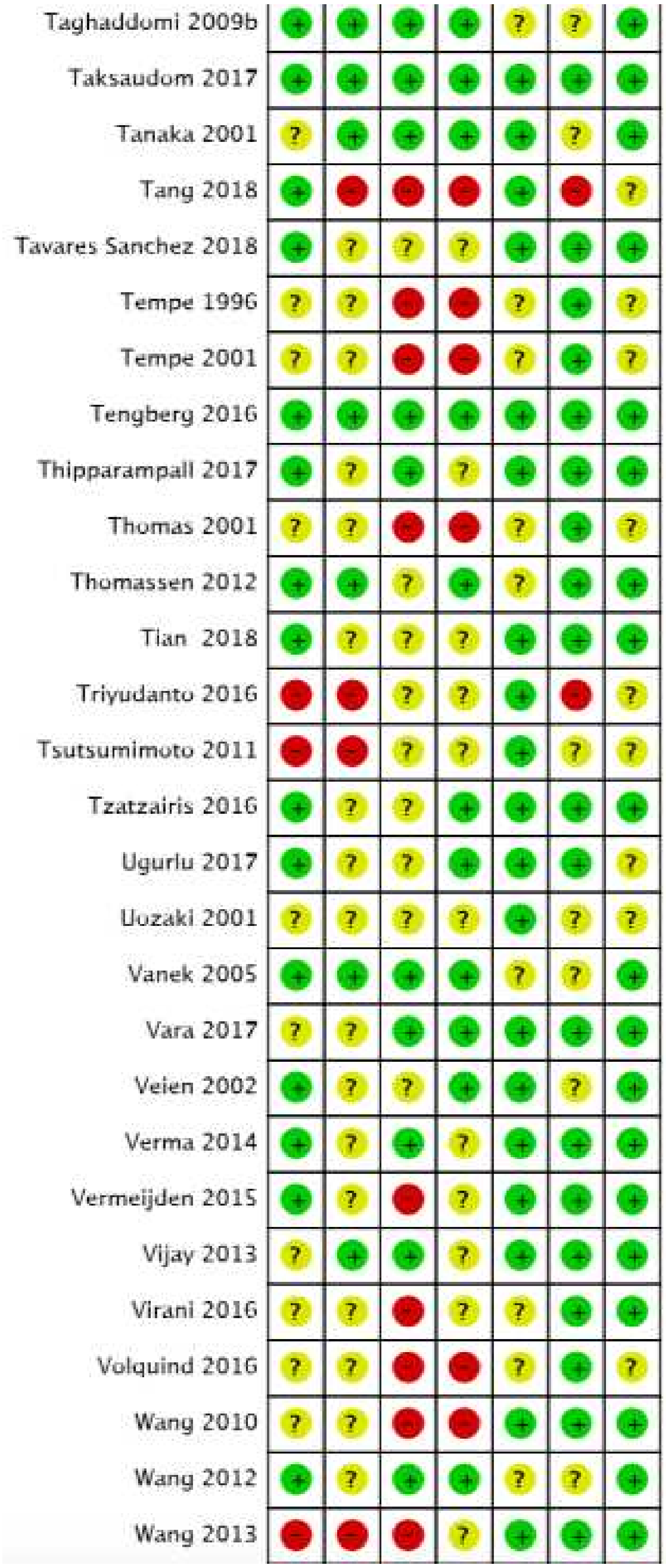
Taghaddomi 2009b
Taksaudom 2017
Tanaka 2001
Tang 2018
Tavares Sanchez 2018
Tempe 1996
Tempe 2001
Tengberg 2016
Thipparampall 2017
Thomas 2001
Thomassen 2012
Tian 2018
Triyudanto 2016
Tsutsumimoto 2011
Tzatzairis 2016
Ugurlu 2017
Uozaki 2001
Vanek 2005
Vara 2017
Veien 2002
Verma 2014
Vermeijden 2015
Vijay 2013
Virani 2016
Volquind 2016
Wang 2010
Wang 2012
Wang 2013

Wang 2015a	+	+	+	+	+	+	+
Wang 2015b	+	+	+	+	+	+	?
Wang 2015c	?	-	-	?	+	+	?
Wang 2016	+	+	+	+	+	+	+
Wang 2017a	+	+	?	?	+	+	+
Wang 2017b	+	+	-	+	+	+	+
Wang 2019	+	+	+	+	+	+	+
Watts 2017	+	+	+	+	+	+	?
Weber 2012	+	+	-	-	?	+	?
Wei 2006	?	?	?	+	+	?	?
Wei 2014	+	+	?	+	+	+	+
Westbrook 2009	?	?	?	?	+	+	?
Wiefferink 2007	+	-	-	?	+	+	+
Wong 2008	+	+	+	+	?	?	+
Wu 2006	?	?	+	+	+	?	?
Xie 2015	?	+	+	+	+	+	+
Xu 2012	-	-	?	?	+	+	?
Xu 2015	?	+	+	+	?	?	+
Xu 2017	?	?	+	+	+	+	+
Xu 2019	+	+	+	-	+	?	?
Yanartas 2015	+	+	+	+	-	+	+
Yang 2015	+	+	+	+	+	?	?
Yassen 1993	-	-	-	?	+	+	?
Yen 2017	+	+	+	+	+	+	?
Yi 2016	+	?	+	+	+	+	+
Yuan 2017	+	+	?	+	+	+	+
Yue 2014	+	+	+	+	+	+	+
Zabeeda 2002	?	?	?	+	?	?	?

163

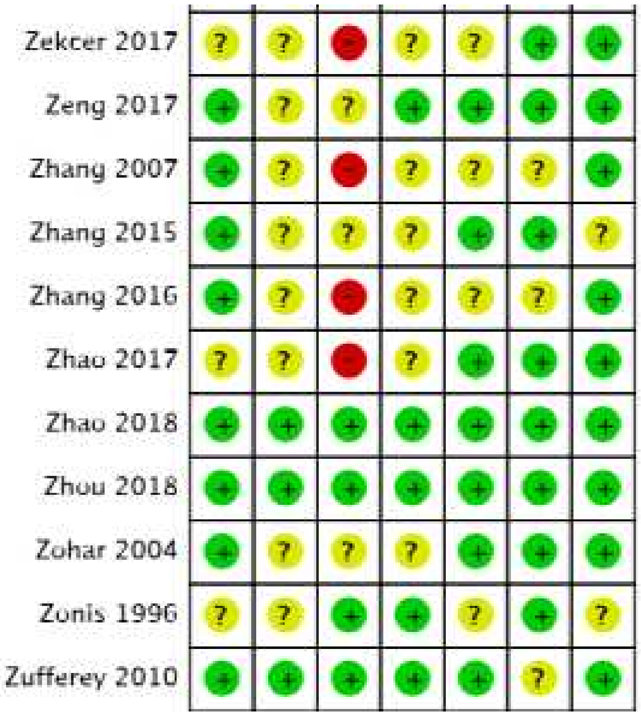
Zekcer 2017
Zeng 2017
Zhang 2007
Zhang 2015
Zhang 2016
Zhao 2017
Zhao 2018
Zhou 2018
Zohar 2004
Zonis 1996
Zufferey 2010

164

6 Secondary outcomes based on Author and Funding Conflicts of Interest. (eTable 2)

Risk ratios (RR) with 95% confidence intervals (CIs) in 'none', 'unclear' and 'any' conflict of interest. Squares indicate study-specific MD estimates; horizontal lines indicate the 95% CI; diamonds indicate the pooled RRs with their 95% CI.

Outcome	CoI Moderator	Subtype	# of studies	Patients (n)	Output measurement type	I^2	P value	Result	P value
**Myocardial Infarction**	**Overall**		54	22414	Risk Ratio (M-H, Random, 95% CI)	0%	0.98	0.95 [0.85, 1.06]	0.34
	Author	None	19	6557	Risk Ratio (M-H, Random, 95% CI)	0%	0.92	1.02 [0.67, 1.55]	0.94
		Unclear	25	3210	Risk Ratio (M-H, Random, 95% CI)	0%	0.97	0.82 [0.56, 1.20]	0.3
		Any	10	12647	Risk Ratio (M-H, Random, 95% CI)	9%	0.36	0.96 [0.85, 1.08]	0.47
	Author Type	Not stated	43	7808	Risk Ratio (M-H, Random, 95% CI)	0%	0.99	0.93 [0.70, 1.24]	0.63
		Non-Profit	4	8688	Risk Ratio (M-H, Random, 95% CI)	46%	0.14	0.95 [0.82, 1.10]	0.47
		Blood service	2	258	Risk Ratio (M-H, Random, 95% CI)	0%	0.6	0.60 [0.08, 4.41]	0.62
		Professional advocacy organisation	2	514	Risk Ratio (M-H, Random, 95% CI)	0%	0.59	0.22 [0.05, 1.06]	0.06
		Industry	5	5660	Risk Ratio (M-H, Random, 95% CI)	0%	0.41	0.96 [0.77, 1.20]	0.72
	Funding	None	14	3752	Risk Ratio (M-H, Random, 95% CI)	0%	0.82	1.08 [0.65, 1.78]	0.78
		Unclear	24	3011	Risk Ratio (M-H, Random, 95% CI)	0%	0.98	0.90 [0.60, 1.37]	0.63
		Any	16	15651	Risk Ratio (M-H, Random, 95% CI)	0%	0.56	0.94 [0.84, 1.06]	0.35
	Funding Type	Not stated	34	4418	Risk Ratio (M-H, Random, 95% CI)	0%	0.98	1.00 [0.72, 1.40]	1
		Non-Profit	10	9803	Risk Ratio (M-H, Random, 95% CI)	0%	0.46	0.94 [0.81, 1.09]	0.41
		Blood service	6	7171	Risk Ratio (M-H, Random, 95% CI)	0%	0.68	0.98 [0.79, 1.22]	0.88
		Professional advocacy organisation	2	514	Risk Ratio (M-H, Random, 95% CI)	0%	0.59	0.22 [0.05, 1.06]	0.06
		Industry	4	1022	Risk Ratio (M-H, Random, 95% CI)	0%	0.71	0.44 [0.17, 1.14]	0.09
**Adverse Reaction**	**Overall**		112	20192	Risk Ratio (M-H, Random, 95% CI)	0%	0.57	0.87 [0.82, 0.93]	**<0.001**
	Author	None	48	8107	Risk Ratio (M-H, Random, 95% CI)	0%	0.52	0.86 [0.78, 0.95]	**0.004**

		Unclear	56	6176	Risk Ratio (M-H, Random, 95% CI)	0%	0.68	0.86 [0.78, 0.94]	**0.002**
		Any	8	5909	Risk Ratio (M-H, Random, 95% CI)	41%	0.1	1.02 [0.83, 1.26]	0.85
	Author Type	Not stated	104	14281	Risk Ratio (M-H, Random, 95% CI)	0%	0.68	0.86 [0.80, 0.92]	**<0.001**
		Non-Profit	3	4831	Risk Ratio (M-H, Random, 95% CI)	4%	0.35	4.51 [1.53, 13.28]	**0.006**
		Blood service	1	102	Risk Ratio (M-H, Random, 95% CI)	N/A	N/A	0.20 [0.01, 4.07]	0.29
		Professional advocacy organisation	4	802	Risk Ratio (M-H, Random, 95% CI)	0%	0.68	0.96 [0.78, 1.17]	0.66
		Industry	4	978	Risk Ratio (M-H, Random, 95% CI)	0%	0.66	0.95 [0.76, 1.19]	0.65
	Funding	None	38	4155	Risk Ratio (M-H, Random, 95% CI)	18%	0.17	0.77 [0.63, 0.94]	**0.009**
		Unclear	49	5373	Risk Ratio (M-H, Random, 95% CI)	0%	0.64	0.72 [0.60, 0.85]	**<0.001**
		Any	25	10664	Risk Ratio (M-H, Random, 95% CI)	0%	0.62	0.94 [0.81, 1.10]	0.45
	Funding Type	Not stated	81	13340	Risk Ratio (M-H, Random, 95% CI)	7%	0.29	0.85 [0.78, 0.93]	**<0.001**
		Non-Profit	19	3389	Risk Ratio (M-H, Random, 95% CI)	0%	0.8	0.86 [0.74, 1.00]	**0.05**
		Blood service	3	1977	Risk Ratio (M-H, Random, 95% CI)	0%	0.63	0.96 [0.73, 1.26]	0.79
		Professional advocacy organisation	4	802	Risk Ratio (M-H, Random, 95% CI)	0%	0.68	0.96 [0.78, 1.17]	0.66
		Industry	9	1486	Risk Ratio (M-H, Random, 95% CI)	49%	0.86	0.95 [0.81, 1.12]	0.54
**Low cardiac output**	**Overall**		25	8708	Risk Ratio (M-H, Random, 95% CI)	40%	0.02	0.97 [0.91, 1.04]	0.39
	Author	None	11	2019	Risk Ratio (M-H, Random, 95% CI)	0%	0.55	0.51 [0.38, 0.70]	**<0.001**
		Unclear	12	1733	Risk Ratio (M-H, Random, 95% CI)	0%	0.58	1.18 [0.78, 1.77]	0.43
		Any	2	4956	Risk Ratio (M-H, Random, 95% CI)	0%	0.49	1.01 [0.94, 1.08]	0.84
	Author Type	Not stated	23	3814	Risk Ratio (M-H, Random, 95% CI)	27%	0.13	0.71 [0.56, 0.90]	**0.005**
		Non-Profit	1	38	Risk Ratio (M-H, Random, 95% CI)	N/A	N/A	0.30 [0.01, 6.97]	0.45
		Blood service	0	0	Risk Ratio (M-H, Random, 95% CI)	N/A	N/A	Not estimable]	**N/A**

		Professional advocacy organisation	1	216	Risk Ratio (M-H, Random, 95% CI)	N/A	N/A	3.11 [0.13, 75.56]	0.82
		Industry	1	4856	Risk Ratio (M-H, Random, 95% CI)	42%	0.06	1.01 [0.94, 1.08]	**<0.001**
	Funding	None	9	1163	Risk Ratio (M-H, Random, 95% CI)	7%	0.38	0.64 [0.39, 1.06]	0.08
		Unclear	6	730	Risk Ratio (M-H, Random, 95% CI)	54%	0.06	0.63 [0.44, 0.90]	**0.01**
		Any	10	6815	Risk Ratio (M-H, Random, 95% CI)	0%	0.47	1.00 [0.94, 1.07]	0.95
	Funding Type	Not stated	13	1633	Risk Ratio (M-H, Random, 95% CI)	26%	0.19	0.64 [0.48, 0.86]	**0.003**
		Non-Profit	6	1260	Risk Ratio (M-H, Random, 95% CI)	0%	0.45	0.44 [0.23, 0.85]	**0.01**
		Blood service	3	5074	Risk Ratio (M-H, Random, 95% CI)	0%	0.63	1.01 [0.95, 1.08]	0.73
		Professional advocacy organisation	1	216	Risk Ratio (M-H, Random, 95% CI)	N/A	N/A	3.11 [0.13, 75.56]	0.49
		Industry	3	741	Risk Ratio (M-H, Random, 95% CI)	0%	0.5	1.30 [0.59, 2.87]	0.52
**Acute Kidney Injury Stage 3**	**Overall**		63	20817	Risk Ratio (M-H, Random, 95% CI)	0%	1	0.97 [0.83, 1.12]	0.66
	Author	None	31	6250	Risk Ratio (M-H, Random, 95% CI)	0%	1	1.01 [0.77, 1.33]	0.93
		Unclear	28	4496	Risk Ratio (M-H, Random, 95% CI)	0%	0.92	0.87 [0.61, 1.25]	0.46
		Any	4	10071	Risk Ratio (M-H, Random, 95% CI)	0%	0.52	0.97 [0.80, 1.19]	0.8
	Author Type	Not stated	59	8843	Risk Ratio (M-H, Random, 95% CI)	0%	1	0.90 [0.70, 1.17]	0.45
		Non-Profit	2	6634	Risk Ratio (M-H, Random, 95% CI)	0%	0.8	1.05 [0.84, 1.31]	0.7
		Blood service	0	0	Risk Ratio (M-H, Random, 95% CI)	N/A	N/A	Not estimable	N/A
		Professional advocacy organisation	4	636	Risk Ratio (M-H, Random, 95% CI)	57%	0.1	0.85 [0.51, 1.41]	0.53
		Industry	2	5340	Risk Ratio (M-H, Random, 95% CI)	4%	0.31	0.92 [0.69, 1.23]	0.58
	Funding	None	25	6135	Risk Ratio (M-H, Random, 95% CI)	0%	1	1.02 [0.79, 1.32]	0.87
		Unclear	21	2728	Risk Ratio (M-H, Random, 95% CI)	0%	0.75	0.81 [0.48, 1.34]	0.41
		Any	17	11954	Risk Ratio (M-H, Random, 95% CI)	0%	0.94	0.96 [0.79, 1.17]	0.7

	Funding Type	Not stated	41	5706	Risk Ratio (M-H, Random, 95% CI)	0%	0.99	0.92 [0.68, 1.24]	0.58
		Non-Profit	13	9004	Risk Ratio (M-H, Random, 95% CI)	0%	0.97	1.02 [0.82, 1.26]	0.89
		Blood service	4	5194	Risk Ratio (M-H, Random, 95% CI)	0%	0.73	0.87 [0.64, 1.20]	0.4
		Professional advocacy organisation	4	636	Risk Ratio (M-H, Random, 95% CI)	57%	0.1	0.85 [0.51, 1.41]	0.53
		Industry	5	913	Risk Ratio (M-H, Random, 95% CI)	0%	0.59	1.15 [0.65, 2.01]	0.64
**Acute Brain Injury**	**Overall**		94	27680	Risk Ratio (M-H, Random, 95% CI)	0%	1	1.00 [0.87, 1.15]	1
	Author	None	43	8925	Risk Ratio (M-H, Random, 95% CI)	0%	0.94	1.06 [0.88, 1.26]	0.55
		Unclear	44	6445	Risk Ratio (M-H, Random, 95% CI)	0%	0.96	0.98 [0.69, 1.38]	0.89
		Any	7	12310	Risk Ratio (M-H, Random, 95% CI)	0%	0.72	0.90 [0.68, 1.20]	0.47
	Author Type	Not stated	85	13329	Risk Ratio (M-H, Random, 95% CI)	0%	0.99	0.94 [0.73, 1.22]	0.66
		Non-Profit	4	8688	Risk Ratio (M-H, Random, 95% CI)	6%	0.36	1.04 [0.87, 1.25]	0.65
		Blood service	1	83	Risk Ratio (M-H, Random, 95% CI)	N/A	N/A	3.07 [0.13, 73.29]	0.49
		Professional advocacy organisation	4	641	Risk Ratio (M-H, Random, 95% CI)	0%	0.79	1.20 [0.47, 3.08]	0.71
		Industry	4	5580	Risk Ratio (M-H, Random, 95% CI)	0%	0.77	0.95 [0.65, 1.37]	0.77
	Funding	None	36	7536	Risk Ratio (M-H, Random, 95% CI)	0%	0.98	1.05 [0.88, 1.26]	0.57
		Unclear	35	3774	Risk Ratio (M-H, Random, 95% CI)	0%	0.81	0.80 [0.53, 1.21]	0.3
		Any	23	16370	Risk Ratio (M-H, Random, 95% CI)	0%	0.92	0.99 [0.76, 1.28]	0.92
	Funding Type	Not stated	60	7534	Risk Ratio (M-H, Random, 95% CI)	0%	0.95	0.87 [0.64, 1.17]	0.34
		Non-Profit	21	11715	Risk Ratio (M-H, Random, 95% CI)	0%	0.86	1.05 [0.88, 1.25]	0.58
		Blood service	5	6916	Risk Ratio (M-H, Random, 95% CI)	0%	0.54	1.02 [0.71, 1.47]	0.92
		Professional advocacy organisation	4	641	Risk Ratio (M-H, Random, 95% CI)	0%	0.79	1.20 [0.47, 3.08]	0.71
		Industry	8	1515	Risk Ratio (M-H, Random, 95% CI)	0%	0.94	1.01 [0.46, 2.24]	0.97

**Sepsis and Infection**	**Overall**		126	29814	Risk Ratio (M-H, Random, 95% CI)	9%	0.24	0.97 [0.91, 1.03]	0.32
	Author	None	60	9214	Risk Ratio (M-H, Random, 95% CI)	3%	0.42	0.96 [0.88, 1.05]	0.4
		Unclear	51	6539	Risk Ratio (M-H, Random, 95% CI)	0%	0.48	0.95 [0.83, 1.10]	0.52
		Any	15	14061	Risk Ratio (M-H, Random, 95% CI)	46%	0.03	0.99 [0.89, 1.09]	0.77
	Author Type	Not stated	110	13902	Risk Ratio (M-H, Random, 95% CI)	0%	0.52	0.93 [0.83, 1.03]	0.18
		Non-Profit	6	8916	Risk Ratio (M-H, Random, 95% CI)	21%	0.27	0.97 [0.88, 1.06]	0.46
		Blood service	1	503	Risk Ratio (M-H, Random, 95% CI)	N/A	N/A	0.35 [0.20, 0.61]	**<0.001**
		Professional advocacy organisation	4	872	Risk Ratio (M-H, Random, 95% CI)	41%	0.17	1.01 [0.80, 1.29]	0.9
		Industry	9	6493	Risk Ratio (M-H, Random, 95% CI)	0%	0.72	1.12 [1.00, 1.26]	**0.05**
	Funding	None	35	9264	Risk Ratio (M-H, Random, 95% CI)	11%	0.28	0.95 [0.89, 1.02]	0.14
		Unclear	46	5014	Risk Ratio (M-H, Random, 95% CI)	26%	0.09	0.86 [0.70, 1.07]	0.18
		Any	27	15536	Risk Ratio (M-H, Random, 95% CI)	0%	0.66	1.05 [0.93, 1.19]	0.44
	Funding Type	Not stated	84	9595	Risk Ratio (M-H, Random, 95% CI)	13%	0.21	0.91 [0.80, 1.02]	0.1
		Non-Profit	26	13089	Risk Ratio (M-H, Random, 95% CI)	19%	0.2	0.94 [0.88, 1.02]	0.13
		Blood service	5	5412	Risk Ratio (M-H, Random, 95% CI)	11%	0.34	1.25 [0.99, 1.59]	0.06
		Professional advocacy organisation	4	872	Risk Ratio (M-H, Random, 95% CI)	41%	0.17	1.01 [0.80, 1.29]	0.9
		Industry	11	1718	Risk Ratio (M-H, Random, 95% CI)	0%	0.8	1.14 [0.91, 1.43]	0.27
**Number of red blood cells transfused**	**Overall**		220	38005	Std. Mean Difference (IV, Random, 95% CI)	96%	<0.001	-0.83 [-0.95, -0.70]	**<0.001**
	Author	None	100	13815	Std. Mean Difference (IV, Random, 95% CI)	95%	<0.001	-0.77 [-0.95, -0.59]	**<0.001**
		Unclear	103	9997	Std. Mean Difference (IV, Random, 95% CI)	91%	<0.001	-0.80 [-0.98, -0.61]	**<0.001**
		Any	17	14193	Std. Mean Difference (IV, Random, 95% CI)	99%	<0.001	-1.28 [-1.76, -0.81]	**<0.001**
	Author Type	Not stated	200	21679	Std. Mean Difference (IV, Random, 95% CI)	92%	<0.001	-0.77 [-0.89, -0.64]	**<0.001**

		Non-Profit	7	8954	Std. Mean Difference (IV, Random, 95% CI)	99%	<0.001	-0.79 [-1.77, 0.20]	**<0.001**
		Blood service	4	852	Std. Mean Difference (IV, Random, 95% CI)	91%	<0.001	-0.76 [-1.56, 0.03]	**<0.001**
		Professional advocacy organisation	7	1029	Std. Mean Difference (IV, Random, 95% CI)	51%	0.008	-0.24 [-0.51, 0.03]	**<0.001**
		Industry	9	6520	Std. Mean Difference (IV, Random, 95% CI)	99%	<0.001	-1.75 [-2.47, -1.03]	**<0.001**
	Funding	None	82	11792	Std. Mean Difference (IV, Random, 95% CI)	97%	<0.001	-0.94 [-1.19, -0.69]	**<0.001**
		Unclear	102	8821	Std. Mean Difference (IV, Random, 95% CI)	90%	<0.001	-0.90 [-1.08, -0.72]	**<0.001**
		Any	36	17392	Std. Mean Difference (IV, Random, 95% CI)	98%	<0.001	-0.41 [-0.67, -0.16]	**<0.001**
	Funding Type	Not stated	163	15570	Std. Mean Difference (IV, Random, 95% CI)	93%	<0.001	-0.93 [-1.09, -0.77]	**<0.001**
		Non-Profit	33	13144	Std. Mean Difference (IV, Random, 95% CI)	98%	<0.001	-0.67 [-1.00, -0.34]	**<0.001**
		Blood service	7	7276	Std. Mean Difference (IV, Random, 95% CI)	99%	<0.001	-0.34 [-0.98, 0.29]	**<0.001**
		Professional advocacy organisation	7	1029	Std. Mean Difference (IV, Random, 95% CI)	51%	0.08	-0.24 [-0.51, 0.03]	**<0.001**
		Industry	17	2015	Std. Mean Difference (IV, Random, 95% CI)	90%	<0.001	-0.44 [-0.85, -0.03]	**<0.001**
**Perioperative blood loss**	**Overall**		319	33071	Std. Mean Difference (IV, Random, 95% CI)	77%	<0.001	-1.06 [-1.16, -0.96]	**<0.001**
	Author	None	152	16017	Std. Mean Difference (IV, Random, 95% CI)	94%	<0.001	-1.01 [-1.15, -0.86]	**<0.001**
		Unclear	146	12868	Std. Mean Difference (IV, Random, 95% CI)	95%	<0.001	-1.18 [-1.36, -1.00]	**<0.001**
		Any	21	4186	Std. Mean Difference (IV, Random, 95% CI)	93%	<0.001	-0.74 [-1.01, -0.47]	**<0.001**
	Author Type	Not stated	298	28972	Std. Mean Difference (IV, Random, 95% CI)	94%	<0.001	-1.09 [-1.20, -0.97]	**<0.001**
		Non-Profit	6	2464	Std. Mean Difference (IV, Random, 95% CI)	97%	<0.001	-1.12 [-2.05, -0.19]	**<0.001**
		Blood service	3	152	Std. Mean Difference (IV, Random, 95% CI)	88%	<0.001	-1.80 [-3.01, -0.59]	**0.003**
		Professional advocacy organisation	8	717	Std. Mean Difference (IV, Random, 95% CI)	50%	0.05	-0.27 [-0.49, -0.05]	**0.02**
		Industry	12	1483	Std. Mean Difference (IV, Random, 95% CI)	81%	0.06	-0.39 [-0.64, -0.14]	**0.002**
	Funding	None	137	12680	Std. Mean Difference (IV, Random, 95% CI)	95%	<0.001	-1.10 [-1.27, -0.92]	**<0.001**

		Unclear	133	11049	Std. Mean Difference (IV, Random, 95% CI)	94%	<0.001	-1.15 [-1.33, -0.97]	**<0.001**
		Any	49	9342	Std. Mean Difference (IV, Random, 95% CI)	93%	<0.001	-0.77 [-0.93, -0.60]	**<0.001**
	Funding Type	Not stated	245	23262	Std. Mean Difference (IV, Random, 95% CI)	94%	<0.001	-1.09 [-1.22, -0.97]	**<0.001**
		Non-Profit	52	7488	Std. Mean Difference (IV, Random, 95% CI)	96%	<0.001	-1.12 [-1.38, -0.86]	**<0.001**
		Blood service	3	353	Std. Mean Difference (IV, Random, 95% CI)	91%	<0.001	-0.50 [-1.23, 0.23]	0.18
		Professional advocacy organisation	5	471	Std. Mean Difference (IV, Random, 95% CI)	64%	0.03	-0.19 [-0.53, 0.14]	0.26
		Industry	19	1968	Std. Mean Difference (IV, Random, 95% CI)	91%	<0.001	-0.61 [-0.92, -0.30]	**<0.001**
**Reoperation for bleeding**	**Overall**		81	23239	Risk Ratio (M-H, Random, 95% CI)	0%	0.93	0.85 [0.74, 0.98]	**0.02**
	Author	None	25	5195	Risk Ratio (M-H, Random, 95% CI)	0%	0.52	0.82 [0.60, 1.12]	0.22
		Unclear	48	6047	Risk Ratio (M-H, Random, 95% CI)	0%	1	0.79 [0.62, 1.01]	0.06
		Any	8	11997	Risk Ratio (M-H, Random, 95% CI)	50%	0.05	0.85 [0.53, 1.35]	0.49
	Author Type	Not stated	72	9351	Risk Ratio (M-H, Random, 95% CI)	0%	0.99	0.82 [0.67, 1.00]	**0.05**
		Non-Profit	4	8691	Risk Ratio (M-H, Random, 95% CI)	0%	0.47	0.59 [0.43, 0.81]	**0.001**
		Blood service	2	65	Risk Ratio (M-H, Random, 95% CI)	0%	0.86	3.23 [0.35, 29.49]	0.3
		Professional advocacy organisation	2	205	Risk Ratio (M-H, Random, 95% CI)	0%	0.47	0.55 [0.21, 1.48]	0.24
		Industry	3	5132	Risk Ratio (M-H, Random, 95% CI)	0%	0.53	1.09 [0.86, 1.39]	0.48
	Funding	None	25	5966	Risk Ratio (M-H, Random, 95% CI)	0%	0.98	0.95 [0.72, 1.26]	0.74
		Unclear	37	3443	Risk Ratio (M-H, Random, 95% CI)	0%	0.97	0.78 [0.57, 1.05]	0.1
		Any	19	13830	Risk Ratio (M-H, Random, 95% CI)	32%	0.09	0.69 [0.48, 1.00]	**0.05**
	Funding Type	Not stated	56	6430	Risk Ratio (M-H, Random, 95% CI)	0%	0.98	0.88 [0.70, 1.11]	0.28
		Non-Profit	14	10831	Risk Ratio (M-H, Random, 95% CI)	0%	0.75	0.60 [0.46, 0.78]	**<0.001**
		Blood service	5	5296	Risk Ratio (M-H, Random, 95% CI)	0%	0.87	1.06 [0.84, 1.34]	0.61

		Professional advocacy organisation	2	205	Risk Ratio (M-H, Random, 95% CI)	0%	0.47	0.55 [0.21, 1.48]	0.24
		Industry	6	682	Risk Ratio (M-H, Random, 95% CI)	0%	0.44	1.03 [0.37, 2.87]	0.96
**Risk of receiving fresh frozen plasma**	**Overall**		33	10546	Risk Ratio (M-H, Random, 95% CI)	49%	<0.001	0.74 [0.63, 0.86]	**<0.001**
	Author	None	15	3611	Risk Ratio (M-H, Random, 95% CI)	62%	<0.001	0.72 [0.55, 0.96]	**0.02**
		Unclear	16	1879	Risk Ratio (M-H, Random, 95% CI)	30%	0.12	0.70 [0.52, 0.94]	**0.02**
		Any	2	5056	Risk Ratio (M-H, Random, 95% CI)	0%	0.64	0.87 [0.79, 0.95]	**0.003**
	Author Type	Not stated	30	3487	Risk Ratio (M-H, Random, 95% CI)	27%	0.09	0.68 [0.57, 0.82]	**<0.001**
		Non-Profit	1	2003	Risk Ratio (M-H, Random, 95% CI)	N/A	N/A	1.05 [0.91, 1.20]	0.49
		Blood service	0	0	Risk Ratio (M-H, Random, 95% CI)	N/A	N/A	Not estimable	N/A
		Professional advocacy organisation	2	205	Risk Ratio (M-H, Random, 95% CI)	33%	0.22	0.43 [0.24, 0.76]	**0.004**
		Industry	2	5056	Risk Ratio (M-H, Random, 95% CI)	0%	0.64	0.87 [0.79, 0.95]	**0.003**
	Funding	None	14	1698	Risk Ratio (M-H, Random, 95% CI)	35%	0.1	0.57 [0.41, 0.79]	**<0.001**
		Unclear	13	3273	Risk Ratio (M-H, Random, 95% CI)	53%	0.01	0.77 [0.59, 1.02]	**0.07**
		Any	6	5575	Risk Ratio (M-H, Random, 95% CI)	0%	0.84	0.87 [0.79, 0.95]	**0.003**
	Funding Type	Not stated	18	2155	Risk Ratio (M-H, Random, 95% CI)	37%	0.06	0.67 [0.54, 0.83]	**<0.001**
		Non-Profit	7	2402	Risk Ratio (M-H, Random, 95% CI)	25%	0.24	0.67 [0.37, 1.21]	0.18
		Blood service	4	5180	Risk Ratio (M-H, Random, 95% CI)	0%	0.64	0.87 [0.79, 0.96]	**0.006**
		Professional advocacy organisation	2	205	Risk Ratio (M-H, Random, 95% CI)	33%	0.22	0.43 [0.24, 0.76]	**0.004**
		Industry	4	809	Risk Ratio (M-H, Random, 95% CI)	41%	0.16	0.70 [0.38, 1.26]	0.23
**Risk of receiving Platelets**	**Overall**		29	10129	Risk Ratio (M-H, Random, 95% CI)	18%	0.19	0.88 [0.78, 0.99]	**0.04**
	Author	None	11	3214	Risk Ratio (M-H, Random, 95% CI)	45%	0.05	0.79 [0.59, 1.07]	0.13
		Unclear	16	1859	Risk Ratio (M-H, Random, 95% CI)	0%	0.66	0.77 [0.61, 0.97]	**0.02**

Roman M, *et al. BMJ Open* 2022; 12:e054582. doi: 10.1136/bmjopen-2021-054582

		Any	2	5056	Risk Ratio (M-H, Random, 95% CI)	0%	1	0.98 [0.90, 1.07]	0.61
	Author Type	Not stated	26	3073	Risk Ratio (M-H, Random, 95% CI)	0%	0.55	0.74 [0.63, 0.88]	**<0.001**
		Non-Profit	1	2000	Risk Ratio (M-H, Random, 95% CI)	N/A	N/A	1.04 [0.93, 1.16]	0.52
		Blood service	0	0	Risk Ratio (M-H, Random, 95% CI)	N/A	N/A	Not estimable	N/A
		Professional advocacy organisation	2	205	Risk Ratio (M-H, Random, 95% CI)	54%	0.14	0.69 [0.38, 1.27]	0.23
		Industry	2	5056	Risk Ratio (M-H, Random, 95% CI)	0%	1	0.98 [0.90, 1.07]	0.61
	Funding	None	11	3016	Risk Ratio (M-H, Random, 95% CI)	50%	0.03	0.76 [0.55, 1.03]	0.08
		Unclear	12	1538	Risk Ratio (M-H, Random, 95% CI)	0%	0.55	0.80 [0.62, 1.04]	0.09
		Any	6	5575	Risk Ratio (M-H, Random, 95% CI)	0%	0.75	0.97 [0.89, 1.06]	0.5
	Funding Type	Not stated	17	1946	Risk Ratio (M-H, Random, 95% CI)	1%	0.44	0.75 [0.63, 0.90]	**0.002**
		Non-Profit	5	2506	Risk Ratio (M-H, Random, 95% CI)	41%	0.15	0.49 [0.17, 1.43]	0.19
		Blood service	4	5180	Risk Ratio (M-H, Random, 95% CI)	0%	078	0.97 [0.89, 1.06]	0.54
		Professional advocacy organisation	2	205	Risk Ratio (M-H, Random, 95% CI)	54%	0.14	0.69 [0.38, 1.27]	0.23
		Industry	3	497	Risk Ratio (M-H, Random, 95% CI)	0%	0.39	0.92 [0.53, 1.59]	0.76
**Intensive care length of stay**	**Overall**		57	20096	Mean Difference (IV, Random, 95% CI)	90%	<0.001	-0.13 [-0.20, -0.06]	**<0.001**
	Author	None	26	4994	Mean Difference (IV, Random, 95% CI)	0%	0.99	-0.03 [-0.07, 0.00	**0.05**
		Unclear	26	4568	Mean Difference (IV, Random, 95% CI)	92%	<0.001	-0.29 [-0.41, -0.18]	**<0.001**
		Any	5	10534	Mean Difference (IV, Random, 95% CI)	98%	<0.001	0.32 [-0.42, 1.07]	0.39
	Author Type	Not stated	120	17032	Mean Difference (IV, Random, 95% CI)	84%	<0.001	-0.36 [-0.47, -0.25]	**<0.001**
		Non-Profit	7	6181	Mean Difference (IV, Random, 95% CI)	44%	0.15	-0.27 [-2.28, 1.74]	0.51
		Blood service	2	301	Mean Difference (IV, Random, 95% CI)	N/A	N/A	-0.30 [-0.79, 0.18]	0.78
		Professional advocacy organisation	5	828	Mean Difference (IV, Random, 95% CI)	0%	0.39	0.03 [-0.46, 0.52]	0.84

		Industry	10	6717	Mean Difference (IV, Random, 95% CI)	0%	0.97	-0.01 [-0.09, 0.07]	**<0.001**
	Funding	None	27	6172	Mean Difference (IV, Random, 95% CI)	36%	0.04	-0.06 [-0.12, 0.00]	0.06
		Unclear	14	1850	Mean Difference (IV, Random, 95% CI)	91%	<0.001	-0.41 [-0.75, -0.07]	**0.02**
		Any	16	12074	Mean Difference (IV, Random, 95% CI)	95%	<0.001	0.03 [-0.08, 0.13]	0.6
	Funding Type	Not stated	33	4675	Mean Difference (IV, Random, 95% CI)	88%	<0.001	-0.26 [-0.38, -0.13]	**<0.001**
		Non-Profit	15	9214	Mean Difference (IV, Random, 95% CI)	43%	0.04	-0.07 [-0.12, -0.02]	**0.005**
		Blood service	3	5242	Mean Difference (IV, Random, 95% CI)	99%	<0.001	0.29 [-0.43, 1.02]	0.42
		Professional advocacy organisation	2	506	Mean Difference (IV, Random, 95% CI)	0%	0.32	0.35 [-0.43, 1.14]	0.38
		Industry	6	965	Mean Difference (IV, Random, 95% CI)	0%	0.71	-0.04 [-0.40, 0.33]	0.85
**Hospital length of stay**	**Overall**		139	30231	Mean Difference (IV, Random, 95% CI)	87%	<0.001	-0.38 [-0.50, -0.26]	**<0.001**
	Author	None	75	11342	Mean Difference (IV, Random, 95% CI)	84%	<0.001	-0.25 [-0.40, -0.10]	**0.001**
		Unclear	47	6864	Mean Difference (IV, Random, 95% CI)	74%	<0.001	-0.51 [-0.71, -0.31]	**<0.001**
		Any	17	12025	Mean Difference (IV, Random, 95% CI)	96%	<0.001	-0.61 [-1.17, -0.05]	**0.03**
	Author Type	Not stated	49	7455	Mean Difference (IV, Random, 95% CI)	79%	<0.001	-0.17 [-0.24, -0.10]	**<0.001**
		Non-Profit	4	6738	Mean Difference (IV, Random, 95% CI)	98%	<0.001	-0.06 [-0.25, 0.12]	**<0.001**
		Blood service	1	218	Mean Difference (IV, Random, 95% CI)	0%	0.42	-0.20 [-1.58, 1.18]	0.22
		Professional advocacy organisation	3	606	Mean Difference (IV, Random, 95% CI)	38%	0.17	0.05 [-0.42, 0.52]	0.91
		Industry	3	5685	Mean Difference (IV, Random, 95% CI)	0%	0.77	0.80 [0.68, 0.92]	0.81
	Funding	None	67	11729	Mean Difference (IV, Random, 95% CI)	84%	<0.001	-0.27 [-0.41, -0.13]	**<0.001**
		Unclear	47	5325	Mean Difference (IV, Random, 95% CI)	73%	<0.001	-0.47 [-0.73, -0.20]	**<0.001**
		Any	25	13177	Mean Difference (IV, Random, 95% CI)	95%	<0.001	-0.57 [-0.94, -0.20]	**0.003**
	Funding Type	Not stated	93	11276	Mean Difference (IV, Random, 95% CI)	81%	<0.001	-0.43 [-0.56, -0.30]	**<0.001**

		Non-Profit	30	10347	Mean Difference (IV, Random, 95% CI)	94%	<0.001	-0.33 [-0.68, 0.03]	0.07
		Blood service	6	7134	Mean Difference (IV, Random, 95% CI)	0%	0.47	-0.02 [-0.10, 0.07]	0.73
		Professional advocacy organisation	3	656	Mean Difference (IV, Random, 95% CI)	31%	0.24	-1.10 [-2.93, 0.73]	0.24
		Industry	10	1474	Mean Difference (IV, Random, 95% CI)	0%	0.84	0.08 [-0.25, 0.41]	0.63

7 Subgroup analysis based on studies that reported their primary outcome as clinical or transfusion related. (eTable 3)

The results are reported as: Risk Ratio (RR), 95% Confidence Intervals and p-values for dichotomous outcomes and Standardised Mean Difference (SMD), 95% Confidence Intervals and P values for continuous outcomes. The heterogeneity was reported as I^2, with P values. The effects considered were random. P values of <0.05 were considered statistically significant. The colour [green] indicates a statistically significant overall treatment effect when there were significant subgroup differences in favour of the intervention.

Outcome	Subgroup/Moderator	Type	# of studies	Patients (n)	Output measurement type	Test for heterogeneity		Test for effect		Test for subgroup differences		Test for overall effect
						I^2	P value	Result	P value	Chi^2	P value	P value
Mortality	Type of primary outcome	Clinical	16	11413	Risk Ratio (M-H, Random, 95% CI)	25%	0.18	1.14 [0.88, 1.49]	0.31	4.04	0.04	0.34
		Transfusion related	77	15353	Risk Ratio (M-H, Random, 95% CI)	0%	0.99	0.81 [0.66, 1.00]	0.05			
Myocardial Infarction	Type of primary outcome	Clinical	12	10207	Risk Ratio (M-H, Random, 95% CI)	0%	0.7	1.04 [0.86, 1.27]	0.67	1.43	0.23	0.34
		Transfusion related	42	12207	Risk Ratio (M-H, Random, 95% CI)	0%	0.99	0.90 [0.79, 1.03]	0.14			
Adverse Reactions	Type of primary outcome	Clinical	5	654	Risk Ratio (M-H, Random, 95% CI)	0%	0.45	1.14 [0.73, 1.79]	0.56	1.46	0.23	<0.001
		Transfusion related	107	19538	Risk Ratio (M-H, Random, 95% CI)	0%	0.58	0.86 [0.81, 0.92]	<0.001			
Low Cardiac Output	Type of primary outcome	Clinical	7	5827	Risk Ratio (M-H, Random, 95% CI)	67%	0.006	0.78 [0.44, 1.40]	0.41	0.02	0.88	0.39
		Transfusion related	18	2881	Risk Ratio (M-H, Random, 95% CI)	15%	0.28	0.83 [0.56, 1.22]	0.34			
Acute Kidney Injury	Type of primary outcome	Clinical	7	7634	Risk Ratio (M-H, Random, 95% CI)	0%	0.86	0.94 [0.74, 1.20]	0.62	0.12	0.73	0.66
		Transfusion related	56	13183	Risk Ratio (M-H, Random, 95% CI)	0%	1	0.99 [0.82, 1.20]	0.93			
Acute Brain Injury	Type of primary outcome	Clinical	14	10899	Risk Ratio (M-H, Random, 95% CI)	0%	0.74	1.04 [0.87, 1.23]	0.68	0.41	0.52	1
		Transfusion related	80	16781	Risk Ratio (M-H, Random, 95% CI)	0%	0.99	0.94 [0.74, 1.20]	0.62			
Sepsis and Infection	Type of primary outcome	Clinical	18	11189	Risk Ratio (M-H, Random, 95% CI)	36%	0.08	1.05 [0.93, 1.17]	0.44	3.6	0.06	0.32
		Transfusion related	108	18625	Risk Ratio (M-H, Random, 95% CI)	0%	0.62	0.90 [0.80, 1.00]	0.05			

Roman M, et al. BMJ Open 2022; 12:e054582. doi: 10.1136/bmjopen-2021-054582

**Risk of receiving red cell transfusion**	Type of primary outcome	Clinical	26	12679	Risk Ratio (M-H, Random, 95% CI)	90%	<0.001	0.58 [0.52, 0.66]	<0.001	0.06	0.81	<0.001
		Transfusion related	286	42867	Risk Ratio (M-H, Random, 95% CI)	72%	<0.001	0.59 [0.56, 0.63]	<0.001			
**Number of red cells transfused**	Type of primary outcome	Clinical	14	10881	Std. Mean Difference (IV, Random, 95% CI)	97%	<0.001	-0.96 [-1.34, -0.59]	<0.001	0.55	0.46	<0.001
		Transfusion related	206	27124	Std. Mean Difference (IV, Random, 95% CI)	94%	<0.001	-0.81 [-0.94, -0.69]	<0.001			
**Perioperative blood loss**	Type of primary outcome	Clinical	14	3525	Std. Mean Difference (IV, Random, 95% CI)	96%	<0.001	-1.01 [-1.45, -0.58]	<0.001	0.04	0.84	<0.001
		Transfusion related	305	29546	Std. Mean Difference (IV, Random, 95% CI)	94%	<0.001	-1.06 [-1.17, -0.95]	<0.001			
**Re-operation for bleeding**	Type of primary outcome	Clinical	8	9921	Risk Ratio (M-H, Random, 95% CI)	0%	0.68	1.05 [0.86, 1.28]	0.65	7.71	0.005	0.02
		Transfusion related	73	13406	Risk Ratio (M-H, Random, 95% CI)	0%	0.98	0.71 [0.59, 0.85]	<0.001			
**Risk of receiving Fresh Frozen Plasma**	Type of primary outcome	Clinical	4	7233	Risk Ratio (M-H, Random, 95% CI)	70%	0.02	0.92 [0.73, 1.16]	0.48	3.9	0.05	<0.001
		Transfusion related	29	3313	Risk Ratio (M-H, Random, 95% CI)	23%	0.14	0.69 [0.58, 0.82]	<0.001			
**Risk of receiving Platelets**	Type of primary outcome	Clinical	4	7230	Risk Ratio (M-H, Random, 95% CI)	16%	0.31	1.00 [0.91, 1.09]	0.99	8.44	0.004	0.04
		Transfusion related	25	2899	Risk Ratio (M-H, Random, 95% CI)	0%	0.61	0.76 [0.64, 0.89]	<0.001			
**Intensive care unit length of stay**	Type of primary outcome	Clinical	15	9324	Mean Difference (IV, Random, 95% CI)	92%	<0.001	0.05 [-0.23, 0.34]	0.71	2.52	0.11	<0.001
		Transfusion related	42	10772	Mean Difference (IV, Random, 95% CI)	88%	<0.001	-0.18 [-0.25, -0.12]	<0.001			
**Hospital length of stay**	Type of primary outcome	Clinical	21	9485	Mean Difference (IV, Random, 95% CI)	81%	<0.001	0.16 [-0.11, 0.43]	0.24	17.02	<0.001	<0.001
		Transfusion related	118	20746	Mean Difference (IV, Random, 95% CI)	87%	<0.001	-0.47 [-0.61, -0.34]	<0.001			

8 Subgroup analysis for mortality and risk of red blood cells transfusion based on the country of origin of the corresponding author. (eTable 4.)

The results are reported as: Risk Ratio (RR), 95% Confidence Intervals and P values. Heterogeneity is expressed as I^2 and P values. The effects considered were random. P values of <0.05 were considered statistically significant.

Outcome	CoI Moderator	Subtype	# of studies	Patients (n)	Output measurement type	I^2	P value	Result	P value
**30-day mortality**	**Overall**		93	26766	Risk Ratio (M-H, Random, 95% CI)	0%	0.92	0.93 [0.81, 1.07]	0.34
	Country	US	18	4865	Risk Ratio (M-H, Random, 95% CI)	0%	0.83	0.87 [0.66, 1.14]	0.31
		Europe	41	7596	Risk Ratio (M-H, Random, 95% CI)	0%	0.89	1.03 [0.80, 1.32]	0.82
		Other	34	14305	Risk Ratio (M-H, Random, 95% CI)	0%	0.51	0.91 [0.74, 1.12]	0.38
**Risk of receiving red cell transfusion**	**Overall**		312	55546	Risk Ratio (M-H, Random, 95% CI)	77%	<0.001	0.6 [0.57, 0.63]	**<0.001**
	Country	US	35	13527	Risk Ratio (M-H, Random, 95% CI)	89%	<0.001	0.67 [0.58, 0.78]	**<0.001**
		Europe	112	15567	Risk Ratio (M-H, Random, 95% CI)	72%	<0.001	0.64 [0.59, 0.69]	**<0.001**
		Other	165	26452	Risk Ratio (M-H, Random, 95% CI)	75%	<0.001	0.54 [0.50, 0.58]	**<0.001**

9 Subgroup analysis for mortality and risk of red blood cells transfusion based on the studies following the International Committee of Medical Journal Editors (ICMJE) guidelines of reporting. (eTable 5.)

The results are reported as: Risk Ratio (RR), 95% Confidence Intervals and P values. Heterogeneity is expressed as I^2 and P values. The effects considered were random. P values of <0.05 were considered statistically significant.

Outcome	CoI Moderator	Subtype	# of studies	Patients (n)	Output measurement type	I^2	P value	Result	P value
**30-day mortality**	**Overall**		93	26766	Risk Ratio (M-H, Random, 95% CI)	0%	0.92	0.93 [0.81, 1.07]	0.34
	ICMJE	Yes	3	8875	Risk Ratio (M-H, Random, 95% CI)	13%	0.31	0.91 [0.71, 1.16]	0.46
		No	90	17891	Risk Ratio (M-H, Random, 95% CI)	0%	0.91	0.95 [0.80, 1.14]	0.6
**Risk of receiving red cell transfusion**	**Overall**		312	55546	Risk Ratio (M-H, Random, 95% CI)	77%	<0.001	0.60 [0.57, 0.63]	**<0.001**
	ICMJE	Yes	14	10061	Risk Ratio (M-H, Random, 95% CI)	92%	<0.001	0.51 [0.40, 0.64]	**<0.001**
		No	298	45485	Risk Ratio (M-H, Random, 95% CI)	73%	<0.001	0.60 [0.57, 0.63]	**<0.001**

10 Subgroup analysis for mortality and risk of red blood cells transfusion based on studies being published prior or after 2010 (Epoch) (eTable 6.)

The results are reported as: Risk Ratio (RR), 95% Confidence Intervals and P values. Heterogeneity is expressed as I^2 and P values. The effects considered were random. P values of <0.05 were considered statistically significant.

Outcome	CoI Moderator	Subtype	# of studies	Patients (n)	Output measurement type	I²	P value	Result	P value
**30-day mortality**	**Overall**		93	26766	Risk Ratio (M-H, Random, 95% CI)	0%	0.92	0.93 [0.81, 1.07]	0.34
	Year	<2010	52	21963	Risk Ratio (M-H, Random, 95% CI)	0%	0.63	0.97 [0.83, 1.12]	0.64
		>2010	41	4803	Risk Ratio (M-H, Random, 95% CI)	0%	0.97	0.74 [0.50, 1.10]	0.14
**Risk of receiving red cell transfusion**	**Overall**		312	55546	Risk Ratio (M-H, Random, 95% CI)	77%	<0.001	0.60 [0.57, 0.63]	**<0.001**
	Year	<2010	204	44237	Risk Ratio (M-H, Random, 95% CI)	76%	<0.001	0.60 [0.56, 0.63]	**<0.001**
		>2010	108	11309	Risk Ratio (M-H, Random, 95% CI)	73%	<0.001	0.61 [0.56, 0.67]	**<0.001**

11 Hidden Conflict of Interest. (eTable 7.)

The authors of included manuscripts were cross-checked with manuscripts previously published by these authors and included in this analysis. The declaration for author and funding conflicts of interest were compiled and used in the sensitivity analysis.

Manuscripts with Hidden COI	Type (Author/Funding)	Changed From	Changed To	Manuscript where CoI identified
**Benoni 1996**	Funding	None	Non-Profit	Elawad 1991
**Boylan 1996**	Funding	Unclear	Industry	Karski 1995
**Claeys 2007**	Funding	Unclear	Industry	Jansen 1999
**Eftekharian 2014**	Funding	Unclear	Non-Profit	Farrokhi 2011
**Horstmann 2014**	Funding	Unclear	Non-Profit	Horstmann 2013
**Karski 2005**	Funding	Non Profit	Industry	Karski 2005
**Liang 2016**	Funding	Unclear	Non-Profit	Liang 2014
**Lidder 2007**	Funding	Unclear	Industry	Edwards 2009
**Lin 2012**	Funding	None	Non-Profit	Lin 2011
**Nuttall 2001**	Funding	Unclear	Industry	Nuttall 2000
**Painter 2018**	Both	Unclear/None	Non-Profit	Myles 2017, Mazer 2017
**Peters 2015**	Author	None	Industry	Verma 2014
**Taghaddomi 2009b**	Funding	Unclear	Non-Profit	Taghaddomi 2009a
**Tengberg 2016**	Funding	None	Non-Profit	Foss 2009
**Wang 2019**	Funding	Unclear	Non-Profit	Zeng 2017
**Xu 2019**	Funding	None	Non-Profit	Shi 2013, Wang 2012
**Yen 2017**	Funding	None	Non-Profit	Lin 2011

Roman M, *et al. BMJ Open* 2022; 12:e054582. doi: 10.1136/bmjopen-2021-054582

12 Sensitivity analysis for mortality and risk of red blood cells transfusion for studies re-classified based on potential undeclared conflicts of interest. (eTable 8.)

The Undeclared Author Conflicts of Interest was assessed by cross-checking each manuscript author with previous studies included in this analysis for declared Conflict of Interests. Where a Conflict of Interest had not been declared within 5 years of a declaration by that author in another trial these were considered Undeclared Conflict of Interest. The definition of Author Conflict of Interest were then recalibrated to include these revised classification and the analysis for the primary outcomes was repeated. The results are reported as: Risk Ratio (RR), 95% Confidence Intervals and P values. Heterogeneity is expressed as I^2 and P values. The effects considered were random. P values of <0.05 were considered statistically significant.

Outcome	CoI Moderator	Subtype	# of studies	Patients (n)	Output measurement type	I^2	P value	Result	P value
**30-day mortality**	**Overall**		93	26766	Risk Ratio (M-H, Random, 95% CI)	0%	0.92	0.93 [0.81, 1.07]	0.34
	Author	None	33	6732	Risk Ratio (M-H, Random, 95% CI)	0%	0.78	1.12 [0.86, 1.45]	0.39
		Unclear	49	6354	Risk Ratio (M-H, Random, 95% CI)	0%	0.8	0.94 [0.7, 1.26]	0.69
		Any	11	13680	Risk Ratio (M-H, Random, 95% CI)	0%	0.83	0.84 [0.69, 1.02]	0.08
	Author Type	Not stated	76	10549	Risk Ratio (M-H, Random, 95% CI)	0%	0.96	1.06 [0.86, 1.31]	0.58
		Non-Profit	5	8831	Risk Ratio (M-H, Random, 95% CI)	13%	0.33	0.89 [0.65, 1.21]	0.44
		Blood service	2	721	Risk Ratio (M-H, Random, 95% CI)	0%	0.58	0.17 [0.02, 1.51]	0.11
		Professional advocacy organisation	5	977	Risk Ratio (M-H, Random, 95% CI)	0%	0.62	0.4 [0.17, 0.92]	**0.03**
		Industry	5	5688	Risk Ratio (M-H, Random, 95% CI)	0%	0.66	0.9 [0.69, 1.17]	0.43
	Funding	None	27	7164	Risk Ratio (M-H, Random, 95% CI)	0%	0.96	1.04 [0.79, 1.36]	0.8
		Unclear	36	3961	Risk Ratio (M-H, Random, 95% CI)	0%	0.5	1.06 [0.79, 1.41]	0.7
		Any	30	15641	Risk Ratio (M-H, Random, 95% CI)	0%	0.79	0.84 [0.69, 1.02]	0.08
	Funding Type	Not stated	49	6273	Risk Ratio (M-H, Random, 95% CI)	0%	0.97	1.02 [0.80, 1.31]	0.87
		Non-Profit	25	12930	Risk Ratio (M-H, Random, 95% CI)	0%	0.65	0.96 [0.77, 1.20]	0.74
		Blood service	4	5244	Risk Ratio (M-H, Random, 95% CI)	0%	0.44	0.86 [0.64, 1.16]	0.34
		Professional advocacy organisation	4	761	Risk Ratio (M-H, Random, 95% CI)	0%	0.45	0.40 [0.17, 0.96]	**0.04**
		Industry	11	1558	Risk Ratio (M-H, Random, 95% CI)	14%	0.31	0.87 [0.44, 1.73]	0.7

**Risk of receiving red cell transfusion**	**Overall**		312	55546	Risk Ratio (M-H, Random, 95% CI)	77%	<0.001	0.6 [0.57, 0.63]	**<0.001**
	Author	None	147	25961	Risk Ratio (M-H, Random, 95% CI)	76%	<0.001	0.59 [0.55, 0.63]	**<0.001**
		Unclear	138	14285	Risk Ratio (M-H, Random, 95% CI)	71%	<0.001	0.61 [0.56, 0.66]	**<0.001**
		Any	27	15300	Risk Ratio (M-H, Random, 95% CI)	88%	<0.001	0.54 [0.45, 0.64]	**<0.001**
	Author Type	Not stated	282	38190	Risk Ratio (M-H, Random, 95% CI)	74%	<0.001	0.59 [0.56, 0.63]	**<0.001**
		Non-Profit	11	9308	Risk Ratio (M-H, Random, 95% CI)	93%	<0.001	0.56 [0.44, 0.7]	**<0.001**
		Blood service	6	975	Risk Ratio (M-H, Random, 95% CI)	60%	0.003	0.58 [0.42, 0.79]	**<0.001**
		Professional advocacy organisation	8	1140	Risk Ratio (M-H, Random, 95% CI)	21%	0.26	0.79 [0.69, 0.91]	**<0.001**
		Industry	13	7073	Risk Ratio (M-H, Random, 95% CI)	42%	0.06	0.65 [0.55, 0.76]	**<0.001**
	Funding	None	118	23009	Risk Ratio (M-H, Random, 95% CI)	72%	<0.001	0.59 [0.55, 0.64]	**<0.001**
		Unclear	128	11718	Risk Ratio (M-H, Random, 95% CI)	82%	<0.001	0.57 [0.52, 0.63]	**<0.001**
		Any	66	20819	Risk Ratio (M-H, Random, 95% CI)	77%	<0.001	0.62 [0.56, 0.66]	**<0.001**
	Funding Type	Not stated	216	28737	Risk Ratio (M-H, Random, 95% CI)	77%	<0.001	0.57 [0.53, 0.61]	**<0.001**
		Non-Profit	64	16785	Risk Ratio (M-H, Random, 95% CI)	79%	<0.001	0.60 [0.54, 0.66]	**<0.001**
		Blood service	8	7356	Risk Ratio (M-H, Random, 95% CI)	46%	0.07	0.75 [0.65, 0.87]	**<0.001**
		Professional advocacy organisation	7	1029	Risk Ratio (M-H, Random, 95% CI)	0%	0.5	0.82 [0.75, 0.90]	**<0.001**
		Industry	24	2668	Risk Ratio (M-H, Random, 95% CI)	49%	0.004	0.67 [0.57, 0.79]	**<0.001**

13 Sensitivity analysis for mortality and risk of red blood cells transfusion excluding all studies considered at high or unclear risk of selection (allocation) bias (eTable 9.)
The results are reported as: Risk Ratio (RR), 95% Confidence Intervals and P values. Heterogeneity is expressed as I^2 and P values. The effects considered were random. P values of <0.05 were considered statistically significant.

Outcome	CoI Moderator	Subtype	# of studies	Patients (n)	Output measurement type	I^2	P value	Result	P value
**30-day mortality**	**Overall**		51	20973	Risk Ratio (M-H, Random, 95% CI)	0%	0.59	0.95 [0.82, 1.12]	0.56
	Author	None	16	4424	Risk Ratio (M-H, Random, 95% CI)	0%	0.63	1.23 [0.89, 1.69]	0.2
		Unclear	27	3572	Risk Ratio (M-H, Random, 95% CI)	0%	0.52	1.09 [0.76, 1.58]	0.64
		Any	8	12977	Risk Ratio (M-H, Random, 95% CI)	0%	0.73	0.82 [0.67, 1.01]	0.06
	Author Type	Not stated	38	5500	Risk Ratio (M-H, Random, 95% CI)	0%	0.82	1.06 [0.86, 1.31]	0.15
		Non-Profit	3	8650	Risk Ratio (M-H, Random, 95% CI)	17%	0.3	0.89 [0.65, 1.21]	0.6
		Blood service	1	503	Risk Ratio (M-H, Random, 95% CI)	N/A	N/A	0.17 [0.02, 1.51]	0.12
		Professional advocacy organisation	5	977	Risk Ratio (M-H, Random, 95% CI)	0%	0.62	0.4 [0.17, 0.92]	**0.03**
		Industry	4	5343	Risk Ratio (M-H, Random, 95% CI)	0%	0.58	0.9 [0.69, 1.17]	0.32
	Funding	None	17	4782	Risk Ratio (M-H, Random, 95% CI)	0%	0.81	1.09 [0.78, 1.53]	0.61
		Unclear	19	2178	Risk Ratio (M-H, Random, 95% CI)	30%	0.13	1.02 [0.60, 1.72]	0.95
		Any	15	14013	Risk Ratio (M-H, Random, 95% CI)	0%	0.9	0.84 [0.69, 1.03]	0.1
	Funding Type	Not stated	26	3370	Risk Ratio (M-H, Random, 95% CI)	0%	0.6	1.18 [0.85, 1.62]	0.33
		Non-Profit	13	10801	Risk Ratio (M-H, Random, 95% CI)	0%	0.62	0.95 [0.75, 1.22]	0.71
		Blood service	3	5026	Risk Ratio (M-H, Random, 95% CI)	15%	0.31	0.96 [0.46, 2.03]	0.92
		Professional advocacy organisation	4	761	Risk Ratio (M-H, Random, 95% CI)	0%	0.45	0.40 [0.17, 0.96]	**0.04**
		Industry	5	1015	Risk Ratio (M-H, Random, 95% CI)	0%	0.47	1.03 [0.52, 2.06]	0.93
**Risk of receiving red cell transfusion**	**Overall**		133	30169	Risk Ratio (M-H, Random, 95% CI)	76%	<0.001	0.61 [0.57, 0.66]	**<0.001**

	Author	None	72	11526	Risk Ratio (M-H, Random, 95% CI)	71%	<0.001	0.58 [0.52, 0.65]	**<0.001**
		Unclear	48	5239	Risk Ratio (M-H, Random, 95% CI)	64%	<0.001	0.65 [0.57, 0.73]	**<0.001**
		Any	13	13404	Risk Ratio (M-H, Random, 95% CI)	93%	<0.001	0.59 [0.48, 0.72]	**<0.001**
	Author Type	Not stated	119	14849	Risk Ratio (M-H, Random, 95% CI)	69%	<0.001	0.59 [0.56, 0.63]	**<0.001**
		Non-Profit	5	8816	Risk Ratio (M-H, Random, 95% CI)	97%	<0.001	0.56 [0.44, 0.7]	**<0.001**
		Blood service	2	543	Risk Ratio (M-H, Random, 95% CI)	0%	0.85	0.58 [0.42, 0.79]	**<0.001**
		Professional advocacy organisation	5	977	Risk Ratio (M-H, Random, 95% CI)	1%	0.4	0.79 [0.69, 0.91]	**<0.001**
		Industry	7	5961	Risk Ratio (M-H, Random, 95% CI)	13%	0.33	0.65 [0.55, 0.76]	**<0.001**
	Funding	None	57	8679	Risk Ratio (M-H, Random, 95% CI)	75%	<0.001	0.62 [0.55, 0.69]	**<0.001**
		Unclear	43	4168	Risk Ratio (M-H, Random, 95% CI)	68%	<0.001	0.53 [0.45, 0.63]	**<0.001**
		Any	33	17322	Risk Ratio (M-H, Random, 95% CI)	85%	<0.001	0.66 [0.58, 0.75]	**<0.001**
	Funding Type	Not stated	83	8774	Risk Ratio (M-H, Random, 95% CI)	72%	<0.001	0.57 [0.53, 0.61]	**<0.001**
		Non-Profit	34	13001	Risk Ratio (M-H, Random, 95% CI)	85%	<0.001	0.60 [0.54, 0.66]	**<0.001**
		Blood service	5	6887	Risk Ratio (M-H, Random, 95% CI)	49%	0.09	0.75 [0.65, 0.87]	**0.003**
		Professional advocacy organisation	5	977	Risk Ratio (M-H, Random, 95% CI)	1%	0.4	0.82 [0.75, 0.90]	**<0.001**
		Industry	11	1507	Risk Ratio (M-H, Random, 95% CI)	33%	0.14	0.67 [0.57, 0.79]	**<0.001**

14 Funnel plots for Mortality and Rate of red blood cells transfusions (eFigure 3.)

Funnel plots (1st figure) and trim and fill (2nd figure) effects were obtained for mortality and risk of red cell transfusions based on the Author and Type of Funding conflicts of interest when each subgroup contained more than 10 trials.

14.1 Mortality - Author COI

None

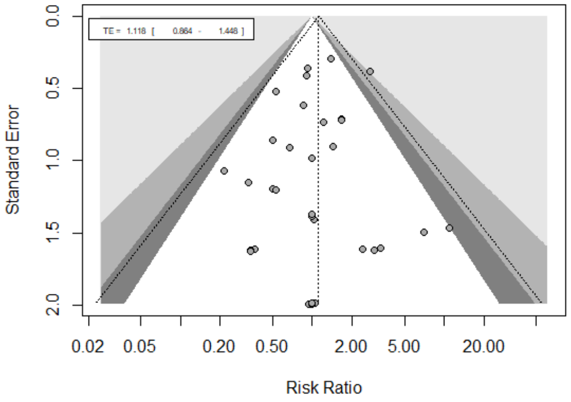

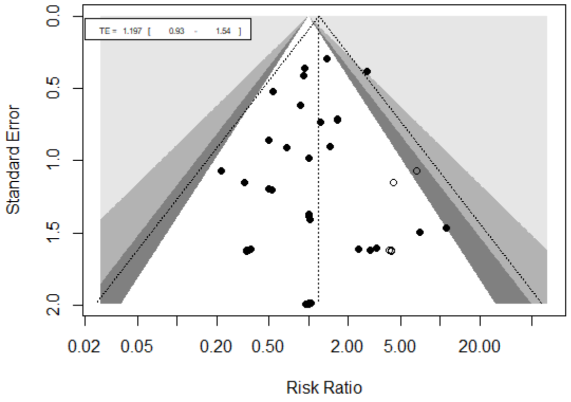

Unclear

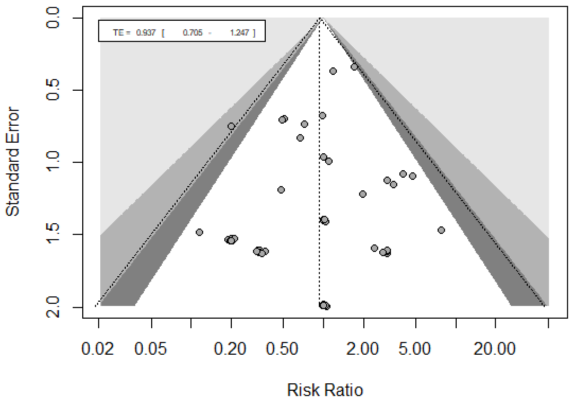
TE = 0.937 [0.705 - 1.247]
Standard Error
0.0
0.5
1.0
1.5
2.0
0.02 0.05 0.20 0.50 2.00 5.00 20.00
Risk Ratio

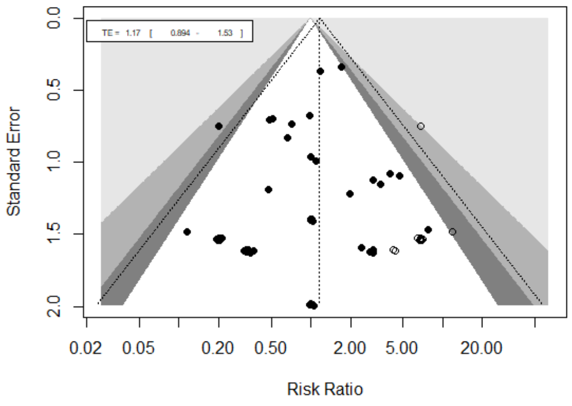
TE = 1.17 [0.894 - 1.53]
Standard Error
0.0
0.5
1.0
1.5
2.0
0.02 0.05 0.20 0.50 2.00 5.00 20.00
Risk Ratio

Any

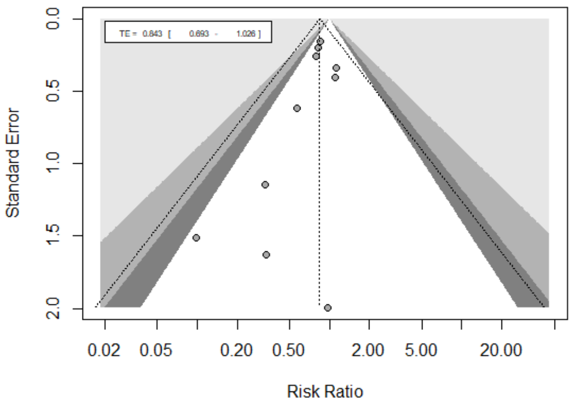
TE = 0.843 [0.693 - 1.026]
Standard Error
0.0
0.5
1.0
1.5
2.0
0.02 0.05 0.20 0.50 2.00 5.00 20.00
Risk Ratio

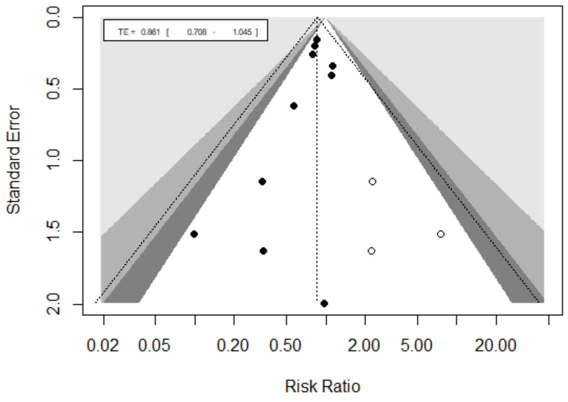
TE = 0.861 [0.708 - 1.045]
Standard Error
0.0
0.5
1.0
1.5
2.0
0.02 0.05 0.20 0.50 2.00 5.00 20.00
Risk Ratio

14.2 Mortality – Type of funding

Not stated

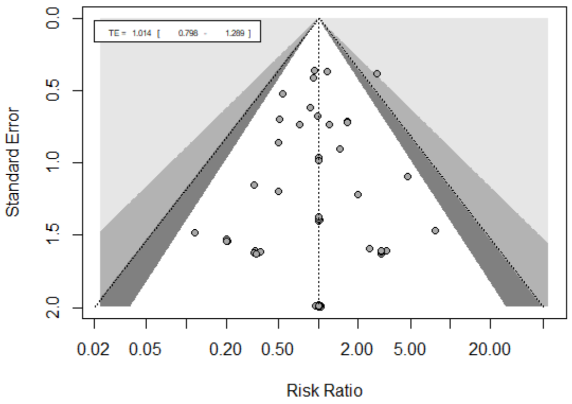

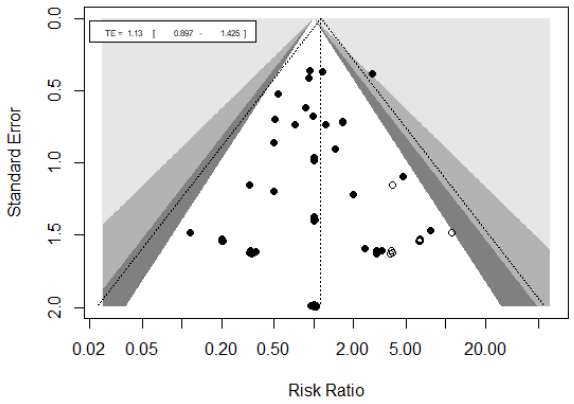

Non-profit

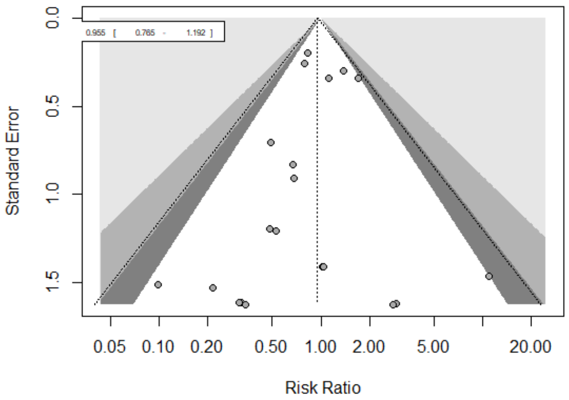

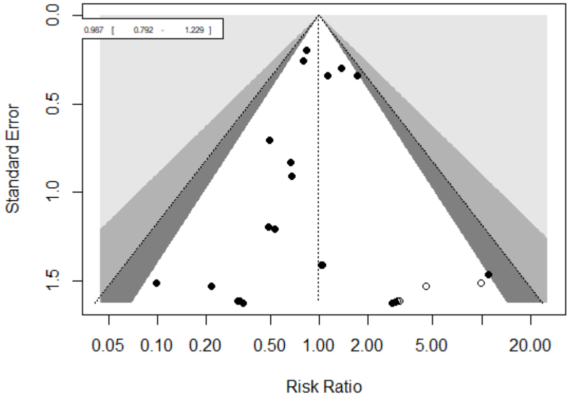

Industry

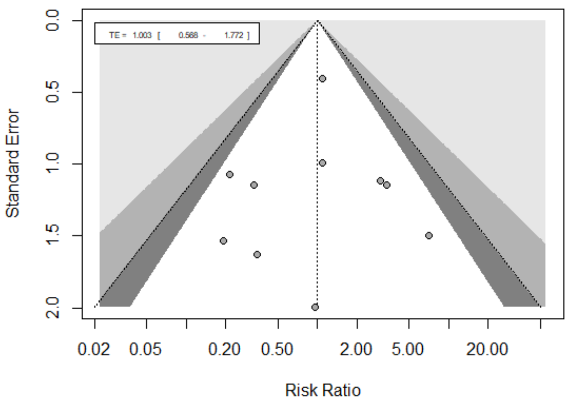
TE = 1.003 [0.568 - 1.772]
Standard Error
0.0
0.5
1.0
1.5
2.0
0.02 0.05 0.20 0.50 2.00 5.00 20.00
Risk Ratio

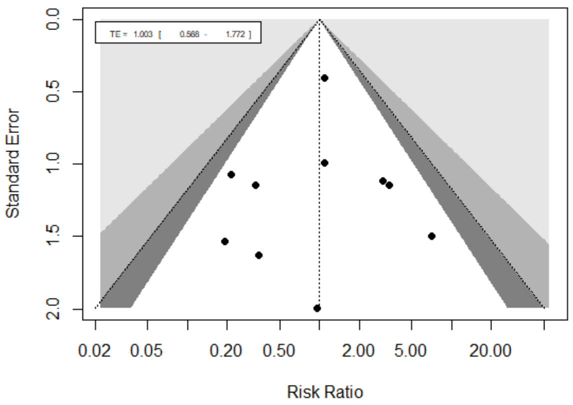
TE = 1.003 [0.568 - 1.772]
Standard Error
0.0
0.5
1.0
1.5
2.0
0.02 0.05 0.20 0.50 2.00 5.00 20.00
Risk Ratio

14.3 Rate of Red blood cells transfusion - Author COI

None

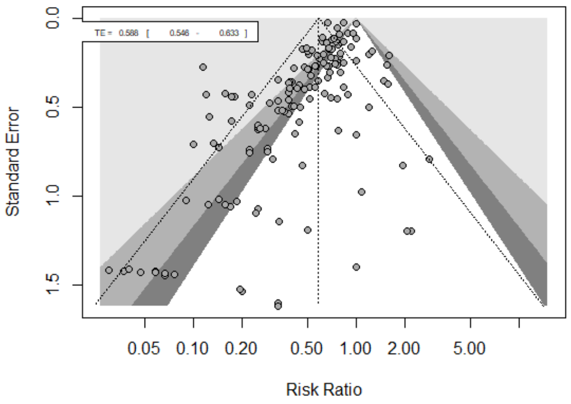

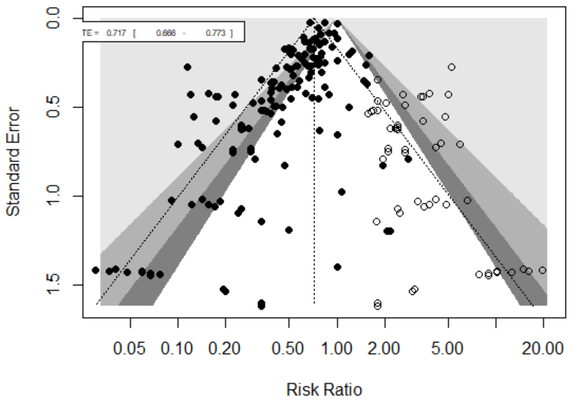

Unclear

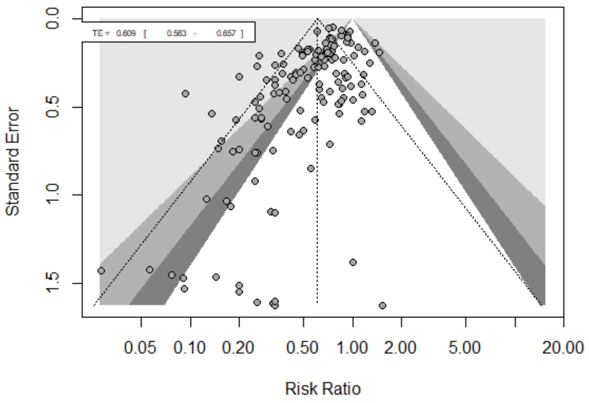

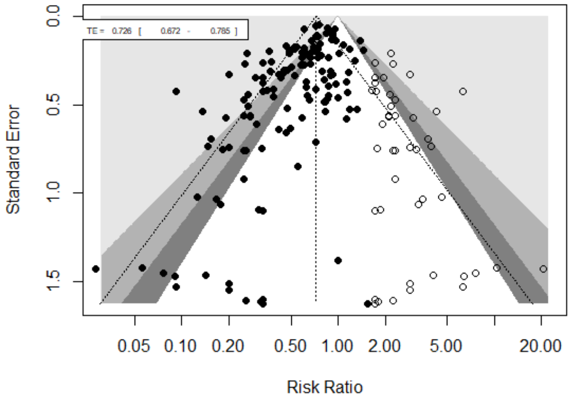

Any

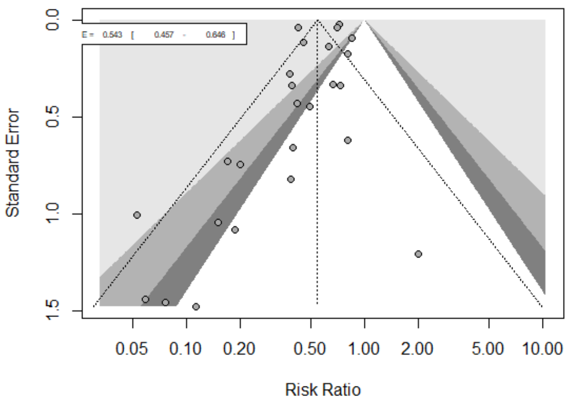
E = 0.543 [0.457 - 0.646]
Standard Error
0.0
0.5
1.0
1.5
0.05
0.10
0.20
0.50
1.00
2.00
5.00
10.00
Risk Ratio

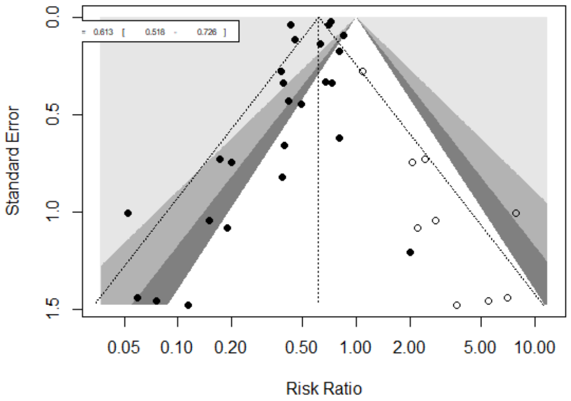
= 0.613 [0.518 - 0.726]
Standard Error
0.0
0.5
1.0
1.5
0.05
0.10
0.20
0.50
1.00
2.00
5.00
10.00
Risk Ratio

14.4 Rate of Red blood cells transfusion - Type of funding

Not stated

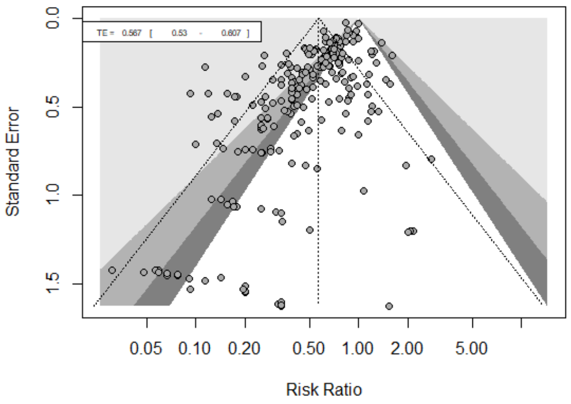

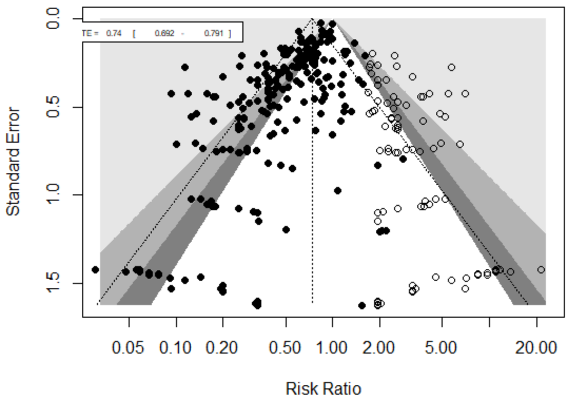

Non-profit

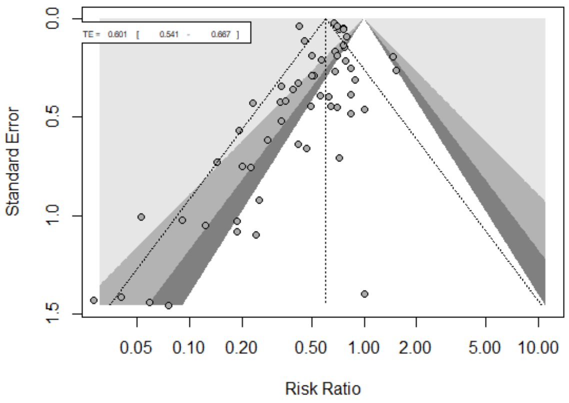

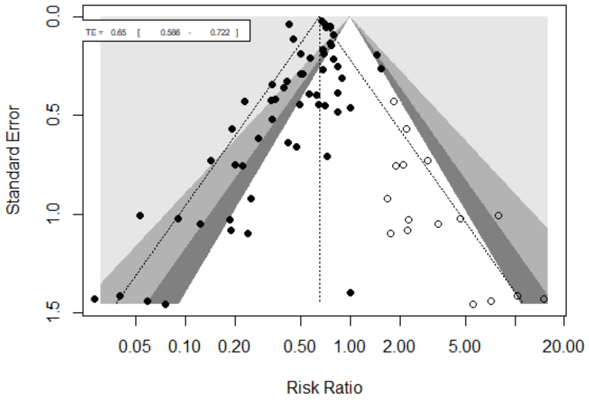

Industry

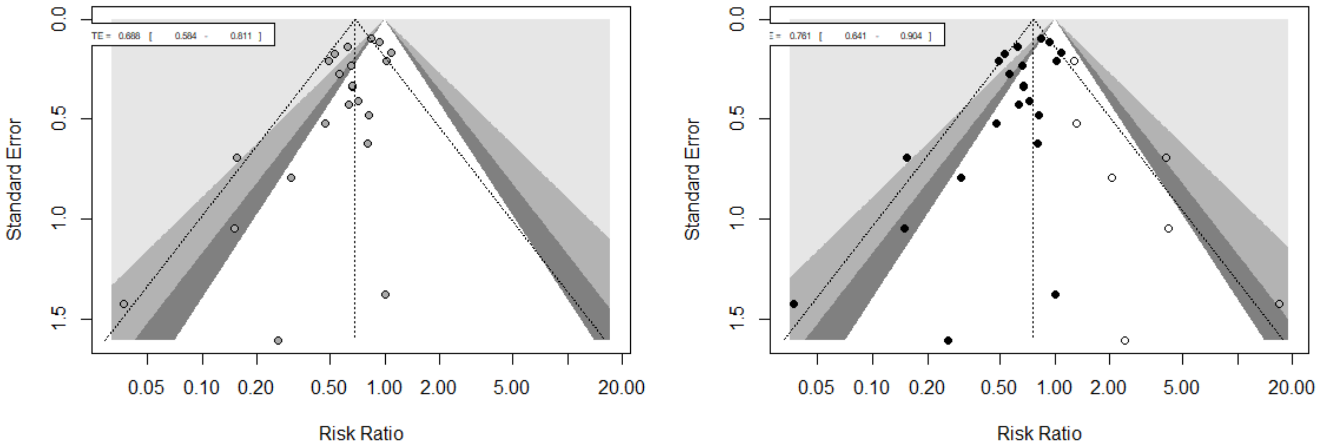
TE = 0.688 [0.584 - 0.811]
Standard Error
0.0
0.5
1.0
1.5
0.05 0.10 0.20 0.50 1.00 2.00 5.00 20.00
Risk Ratio
0.761 [0.641 - 0.904]
Standard Error
0.0
0.5
1.0
1.5
0.05 0.10 0.20 0.50 1.00 2.00 5.00 20.00
Risk Ratio

15 References

1. Alshryda S, Mason J, Hungin APS, et al. Topical (intra-articular) tranexamic acid reduces blood loss and transfusion rates following total hip replacement: A randomized controlled trial (TRANX-H). *Journal of Bone and Joint Surgery - Series A* 2013; **95**(21): 1969-74.
2. Clave A, Gerard R, Lacroix J, et al. A randomized, double-blind, placebo-controlled trial on the efficacy of tranexamic acid combined with rivaroxaban thromboprophylaxis in reducing blood loss after primary cementless total hip arthroplasty. *Bone and Joint Journal* 2019; (2): 207-12.
3. Cvetanovich G, Fillingham Y, O'Brien M, et al. Tranexamic acid reduces blood loss after primary shoulder arthroplasty: a double-blind, placebo-controlled, prospective, randomized controlled trial. *JSES open access*, 2018. http://cochranelibrary-wiley.com/o/cochrane/clcentral/articles/201/CN-01465201/frame.html https://ac.els-cdn.com/S2468602618300081/1-s2.0-S2468602618300081-main.pdf?_tid=68e045f0-7585-49a1-8b4a-eaac3adbeb45&acdnat=1535708790_e5a329293d00e72c7ea46d7d9a395fa6 (accessed.
4. Georgiadis A, Muh S, Silverton C, Weir R, Laker M. A prospective double-blind placebo controlled trial of topical tranexamic acid in total knee arthroplasty. *Journal of arthroplasty*, 2013. http://cochranelibrary-wiley.com/o/cochrane/clcentral/articles/497/CN-01261497/frame.html https://ac.els-cdn.com/S088354031300483X/1-s2.0-S088354031300483X-main.pdf?_tid=59354fa5-0311-4c70-9313-dfba78f11d98&acdnat=1535708682_61f1e74ff34906beb0830675cdb9cc33 (accessed.
5. Gillespie R, Joseph S, Streit JJ, Shishani Y, Gobezie R. Neer Award 2015: A randomized, prospective evaluation on the effectiveness of tranexamic acid in reducing blood loss after total shoulder arthroplasty. *Journal of Shoulder and Elbow Surgery* 2015; **24**(11): 1679-84.
6. Goobie SM, Zurakowski D, McCann ME, et al. Tranexamic acid is efficacious at decreasing the rate of blood loss in adolescent scoliosis surgery: A randomized placebo-controlled trial. *Journal of Bone and Joint Surgery - American Volume* 2018; **100**(23): 2024-32.
7. Johansson PI, Rasmussen AS, Thomsen LL. Intravenous iron isomaltoside 1000 (Monofer) reduces postoperative anaemia in preoperatively non-anaemic patients undergoing elective or subacute coronary artery bypass graft, valve replacement or a combination thereof: A randomized double-blind placebo-controlled clinical trial (the PROTECT trial). *Vox Sanguinis* 2015; **109**(3): 257-66.
8. Laine A, Niemi T, Schramko A. Transfusion threshold of hemoglobin 80 g/L Is comparable to 100 g/L in terms of bleeding in cardiac surgery: a prospective randomized study. *Journal of Cardiothoracic and Vascular Anesthesia* 2017.
9. Langille M, Chiarella A, Côté D, et al. Intravenous tranexamic acid and intraoperative visualization during functional endoscopic sinus surgery: a double-blind randomized controlled trial. *International forum of allergy & rhinology*, 2013. http://cochranelibrary-wiley.com/o/cochrane/clcentral/articles/740/CN-00861740/frame.html https://onlinelibrary.wiley.com/doi/pdf/10.1002/alr.21100 (accessed.
10. Mazer CD, Whitlock RP, Fergusson DA, et al. Restrictive or liberal red-cell transfusion for cardiac surgery. *The New England Journal of Medicine* 2017.
11. Murphy GJ, Allen SM, Unsworth-White J, Lewis CT, Dalrymple-Hay MJ. Safety and efficacy of perioperative cell salvage and autotransfusion after coronary artery bypass grafting: a randomized trial. *Ann Thorac Surg* 2004; **77**(5): 1553-9.
12. Onodera T, Majima T, Sawaguchi N, Kasahara Y, Ishigaki T, Minami A. Risk of deep venous thrombosis in drain clamping with tranexamic acid and carbazochrome sodium sulfonate hydrate in total knee arthroplasty. *J Arthroplasty* 2012; **27**(1): 105-8.
13. Palmieri TL, Holmes JHt, Arnoldo B, et al. Transfusion Requirement in Burn Care Evaluation (TRIBE): a multicenter randomized prospective trial of blood transfusion in major burn injury. *Annals of Surgery* 2017.
14. Perez-Jimeno N, Munoz M, Mateo J, Mayoral AP, Herrera A. Efficacy of topical tranexamic acid within a blood-saving programme for primary total hip arthroplasty: a pragmatic, open-label randomised study. *Blood Transfus* 2018; **16**(6): 490-7.
15. Spahn DR, Spahn GH, Stein P, et al. Effect of ultra-short-term treatment of patients with iron deficiency or anaemia undergoing cardiac surgery: a prospective randomised trial. *The Lancet* 2019.
16. Springer B, Odum S, Fehring T. What Is the Benefit of Tranexamic Acid vs Reinfusion Drains in Total Joint Arthroplasty? *Journal of arthroplasty*, 2016. http://cochranelibrary-wiley.com/o/cochrane/clcentral/articles/531/CN-01127531/frame.html https://ac.els-cdn.com/S0883540315007275/1-s2.0-S0883540315007275-main.pdf?_tid=a5805646-6da4-47ab-b486-9c577c32c413&acdnat=1535708858_0f7d2c78455f649d221d09d895abe26d (accessed.

17. Vara A, Koueiter D, Pinkas D, Gowda A, Wiater B, Wiater J. Intravenous tranexamic acid reduces total blood loss in reverse total shoulder arthroplasty: a prospective, double-blinded, randomized, controlled trial. *Journal of shoulder and elbow surgery*, 2017. http://cochranelibrary-wiley.com/o/cochrane/clcentral/articles/842/CN-01454842/frame.html https://ac.els-cdn.com/S1058274617300162/1-s2.0-S1058274617300162-main.pdf?_tid=491a6605-cb47-4e20-8534-62c6ec803096&acdnat=1535708629_ab9fa88324bf3ebbb9aa83d56d7c5f89 (accessed.
18. Verma K, Errico T, Diefenbach C, et al. The relative efficacy of antifibrinolytics in adolescent idiopathic scoliosis: a prospective randomized trial. *Journal of bone and joint surgery American volume*, 2014. http://cochranelibrary-wiley.com/o/cochrane/clcentral/articles/970/CN-00990970/frame.html (accessed.
19. Watts C, Houdek M, Sems S, Cross W, Pagnano M. Tranexamic Acid Safely Reduced Blood Loss in Hemi- and Total Hip Arthroplasty for Acute Femoral Neck Fracture: a Randomized Clinical Trial. *Journal of orthopaedic trauma*, 2017. http://cochranelibrary-wiley.com/o/cochrane/clcentral/articles/279/CN-01600279/frame.html (accessed.
20. Aguilera X, Martinez-Zapata M, Bosch A, et al. Efficacy and safety of fibrin glue and tranexamic acid to prevent postoperative blood loss in total knee arthroplasty: a randomized controlled clinical trial. *Journal of bone and joint surgery American volume*, 2013. (accessed.
21. Blauhut B, Harringer W, Bettelheim P, Doran JE, Spath P, Lundsgaard-Hansen P. Comparison of the effects of aprotinin and tranexamic acid on blood loss and related variables after cardiopulmonary bypass. *J Thorac Cardiovasc Surg* 1994; **108**(6): 1083-91.
22. Grover M, Talwalkar S, Casbard A, et al. Silent myocardial ischaemia and haemoglobin concentration: a randomized controlled trial of transfusion strategy in lower limb arthroplasty. *Vox Sang* 2006; **90**(2): 105-12.
23. Kuitunen A, Hiippala S, Vahtera E, Rasi V, Salmenpera M. The effects of aprotinin and tranexamic acid on thrombin generation and fibrinolytic response after cardiac surgery. *Acta Anaesthesiol Scand* 2005; **49**(9): 1272-9.
24. So-Osman C, Nelissen R, Brand R, et al. The impact of a restrictive transfusion trigger on post-operative complication rate and well-being following elective orthopaedic surgery: a post-hoc analysis of a randomised study. *Blood transfusion = Trasfusione del sangue* 2013; **11**(2): 289-95.
25. Carson JL, Terrin ML, Noveck H, et al. Liberal or restrictive transfusion in high-risk patients after hip surgery. *N Engl J Med* 2011; **365**(26): 2453-62.
26. Huang Z, Xie X, Li L, et al. Intravenous and topical tranexamic acid alone are superior to tourniquet use for primary total knee arthroplasty : A prospective, randomized controlled trial. *Journal of Bone and Joint Surgery - American Volume* 2017; **99**(24): 2053-61.
27. Lin PC, Hsu CH, Chen WS, Wang JW. Does tranexamic acid save blood in minimally invasive total knee arthroplasty? *Clinical Orthopaedics and Related Research* 2011; **469**(7): 1995-2002.
28. Myles P, Smith J, Forbes A, et al. Tranexamic acid in patients undergoing coronary-artery surgery. *New england journal of medicine*, 2017. http://cochranelibrary-wiley.com/o/cochrane/clcentral/articles/536/CN-01297536/frame.html https://www.nejm.org/doi/pdf/10.1056/NEJMoa1606424 (accessed.
29. Yi Z, Bin S, Jing Y, Zongke Z, Pengde K, Fuxing P. Tranexamic acid administration in primary total hip arthroplasty a randomized controlled trial of intravenous combined with topical versus single-dose intravenous administration. *Journal of Bone and Joint Surgery - American Volume* 2016; **98**(12): 983-91.
30. Zonis Z, Seear M, Reichert C, Sett S, Allen C. The effect of preoperative tranexamic acid on blood loss after cardiac operations in children. *J Thorac Cardiovasc Surg* 1996; **111**(5): 982-7.
31. Laoruengthana A, Rattanaprichavej P, Rasamimongkol S, Galassi M, Weerakul S, Pongpirul K. Intra-Articular Tranexamic Acid Mitigates Blood Loss and Morphine Use After Total Knee Arthroplasty. A Randomized Controlled Trial. *Journal of arthroplasty* 2019.
32. Aghdaii N, Yazdanian F, Kabiri M, Ghaffarinejad MH. Effect of retransfusion of heparin remaining in the salvaged blood on postoperative blood loss in coronary artery bypass grafting: Comparison with homologous blood transfusion (running title: Postoperative blood loss in CABG). *Iranian Heart Journal* 2012; **13**(2): 24-34.
33. Ahn S, Shim J, Youn Y, et al. Effect of tranexamic acid on transfusion requirement in dual antiplatelet-treated anemic patients undergoing off-pump coronary artery bypass graft surgery-a randomized controlled study. *Circulation journal*, 2012. (accessed.
34. Albirmawy O, Saafan M, Shehata E, Basuni A, Eldaba A. Topical application of tranexamic acid after adenoidectomy: a double-blind, prospective, randomized, controlled study. *International journal of pediatric otorhinolaryngology*, 2013. (accessed.

35. Ali Shah MU, Asghar MI, Siddiqi R, Chaudhri MS, Janjua AM, Iqbal A. Topical application of tranexamic acid reduces postoperative bleeding in open-heart surgery: myth or fact? *Journal of the College of Physicians and Surgeons--Pakistan : JCPSP* 2015; **25**(3): 161-5.
36. Alipour M, Tabari M, Keramati M, Zarmehri A, Makhmalbaf H. Effectiveness of oral Tranexamic acid administration on blood loss after knee artroplasty: a randomized clinical trial. *Transfusion and apheresis science*, 2013. (accessed.
37. Altun G, Hemsinli D, Pulathan Z, Civelek A. Emergency coronary bypass surgery in patients under the influence of dual antiplatelet therapy: effects of tranexamic acid and desmopressin acetate. *Turkish journal of medical sciences*, 2017. (accessed.
38. Alvarez JC, Santiveri FX, Ramos I, Vela E, Puig L, Escolano F. Tranexamic acid reduces blood transfusion in total knee arthroplasty even when a blood conservation program is applied. *Transfusion* 2008; **48**(3): 519-25.
39. Andreasen JJ, Nielsen C. Prophylactic tranexamic acid in elective, primary coronary artery bypass surgery using cardiopulmonary bypass. *Eur J Cardiothorac Surg* 2004; **26**(2): 311-7.
40. Antinolfi P, Innocenti B, Caraffa A, Peretti G, Cerulli G. Post-operative blood loss in total knee arthroplasty: knee flexion versus pharmacological techniques. *Knee surgery, sports traumatology, arthroscopy*, 2014. (accessed.
41. Armellin G, Casella S, Guzzinati S, Pasini L, Marcassa A, Giron G. Tranexamic acid in aortic valve replacement. *J Cardiothorac Vasc Anesth* 2001; **15**(3): 331-5.
42. Auvinen O, Baer GA, Nordback I, Saaristo J. Antifibrinolytic therapy for prevention of hemorrhage during surgery of the thyroid gland. *Klin Wochenschr* 1987; **65**(6): 253-5.
43. Avidan MS, Alcock EL, Da Fonseca J, et al. Comparison of structured use of routine laboratory tests or near-patient assessment with clinical judgement in the management of bleeding after cardiac surgery. *Br J Anaesth* 2004; **92**(2): 178-86.
44. Basavaraj K, Hegde R. A randomized prospective study of efficacy of tranexamicacid on perioperative blood loss in thoracicspine fixation. *Sri lankan journal of anaesthesiology*, 2017. (accessed.
45. Beikaei M, Ghazipour A, Derakhshande V, Saki N, Nikakhlagh S. Evaluating the effect of intravenous tranexamic acid on intraoperative bleeding during elective rhinoplasty surgery. *Biomedical and pharmacology journal*, 2015. (accessed.
46. Benoni G, Fredin H, Knebel R, Nilsson P. Blood conservation with tranexamic acid in total hip arthroplasty: a randomized, double-blind study in 40 primary operations. *Acta Orthop Scand* 2001; **72**(5): 442-8.
47. Blatsoukas KS, Drosos GI, Kazakos K, et al. Prospective comparative study of two different autotransfusion methods versus control group in total knee replacement. *Archives of Orthopaedic & Trauma Surgery* 2010; **130**(6): 733-7.
48. Boylan JF, Klinck JR, Sandler AN, et al. Tranexamic acid reduces blood loss, transfusion requirements, and coagulation factor use in primary orthotopic liver transplantation. *Anesthesiology* 1996; **85**(5): 1043-8; discussion 30A-31A.
49. Bracey AW, Radovancevic R, Riggs SA, et al. Lowering the hemoglobin threshold for transfusion in coronary artery bypass procedures: effect on patient outcome. *Transfusion* 1999; **39**(10): 1070-7.
50. Bradshaw AR, Monoghan J, Campbell D. Oral tranexamic acid reduces blood loss in total knee replacement arthroplasty. *Current Orthopaedic Practice* 2012; **23**(3): 209-12.
51. Brown RS, Thwaites BK, Mongan PD. Tranexamic acid is effective in decreasing postoperative bleeding and transfusions in primary coronary artery bypass operations: a double-blind, randomized, placebo-controlled trial. *Anesth Analg* 1997; **85**(5): 963-70.
52. Bulutcu FS, Ozbek U, Polat B, Yalcin Y, Karaci AR, Bayindir O. Which may be effective to reduce blood loss after cardiac operations in cyanotic children: tranexamic acid, aprotinin or a combination? *Paediatr Anaesth* 2005; **15**(1): 41-6.
53. Bush RL, Pevec WC, Holcroft JW. A prospective, randomized trial limiting perioperative red blood cell transfusions in vascular patients. *Am J Surg* 1997; **174**(2): 143-8.
54. Cao WJ, Zhu SL, Liu XD, et al. Tranexamic acid reduces blood loss in total knee arthroplasty: Effectiveness and safety. *Chinese Journal of Tissue Engineering Research* 2015; **19**(31): 4944-8.

55. Carabini L, Moreland N, Vealey R, et al. A Randomized Controlled Trial of Low-Dose Tranexamic Acid versus Placebo to Reduce Red Blood Cell Transfusion During Complex Multilevel Spine Fusion Surgery. *World neurosurgery*, 2017. http://cochranelibrary-wiley.com/o/cochrane/clcentral/articles/849/CN-01452849/frame.html https://ac.els-cdn.com/S1878875017319939/1-s2.0-S1878875017319939-main.pdf?_tid=71586c32-ddce-43c2-87ef-6456818f9d1d&acdnat=1535708690_482e586b3ac442897050ce4f6f2091bf (accessed.
56. Carson JL, Terrin ML, Barton FB, et al. A pilot randomized trial comparing symptomatic vs. hemoglobin-level- driven red blood cell transfusions following hip fracture. *Transfusion* 1998; **38**(6): 522-9.
57. Casati V, Bellotti F, Gerli C, et al. Tranexamic acid administration after cardiac surgery: a prospective, randomized, double-blind, placebo-controlled study. *Anesthesiology* 2001; **94**(1): 8-14.
58. Casati V, Sandrelli L, Speziali G, Calori G, Grasso MA, Spagnolo S. Hemostatic effects of tranexamic acid in elective thoracic aortic surgery: a prospective, randomized, double-blind, placebo-controlled study. *J Thorac Cardiovasc Surg* 2002; **123**(6): 1084-91.
59. Casati V, Della Valle P, Benussi S, et al. Effects of tranexamic acid on postoperative bleeding and related hematochemical variables in coronary surgery: Comparison between on-pump and off-pump techniques. *J Thorac Cardiovasc Surg* 2004; **128**(1): 83-91.
60. Chakravarthy M, Muniraj G, Patil S, Suryaprakash S, Mitra S, Shivalingappa B. A randomized prospective analysis of alteration of hemostatic function in patients receiving tranexamic acid and hydroxyethyl starch (130/0.4) undergoing off pump coronary artery bypass surgery. *Annals of cardiac anaesthesia*, 2012. http://cochranelibrary-wiley.com/o/cochrane/clcentral/articles/564/CN-00880564/frame.html http://www.annals.in/article.asp?issn=0971-9784;year=2012;volume=15;issue=2;spage=105;epage=110;aulast=Chakravarthy (accessed.
61. Chauhan S, Bisoi A, Modi R, Gharde P, Rajesh MR. Tranexamic acid in paediatric cardiac surgery. *Indian J Med Res* 2003; **118**: 86-9.
62. Chauhan S, Bisoi A, Kumar N, et al. Dose comparison of tranexamic acid in pediatric cardiac surgery. *Asian Cardiovasc Thorac Ann* 2004; **12**(2): 121-4.
63. Chen TT, Jiandong L, Wang G, Jiang SL, Li LB, Gao CQ. Combined treatment of ulinastatin and tranexamic acid provides beneficial effects by inhibiting inflammatory and fibrinolytic response in patients undergoing heart valve replacement surgery. *Heart Surg Forum* 2013; **16**(1): E38-47.
64. Choudhuri P, Biswas B. Intraoperative Use of Epsilon Amino Caproic Acid and Tranexamic Acid in Surgeries Performed Under Cardiopulmonary Bypass: a Comparative Study To Assess Their Impact On Reopening Due To Postoperative Bleeding. *Ethiopian journal of health sciences*, 2015. http://cochranelibrary-wiley.com/o/cochrane/clcentral/articles/788/CN-01179788/frame.html https://www.ncbi.nlm.nih.gov/pmc/articles/PMC4650883/pdf/EJHS2503-0273.pdf (accessed.
65. Christabel A, Muthusekhar M, Narayanan V, et al. Effectiveness of tranexamic acid on intraoperative blood loss in isolated Le Fort I osteotomies--a prospective, triple blinded randomized clinical trial. *Journal of cranio-maxillo-facial surgery*, 2014. http://cochranelibrary-wiley.com/o/cochrane/clcentral/articles/551/CN-01022551/frame.html https://ac.els-cdn.com/S1010518214000833/1-s2.0-S1010518214000833-main.pdf?_tid=fbbad2f1-b5d9-43de-aa95-4f9102858438&acdnat=1535708522_73d10d9da2da5004e14685869ac93b02 (accessed.
66. Claeys MA, Vermeersch N, Haentjens P. Reduction of blood loss with tranexamic acid in primary total hip replacement surgery. *Acta Chir Belg* 2007; **107**(4): 397-401.
67. Clagett GP, Valentine RJ, Jackson MR, Mathison C, Kakish HB, Bengtson TD. A randomized trial of intraoperative autotransfusion during aortic surgery. *Journal of Vascular Surgery* 1999; **29**(1): 22-30.
68. Coffey A, Pittmam J, Halbrook H, Fehrenbacher J, Beckman D, Hormuth D. The use of tranexamic acid to reduce postoperative bleeding following cardiac surgery: a double-blind randomized trial. *Am Surg* 1995; **61**(7): 566-8.
69. Corbeau JJ, Monrigal JP, Jacob JP, et al. Comparative effects of aprotinin and tranexamic acid on blood loss in cardiac surgery. *Annales Françaises d'Anesthésie et de Réanimation* 1995; **14**(2): 154-61.
70. Cui Y, Hei F, Long C, et al. Perioperative monitoring of thromboelastograph on blood protection and recovery for severely cyanotic patients undergoing complex cardiac surgery. *Artif Organs* 2010; **34**(11): 955-60.

71. Dadure C, Sauter M, Bringuier S, et al. Intraoperative Tranexamic Acid Reduces Blood Transfusion in Children Undergoing Craniosynostosis Surgery: A Randomized Double-blind Study. *Anesthesiology* 2011; **114**(4): 856-61.
72. Dalmau A, Sabate A, Acosta F, et al. Tranexamic acid reduces red cell transfusion better than epsilon-aminocaproic acid or placebo in liver transplantation. *Anesth Analg* 2000; **91**(1): 29-34.
73. Dalrymple-Hay MJ, Pack L, Deakin CD, et al. Autotransfusion of washed shed mediastinal fluid decreases the requirement for autologous blood transfusion following cardiac surgery: a prospective randomized trial. *European Journal of Cardio-Thoracic Surgery* 1999; **15**(6): 830-4.
74. Damgaard S, Steinbruchel DA, Andersen LW, Tvede M, Nielsen CH, Bendtzen K. Cell Saver for On-pump Coronary Operations Reduces Systemic Inflammatory Markers: A Randomized Trial. *Annals of Thoracic Surgery* 2010; **89**(5): 1511-7.
75. Dell'Amore A, Caroli G, Nizar A, et al. Can topical application of tranexamic acid reduce blood loss in thoracic surgery? A prospective randomised double blind investigation. *Heart, lung & circulation*, 2012. http://cochranelibrary-wiley.com/o/cochrane/clcentral/articles/432/CN-00879432/frame.html https://ac.els-cdn.com/S1443950612011110/1-s2.0-S1443950612011110-main.pdf?_tid=138aaabf-71be-4d8b-8e4e-0a242f34628b&acdnat=1535708391_f59917e90cb212d50cc6f204e138b448 (accessed.
76. Dietrich W, Barankay A, Dilthey G, Mitto HP, Richter JA. Reduction of blood utilization during myocardial revascularization. *Journal of Thoracic & Cardiovascular Surgery* 1989; **97**(2): 213-9.
77. Diprose P, Herbertson MJ, O'Shaughnessy D, Deakin CD, Gill RS. Reducing allogeneic transfusion in cardiac surgery: a randomized double-blind placebo-controlled trial of antifibrinolytic therapies used in addition to intra-operative cell salvage. *Br J Anaesth* 2005; **94**(3): 271-8.
78. Eftekharian H, Vahedi R, Karagah T, Tabrizi R. Effect of tranexamic acid irrigation on perioperative blood loss during orthognathic surgery: a double-blind, randomized controlled clinical trial. *Journal of oral and maxillofacial surgery*, 2014. http://cochranelibrary-wiley.com/o/cochrane/clcentral/articles/915/CN-01036915/frame.html (accessed.
79. Ekback G, Axelsson K, Ryttberg L, et al. Tranexamic acid reduces blood loss in total hip replacement surgery. *Anesth Analg* 2000; **91**(5): 1124-30.
80. El Shal SM, Hasanein R. Effect of intravenous tranexamic acid and epsilon aminocaproic acid on bleeding and surgical field quality during functional endoscopic sinus surgery (FESS). *Egyptian Journal of Anaesthesia* 2015; **31**(1): 1-7.
81. Elawad A, Ohlin AK, Berntorp E, Nilsson IM, Fredin H. Intraoperative autotransfusion in primary hip arthroplasty - A randomized comparison with homologous blood. *Acta Orthopaedica Scandinavica, Supplement* 1991; **62**(246): 3.
82. Engel JM, Hohaus T, Ruwoldt R, Menges T, Jurgensen I, Hempelmann G. Regional hemostatic status and blood requirements after total knee arthroplasty with and without tranexamic acid or aprotinin. *Anesth Analg* 2001; **92**(3): 775-80.
83. Felli L, Revello S, Gatto P, et al. Single Intravenous Administration of Tranexamic Acid in Anterior Cruciate Ligament Reconstruction to Reduce Postoperative Hemarthrosis and Increase Functional Outcomes in the Early Phase of Postoperative Rehabilitation: A Randomized Controlled Trial. *Arthroscopy - Journal of Arthroscopic and Related Surgery* 2019; **35**(1): 149-57.
84. Garneti N, Field J. Bone bleeding during total hip arthroplasty after administration of tranexamic acid. *J Arthroplasty* 2004; **19**(4): 488-92.
85. Ghaffari Nejad MH, Baharestani B, Esfandiari R, Hashemi J, Panahipoor A. Evaluation and Comparison of Using Low-Dose Aprotinin and Tranexamic Acid in CABG: a Double Blind Randomized Clinical Trial. *Journal of Tehran University Heart Center* 2012; **7**(1): 15-8.
86. Gill JB, Chase E, Rosenstein AD. The use of tranexamic acid in revision total hip arthroplasty: a pilot study. *Current Orthopaedic Practice* 2009; **20**(2): 152-6.
87. Good L, Peterson E, Lisander B. Tranexamic acid decreases external blood loss but not hidden blood loss in total knee replacement. *Br J Anaesth* 2003; **90**(5): 596-9.
88. Gregersen M, Borris LC, Damsgaard EM. Postoperative blood transfusion strategy in frail, anemic elderly patients with hip fracture: the TRIFE randomized controlled trial. *Acta Orthop* 2015; **86**(3): 363-72.
89. Greiff G, Stenseth R, Bjella L, et al. Tranexamic acid reduces blood transfusions in elderly patients undergoing combined aortic valve and coronary artery bypass graft surgery: A randomized controlled trial. *Journal of Cardiothoracic and Vascular Anesthesia* 2012; **26**(2): 232-8.
90. Hajjar LA, Vincent JL, Galas FR, et al. Transfusion requirements after cardiac surgery: the TRACS randomized controlled trial. *Jama* 2010; **304**(14): 1559-67.

91. Hardy J-FMD, Bélisle SMD, Dupont CM, et al. Prophylactic Tranexamic Acid and ϵ-Aminocaproic Acid for Primary Myocardial Revascularization. *The Annals of Thoracic Surgery* 1998; **65**(2): 371-6.

92. Hiippala S, Strid L, Wennerstrand M, et al. Tranexamic acid (Cyklokapron) reduces perioperative blood loss associated with total knee arthroplasty. *Br J Anaesth* 1995; **74**(5): 534-7.

93. Hiippala ST, Strid LJ, Wennerstrand MI, et al. Tranexamic acid radically decreases blood loss and transfusions associated with total knee arthroplasty. *Anesth Analg* 1997; **84**(4): 839-44.

94. Horrow JC, Hlavacek J, Strong MD, et al. Prophylactic tranexamic acid decreases bleeding after cardiac operations. *J Thorac Cardiovasc Surg* 1990; **99**(1): 70-4.

95. Horrow JC, Van Riper DF, Strong MD, Brodsky I, Parmet JL. Hemostatic effects of tranexamic acid and desmopressin during cardiac surgery. *Circulation* 1991; **84**(5): 2063-70.

96. Horrow JC, Van Riper DF, Strong MD, Grunewald KE, Parmet JL. The dose-response relationship of tranexamic acid. *Anesthesiology* 1995; **82**(2): 383-92.

97. Horstmann WG, Swierstra MJ, Ohanis D, Rolink R, Kollen BJ, Verheyen CC. Favourable results of a new intraoperative and postoperative filtered autologous blood re-transfusion system in total hip arthroplasty: a randomised controlled trial. *International Orthopaedics* 2014; **38**(1): 13-8.

98. Hou ZY, Su CZ, Pang T, et al. Primary unilateral cemented total knee arthroplasty: Effect of tranexamic acid usage on blood loss. *Chinese Journal of Tissue Engineering Research* 2015; **19**(9): 1329-34.

99. Hu W-H. Efficacy of intravenous versus topical administration of tranexanmic acid in primary total knee arthroplasty. *Chinese journal of tissue engineering research*, 2018. http://cochranelibrary-wiley.com/o/cochrane/clcentral/articles/953/CN-01570953/frame.html (accessed.

100. Huang Z, Zhang W, Li W, Bai G, Zhang C, Lin J. A prospective randomized self-controlled study on effect of tranexamic acid in reducing blood loss in total knee arthroplasty. *Zhongguo xiu fu chong jian wai ke za zhi [Chinese journal of reparative and reconstructive surgery]*, 2015. http://cochranelibrary-wiley.com/o/cochrane/clcentral/articles/474/CN-01169474/frame.html (accessed.

101. Imai N, Dohmae Y, Suda K, Miyasaka D, Ito T, Endo N. Tranexamic Acid for Reduction of Blood Loss During Total Hip Arthroplasty. *Journal of arthroplasty*, 2012. http://cochranelibrary-wiley.com/o/cochrane/clcentral/articles/003/CN-00900003/frame.htmlhttps://www.sciencedirect.com/science/article/pii/S088354031200294X?via%3Dihub https://ac.els-cdn.com/S088354031200294X/1-s2.0-S088354031200294X-main.pdf?_tid=a4d8fb5f-35b5-4957-95a7-3081978ec92b&acdnat=1535708771_9e006b32bc467c3207b93b30566e5a6f (accessed.

102. Ishida K, Tsumura N, Kitagawa A, et al. Intra-articular injection of tranexamic acid reduces not only blood loss but also knee joint swelling after total knee arthroplasty. *International Orthopaedics* 2011; **35**(11): 1639-45.

103. Jansen AJ, Andreica S, Claeys M, D'Haese J, Camu F, Jochmans K. Use of tranexamic acid for an effective blood conservation strategy after total knee arthroplasty. *Br J Anaesth* 1999; **83**(4): 596-601.

104. Jares M, Vanek T, Straka Z, Brucek P. Tranexamic acid reduces bleeding after off-pump coronary artery bypass grafting. *J Cardiovasc Surg (Torino)* 2003; **44**(2): 205-8.

105. Jaszczyk M, Kozerawski D, Kolodziej L, Kazimierczak A, Sarnecki P, Sieczka L. Effect of Single Preoperative Dose of Tranexamic Acid on Blood Loss and Transfusion in Hip Arthroplasty. *Ortopedia, traumatologia, rehabilitacja*, 2015. http://cochranelibrary-wiley.com/o/cochrane/clcentral/articles/991/CN-01341991/frame.html (accessed.

106. Kakar PN, Gupta N, Govil P, Shah V. Efficacy and safety of tranexamic acid in control of bleeding following tkr: a randomized clinical trial. *Indian journal of anaesthesia* 2009; **53**(6): 667-71.

107. Karimi A, Hasheminasab M, Mohammadi SS. Efficacy of tranexamic acid on blood loss during bimaxilary osteotomy: A randomized double blind clinical trial. *Saudi Journal of Anaesthesia* 2012; **6**(1): 41-5.

108. Karski J, Djaiani G, Carroll J, et al. Tranexamic acid and early saphenous vein graft patency in conventional coronary artery bypass graft surgery: a prospective randomized controlled clinical trial. *J Thorac Cardiovasc Surg* 2005; **130**(2): 309-14.

109. Karski JM, Teasdale SJ, Norman P, et al. Prevention of bleeding after cardiopulmonary bypass with high-dose tranexamic acid. Double-blind, randomized clinical trial. *J Thorac Cardiovasc Surg* 1995; **110**(3): 835-42.

110. Kaspar M, Ramsay MA, Nguyen AT, Cogswell M, Hurst G, Ramsay KJ. Continuous small-dose tranexamic acid reduces fibrinolysis but not transfusion requirements during orthotopic liver transplantation. *Anesth Analg* 1997; **85**(2): 281-5.

111. Katoh J, Tsuchiya K, Sato W, Nakajima M, Iida Y. Additional postbypass administration of tranexamic acid reduces blood loss after cardiac operations. *J Thorac Cardiovasc Surg* 1997; **113**(4): 802-4.

112. Katsaros D, Petricevic M, Snow NJ, Woodhall DD, Van Bergen R. Tranexamic acid reduces postbypass blood use: a double-blinded, prospective, randomized study of 210 patients. *Ann Thorac Surg* 1996; **61**(4): 1131-5.

113. Keyhani S, Esmailiejah A, Abbasian M, Safdari F. Which route of tranexamic acid administration is more effective to reduce blood loss following total knee arthroplasty? *Archives of bone and joint surgery*, 2016. http://cochranelibrary-wiley.com/o/cochrane/clcentral/articles/104/CN-01208104/frame.html https://www.ncbi.nlm.nih.gov/pmc/articles/PMC4733239/pdf/ABJS-4-65.pdf (accessed.

114. Kim T, Chang C, Kang Y, et al. Clinical value of tranexamic acid in unilateral and simultaneous bilateral TKAs under a contemporary blood-saving protocol: a randomized controlled trial. *Knee surgery, sports traumatology, arthroscopy*, 2014. http://cochranelibrary-wiley.com/o/cochrane/clcentral/articles/734/CN-00861734/frame.html https://link.springer.com/content/pdf/10.1007%2Fs00167-013-2492-1.pdf (accessed.

115. Klein AA, Nashef SA, Sharples L, et al. A randomized controlled trial of cell salvage in routine cardiac surgery. *Anesthesia & Analgesia* 2008; **107**(5): 1487-95.

116. Koch CG, Sessler DI, Mascha EJ, et al. A randomized clinical trial of red blood cell transfusion triggers in cardiac surgery. *The Annals of Thoracic Surgery* 2017.

117. Kojima T, Gando S, Morimoto Y, et al. Systematic elucidation of effects of tranexamic acid on fibrinolysis and bleeding during and after cardiopulmonary bypass surgery. *Thromb Res* 2001; **104**(5): 301-7.

118. Kuitunen AH, Suojaranta-Ylinen RT, Kukkonen SI, Niemi TT. Tranexamic acid does not correct the haemostatic impairment caused by hydroxyethyl starch (200 kDa/0.5) after cardiac surgery. *Blood Coagul Fibrinolysis* 2006; **17**(8): 639-45.

119. Kumar S, Randhawa MS, Ganesamoni R, Singh SK. Tranexamic acid reduces blood loss in percutaneous nephrolithotomy: A prospective randomized controlled study. *Journal of Endourology* 2013; **27**(2): 124-5.

120. Later AF, Maas JJ, Engbers FH, et al. Tranexamic acid and aprotinin in low- and intermediate-risk cardiac surgery: a non-sponsored, double-blind, randomised, placebo-controlled trial. *Eur J Cardiothorac Surg* 2009; **36**(2): 322-9.

121. Laub GW, Dharan M, Riebman JB, et al. The impact of intraoperative autotransfusion on cardiac surgery. A prospective randomized double-blind study. *Chest* 1993; **104**(3): 686-9.

122. Lee S, Cho K, Khurana S, Kim K. Less blood loss under concomitant administration of tranexamic acid and indirect factor Xa inhibitor following total knee arthroplasty: a prospective randomized controlled trial. *Knee surgery, sports traumatology, arthroscopy*, 2013. http://cochranelibrary-wiley.com/o/cochrane/clcentral/articles/310/CN-00995310/frame.html https://link.springer.com/content/pdf/10.1007%2Fs00167-012-2213-1.pdf (accessed.

123. Lee Y, Park S, Kim J, Cho C. Effect of tranexamic acid on reducing postoperative blood loss in combined hypotensive epidural anesthesia and general anesthesia for total hip replacement. *Journal of clinical anesthesia*, 2013. http://cochranelibrary-wiley.com/o/cochrane/clcentral/articles/074/CN-00914074/frame.html https://ac.els-cdn.com/S0952818013001785/1-s2.0-S0952818013001785-main.pdf?_tid=8d1380cd-ef08-4783-9787-c90f35a7f435&acdnat=1535708497_4733ace813462b3b05d9fdc06e268958 (accessed.

124. Lemay E, Guay J, Cote C, Roy A. Tranexamic acid reduces the need for allogenic red blood cell transfusions in patients undergoing total hip replacement. *Can J Anaesth* 2004; **51**(1): 31-7.

125. Li X, Xu X, Liu W, Wang Z. Longtime soaking of high concentration tranexamic acid in total hip arthroplasty: A prospective randomized controlled trial in 224 patients. *Pakistan Journal of Medical Sciences* 2015; **31**(6): 1306-11.

126. Liang J, Liu H, Huang X, et al. Using tranexamic acid soaked absorbable gelatin sponge following complex posterior lumbar spine surgery: a randomized control trial. *Clinical neurology and neurosurgery*, 2016. http://cochranelibrary-wiley.com/o/cochrane/clcentral/articles/420/CN-01178420/frame.html https://ac.els-cdn.com/S0303846716302141/1-s2.0-S0303846716302141-main.pdf?_tid=017916cd-cc44-412d-b1ee-6f20a62751cf&acdnat=1535708847_c43e89ccf907584fb73748bccbe35ddf (accessed.
127. Lin S, Chen C, Fu Y, Huang P, Chang J, Huang H. The efficacy of combined use of intraarticular and intravenous tranexamic acid on reducing blood loss and transfusion rate in total knee arthroplasty. *Journal of arthroplasty*, 2015. http://cochranelibrary-wiley.com/o/cochrane/clcentral/articles/026/CN-01255026/frame.html https://ac.els-cdn.com/S0883540314009140/1-s2.0-S0883540314009140-main.pdf?_tid=8e64fa74-01d2-403c-966b-ecf8611fda04&acdnat=1535708572_e05731c7a5e44e03b859613b12008c7d (accessed.
128. Lotke PA, Barth P, Garino JP, Cook EF. Predonated autologous blood transfusions after total knee arthroplasty: immediate versus delayed administration. *Journal of Arthroplasty* 1999; **14**(6): 647-50.
129. MacGillivray RG, Tarabichi SB, Hawari MF, Raoof NT. Tranexamic acid to reduce blood loss after bilateral total knee arthroplasty: a prospective, randomized double blind study. *Journal of Arthroplasty* 2011; **26**(1): 24-8.
130. Maddali MM, Rajakumar MC. Tranexamic acid and primary coronary artery bypass surgery: a prospective study. *Asian Cardiovasc Thorac Ann* 2007; **15**(4): 313-9.
131. Malhotra R, Kumar V, Garg B. The use of tranexamic acid to reduce blood loss in primary cementless total hip arthroplasty. *European Journal of Orthopaedic Surgery and Traumatology* 2011; **21**(2): 101-4.
132. Marberg H, Jeppsson A, Brandrup-Wognsen G. Postoperative autotransfusion of mediastinal shed blood does not influence haemostasis after elective coronary artery bypass grafting. *European Journal of Cardio-thoracic Surgery* 2010; **38**(6): 767-72.
133. Markatou M TK, Rizos R and Fassoulaki A. Targeting Perioperative Hemoglobin in Major Abdominal Surgery. *Journal of Anesthesia & Clinical Research* 2012; **3** (2).
134. McGill N, O'Shaughnessy D, Pickering R, Herbertson M, Gill R. Mechanical methods of reducing blood transfusion in cardiac surgery: Randomised controlled trial. *British Medical Journal* 2002; **324**(7349): 1299-302.
135. Mehr-Aein A, Davoodi S, Madani-Civi M. Effects of tranexamic acid and autotransfusion in coronary artery bypass. *Asian Cardiovasc Thorac Ann* 2007; **15**(1): 49-53.
136. Menges T, Rupp D, van Lessen A, Hempelmann G. [Measures for reducing the use of homologous blood. Effects on blood coagulation during total endoprosthesis]. *Anaesthesist* 1992; **41**(1): 27-33.
137. Menichetti A, Tritapepe L, Ruvolo G, et al. Changes in coagulation patterns, blood loss and blood use after cardiopulmonary bypass: aprotinin vs tranexamic acid vs epsilon aminocaproic acid. *J Cardiovasc Surg (Torino)* 1996; **37**(4): 401-7.
138. Mercer KG, Spark JI, Berridge DC, Kent PJ, Scott DJ. Randomized clinical trial of intraoperative autotransfusion in surgery for abdominal aortic aneurysm. *British Journal of Surgery* 2004; **91**(11): 1443-8.
139. Miller RA, May MW, Hendry WF, Whitfield HN, Wickham JE. The prevention of secondary haemorrhage after prostatectomy: the value of antifibrinolytic therapy. *Br J Urol* 1980; **52**(1): 26-8.
140. Mohib Y, Rashid RH, Ali M, Zubairi AJ, Umer M. Does tranexamic acid reduce blood transfusion following surgery for inter-trochanteric fracture? A randomized control trial. *JPMA The Journal of the Pakistan Medical Association* 2015; **65**(11).
141. Mu X, Wei J, Wang C, et al. Intravenous Administration of Tranexamic Acid Significantly Reduces Visible and Hidden Blood Loss Compared with Its Topical Administration for Double-Segment Posterior Lumbar Interbody Fusion: A Single-Center, Placebo-Controlled, Randomized Trial. *World neurosurgery* 2019; **122**: e821.
142. Murphy GJ, Rogers CS, Alwair H, et al. Safety, efficacy, and cost of intraoperative cell salvage and autotransfusion after off-pump coronary artery bypass surgery: A randomized trial. *Journal of Thoracic and Cardiovascular Surgery* 2005; **130**(1): 20-8.
143. Murphy GJ, Mango E, Lucchetti V, et al. A randomized trial of tranexamic acid in combination with cell salvage plus a meta-analysis of randomized trials evaluating tranexamic acid in off-pump coronary artery bypass grafting. *J Thorac Cardiovasc Surg* 2006; **132**(3): 475-80, 80 e1-8.

144. Nagabhushan RM, Shetty AP, Dumpa SR, Subramanian B, Kanna RM, Shanmuganathan R. Effectiveness and Safety of Batroxobin, Tranexamic Acid and a Combination in Reduction of Blood Loss in Lumbar Spinal Fusion Surgery. *Spine* 2018; **43**(5): E267-E73.
145. Neilipovitz DT, Murto K, Hall L, Barrowman NJ, Splinter WM. A randomized trial of tranexamic acid to reduce blood transfusion for scoliosis surgery. *Anesth Analg* 2001; **93**(1): 82-7.
146. Niskanen RO, Korkala OL. Tranexamic acid reduces blood loss in cemented hip arthroplasty: a randomized, double-blind study of 39 patients with osteoarthritis. *Acta Orthop* 2005; **76**(6): 829-32.
147. Nouraei M, Ghafari R, Gholipour Baradari A, Habibi MR, Sharifi N, Emami Zeydi A. Decreasing blood loss and the need for transfusion after CABG surgery: A double-blind randomized clinical trial of topical tranexamic acid. *Turkish Journal of Medical Sciences* 2013; **43**(2): 273-8.
148. Nuttall GA, Oliver WC, Ereth MH, et al. Comparison of blood-conservation strategies in cardiac surgery patients at high risk for bleeding. *Anesthesiology* 2000; **92**(3): 674-82.
149. Nuttall GA, Oliver WC, Santrach PJ, et al. Efficacy of a simple intraoperative transfusion algorithm for nonerythrocyte component utilization after cardiopulmonary bypass. *Anesthesiology* 2001; **94**(5): 773-81; discussion 5A-6A.
150. Oertli D, Laffer U, Haberthuer F, Kreuter U, Harder F. Perioperative and postoperative tranexamic acid reduces the local wound complication rate after surgery for breast cancer. *Br J Surg* 1994; **81**(6): 856-9.
151. Orpen NM, Little C, Walker G, Crawfurd EJ. Tranexamic acid reduces early post-operative blood loss after total knee arthroplasty: a prospective randomised controlled trial of 29 patients. *Knee* 2006; **13**(2): 106-10.
152. Painter TW, Daly DJ, Kluger R, et al. Intravenous tranexamic acid and lower limb arthroplasty-a randomised controlled feasibility study. *Anaesthesia and intensive care* 2018; **46**(4): 386-95.
153. Parrot D, Lancon JP, Merle JP, et al. Blood salvage in cardiac surgery. *Journal of Cardiothoracic & Vascular Anesthesia* 1991; **5**(5): 454-6.
154. Pauzenberger L, Domej M, Heuberer P, et al. The effect of intravenous tranexamic acid on blood loss and early post-operative pain in total shoulder arthroplasty. *The bone & joint journal*, 2017. http://cochranelibrary-wiley.com/o/cochrane/clcentral/articles/816/CN-01403816/frame.html (accessed.
155. Penta de Peppo A, Pierri MD, Scafuri A, et al. Intraoperative antifibrinolysis and blood-saving techniques in cardiac surgery. Prospective trial of 3 antifibrinolytic drugs. *Texas Heart Institute journal* 1995; **22**(3): 231-6.
156. Pertlícek J, Stehlík J, Sadovský P, Musil D, Mezera V. The Effect of Tranexamic Acid on Blood Loss after Primary Unilateral Total Knee Arthroplasty. Prospective Single-Centre Study. *Acta chirurgiae orthopaedicae ET traumatologiae cechoslovaca*, 2015. http://cochranelibrary-wiley.com/o/cochrane/clcentral/articles/361/CN-01200361/frame.html (accessed.
157. Pinosky ML, Kennedy DJ, Fishman RL, et al. Tranexamic acid reduces bleeding after cardiopulmonary bypass when compared to epsilon aminocaproic acid and placebo. *J Card Surg* 1997; **12**(5): 330-8.
158. Pourfakhr P, Gatavi E, Etezadi F, et al. Local administration of tranexamic acid during prostatectomy surgery: Effects on reducing the amount of bleeding. *Nephro-Urology Monthly* 2016; **8**(6).
159. Prabhu T, Deepak M, Harish R, Narasimhan V. Efficacy of tranexamic acid in conservation of blood loss in total knee arthroplasty patients. *Research journal of pharmaceutical, biological and chemical sciences*, 2015. http://cochranelibrary-wiley.com/o/cochrane/clcentral/articles/237/CN-01125237/frame.html (accessed.
160. Pugh SC, Wielogorski AK. A comparison of the effects of tranexamic acid and low-dose aprotinin on blood loss and homologous blood usage in patients undergoing cardiac surgery. *J Cardiothorac Vasc Anesth* 1995; **9**(3): 240-4.
161. Raksakietisak M, Sathitkarnmanee B, Srisaen P, et al. Two Doses of Tranexamic Acid Reduce Blood Transfusion in Complex Spine Surgery: a Prospective Randomized Study. *Spine*, 2015. http://cochranelibrary-wiley.com/o/cochrane/clcentral/articles/943/CN-01258943/frame.html (accessed.
162. Rannikko A, Petas A, Taari K. Tranexamic acid in control of primary hemorrhage during transurethral prostatectomy. *Urology* 2004; **64**(5): 955-8.
163. Reid RW, Zimmerman AA, Laussen PC, Mayer JE, Gorlin JB, Burrows FA. The efficacy of tranexamic acid versus placebo in decreasing blood loss in pediatric patients undergoing repeat cardiac surgery. *Anesth Analg* 1997; **84**(5): 990-6.

164. Reyes G, Alvarez P, Bustamante J, et al. Do cell saver systems reduce the need of transfusion in low risk patients undergoing cardiac surgery? *Interactive Cardiovascular and Thoracic Surgery* 2010; **10**.
165. Rollo VJ, Chao W, Hozack WJ, Rothman RH, Eng KO. Prospective randomized evaluation of blood salvage techniques for primary total hip arthroplasty. *Journal of Arthroplasty* 1995; **10**(4): 532-9.
166. Royston D, von Kier S. Reduced haemostatic factor transfusion using heparinase-modified thrombelastography during cardiopulmonary bypass. *Br J Anaesth* 2001; **86**(4): 575-8.
167. Sa-Ngasoongsong P, Channoom T, Kawinwonggowit V, et al. Postoperative blood loss reduction in computer-assisted surgery total knee replacement by low dose intra-articular tranexamic acid injection together with 2-hour clamp drain: a prospective triple-blinded randomized controlled trial. *Orthopedic reviews* 2011; **3**(2): e12.
168. Santos AT, Kalil RA, Bauemann C, Pereira JB, Nesralla IA. A randomized, double-blind, and placebo-controlled study with tranexamic acid of bleeding and fibrinolytic activity after primary coronary artery bypass grafting. *Braz J Med Biol Res* 2006; **39**(1): 63-9.
169. Sarkanovic ML, Gvozdenovic L, Savic D, Ilic MP, Jovanovic G. Autologous blood transfusion in total knee replacement surgery. *Vojnosanitetski Pregled* 2013; **70**(3): 274-8.
170. Savvidou C, Pilichou A, Pneumaticos SG, Chatziioannou SN. Efficacy and cost-effectiveness of cell saving blood autotransfusion in adult lumbar fusion. *Transfusion Medicine* 2009; **19**(4): 202-6.
171. Seddighi A, Nikouei A, Seddighi AS, et al. The role of tranexamic acid in prevention of hemorrhage in major spinal surgeries. *Asian journal of neurosurgery* 2017; **12**(3): 501-5.
172. Seo J, Moon Y, Park S, Kim S, Ko K. The comparative efficacies of intra-articular and IV tranexamic acid for reducing blood loss during total knee arthroplasty. *Knee surgery, sports traumatology, arthroscopy*, 2013. http://cochranelibrary-wiley.com/o/cochrane/clcentral/articles/714/CN-01124714/frame.html https://link.springer.com/content/pdf/10.1007%2Fs00167-012-2079-2.pdf (accessed.
173. Sethna NF, Zurakowski D, Brustowicz RM, Bacsik J, Sullivan LJ, Shapiro F. Tranexamic acid reduces intraoperative blood loss in pediatric patients undergoing scoliosis surgery. *Anesthesiology* 2005; **102**(4): 727-32.
174. Shehata N, Burns LA, Nathan H, et al. A randomized controlled pilot study of adherence to transfusion strategies in cardiac surgery. *Transfusion* 2012; **52**(1): 91-9.
175. Shenolikar A, Wareham K, Newington D, Thomas D, Hughes J, Downes M. Cell salvage auto transfusion in total knee replacement surgery. *Transfusion Medicine* 1997; **7**(4): 277-80.
176. Shimizu K, Toda Y, Iwasaki T, et al. Effect of tranexamic acid on blood loss in pediatric cardiac surgery: A randomized trial. *Journal of Anesthesia* 2011; **25**(6): 823-30.
177. Shore-Lesserson L, Reich DL, Vela-Cantos F, Ammar T, Ergin MA. Tranexamic acid reduces transfusions and mediastinal drainage in repeat cardiac surgery. *Anesth Analg* 1996; **83**(1): 18-26.
178. Shore-Lesserson L, Manspeizer HE, DePerio M, Francis S, Vela-Cantos F, Ergin MA. Thromboelastography-guided transfusion algorithm reduces transfusions in complex cardiac surgery. *Anesth Analg* 1999; **88**(2): 312-9.
179. Spark JI, Chetter IC, Kester RC, Scott DJ. Allogeneic versus autologous blood during abdominal aortic aneurysm surgery. *European Journal of Vascular & Endovascular Surgery* 1997; **14**(6): 482-6.
180. Speekenbrink RG, Vonk AB, Wildevuur CR, Eijsman L. Hemostatic efficacy of dipyridamole, tranexamic acid, and aprotinin in coronary bypass grafting. *Ann Thorac Surg* 1995; **59**(2): 438-42.
181. Stowers M, Aoina J, Vane A, Poutawera V, Hill A, Munro J. Tranexamic Acid in Knee Surgery Study-A Multicentered, Randomized, Controlled Trial. *Journal of arthroplasty*, 2017. http://cochranelibrary-wiley.com/o/cochrane/clcentral/articles/450/CN-01604450/frame.html https://ac.els-cdn.com/S0883540317304989/1-s2.0-S0883540317304989-main.pdf?_tid=a53dc724-8792-4a1f-ad3f-8541c7de0007&acdnat=1535708776_7a671c8510d4de6db48a73defd629eb4 (accessed.
182. Taghaddomi RJ, Mirzaee A, Attar AS, Shirdel A. Tranexamic acid reduces blood loss in off-pump coronary artery bypass surgery. *J Cardiothorac Vasc Anesth* 2009; **23**(3): 312-5.

183. Tanaka N, Sakahashi H, Sato E, Hirose K, Ishima T, Ishii S. Timing of the administration of tranexamic acid for maximum reduction in blood loss in arthroplasty of the knee. *J Bone Joint Surg Br* 2001; **83**(5): 702-5.
184. Tempe D, Bajwa R, Cooper A, et al. Blood conservation in small adults undergoing valve surgery. *Journal of Cardiothoracic & Vascular Anesthesia* 1996; **10**(4): 502-6.
185. Tempe DK, Banerjee A, Virmani S, et al. Comparison of the effects of a cell saver and low-dose aprotinin on blood loss and homologous blood usage in patients undergoing valve surgery. *Journal of Cardiothoracic & Vascular Anesthesia* 2001; **15**(3): 326-30.
186. Tengberg P, Foss N, Palm H, Kallemose T, Troelsen A. Tranexamic acid reduces blood loss in patients with extracapsular fractures of the hip: results of a randomised controlled trial. *The bone & joint journal*, 2016. http://cochranelibrary-wiley.com/o/cochrane/clcentral/articles/776/CN-01263776/frame.html (accessed.
187. Thomas D, Wareham K, Cohen D, Hutchings H. Autologous blood transfusion in total knee replacement surgery. *British Journal of Anaesthesia* 2001; **86**(5): 669-73.
188. Thomassen BJW, Pilot P, Scholtes VAB, et al. Limit Allogeneic Blood Use with Routine Re-use of Patient's Own Blood: A Prospective, Randomized, Controlled Trial in Total Hip Surgery. *PLoS ONE* 2012; **7**(9).
189. Tsutsumimoto T, Shimogata M, Ohta H, Yui M, Yoda I, Misawa H. Tranexamic Acid reduces perioperative blood loss in cervical laminoplasty: a prospective randomized study. *Spine (03622436)* 2011; **36**(23): 1913-8.
190. Ugurlu M, Aksekili M, Ça?lar C, Yüksel K, ahin E, Akyol M. Effect of Topical and Intravenously Applied Tranexamic Acid Compared to Control Group on Bleeding in Primary Unilateral Total Knee Arthroplasty. *Journal of knee surgery*, 2017. https://www.thieme-connect.de/DOI/DOI?10.1055/s-0036-1583270 (accessed.
191. Uozaki Y, Watanabe G, Kotou K, Ueyama K, Doi Y, Misaki T. Effect of tranexamic acid on blood loss reduction after cardiopulmonary bypass. *Jpn J Thorac Cardiovasc Surg* 2001; **49**(5): 273-8.
192. Vanek T, Jares M, Fajt R, et al. Fibrinolytic inhibitors in off-pump coronary surgery: a prospective, randomized, double-blind TAP study (tranexamic acid, aprotinin, placebo). *Eur J Cardiothorac Surg* 2005; **28**(4): 563-8.
193. Veien M, Sorensen JV, Madsen F, Juelsgaard P. Tranexamic acid given intraoperatively reduces blood loss after total knee replacement: a randomized, controlled study. *Acta Anaesthesiol Scand* 2002; **46**(10): 1206-11.
194. Vermeijden WJ, Van Klarenbosch J, Gu YJ, et al. Effects of cell-saving devices and filters on transfusion in cardiac surgery: A multicenter randomized study. *Annals of Thoracic Surgery* 2015; **99**(1): 26-32.
195. Virani S, Dahapute A, Panda I, Bava S. Role of local infiltration of tranexamic acid in reducing blood loss in peritrochanteric fracture surgery in the elderly population. *Malaysian orthopaedic journal*, 2016. http://cochranelibrary-wiley.com/o/cochrane/clcentral/articles/345/CN-01331345/frame.html https://www.ncbi.nlm.nih.gov/pmc/articles/PMC5333680/pdf/moj-10-026.pdf (accessed.
196. Wang SC, Shieh JF, Chang KY, et al. Thromboelastography-guided transfusion decreases intraoperative blood transfusion during orthotopic liver transplantation: randomized clinical trial. *Transplant Proc* 2010; **42**(7): 2590-3.
197. Weber CF, Gorlinger K, Meininger D, et al. Point-of-care testing: a prospective, randomized clinical trial of efficacy in coagulopathic cardiac surgery patients. *Anesthesiology* 2012; **117**(3): 531-47.
198. Wei M, Jian K, Guo Z, et al. Tranexamic acid reduces postoperative bleeding in off-pump coronary artery bypass grafting. *Scand Cardiovasc J* 2006; **40**(2): 105-9.
199. Westbrook AJ, Olsen J, Bailey M, Bates J, Scully M, Salamonsen RF. Protocol based on thromboelastograph (TEG) out-performs physician preference using laboratory coagulation tests to guide blood replacement during and after cardiac surgery: a pilot study. *Heart Lung Circ* 2009; **18**(4): 277-88.
200. Wong J, El Beheiry H, Rampersaud YR, et al. Tranexamic Acid reduces perioperative blood loss in adult patients having spinal fusion surgery. *Anesth Analg* 2008; **107**(5): 1479-86.
201. Wu CC, Ho WM, Cheng SB, et al. Perioperative parenteral tranexamic acid in liver tumor resection: a prospective randomized trial toward a "blood transfusion"-free hepatectomy. *Ann Surg* 2006; **243**(2): 173-80.
202. Xu C, Wu A, Yue Y. Which is more effective in adolescent idiopathic scoliosis surgery: batroxobin, tranexamic acid or a combination? *Archives of orthopaedic and trauma surgery*, 2012. http://cochranelibrary-wiley.com/o/cochrane/clcentral/articles/447/CN-00882447/frame.html

https://link.springer.com/content/pdf/10.1007%2Fs00402-011-1390-6.pdf (accessed.
203. Xu X, Li X, Liu W, Wang Z. Longtime soaking of high concentration tranexamic acid in total hip arthroplasty: A prospective randomized controlled trial in 224 patients. *Pakistan journal of medical sciences* 2015; **31**(6): 1306-11.
204. Xu H, Duan Y, Yuan X, Wu H, Sun H, Ji H. Intravenous Iron Versus Placebo in the Management of Postoperative Functional Iron Deficiency Anemia in Patients Undergoing Cardiac Valvular Surgery: A Prospective, Single-Blinded, Randomized Controlled Trial. *Journal of Cardiothoracic and Vascular Anesthesia* 2019.
205. Yassen K, Bellamy M, Sadek S, Webster N. Tranexamic acid reduces blood loss during orthotopic liver transplantation. *Clinical Transplantation* 1993; **7**: 453-8.
206. Zabeeda D, Medalion B, Sverdlov M, et al. Tranexamic acid reduces bleeding and the need for blood transfusion in primary myocardial revascularization. *The Annals of Thoracic Surgery* 2002; **74**(3): 733-8.
207. Zhao H, Zhao Z, Quan X, Cheng Z, Ma H, Meng L. Application of autologous blood cell salvage in off-pump coronary artery bypass graft operation. *Heart Surgery Forum* 2017; **20**(3).
208. Zhao H, Xiang M, Shi X, Pei FX, Kang P, Xia Y. Efficacy of oral tranexamic acid on blood loss in primary total hip arthroplasty using a direct anterior approach: a prospective randomized controlled trial. *International Orthopaedics* 2018: 1-8.
209. Zohar E, Ellis M, Ifrach N, Stern A, Sapir O, Fredman B. The postoperative blood-sparing efficacy of oral versus intravenous tranexamic acid after total knee replacement. *Anesth Analg* 2004; **99**(6): 1679-83, table of contents.
210. Zufferey PJ, Miquet M, Quenet S, et al. Tranexamic acid in hip fracture surgery: a randomized controlled trial. *Br J Anaesth* 2010; **104**(1): 23-30.
211. Slagis SV, Benjamin JB, Volz RG, Giordano GF. Postoperative blood salvage in total hip and knee arthroplasty: A randomised controlled trial. *Journal of Bone and Joint Surgery - Series B* 1991; **73**(4): 591-4.
212. Aguilera X, Jordan M, Gonzalez JC, et al. Topical and intravenous tranexamic acid reduce blood loss compared to routine hemostasis in total knee arthroplasty: a multicenter, randomized, controlled trial. *Archives of Orthopaedic and Trauma Surgery* 2015; **135**(7): 1017-25.
213. Ak K, Isbir CS, Tetik S, et al. Thromboelastography-based transfusion algorithm reduces blood product use after elective CABG: a prospective randomized study. *J Card Surg* 2009; **24**(4): 404-10.
214. Alizadeh Ghavidel A, Totonchi Z, Chitsazan M, et al. Safety and efficacy of caproamin fides and tranexamic Acid versus placebo in patients undergoing coronary artery revascularization. *J Cardiovasc Thorac Res* 2014; **6**(3): 197-202.
215. Apipan B, Rummasak D, Narainthonsaenee T. The effect of different dosage regimens of tranexamic acid on blood loss in bimaxillary osteotomy: a randomized, double-blind, placebo-controlled study. *International journal of oral and maxillofacial surgery*, 2017. (accessed.
216. Arantes G, Pereira R, Melo D, Alonso N, Duarte M. Effectiveness of tranexamic acid for reducing intraoperative bleeding in palatoplasties: a randomized clinical trial. *Journal of cranio-maxillofacial surgery (no pagination), 2016*, 2016. (accessed.
217. Ausen K, Fossmark R, Spigset O, Pleym H. Randomized clinical trial of topical tranexamic acid after reduction mammoplasty. *British journal of surgery*, 2015. (accessed.
218. Bansal A, Arora A. A double-blind, placebo-controlled randomized clinical trial to evaluate the efficacy of tranexamic acid in irrigant solution on blood loss during percutaneous nephrolithotomy: a pilot study from tertiary care center of North India. *World journal of urology*, 2017. (accessed.
219. Baradaranfar M, Dadgarnia M, Mahmoudi H, et al. The effect of topical tranexamic acid on bleeding reduction during functional endoscopic sinus surgery. *Iranian journal of otorhinolaryngology*, 2017. (accessed.
220. Barrachina B, Fondarella A, Iriarte I, et al. Tranexamic acid compared with placebo for reducing total blood loss in hip replacement surgery: A randomized clinical trial. *Anesthesia and Analgesia* 2016; **122**(4): 986-95.
221. Baruah R, Borah P, Haque R. Use of tranexamic acid in dynamic hip screw plate fixation for trochanteric fractures. *Journal of orthopaedic surgery (hong kong)*, 2016. (accessed.
222. Benoni G, Fredin H. Fibrinolytic inhibition with tranexamic acid reduces blood loss and blood transfusion after knee arthroplasty: a prospective, randomised, double-blind study of 86 patients. *J Bone Joint Surg Br* 1996; **78**(3): 434-40.

223. Benoni G, Lethagen S, Nilsson P, Fredin H. Tranexamic acid, given at the end of the operation, does not reduce postoperative blood loss in hip arthroplasty. *Acta Orthop Scand* 2000; **71**(3): 250-4.
224. Bernabeu-Wittel M, Romero M, Ollero-Baturone M, et al. Ferric carboxymaltose with or without erythropoietin in anemic patients with hip fracture: a randomized clinical trial. *Transfusion* 2016; **56**(9): 2199-211.
225. Bidolegui F, Arce G, Lugones A, Pereira S, Vindver G. Tranexamic Acid Reduces Blood Loss and Transfusion in Patients Undergoing Total Knee Arthroplasty without Tourniquet: a Prospective Randomized Controlled Trial. *Open orthopaedics journal*, 2014. (accessed.
226. Campbell J, Holland C, Richens D, Skinner H. Impact of cell salvage during cardiac surgery on the thrombelastomeric coagulation profile: a pilot study. *Perfusion* 2012; **27**(3): 221-4.
227. Carvalho L, Frois TE, Machado SL, Gonçalves M, Paiva CL, Tavares dSM. Bleeding reduction after topical application of tranexamic acid together with Betadine solution in total knee arthroplasty. A randomised controlled study. *Orthopaedics & traumatology, surgery & research : OTSR*, 2015. http://cochranelibrary-wiley.com/o/cochrane/clcentral/articles/638/CN-01052638/frame.html https://ac.els-cdn.com/S1877056814003168/1-s2.0-S1877056814003168-main.pdf?_tid=ea534ac4-9b8b-44cc-b321-c969482e8978&acdnat=1535708384_66656132f161e5da8be8cf3e1b083274 (accessed.
228. Castro-Menéndez M, Pena-Paz S, Rocha-García F, Rodríguez-Casas N, Huici-Izco R, Montero-Viéites A. Efficacy of 2 grammes of intravenous transexamic acid in the reduction of post-surgical bleeding after total hip and knee replacement. *Revista espanola de cirugia ortopedica y traumatologia*, 2016. http://cochranelibrary-wiley.com/o/cochrane/clcentral/articles/810/CN-01368810/frame.html (accessed.
229. Chareancholvanich K, Siriwattanasakul P, Narkbunnam R, Pornrattanamaneewong C. Temporary clamping of drain combined with tranexamic acid reduce blood loss after total knee arthroplasty: a prospective randomized controlled trial. *BMC musculoskeletal disorders*, 2012. http://cochranelibrary-wiley.com/o/cochrane/clcentral/articles/038/CN-00842038/frame.html https://www.ncbi.nlm.nih.gov/pmc/articles/PMC3416573/pdf/1471-2474-13-124.pdf (accessed.
230. Charoencholvanich K, Siriwattanasakul P. Tranexamic acid reduces blood loss and blood transfusion after TKA: a prospective randomized controlled trial. *Clinical orthopaedics and related research* 2011; **469**(10): 2874-80.
231. Chaudhary F, Pervaz Z, Ilyas S, Niaz M. Topical use of tranexamic acid in open heart surgery. *Journal of the pakistan medical association*, 2018. http://cochranelibrary-wiley.com/o/cochrane/clcentral/articles/977/CN-01464977/frame.html (accessed.
232. Chen CC, Wang CC, Wang CP, Lin TH, Lin WD, Liu SA. Prospective, randomized, controlled trial of tranexamic acid in patients who undergo head and neck procedures. *Otolaryngol Head Neck Surg* 2008; **138**(6): 762-7.
233. Chen X, Cao X, Yang C, Guo K, Zhu Q, Zhu J. Effectiveness and Safety of Fixed-Dose Tranexamic Acid in Simultaneous Bilateral Total Knee Arthroplasty: a Randomized Double-Blind Controlled Trial. *Journal of arthroplasty*, 2016. http://cochranelibrary-wiley.com/o/cochrane/clcentral/articles/796/CN-01413796/frame.html https://ac.els-cdn.com/S0883540316300511/1-s2.0-S0883540316300511-main.pdf?_tid=ede0afce-e442-44a1-a364-60b6afaae4ad&acdnat=1535708505_cc70fb0295562d31e46fb22958a01d4c (accessed.
234. Cholette JM, Powers KS, Daugherty LE, et al. Transfusion of cell saver salvaged blood in neonates and infants undergoing open heart surgery significantly reduces RBC and coagulant product transfusions and donor exposures: Results of a prospective, randomized, clinical trial. *Pediatric Critical Care Medicine* 2013; **14**(2): 137-47.
235. Cip J, Widemschek M, Martin A, Benesch T, Waibel R. Does single use of an autologous transfusion system in TKA reduce the need for allogenic blood?: A prospective randomized trial general. *Clinical Orthopaedics and Related Research* 2013; **471**(4): 1319-25.
236. Colomina M, Koo M, Basora M, Pizones J, Mora L, Bago J. Intraoperative tranexamic acid use in major spine surgery in adults: a multicentre, randomized, placebo-controlled trial. *British journal of anaesthesia*, 2017. http://cochranelibrary-wiley.com/o/cochrane/clcentral/articles/703/CN-01370703/frame.html http://diposit.ub.edu/dspace/bitstream/2445/108163/1/667959.pdf (accessed.
237. Crescenti A, Borghi G, Bignami E, et al. Intraoperative administration of tranexamic acid to reduce transfusion rate in patients undergoing radical retropubic prostatectomy: double blind, randomised, placebo controlled trial. *BMJ: British Medical Journal (Overseas & Retired Doctors Edition)* 2011; **343**(7829): 885-.

238. Das A, Chattopadhyay S, Mandal D, et al. Does the preoperative administration of tranexamic acid reduce perioperative blood loss and transfusion requirements after head neck cancer surgery? A randomized, controlled trial. *Anesthesia, essays and researches* 2015; **9**(3): 384-90.
239. de Almeida JP, Vincent JL, Galas FR, et al. Transfusion requirements in surgical oncology patients: a prospective, randomized controlled trial. *Anesthesiology* 2015; **122**(1): 29-38.
240. De Napoli G, Ottolenghi J, Melo LM. Comparison of bleeding and transfusions in primary hip and knee arthroplasties with single doses of tranexamic acid vs. placebo in a University Hospital. A prospective study. *Revista Colombiana de Ortopedia y Traumatologia* 2016; **30**(3): 101-6.
241. Dell'Atti L, Stefano P, Gaetano C, Carmelo I. Efficacy of a short prophylaxis with tranexamic acid on hemostasis during transrectal prostate biopsy in patients taking oral anti-platelet treatment. *Journal of BUON*, 2016. http://cochranelibrary-wiley.com/o/cochrane/clcentral/articles/999/CN-01248999/frame.html (accessed.
242. Digas G, Koutsogiannis I, Meletiadis G, Antonopoulou E, Karamoulas V, Bikos C. Intra-articular injection of tranexamic acid reduce blood loss in cemented total knee arthroplasty. *European journal of orthopaedic surgery & traumatology : orthopedie traumatologie*, 2015. http://cochranelibrary-wiley.com/o/cochrane/clcentral/articles/259/CN-01259259/frame.html https://link.springer.com/content/pdf/10.1007%2Fs00590-018-2147-5.pdf (accessed.
243. Drakos A, Raoulis V, Karatzios K, et al. Efficacy of Local Administration of Tranexamic Acid for Blood Salvage in Patients Undergoing Intertrochanteric Fracture Surgery. *Journal of orthopaedic trauma*, 2016. http://cochranelibrary-wiley.com/o/cochrane/clcentral/articles/036/CN-01177036/frame.html (accessed.
244. Drosos G, Ververidis A, Valkanis C, et al. A randomized comparative study of topical versus intravenous tranexamic acid administration in enhanced recovery after surgery (ERAS) total knee replacement. *Journal of orthopaedics*, 2016. http://cochranelibrary-wiley.com/o/cochrane/clcentral/articles/892/CN-01141892/frame.html https://www.ncbi.nlm.nih.gov/pmc/articles/PMC4821446/pdf/main.pdf (accessed.
245. Edwards TJ, Noble EJ, Durran A, Mellor N, Hosie KB. Randomized clinical trial of preoperative intravenous iron sucrose to reduce blood transfusion in anaemic patients after colorectal cancer surgery. *Br J Surg* 2009; **96**(10): 1122-8.
246. Eldaba AA, Amr YM, Albirmawy OA. Effects of tranexamic acid during endoscopic sinsus surgery in children. *Saudi Journal of Anaesthesia* 2013; **7**(3): 229-33.
247. Elshamaa H, Elokda S. Effect of activated recombinant factor VII versus tranexamic acid infusion on bleeding during spine surgery, randomized, controlled, double blinded trial. *Egyptian journal of anaesthesia*, 2015. http://cochranelibrary-wiley.com/o/cochrane/clcentral/articles/560/CN-01070560/frame.html https://ac.els-cdn.com/S1110184915000021/1-s2.0-S1110184915000021-main.pdf?_tid=a0086a4a-1331-4f57-940c-463a0f3e7ca8&acdnat=1535708461_24ec3c39d0aa0c32ecac136019ea6abb (accessed.
248. Elwatidy S, Jamjoom Z, Elgamal E, Zakaria A, Turkistani A, El-Dawlatly A. Efficacy and safety of prophylactic large dose of tranexamic acid in spine surgery: a prospective, randomized, double-blind, placebo-controlled study. *Spine (Phila Pa 1976)* 2008; **33**(24): 2577-80.
249. Emara WM, Moez KK, Elkhouly AH. Topical versus intravenous tranexamic acid as a blood conservation intervention for reduction of post-operative bleeding in hemiarthroplasty. *Anesthesia, essays and researches* 2014; **8**(1): 48-53.
250. Esfandiari B, Bistgani M, Kabiri M. Low dose tranexamic acid effect on post-coronary artery bypass grafting bleeding. *Asian cardiovascular & thoracic annals*, 2013. http://cochranelibrary-wiley.com/o/cochrane/clcentral/articles/410/CN-00911410/frame.html (accessed.
251. Fan YX, Liu FF, Jia M, et al. Comparison of restrictive and liberal transfusion strategy on postoperative delirium in aged patients following total hip replacement: a preliminary study. *Arch Gerontol Geriatr* 2014; **59**(1): 181-5.
252. Faraoni D, Cacheux C, Van Aelbrouck C, Ickx BE, Barvais L, Levy JH. Effect of two doses of tranexamic acid on fibrinolysis evaluated by thromboelastography during cardiac surgery: a randomised, controlled study. *Eur J Anaesthesiol* 2014; **31**(9): 491-8.
253. Farrokhi MR, Kazemi AP, Eftekharian HR, Akbari K. Efficacy of prophylactic low dose of tranexamic Acid in spinal fixation surgery: a randomized clinical trial. *Journal of Neurosurgical Anesthesiology* 2011; **23**(4): 290-6.
254. Fernández-Cortiñas A, Quintáns-Vázquez J, Gómez-Suárez F, Murillo O, Sánchez-López B, Pena-Gracía J. Effect of tranexamic acid administration on bleeding in primary total hip arthroplasty. *Revista espanola de cirugia ortopedica y traumatologia*, 2017. http://cochranelibrary-wiley.com/o/cochrane/clcentral/articles/420/CN-01604420/frame.html (accessed.

255. Foss NB, Kristensen MT, Jensen PS, Palm H, Krasheninnikoff M, Kehlet H. The effects of liberal versus restrictive transfusion thresholds on ambulation after hip fracture surgery. *Transfusion* 2009; **49**(2): 227-34.
256. Fraval A, Effeney P, Fiddelaers L, Smith B, Towell B, Tran P. OBTAIN A: outcome Benefits of Tranexamic Acid in Hip Arthroplasty. A Randomized Double-Blinded Controlled Trial. *Journal of arthroplasty (no pagination), 2016*, 2016. http://cochranelibrary-wiley.com/o/cochrane/clcentral/articles/716/CN-01299716/frame.html https://ac.els-cdn.com/S0883540316308452/1-s2.0-S0883540316308452-main.pdf?_tid=568453f8-f8a6-46c5-b973-7e683556b1a3&acdnat=1535708653_bcbbf449b1501753c1d44ae7936b4ac6 (accessed.
257. Fraval A, Duncan S, Murray T, Duggan J, Tirosh O, Tran P. OBTAIN E: outcome benefits of tranexamic acid in hip arthroplasty with enoxaparin: a randomised double-blinded controlled trial. *Hip Int* 2018: 1120700018780125.
258. Froessler B, Palm P, Weber I, Hodyl NA, Singh R, Murphy EM. The Important Role for Intravenous Iron in Perioperative Patient Blood Management in Major Abdominal Surgery: A Randomized Controlled Trial. *Annals of surgery* 2016; **264**(1): 41-6.
259. Garrido-Martin P, Nassar-Mansur MI, de la Llana-Ducros R, et al. The effect of intravenous and oral iron administration on perioperative anaemia and transfusion requirements in patients undergoing elective cardiac surgery: a randomized clinical trial. *Interact Cardiovasc Thorac Surg* 2012; **15**(6): 1013-8.
260. Gatling J, Ramsingh D, Horricks J, et al. Blood conservation using tranexamic acid versus epsilon aminocaproic acid in cardiac surgery: A randomized controlled trial. *Journal of Anesthesia and Perioperative Medicine* 2018; **5**(4): 169-75.
261. Gautam V, Sambandam B, Singh S, Gupta P, Gupta R, Maini L. The role of tranexamic acid in reducing blood loss in total knee replacement. *Journal of clinical orthopaedics and trauma*, 2013. http://cochranelibrary-wiley.com/o/cochrane/clcentral/articles/588/CN-00907588/frame.html https://www.ncbi.nlm.nih.gov/pmc/articles/PMC3880537/pdf/main.pdf (accessed.
262. Geng T, Chen Y, Zhang L. Safety and efficacy of tranexamic acid in the application of spinal tuberculosis surgery. *International journal of clinical and experimental medicine*, 2017. http://cochranelibrary-wiley.com/o/cochrane/clcentral/articles/997/CN-01367997/frame.html (accessed.
263. Girdauskas E, Kempfert J, Kuntze T, et al. Thromboelastometrically guided transfusion protocol during aortic surgery with circulatory arrest: a prospective, randomized trial. *J Thorac Cardiovasc Surg* 2010; **140**(5): 1117-24 e2.
264. Guerreiro JPF, Badaro BS, Balbino JRM, Danieli MV, Queiroz AO, Cataneo DC. Application of Tranexamic Acid in Total Knee Arthroplasty - Prospective Randomized Trial. *The open orthopaedics journal* 2017; **11**: 1049-57.
265. Gupta K, Rastogi B, Krishan A, Gupta A, Singh V, Agarwal S. The prophylactic role of tranexamic acid to reduce blood loss during radical surgery: a prospective study. *Albang maqalat wa abhat fi altahdir waalinas*, 2012. http://cochranelibrary-wiley.com/o/cochrane/clcentral/articles/656/CN-01076656/frame.html http://www.aeronline.org/article.asp?issn=0259-1162;year=2012;volume=6;issue=1;spage=70;epage=73;aulast=Gupta (accessed.
266. Guzel Y, Gurcan O, Golge U, Dulgeroglu T, Metineren H. Topical tranexamic acid versus autotransfusion after total knee arthroplasty. *Journal of orthopaedic surgery (hong kong)*, 2016. http://cochranelibrary-wiley.com/o/cochrane/clcentral/articles/539/CN-01211539/frame.html (accessed.
267. Haghighi M, Ettehad H, Mardani-Kivi M, et al. Does tranexamic acid reduce bleeding during femoral fracture operation? *Archives of bone and joint surgery*, 2017. http://cochranelibrary-wiley.com/o/cochrane/clcentral/articles/132/CN-01338132/frame.html https://www.ncbi.nlm.nih.gov/pmc/articles/PMC5410741/pdf/ABJS-5-103.pdf (accessed.
268. Hashemi J, Ghaffari Nejad MH, Baharestani B, Esfandiari R, Panahipoor A. Evaluation and comparison of use of low-dose aprotinin and tranexamic acid in CABG: A double-blind, prospective, randomized study of 150 patients. *Iranian Heart Journal* 2011; **12**(1): 40-4.
269. Hogan M, Needham A, Ortmann E, et al. Haemoconcentration of residual cardiopulmonary bypass blood using Hemosep : a randomised controlled trial. *Anaesthesia* 2015; **70**(5): 563-70.
270. Hooda B, Chouhan R, Rath G, Bithal P, Suri A, Lamsal R. Effect of tranexamic acid on intraoperative blood loss and transfusion requirements in patients undergoing excision of intracranial meningioma. *Journal of clinical neuroscience*, 2017. http://cochranelibrary-wiley.com/o/cochrane/clcentral/articles/605/CN-01443605/frame.html https://ac.els-cdn.com/S0967586816314916/1-s2.0-S0967586816314916-main.pdf?_tid=2e98fb31-9bf5-4e07-9569-d55b8d5e4d90&acdnat=1535708492_56e1bef7a852c8c5a0644920c930ce4a (accessed.

271. Horstmann WG, Swierstra MJ, Ohanis D, Castelein RM, Kollen BJ, Verheyen CC. Reduction of blood loss with the use of a new combined intra-operative and post-operative autologous blood transfusion system compared with no drainage in primary total hip replacement. *Bone & Joint Journal* 2013; **95-B**(5): 616-22.
272. Hosseini H, Rahimianfar AA, Abdollahi MH, et al. Evaluations of topical application of tranexamic acid on post-operative blood loss in off-pump coronary artery bypass surgery. *Saudi J Anaesth* 2014; **8**(2): 224-8.
273. Hsu C, Lin P, Kuo F, Wang J. A regime of two intravenous injections of tranexamic acid reduces blood loss in minimally invasive total hip arthroplasty: a prospective randomised double-blind study. *The bone & joint journal*, 2015. http://cochranelibrary-wiley.com/o/cochrane/clcentral/articles/704/CN-01085704/frame.html (accessed.
274. Huang G, Jia X, Xiang Z, et al. Tranexamic Acid Reduces Hidden Blood Loss in Patients Undergoing Total Knee Arthroplasty: a Comparative Study and Meta-Analysis. *Medical science monitor*, 2016. http://cochranelibrary-wiley.com/o/cochrane/clcentral/articles/015/CN-01260015/frame.html https://www.ncbi.nlm.nih.gov/pmc/articles/PMC4790217/pdf/medscimonit-22-797.pdf (accessed.
275. Husted H, Blond L, Sonne-Holm S, Holm G, Jacobsen TW, Gebuhr P. Tranexamic acid reduces blood loss and blood transfusions in primary total hip arthroplasty: a prospective randomized double-blind study in 40 patients. *Acta Orthop Scand* 2003; **74**(6): 665-9.
276. Jendoubi A, Malouch A, Bouzouita A, et al. [Safety and efficacy of intravenous tranexamic acid in endoscopic transurethral resections in urology: Prospective randomized trial]. *Progres en urologie : journal de l'Association francaise d'urologie et de la Societe francaise d'urologie* 2017; **27**(16): 1036-42.
277. Jimenez JJ, Iribarren JL, Brouard M, et al. Safety and effectiveness of two treatment regimes with tranexamic acid to minimize inflammatory response in elective cardiopulmonary bypass patients: a randomized double-blind, dose-dependent, phase IV clinical trial. *Journal of cardiothoracic surgery* 2011; **6**: 138.
278. Johansson T, Pettersson LG, Lisander B. Tranexamic acid in total hip arthroplasty saves blood and money: a randomized, double-blind study in 100 patients. *Acta Orthop* 2005; **76**(3): 314-9.
279. Karaaslan F, Karaoglu S, Mermerkaya MU, Baktir A. Reducing blood loss in simultaneous bilateral total knee arthroplasty: combined intravenous-intra-articular tranexamic acid administration. A prospective randomized controlled trial. *Knee* 2015; **22**(2): 131-5.
280. Karaaslan F, Karaoglu S, Yurdakul E. Reducing Intra-articular Hemarthrosis After Arthroscopic Anterior Cruciate Ligament Reconstruction by the Administration of Intravenous Tranexamic Acid: A Prospective, Randomized Controlled Trial. *Am J Sports Med* 2015; **43**(11): 2720-6.
281. Kazemi SM, Mosaffa F, Eajazi A, et al. The effect of tranexamic acid on reducing blood loss in cementless total hip arthroplasty under epidural anesthesia. *Orthopedics* 2010; **33**(1): 17.
282. Kim K, Kim C, Kim Y, et al. The effectiveness of low-dose and high-dose tranexamic acid in posterior lumbar interbody fusion: a double-blinded, placebo-controlled randomized study. *European spine journal*, 2017. http://cochranelibrary-wiley.com/o/cochrane/clcentral/articles/358/CN-01604358/frame.html https://link.springer.com/content/pdf/10.1007%2Fs00586-017-5230-4.pdf (accessed.
283. Kim EJ, Kim YO, Shim KW, Ko BW, Lee JW, Koo B-N. Effects of Tranexamic Acid Based on its Population Pharmacokinetics in Pediatric Patients Undergoing Distraction Osteogenesis for Craniosynostosis: Rotational Thromboelastometry (ROTEM(TM)) Analysis. *International journal of medical sciences* 2018; **15**(8): 788-95.
284. Kimenai DM, Gerritse BM, Lucas C, et al. Effectiveness of pericardial lavage with or without tranexamic acid in cardiac surgery patients receiving intravenous tranexamic acid: a randomized controlled trial. *Eur J Cardiothorac Surg* 2016; **50**(6): 1124-31.
285. Kulkarni AP, Chaukar DA, Patil VP, Metgudmath RB, Hawaldar RW, Divatia JV. Does tranexamic acid reduce blood loss during head and neck cancer surgery? *Indian journal of anaesthesia* 2016; **60**(1): 19-24.
286. Kultufan Turan S, Aidinli B, Ayik I, et al. The role of rotational thromboelastgraphy on decision of blood transfusion in open heart surgery. *Göğüs-Kalp-Damar Anestezi ve Yoğun Bakım Derneği Dergisi* 2006; **12**: 154–9.
287. Kundu R, Das A, Basunia SR, Bhattacharyya T, Chattopadhyay S, Mukherjee A. Does a single loading dose of tranexamic acid reduce perioperative blood loss and transfusion requirements after total knee replacement surgery? A randomized, controlled trial. *Journal of natural science, biology, and medicine* 2015; **6**(1): 94-9.
288. Lack W, Crist B, Seymour R, Harvin W, Karunakar M. Effect of Tranexamic Acid on Transfusion: a Randomized Clinical Trial in Acetabular Fracture Surgery. *Journal of orthopaedic trauma*, 2017. http://cochranelibrary-wiley.com/o/cochrane/clcentral/articles/172/CN-01417172/frame.html (accessed.

289. Lacko M, Cellar R, Schreierova D, Vasko G. Comparison of intravenous and intra-articular tranexamic acid in reducing blood loss in primary total knee replacement. *Eklem hastaliklari ve cerrahisi [Joint diseases & related surgery]*, 2017. http://cochranelibrary-wiley.com/o/cochrane/clcentral/articles/196/CN-01454196/frame.html (accessed.
290. Laoruengthana A, Rattanaprichavej P, Chaibhuddanugul N, Varakornpipat P, Galassi M, Pongpirul K. Blood loss reduction: effect of different knee prosthesis designs and use of tranexamic acid-a randomized controlled trial. *European journal of orthopaedic surgery & traumatology : orthopedie traumatologie* 2019.
291. Lee QJ, Ching WY, Wong YC. Blood Sparing Efficacy of Oral Tranexamic Acid in Primary Total Knee Arthroplasty: A Randomized Controlled Trial. *Knee surgery & related research* 2017; **29**(1): 57-62.
292. Lei J, Zhang B, Cong Y, et al. Tranexamic acid reduces hidden blood loss in the treatment of intertrochanteric fractures with PFNA: a single-center randomized controlled trial. *J Orthop Surg Res* 2017; **12**(1): 124.
293. Liang J, Shen J, Fan Y, et al. Does intraoperative cell salvage system effectively decrease the need for allogeneic transfusions in scoliotic patients undergoing posterior spinal fusion? A prospective randomized study. *European Spine Journal* 2014.
294. Lidder PG, Sanders G, Whitehead E, et al. Pre-operative oral iron supplementation reduces blood transfusion in colorectal surgery - a prospective, randomised, controlled trial. *Ann R Coll Surg Engl* 2007; **89**(4): 418-21.
295. Lin P, Hsu C, Huang C, Chen W, Wang J. The blood-saving effect of tranexamic acid in minimally invasive total knee replacement: is an additional pre-operative injection effective? *Journal of bone and joint surgery British volume*, 2012. http://cochranelibrary-wiley.com/o/cochrane/clcentral/articles/851/CN-00879851/frame.html (accessed.
296. Liu W, Yang C, Huang X, Liu R. Tranexamic Acid Reduces Occult Blood Loss, Blood Transfusion, and Improves Recovery of Knee Function after Total Knee Arthroplasty: a Comparative Study. *Journal of knee surgery*, 2017. http://cochranelibrary-wiley.com/o/cochrane/clcentral/articles/821/CN-01374821/frame.html https://www.thieme-connect.de/DOI/DOI?10.1055/s-0037-1602248 (accessed.
297. Lundin E, Johansson T, Zachrisson H, et al. Single dose of tranexamic acid reduces blood loss and transfusions in surgery for advanced ovarian cancer. *International journal of gynecological cancer*, 2013. http://cochranelibrary-wiley.com/o/cochrane/clcentral/articles/169/CN-01058169/frame.html (accessed.
298. Luo X, He S, Lin Z, Li Z, Huang C, Li Q. Efficacy and Safety of Tranexamic Acid for Controlling Bleeding During Surgical Treatment of Intertrochanteric Fragility Fracture with Proximal Femoral Nail Anti-rotation: A Randomized Controlled Trial. *Indian journal of orthopaedics* 2019; **53**(2): 263-9.
299. Maniar R, Kumar G, Singhi T, Nayak R, Maniar P. Most effective regimen of tranexamic acid in knee arthroplasty: a prospective randomized controlled study in 240 patients. *Clinical orthopaedics and related research*, 2012. http://cochranelibrary-wiley.com/o/cochrane/clcentral/articles/879/CN-00880879/frame.html https://www.ncbi.nlm.nih.gov/pmc/articles/PMC3830076/pdf/11999_2012_Article_2310.pdf (accessed.
300. Mansouri M, Attary M, Bagheri K, Massoumi G, Ghavami B. Comparative evaluation of the effects of tranexamic acid and low-dose aprotinin on post-valvular heart surgery bleeding and allogenic transfusion. *Interactive cardiovascular and thoracic surgery* 2012; **15**(1): 23-7.
301. Martin J, Cassatt K, Kincaid-Cinnamon K, Westendorf D, Garton A, Lemke J. Topical administration of tranexamic acid in primary total hip and total knee arthroplasty. *Journal of arthroplasty*, 2014. http://cochranelibrary-wiley.com/o/cochrane/clcentral/articles/834/CN-00988834/frame.html https://ac.els-cdn.com/S0883540313007870/1-s2.0-S0883540313007870-main.pdf?_tid=631d075c-a618-452f-bd2a-6f0fc417128a&acdnat=1535708749_9aada27e956be182a0373b0c9e0b4647 (accessed.
302. McConnell JS, Shewale S, Munro NA, Shah K, Deakin AH, Kinninmonth AWG. Reduction of blood loss in primary hip arthroplasty with tranexamic acid or fibrin spray: A randomized controlled trial. *Acta Orthopaedica* 2011; **82**(6): 660-3.
303. Melo GLR, Lages DS, Madureira Junior JL, Pellucci GdP, Pellucci JWJ. The use of tranexamic acid in patients submitted to primary total hip arthroplasty: an evaluation of its impact in different administration protocols. *Revista brasileira de ortopedia* 2017; **52**: 34-9.
304. Meng Q-Q, Pan N, Xiong J-Y, Liu N. Tranexamic acid is beneficial for reducing perioperative blood loss in transurethral resection of the prostate. *Experimental and therapeutic medicine* 2019; **17**(1): 943-7.
305. Min P, Peng Y, Hu J, Gu Z. Efficacy and safety of tranexamic acid on blood loss after unilateral total knee arthroplasty. *Chinese journal of tissue engineering research*, 2015. http://cochranelibrary-wiley.com/o/cochrane/clcentral/articles/385/CN-01129385/frame.html

http://www.crter.org/CN/10.3969/j.issn.2095-4344.2015.17.006 (accessed.
306. Mirmohammadsadeghi A, Mirmohammadsadeghi M, Kheiri M. Does topical tranexamic acid reduce postcoronary artery bypass graft bleeding? *Journal of Research in Medical Sciences* 2018; **23**(1): 1-4.
307. Moller A, Nielsen HB, Wetterslev J, et al. Low vs. high hemoglobin trigger for Transfusion in Vascular surgery (TV): a randomized clinical feasibility trial. *Blood* 2019; **11**: 11.
308. Molloy DO, Archbold HA, Ogonda L, McConway J, Wilson RK, Beverland DE. Comparison of topical fibrin spray and tranexamic acid on blood loss after total knee replacement: a prospective, randomised controlled trial. *J Bone Joint Surg Br* 2007; **89**(3): 306-9.
309. Motififard M, Tahririan MA, Saneie M, Badiei S, Nemati A. Low Dose Perioperative Intravenous Tranexamic Acid in Patients Undergoing Total Knee Arthroplasty: A Double-Blind Randomized Placebo Controlled Clinical Trial. *Journal of blood transfusion* 2015; **2015**: 948304.
310. Na H, Shin H, Lee Y, Kim J, Koo K, Do S. The effect of tranexamic acid on blood coagulation in total hip replacement arthroplasty: rotational thromboelastographic (ROTEM®) analysis. *Anaesthesia*, 2016. http://cochranelibrary-wiley.com/o/cochrane/clcentral/articles/020/CN-01169020/frame.html https://onlinelibrary.wiley.com/doi/pdf/10.1111/anae.13270 (accessed.
311. Napoli G, Ottolenghi J, Melo L. Comparison of bleeding and transfusions in primary hip and knee arthroplasties with single doses of tranexamic acid vs. placebo in a University Hospital. A prospective study. *Revista colombiana de ortopedia y traumatologia*, 2016. http://cochranelibrary-wiley.com/o/cochrane/clcentral/articles/844/CN-01289844/frame.html (accessed.
312. Oremus K, Sostaric S, Trkulja V, Haspl M. Influence of tranexamic acid on postoperative autologous blood retransfusion in primary total hip and knee arthroplasty: a randomized controlled trial. *Transfusion*, 2014. http://cochranelibrary-wiley.com/o/cochrane/clcentral/articles/732/CN-00861732/frame.html https://onlinelibrary.wiley.com/doi/pdf/10.1111/trf.12224 (accessed.
313. Özta S, Öztürk A, Akalin Y, et al. The effect of local and systemic application of tranexamic acid on the amount of blood loss and allogeneic blood transfusion after total knee replacement. *Acta orthopaedica belgica*, 2015. http://cochranelibrary-wiley.com/o/cochrane/clcentral/articles/386/CN-01168386/frame.html (accessed.
314. Parker MJ. Randomised trial of blood transfusion versus a restrictive transfusion policy after hip fracture surgery. *Injury* 2013; **44**(12): 1916-8.
315. Pawar P, Kansal S, Chaudhary M, Baldha M, Makwana N, Makwana H. Comparative Study of Role of Pre-operative Injection Tranexamic Acid in 80 Cases of Transurethral Resection of Prostate. *International Journal of Scientific Study* 2016; **4**(2): 167-70.
316. Peters A, Verma K, Slobodyanyuk K, et al. Antifibrinolytics reduce blood loss in adult spinal deformity surgery: a prospective, randomized controlled trial. *Spine (Phila Pa 1976)* 2015; **40**(8): E443-9.
317. Prakash J, Seon JK, Park YJ, Jin C, Song EK. A randomized control trial to evaluate the effectiveness of intravenous, intra-articular and topical wash regimes of tranexamic acid in primary total knee arthroplasty. *J Orthop Surg (Hong Kong)* 2017; **25**(1): 2309499017693529.
318. Prasad R, Patki A, Padhy S, Ramchandran G. Single intravenous bolus versus perioperative continuous infusion of tranexamic acid to reduce blood loss in abdominal oncosurgical procedures: A prospective randomized double-blind clinical study. *Journal of Anaesthesiology Clinical Pharmacology* 2018; **34**(4): 529-34.
319. Raviraj A, Anand A, Chakravarthy M, Kumarswamy S, Prabhu A, Pai S. Tranexamic acid reduces blood loss in simultaneous bilateral total knee arthroplasty: a randomized control trial. *European journal of orthopaedic surgery & traumatology : orthopedie traumatologie*, 2012. http://cochranelibrary-wiley.com/o/cochrane/clcentral/articles/032/CN-00903032/frame.html https://link.springer.com/content/pdf/10.1007%2Fs00590-018-2147-5.pdf (accessed.
320. Roy SP, Tanki UF, Dutta A, Jain SK, Nagi ON. Efficacy of intra-articular tranexamic acid in blood loss reduction following primary unilateral total knee arthroplasty. *Knee Surg Sports Traumatol Arthrosc* 2012; **20**(12): 2494-501.
321. Sabry MM, Sallam AA, Elgebaly AS, Abdelwahab AA. Evaluation of local intra-pleural application of tranexamic acid on postoperative blood loss in lung decortication surgery, a prospective, randomized, double-blind, placebo-controlled study. *Annals of Cardiac Anaesthesia* 2018; **21**(4): 409-12.
322. Sadeghi M, Mehr-Aein A. Does a single bolus dose of tranexamic acid reduce blood loss and transfusion requirements during hip fracture surgery? a prospective randomezed double blind study in 67 patients. . 2007; **45**(6): 437-42.

323. Sa-Ngasoongsong P, Wongsak S, Chanplakorn P, et al. Efficacy of low-dose intra-articular tranexamic acid in total knee replacement; A prospective triple-blinded randomized controlled trial. *BMC Musculoskeletal Disorders* 2013; **14**.
324. Sarzaeem M, Razi M, Kazemian G, Moghaddam M, Rasi A, Karimi M. Comparing efficacy of three methods of tranexamic acid administration in reducing hemoglobin drop following total knee arthroplasty. *Journal of arthroplasty*, 2014. http://cochranelibrary-wiley.com/o/cochrane/clcentral/articles/733/CN-00998733/frame.html https://ac.els-cdn.com/S0883540314001454/1-s2.0-S0883540314001454-main.pdf?_tid=cdb0069d-86b0-4233-a6e5-35248f3d1c06&acdnat=1535708421_542223ea8b354ca3a8fc9f425f69d62a (accessed.
325. Schiavone A, Bisaccia M, Inkov I, et al. Tranexamic Acid in Pertrochanteric Femoral Fracture: Is it a Safe Drug or Not? *Folia medica* 2018; **60**(1): 67-78.
326. Scrascia G, Rotunno C, Nanna D, et al. Pump blood processing, salvage and re-transfusion improves hemoglobin levels after coronary artery bypass grafting, but affects coagulative and fibrinolytic systems. *Perfusion* 2012; **27**(4): 270-7.
327. Seol Y, Seon J, Lee S, et al. Effect of Tranexamic Acid on Blood Loss and Blood Transfusion Reduction after Total Knee Arthroplasty. *Knee surg relat res*, 2016. http://cochranelibrary-wiley.com/o/cochrane/clcentral/articles/605/CN-01306605/frame.html https://www.ncbi.nlm.nih.gov/pmc/articles/PMC5009042/pdf/ksrr-28-188.pdf (accessed.
328. Serrano-Trenas JA, Ugalde PF, Cabello LM, Chofles LC, Lazaro PS, Benitez PC. Role of perioperative intravenous iron therapy in elderly hip fracture patients: a single-center randomized controlled trial. *Transfusion* 2011; **51**(1): 97-104.
329. Seviciu A, Gross I, Fathima S, Walsh S. Effects of tranexamic acid and bipolar sealer alone or in combination in primary total knee arthroplasty: a prospective, randomized, controlled trial. *Arthroplasty today*, 2016. http://cochranelibrary-wiley.com/o/cochrane/clcentral/articles/775/CN-01177775/frame.html https://www.ncbi.nlm.nih.gov/pmc/articles/PMC4957169/pdf/main.pdf (accessed.
330. Shakeri M, Salehpour F, Shokouhi G, et al. Minimal Dose of Tranexamic Acid Is Effective in Reducing Blood Loss in Complex Spine Surgeries: A Randomized Double-Blind Placebo Controlled Study. *Asian spine journal* 2018; **12**(3): 484-9.
331. Shen P, Hou W, Chen J, Wang B, Qu Y. Effectiveness and safety of tranexamic acid for total knee arthroplasty: a prospective randomized controlled trial. *Medical science monitor*, 2015. http://cochranelibrary-wiley.com/o/cochrane/clcentral/articles/416/CN-01052416/frame.html https://www.ncbi.nlm.nih.gov/pmc/articles/PMC4347720/pdf/medscimonit-21-576.pdf (accessed.
332. Shen S, Zhang J, Wang W, Zheng J, Xie Y. Impact of intra-operative cell salvage on blood coagulation in high-bleeding-risk patients undergoing cardiac surgery with cardiopulmonary bypass: a prospective randomized and controlled trial. *Journal of Translational Medicine* 2016; **14**(1): 228.
333. Shi J, Ji H, Li L, et al. Protective effects of tranexamic acid on clopidogrel before coronary artery bypass grafting: A multicenter randomized trial. *JAMA Surgery* 2013; **148**(6): 538-47.
334. Shi J, Wang Y, Xue Q, Yuan S, Wang G, Li L. Effectiveness and safety of tranexamic acid in patients receiving on-pump coronary artery bypass grafting without clopidogrel and aspirin cessation. *Zhonghua wai ke za zhi [chinese journal of surgery]*, 2013. http://cochranelibrary-wiley.com/o/cochrane/clcentral/articles/760/CN-00999760/frame.html (accessed.
335. Shi H, Ou Y, Jiang D, Quan Z, Zhao Z, Zhu Y. Tranexamic acid reduces perioperative blood loss of posterior lumbar surgery for stenosis or spondylolisthesis a randomized trial. *Medicine (United States)* 2017; **96**(1).
336. Shinde A, Sobti A, Maniar S, Mishra A, Gite R, Shetty V. Tranexamic acid reduces blood loss and need of blood transfusion in total knee arthroplasty: A prospective, randomized, double-blind study in Indian population. *Asian journal of transfusion science* 2015; **9**(2): 168-72.
337. Song E, Seon J, Prakash J, Seol Y, Park Y, Jin C. Combined Administration of IV and Topical Tranexamic Acid is Not Superior to Either Individually in Primary Navigated TKA. *Journal of arthroplasty*, 2017. http://cochranelibrary-wiley.com/o/cochrane/clcentral/articles/828/CN-01424828/frame.html https://ac.els-cdn.com/S0883540316303461/1-s2.0-S0883540316303461-main.pdf?_tid=9ed81f39-beba-4682-b8be-3b7bc6c4b896&acdnat=1535708404_6431611c2346de4a371c39444c2c05fa (accessed.

338. So-Osman C, Van Hilten JA, Brand A, et al. Patient blood management in elective total hip- And knee-replacement surgery (Part 2): A randomized controlled trial on blood salvage as transfusion alternative using a restrictive transfusion policy in patients with a preoperative hemoglobin above 13 g/dl. *Anesthesiology* 2014; **120**(4): 852-60.
339. Spitler CA, Kiner DW, Row ER, et al. Tranexamic Acid Use in Open Reduction and Internal Fixation of Fractures of the Pelvis, Acetabulum, and Proximal Femur: A Randomized Controlled Trial. *Journal of orthopaedic trauma* 2019.
340. Sudprasert W, Tanaviriyachai T, Choovongkomol K, Jongkittanakul S, Piyapromdee U. A Randomized Controlled Trial of Topical Application of Tranexamic Acid in Patients with Thoracolumbar Spine Trauma Undergoing Long-Segment Instrumented Posterior Spinal Fusion. *Asian spine journal* 2019; **13**(1): 146-54.
341. Sun Q, Yu X, Wu J, Ge W, Cai M, Li S. Efficacy of a Single Dose and an Additional Dose of Tranexamic Acid in Reduction of Blood Loss in Total Knee Arthroplasty. *Journal of arthroplasty*, 2017. http://cochranelibrary-wiley.com/o/cochrane/clcentral/articles/061/CN-01455061/frame.html https://ac.els-cdn.com/S0883540316306957/1-s2.0-S0883540316306957-main.pdf?_tid=c62fd56e-03d4-400c-bc28-ee431e5b3307&acdnat=1535708562_8c7e5f59a64fdb9d0067117624c7df03 (accessed.
342. Taghaddomi RJ, Mashhadinezhad H, Attar ARS, Peivandi A. The Effect of Intravenous Tranexamic Acid on Blood Loss in Lumbar Hernial Disc Resection under Inhalation and Total Intravenous Anesthesia. *Iran Red Crescent Med J* 2009; **11**(3): 265-70.
343. Taksaudom N, Siwachat S, Tantraworasin A. Additional effects of topical tranexamic acid in on-pump cardiac surgery. *Asian cardiovascular & thoracic annals*, 2017. http://cochranelibrary-wiley.com/o/cochrane/clcentral/articles/046/CN-01263046/frame.html (accessed.
344. Tang J, Zhang ZX, Li X, Wang Y. Effects of tranexamic acid on the postoperative hemorrhage and complications after arthrolysis for elbow stiffness. *International Journal of Clinical and Experimental Medicine* 2018; **11**(3): 2278-84.
345. Tavares Sánchez-Monge FJ, Aguado Maestro I, Bañuelos Díaz A, Martín Ferrero MÁ, García Alonso MF. Efficacy and safety of the topical application of tranexamic acid in primary cementless hip arthroplasty: prospective, randomised, double-blind and controlled study. *Revista espanola de cirugia ortopedica y traumatologia* 2018; **62**(1): 47-54.
346. Thipparampall AK, Gurajala I, Gopinath R. The effect of different dose regimens of tranexamic acid in reducing blood loss during hip surgery. *Indian Journal of Anaesthesia* 2017; **61**(3): 235-9.
347. Tian S, Shen Z, Liu Y, Zhang Y, Peng A. The effect of tranexamic acid on hidden bleeding in older intertrochanteric fracture patients treated with PFNA. *Injury* 2018; **49**(3): 680-4.
348. Triyudanto A, Lubis A. The effects of intra-articular tranexamic acid given intraoperatively and intravenous tranexamic acid given preoperatively on post surgical bleeding and transfusion rate post total knee arthroplasty. *Medical journal of indonesia*, 2016. http://cochranelibrary-wiley.com/o/cochrane/clcentral/articles/846/CN-01331846/frame.html http://mji.ui.ac.id/journal/index.php/mji/article/download/1502/1153 (accessed.
349. Tzatzairis T, Drosos G, Kotsios S, Ververidis A, Vogiatzaki T, Kazakos K. Intravenous vs Topical Tranexamic Acid in Total Knee Arthroplasty Without Tourniquet Application: a Randomized Controlled Study. *Journal of arthroplasty*, 2016. http://cochranelibrary-wiley.com/o/cochrane/clcentral/articles/793/CN-01413793/frame.html https://ac.els-cdn.com/S0883540316301401/1-s2.0-S0883540316301401-main.pdf?_tid=c1cf8247-c1be-43a5-8eb8-b648f5d9ae11&acdnat=1535708633_33c9ca2097e224119c1e8cbb6aaf83a5 (accessed.
350. Vijay B, Bedi V, Mitra S, Das B. Role of tranexamic acid in reducing postoperative blood loss and transfusion requirement in patients undergoing hip and femoral surgeries. *Saudi journal of anaesthesia*, 2013. http://cochranelibrary-wiley.com/o/cochrane/clcentral/articles/356/CN-00907356/frame.html http://www.saudija.org/article.asp?issn=1658-354X;year=2013;volume=7;issue=1;spage=29;epage=32;aulast=Vijay (accessed.
351. Volquind D, Zardo R, Winkler B, Londero B, Zanelatto N, Leichtweis G. Use of tranexamic acid in primary total knee replacement: effects on perioperative blood loss. *Brazilian journal of anesthesiology (elsevier)*, 2016. http://cochranelibrary-wiley.com/o/cochrane/clcentral/articles/948/CN-01368948/frame.html https://ac.els-cdn.com/S0104001415000482/1-s2.0-S0104001415000482-main.pdf?_tid=25b52123-475c-43bc-9609-fdfc4125e328&acdnat=1535708835_3c7e0ba9c17b5d1f0e6c8405059f47db (accessed.

352. Wang G, Wang Y, Wang W, et al. Tranexamic acid reduces blood loss after off-pump coronary surgery: A prospective, randomized, double-blind, placebo-controlled study. *Anesthesia and Analgesia* 2012; **115**(2): 239-43.
353. Wang Q, Liu J, Chen Y, et al. Tranexamic acid reduces postoperative blood loss of degenerative lumbar instability with stenosis in posterior approach lumbar surgery: A randomized controlled trial. *European Spine Journal* 2013; **22**(9): 2035-8.
354. Wang C, Sun Z, Liu J, Cao J, Li Z. Safety and efficacy of intra-articular tranexamic acid injection without drainage on blood loss in total knee arthroplasty: a randomized clinical trial. *International journal of surgery (london, england)*, 2015. http://cochranelibrary-wiley.com/o/cochrane/clcentral/articles/749/CN-01257749/frame.html https://ac.els-cdn.com/S1743919115002599/1-s2.0-S1743919115002599-main.pdf?_tid=9340e556-027f-47b6-a199-403d80c25e52&acdnat=1535708728_21c2c1f528ec17c68d6a1cf0a68c2eaf (accessed.
355. Wang G, Wang D, Sun S, Wang B, Lin Y. Efficacy and safety evaluation of intra-articular injection of tranexamic acid in total knee arthroplasty operation with temporarily drainage close. *International Journal of Clinical and Experimental Medicine* 2015; **8**(8): 14328-34.
356. Wang R, Tian SQ, Ha CZ, Sun K, Song RX. Efficacy and safety of tranexamic acid on reducing blood loss in bilateral total knee arthroplasty. *Chinese Journal of Tissue Engineering Research* 2015; **19**(22): 3451-6.
357. Wang C, Kang P, Ma J, Yue C, Xie J, Pei F. Single-dose tranexamic acid for reducing bleeding and transfusions in total hip arthroplasty: a double-blind, randomized controlled trial of different doses. *Thrombosis research*, 2016. http://cochranelibrary-wiley.com/o/cochrane/clcentral/articles/995/CN-01140995/frame.html https://ac.els-cdn.com/S004938481630055X/1-s2.0-S004938481630055X-main.pdf?_tid=9ab6ee6f-a8db-4344-b26f-a4f63c930f63&acdnat=1535708737_c16456c29371bef04837a1aeb51200a0 (accessed.
358. Wang J, Chen B, Lin P, Yen S, Huang C, Kuo F. The Efficacy of Combined Use of Rivaroxaban and Tranexamic Acid on Blood Conservation in Minimally Invasive Total Knee Arthroplasty a Double-Blind Randomized, Controlled Trial. *Journal of arthroplasty*, 2017. http://cochranelibrary-wiley.com/o/cochrane/clcentral/articles/821/CN-01424821/frame.html https://ac.els-cdn.com/S0883540316305228/1-s2.0-S0883540316305228-main.pdf?_tid=71093264-d999-45ec-8b5a-2bc593d25346&acdnat=1535708576_39d57612f9dec6ed888c85c40c9ee463 (accessed.
359. Wang J, Wang Q, Zhang X. Intra-articular Application is More Effective Than Intravenous Application of Tranexamic Acid in Total Knee Arthroplasty: A Prospective Randomized Controlled Trial. *Journal of Arthroplasty* 2017; **32**(11): 3385-9.
360. Wang D, Luo ZY, Pei FX, et al. Effect of Multiple Doses of Oral Tranexamic Acid on Haemostasis and Inflammatory Reaction in Total Hip Arthroplasty: A Randomized Controlled Trial. *Thrombosis and Haemostasis* 2019; **119**(1): 92-103.
361. Wei W, Wei B. Comparison of topical and intravenous tranexamic acid on blood loss and transfusion rates in total hip arthroplasty. *Journal of arthroplasty*, 2014. http://cochranelibrary-wiley.com/o/cochrane/clcentral/articles/471/CN-01022471/frame.html https://ac.els-cdn.com/S0883540314004872/1-s2.0-S0883540314004872-main.pdf?_tid=5c2d7ea2-cd72-4fab-ac31-2a927571816d&acdnat=1535708437_153ccc3149d1e267f32b4fde7844c9d3 (accessed.
362. Wiefferink A, Weerwind PW, van Heerde W, et al. Autotransfusion management during and after cardiopulmonary bypass alters fibrin degradation and transfusion requirements. *Journal of Extra-Corporeal Technology* 2007; **39**(2): 66-70.
363. Xie Y, Shen S, Wang W, Zhang J, Zheng J. The efficacy, safety and cost-effectiveness of intra-operative cell salvage in high-bleeding-risk cardiac surgery with cardiopulmonary bypass: A prospective randomized and controlled trial. *International Journal of Medical Sciences* 2015; **12**(4): 322-8.
364. Xie B, Tian J, Zhou D-P. Administration of Tranexamic Acid Reduces Postoperative Blood Loss in Calcaneal Fractures: a Randomized Controlled Trial. *Journal of foot and ankle surgery*, 2015. http://cochranelibrary-wiley.com/o/cochrane/clcentral/articles/669/CN-01104669/frame.html https://ac.els-cdn.com/S1067251615003087/1-s2.0-S1067251615003087-main.pdf?_tid=a5116a77-0782-48e6-8d9f-1dad2c9aa7f3&acdnat=1535708867_b4c80a0fbc21cde0f114e8ac992ad6f8 (accessed.
365. Xu D, Zhuang Q, Li Z, Ren Z, Chen X, Li S. A randomized controlled trial on the effects of collagen sponge and topical tranexamic acid in posterior spinal fusion surgeries. *Journal of orthopaedic surgery and research* 2017; **12**(1): 166-.

366. Yanartas M, Aydin E, Cevirme D, et al. The effects of tranexamic acid and 6% hydroxyethyl starch (HES) solution (130/0.4) on postoperative bleeding in coronary artery bypass graft (CABG) surgery. *International Journal of Clinical and Experimental Medicine* 2015; **8**(4): 5959-71.

367. Yang Y, Lv Y, Ding P, Li J, Ying-Ze Z. The reduction in blood loss with intra-articular injection of tranexamic acid in unilateral total knee arthroplasty without operative drains: a randomized controlled trial. *European journal of orthopaedic surgery & traumatology : orthopedie traumatologie*, 2015. http://cochranelibrary-wiley.com/o/cochrane/clcentral/articles/811/CN-01000811/frame.html https://link.springer.com/content/pdf/10.1007%2Fs00590-018-2147-5.pdf (accessed.

368. Yen SH, Lin PC, Wang JW, Chen B, Huang CC. Topical tranexamic acid reduces blood loss in minimally invasive total knee arthroplasty receiving rivaroxaban. *BioMed Research International* 2017; **2017**.

369. Yuan X, Li B, Wang Q, Zhang X. Comparison of 3 Routes of Administration of Tranexamic Acid on Primary Unilateral Total Knee Arthroplasty: A Prospective, Randomized, Controlled Study. *Journal of Arthroplasty* 2017; **32**(9): 2738-43.

370. Yue C, Kang P, Yang P, Xie J, Pei F. Topical application of tranexamic acid in primary total hip arthroplasty: a randomized double-blind controlled trial. *Journal of arthroplasty*, 2014. http://cochranelibrary-wiley.com/o/cochrane/clcentral/articles/493/CN-01037493/frame.html https://ac.els-cdn.com/S0883540314002204/1-s2.0-S0883540314002204-main.pdf?_tid=39182dd9-6e10-42cb-8dac-f8ff4f50ca89&acdnat=1535708758_b405eb9c61a89fccdd664d956ce6bc4e (accessed.

371. Zekcer A, Priori RD, Tieppo C, Silva RSd, Severino NR. Comparative study of topical vs. intravenous tranexamic acid regarding blood loss in total knee arthroplasty. *Revista brasileira de ortopedia* 2017; **52**(5): 589-95.

372. Zeng Y, Si H, Shen B, et al. Intravenous Combined with Topical Administration of Tranexamic Acid in Primary Total Hip Arthroplasty: a Randomized Controlled Trial. *Orthopaedic surgery*, 2017. http://cochranelibrary-wiley.com/o/cochrane/clcentral/articles/959/CN-01394959/frame.html https://onlinelibrary.wiley.com/doi/pdf/10.1111/os.12287 (accessed.

373. Zhang F, Gao Z, Yu J. [Clinical comparative studies on effect of tranexamic acid on blood loss associated with total knee arthroplasty]. *Zhongguo Xiu Fu Chong Jian Wai Ke Za Zhi* 2007; **21**(12): 1302-4.

374. Zhang CH, Liu Y, Zhao JN, Meng J, Yuan T, Ni-Rong B. Intravenous drip and topical application using tranexamic acid decrease hidden blood loss after total hip arthroplasty. *Chinese Journal of Tissue Engineering Research* 2015; **19**(44): 7071-6.

375. Zhang Y, Zhang L, Ma X, et al. What is the optimal approach for tranexamic acid application in patients with unilateral total hip arthroplasty? *Der orthopade*, 2016. http://cochranelibrary-wiley.com/o/cochrane/clcentral/articles/254/CN-01414254/frame.html https://link.springer.com/content/pdf/10.1007%2Fs00132-016-3252-y.pdf (accessed.

376. Zhou K-d, Wang H-Y, Wang Y, Liu Z-H, He C, Feng J-M. Is topical or intravenous tranexamic acid preferred in total hip arthroplasty? A randomized, controlled, noninferiority clinical trial. *PloS one* 2018; **13**(10): e0204551.

377. Dryden PJ, O'Connor JP, Jamieson WR, et al. Tranexamic acid reduces blood loss and transfusion in reoperative cardiac surgery. *Can J Anaesth* 1997; **44**(9): 934-41.

378. Johnson RG, Thurer RL, Kruskall MS, et al. Comparison of two transfusion strategies after elective operations for myocardial revascularization. *Journal of Thoracic & Cardiovascular Surgery* 1992; **104**(2): 307-14.

379. Murphy GJ, Pike K, Rogers CA, et al. Liberal or Restrictive Transfusion after Cardiac Surgery. *New England Journal of Medicine* 2015; **372**(11): 997-1008.

380. Nielsen K, Johansson PI, Dahl B, et al. Perioperative transfusion threshold and ambulation after hip revision surgery – a randomized trial. *BMC Anesthesiology* 2014; **14**(1): 89.

381. Karkouti K, Callum J, Wijeysundera DN, et al. Point-of-Care Hemostatic Testing in Cardiac Surgery: A Stepped-Wedge Clustered Randomized Controlled Trial. *Circulation* 2016; **134**(16): 1152-62.
